# Transition-Metal-Based Dearomatization Strategy for the Synthesis of Functionalized *cis*-Tetrahydro-2-Oxindoles

**DOI:** 10.1021/acs.organomet.5c00371

**Published:** 2025-10-28

**Authors:** Paolo Siano, Louis A. Diment, Daniel J. Siela, Megan N. Ericson, Matt McGraw, Brian C. Song, Benjamin F. Livaudais, Spenser R. Simpson, Diane A. Dickie, W. Dean Harman

**Affiliations:** Department of Chemistry, 2358 University of Virginia, Charlottesville, Virginia 22904, United States

## Abstract

*cis*-Tetrahydro-2-oxindoles are valuable scaffolds in medicinal chemistry. Herein, we report a transition-metal-mediated dearomatization strategy to access these compounds. The process begins with the coordination of a phenyl sulfone by a tungsten complex designed to bind two carbons of the phenyl ring, rendering it dearomatized. This is followed by protonation of the η^2^-bound arene followed by the addition of an ester nucleophile. The resulting η^2^-diene complex then undergoes a second protonation in the ring, and a primary amine is introduced. Dihydro-2-oxindole complexes form spontaneously through the construction of a γ-lactam and the elimination of a sulfinic acid. Dihydro-2-oxindoles are practically unknown—presumably owing to their ability to form indolines—but coordination to tungsten stabilizes these intermediates. The terminal position of the coordinated diene (C4 of the 2-oxindole core) can then be protonated to generate an η^2^-allyl complex, which undergoes nucleophilic addition with C-, N-, or S-type nucleophiles to form the corresponding tetrahydro-2-oxindole complexes. Finally, the organic ligand is obtained through the oxidative decomplexation of the metal. This methodology provides a modular approach for accessing 1,2,5-functionalized *cis*-2-oxindole compounds.

## Graphical abstract

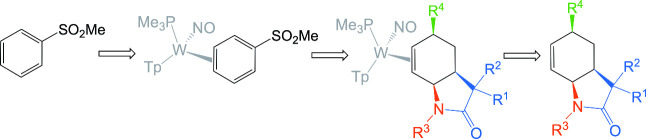

## Introduction

Perhydroindoles (octahydroindoles) represent a major class of natural products and pharmaceuticals.^1^ Examples include *Sceletium* alkaloids such as the serotonin reuptake inhibitor mesembrine, the serine protease inhibitor Dysinosin A,^2^ the *Aeruginosins*,^3,4^ the stenine group of *Stemona* alkaloids,^5^ and the popular ACE inhibitor perindopril. Perhydroindoles are commonly synthesized via various ring-closure strategies (Figure 1A) including amine-tethered cyclohexenes or cyclohexenones,^4,6^ intramolecular Diels–Alder reactions,^7^ aza-Prins cyclizations,^8^ and RCM of diallylpyrrolidines. Perhydro-2-oxindoles (hexahydro-2-oxindoles) are also attractive intermediates in the preparation of perhydroindoles.^9–11^ Common methods for their synthesis include metal-catalyzed annulation reactions^4,12–14^ and the iridium-catalyzed photocyclization of cyclohexenamides (Figure 1A).^15,16^ Alternatively, transition-metal-catalyzed hydrogenation of indoles or 2-oxindoles has been proven to be a reliable method for the *cis*-isomer of perhydroindoles and perhydrooxindoles (Figure 1A).^17–20^

**Figure 1:**
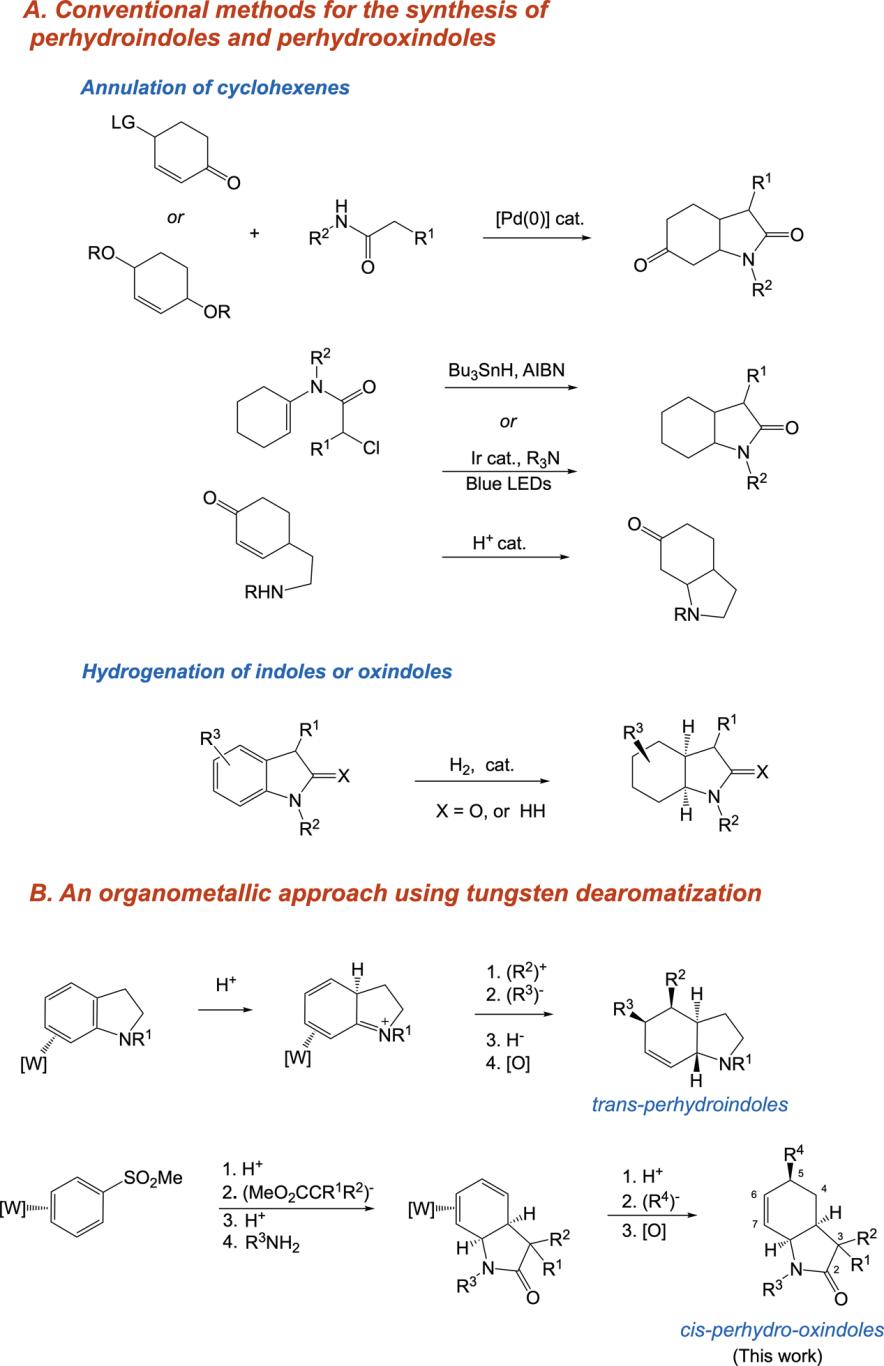
(A) Conventional methods for the synthesis of perhydroindole and perhydro-2-oxindole skeletons including annulations of cyclohexenes and hydrogenations of indoles and oxindoles. (B) Approaches to perhydroindole and perhydro-2-oxindole based on transition-metal dearomatization.

Over the past decade, we have explored several organometallic approaches to the perhydroindole core.^21^ In one study, we found that indolines could be coordinated to the dearomatization agent {WTp­(NO)­(PMe_3_)}, (Tp = hydridotris­(pyrazolyl)­borate) and elaborated into various hexahydroindoles via a proton/electrophile/nucleophile/hydride addition sequence (Figure 1B). Since protonation and hydride addition occur on opposite faces of the core,^21^ a trans-fused ring system results. Herein, we set out to find a complementary organometallic approach to these important heterobicyclics that could lead to *cis*-fused-2-oxindoles. Our approach leverages the ability of {WTp­(NO)­(PMe_3_)} to construct the 2-oxindole core from a dihapto-coordinated benzene (Figure 1).^22–24^ Using a phenyl sulfone as the arene, various functionalized *cis*-dihydro-2-oxindole complexes can be prepared via the elimination of sulfinic acid. This metal-stabilized dihydro-2-oxindole can be elaborated into various C5-functionalized *cis-*tetrahydro-2-oxindoles (Figure 1B).

## Results and Discussion

### Formation of *cis*-Dihydro-2-oxindole Complexes

The synthesis of *cis*-fused bicyclic γ-lactams from compounds of the form WTp­(NO)­(PMe_3_)­(η^2^-arene) (arene = benzene or trifluorotoluene (TFT)) has been previously reported,^22–24^ including in the synthesis of γ-lycorane.^24^ While these methods allow for the diversification of the heterocyclic ring, they lack the ability to functionalize the carbocyclic ring. We reasoned that if methyl phenyl sulfone was employed as the arene (**1** in Figure 2), then the SO_2_Me group would not only stabilize the arene complex but could serve as a facile leaving group, the elimination of which could allow the formation of various *cis*-dihydro-2-oxindole complexes (**8** in Figure 2). As described previously,^25^ the phenylsulfone complex **1** protonates at C4, ortho to the withdrawing group to form **2** (Figure 2). Addition of an ester enolate (R^1^ = R^2^ = H; R^1^ = R^2^ = Me; R^1^ = H, R^2^ = COOMe) forms the sulfonated diene complex **3**, isolated as a single coordination diastereomer with the sulfonyl group oriented toward the PMe_3_ group (**4**).^25^ At this point, the π-basic metal facilitates the protonation of the sulfonyldiene ligand at C5, the carbon bearing the SO_2_Me group. While the initial protonation stereochemistry is uncertain, complexes of the form **4** can be obtained with the sulfonyl group oriented trans to the metal.^25^ Subsequently, various primary amines (H_2_NR^3^) can be added (**5** in Figure 2). While this addition is reversible,^24^ we found that by allowing the reaction mixture to stir (first at −30 °C for 18 h and then at ambient temperature for 18 h), the corresponding γ-lactam complex **6** could be formed, analogous to what was first observed for the CF_3_ analogue.^23^ When the amine used in the lactamization is tethered to an additional nucleophilic heteroatom, various tricyclic species can be produced through the displacement of the sulfinate group.^26^ However, for the purposes of this study, we sought to eliminate HSO_2_Me via an allyl intermediate of type **7**,^25^ thereby generating *cis*-dihydro-2-oxindole complexes of type **8**. This overall conversion of **3** → **8** was then optimized as a single-pot process for 18 different primary amines and 3 different esters, with overall yields ranging from 51 to 89% (**9**–**26**; Figure 3).

**Figure 2:**
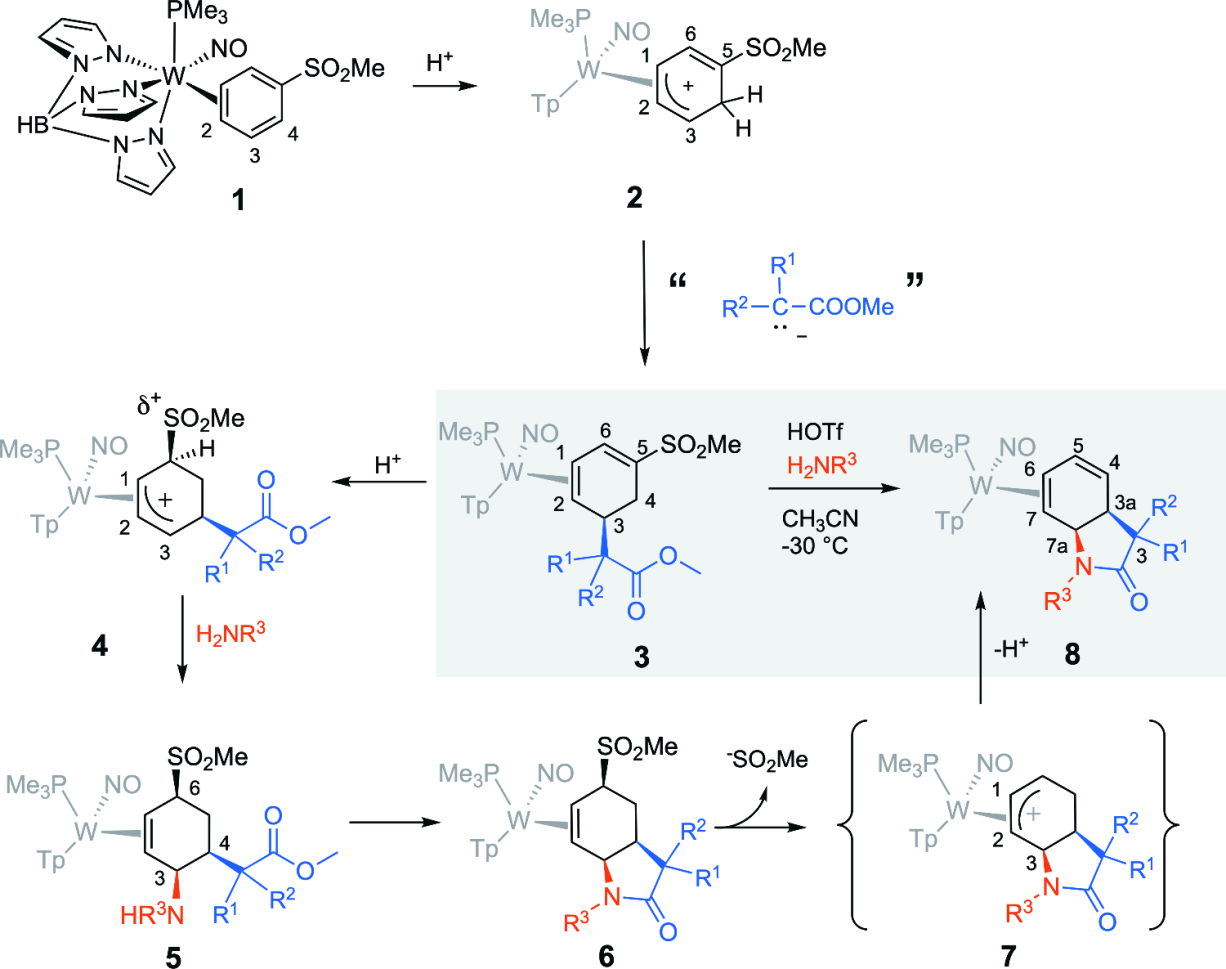
Reaction Scheme summarizing the synthesis of dihydro-2-oxindole complexes.

**Figure 3:**
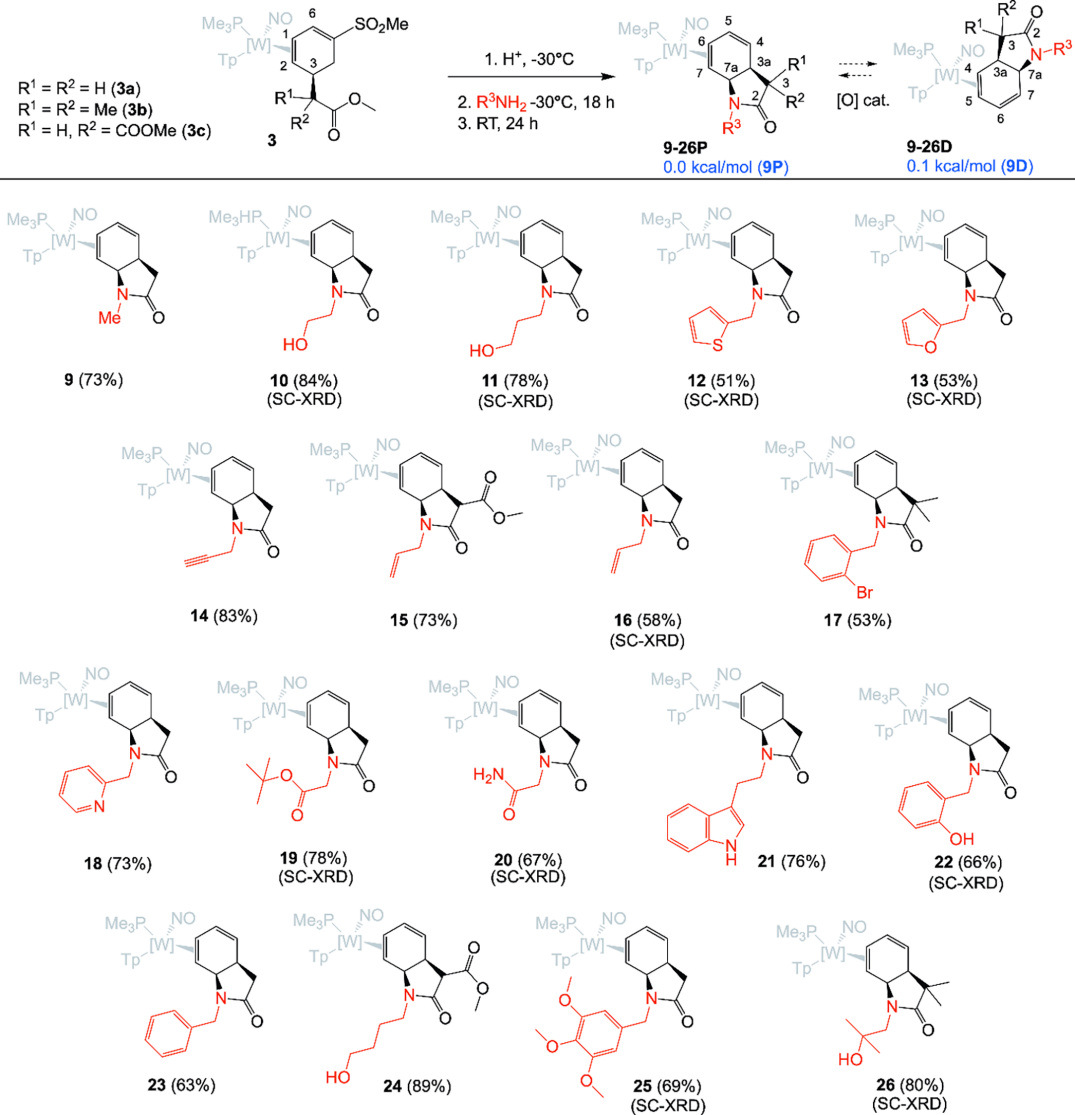
One-pot syntheses of bicyclic γ-lactam-diene complexes. SC-XRD: structure confirmed by single-crystal X-ray diffraction. Compounds **9**–**26** were isolated with ir >20:1 as their proximal isomers (**P**); however, extended exposure to oxygen or Lewis acids induces their partial isomerization to the distal form (**D**). All complexes depicted are racemic mixtures.

2D NMR data were used to confirm the connectivity and stereochemistry of compounds **9**–**26**, and single-crystal X-ray diffraction (SC-XRD) data confirmed this *cis*-fused ring for **10**–**13**, **16**, **19**, **20**, **22**, **25**, and **26** (Supporting Information). Key spectroscopic features include NOE interactions between H5 and the PMe_3_, NOE interaction between H7a and Tp3A, and a diagnostic set of peaks at 6.5 ppm (m, H5) and 4.5 ppm (d, H4), characteristic of analogous η^2^-diene complexes previously reported (Supporting Information).^25^

### Formation of η^2^-Allyl Lactam Complexes

While organic 3a,7a-dihydro-2-oxindoles are practically unknown, perhaps because of their susceptibility to aromatization, complexes in Figure 3 were stable as solids under a nitrogen atmosphere at temperatures up to 200 °C, with no observable decomposition. However, extended exposure to air or Lewis acids (e.g., silica) induced a partial linkage isomerization,^27^ in which the {TpW­(PMe_3_)­NO} fragment shifted to the other double bond, from C6–C7­(**P** = diene proximal) to C4–C5 (**D** = diene distal). This isomerization was incomplete, with the final equilibrium ratio of the two constitutional isomers being close to 1:1 in several cases. Supporting this observation, DFT analysis found that the distal (**9D**) and proximal (**9P**) forms of the *N*-methyl derivative were separated by a free energy of only 0.1 kcal/mol. In the case of *N*-allyl derivative **16**, we were able to grow crystals of each of the two isomers (Figure 4). While the diene complexes in Figure 3 were all isolated as single (proximal) isomers (ir >20:1 for **9**–**26**), this redox-catalyzed linkage isomerization^27^ posed potential difficulties for the regioselective functionalization of the carbocycle (vide infra). To overcome this obstacle, we treated compounds **9**, **10**, **15**, **16**, **21**–**24**, and **26** with strong acid (HOTf, 1 M) in acetonitrile and induced the precipitation of the corresponding allyl complex in ether as a triflate salt. By this method, we were able to isolate compounds **27**–**35** (Figure 5). Allyl complexes of {WTp­(NO)­(PMe_3_)} are present in solution as two dominant conformers, primarily differing in C–W bond lengths of the terminal allyl carbons.^28^ These “η^2^-allyl” species were observed to be stable as solids for up to 3 months under a nitrogen atmosphere, but attempts to purify them largely resulted in their decomposition. In the case of **27**, 2D NMR data was used to confirm the structure, but other allyl complexes (**28**–**35**) were carried out without full characterization.

**Figure 4:**
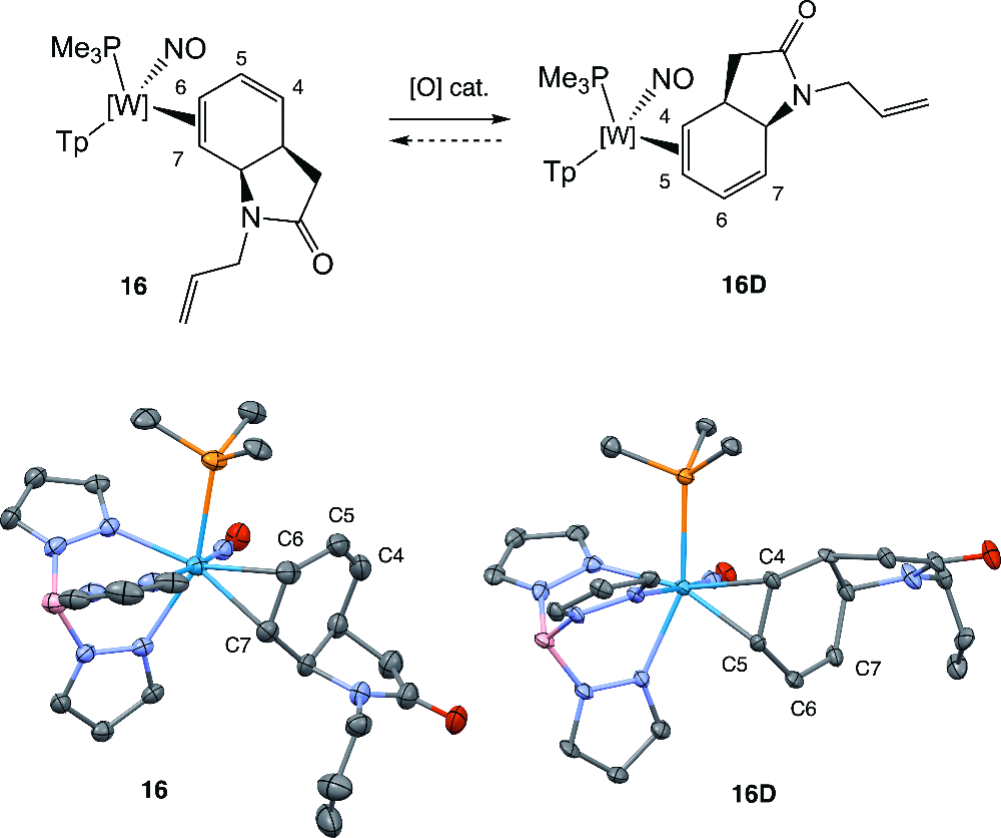
Redox-catalyzed linkage isomerization of the bicyclic lactam **16** (i.e., **16P**) from a proximal η^2^-bound diene to a distal η^2^-bound diene complex (**16D**). [O] = exposure to air. SC-XRD molecular structures were represented with 35% ellipsoids.

**Figure 5:**
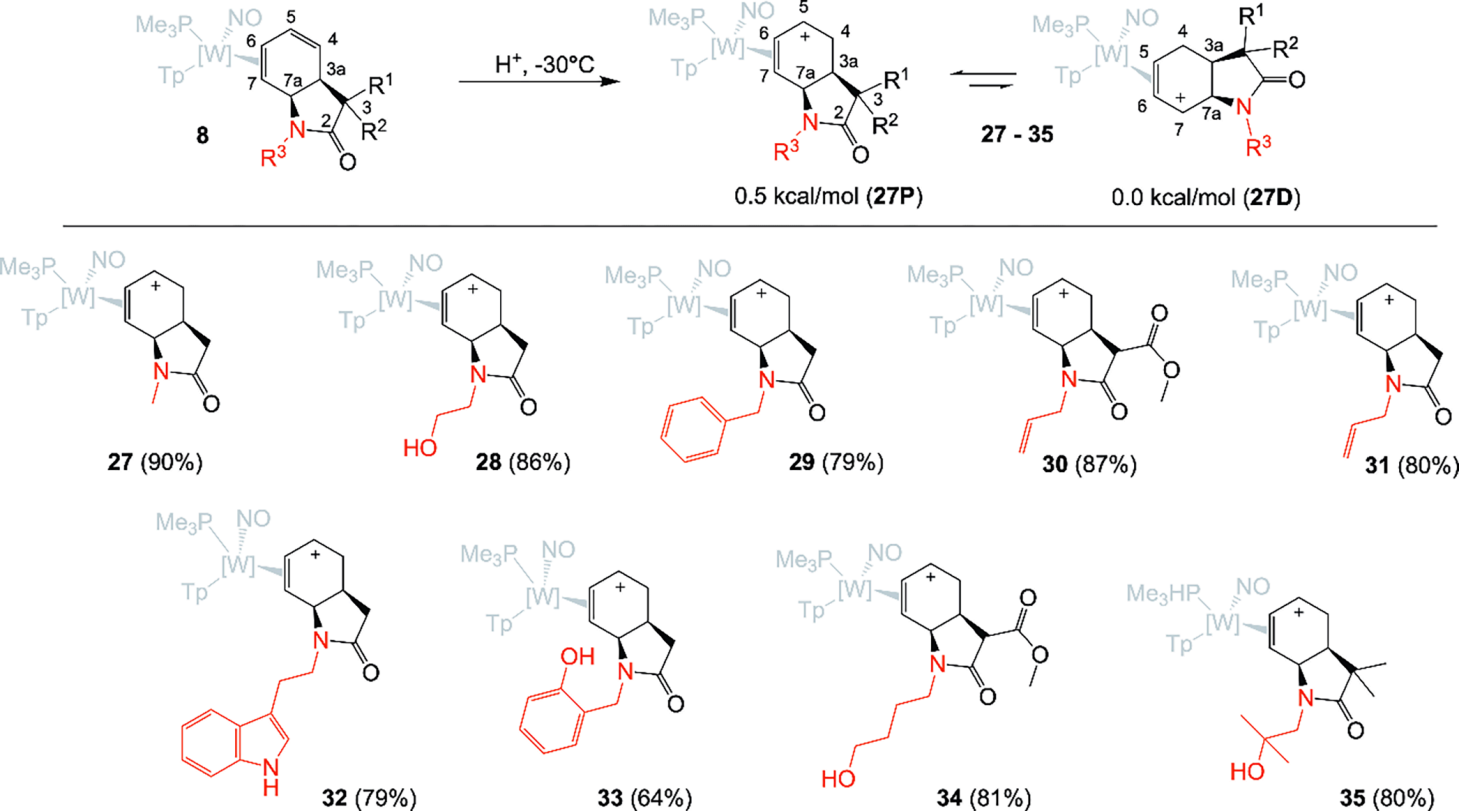
Synthesis of **27**–**35** (shown as their proximal η^2^-allyl conformers) by treatment of η^2^-diene complexes of type **8** with excess acid (HOTf, 1 M) in acetonitrile. All complexes depicted are racemic mixtures.

### Synthesis of Tetrahydro-2-oxindole Complexes

To add another point of diversity to these 2-oxindole cores, we sought to develop a protocol for functionalizing C5 using various C, N, and S nucleophiles (Figure 6). Addition of a collection of nucleophiles to the *N*-methylated η^2^-allyl complex **27** resulted in complexes **36**–**41**. In all cases, despite a slight preference for the distal allyl conformation (e.g., **27D**; favored by 0.5 kcal/mol),^28^ addition occurs exclusively at C5 rather than C7, even in the case of the relatively small nucleophile CN^–^ (**39**). DFT calculations for the corresponding tetrahydro-2-oxindole indicate no significant thermodynamic preference for **39** over its constitutional isomer, suggesting that the selectivity for C5 is entirely based on kinetics. We attribute this selectivity to the steric factors associated with a nucleophilic addition adjacent and syn to the lactam nitrogen.

**Figure 6:**
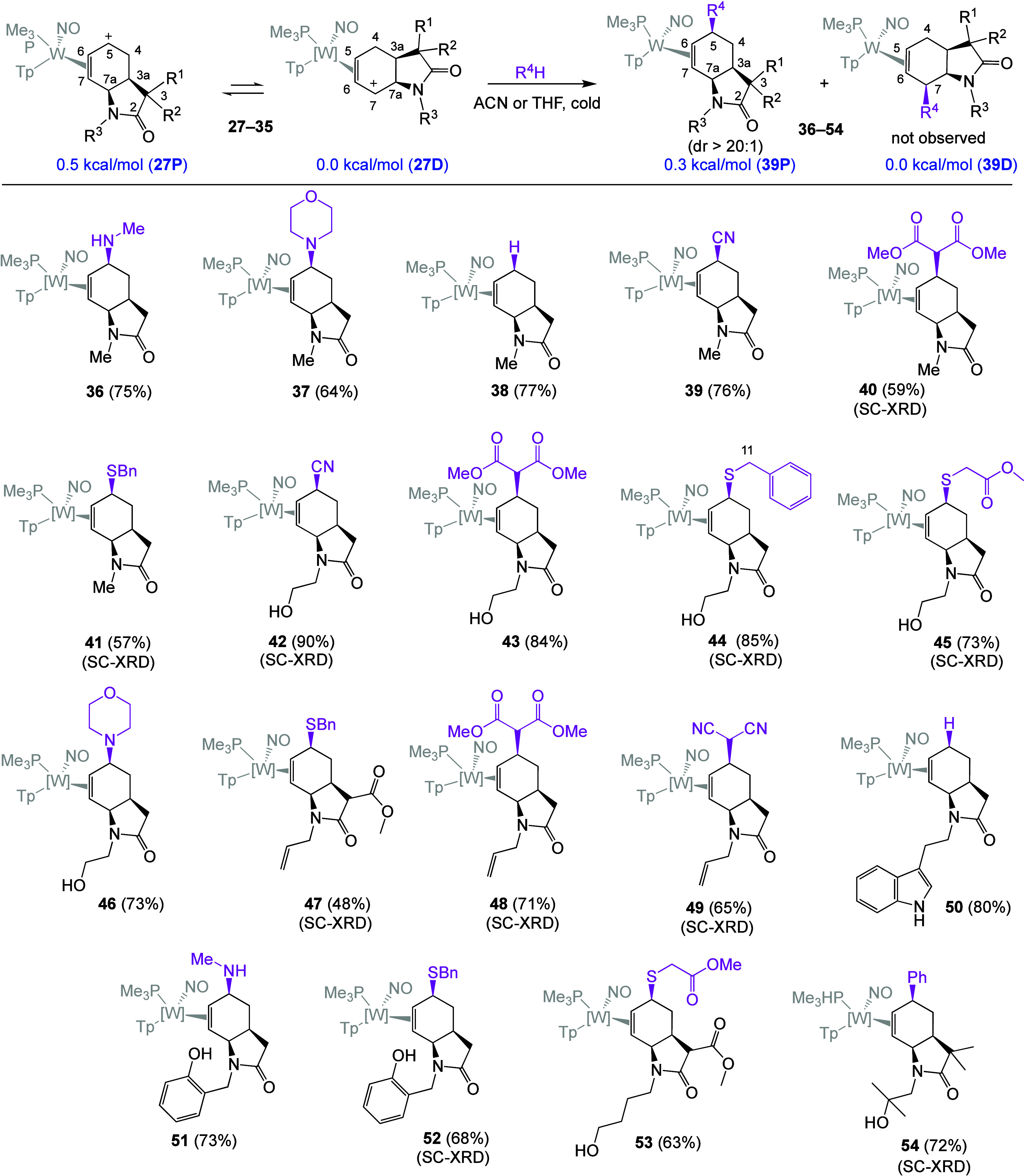
Selective addition of a nucleophile to η^2^-allyl γ-lactam complexes. SC-XRD: structure confirmed by single-crystal X-ray diffraction. All complexes depicted are racemic mixtures.

Additions of amines such as methylamine or morpholine resulted in η^2^-allylamine complexes, which are acid-sensitive.^24^ Hence, a base quench (e.g., trace KO^*t*^B_u_) was needed to prevent elimination back the original diene complex (i.e., **9**). Sulfur nucleophiles needed to be deprotonated with a base to increase their nucleophilicity (generally KO^*t*^B_u_). Compounds derived from carbon nucleophiles or hydrides were purified through a water-based extraction procedure (H_2_O: DCM). These nucleophilic addition reactions were generally successful with other η^2^-allyl complexes (**28**–**35**) as well. A full 2D NMR analysis confirmed the structures of all the compounds shown in Figure 6 (**36**–**54**); SC-XRD data was successfully obtained for **40**–**42**, **44**, **45**, **47**–**49**, **52**, and **54**. Key spectroscopic features again included strong NOE interactions between H5 (now a triplet at ∼3.7 ppm) and the PMe_3_ ligand.

### Liberation of the Tetrahydro-2-oxindole from the Metal

To liberate the synthesized tetrahydro-2-oxindoles from the metal fragment, different approaches were employed. In most cases, NOPF_6_ effectively liberated the organic products. In the case of the amine derivatives **55** and **65**, low temperature and short reaction time were essential to avoid overoxidation. When the compound contained sulfur, 2,3-dichloro-5,6-dicyano-1,4-benzoquinone (DDQ) was shown to be the most effective in delivering the decomplexed product (**60**–**62** and **66**; Figure 7). Purified product yields ranged from 53 to 80%. The closest general methods to compounds similar to those in Figure 7 are those available through hydrogenation of 5-substituted 2-oxindole derivatives, which share the common feature of all-cis stereochemistry.^17^ Using the hydrogenation protocol, a different 2-oxindole would need to be synthesized for each hexahydro-2-oxindole targeted, and the carbocycle would be completely saturated. Compounds **55**–**66** have the advantage of the alkene functional group, which could provide additional points of diversity.

**Figure 7:**
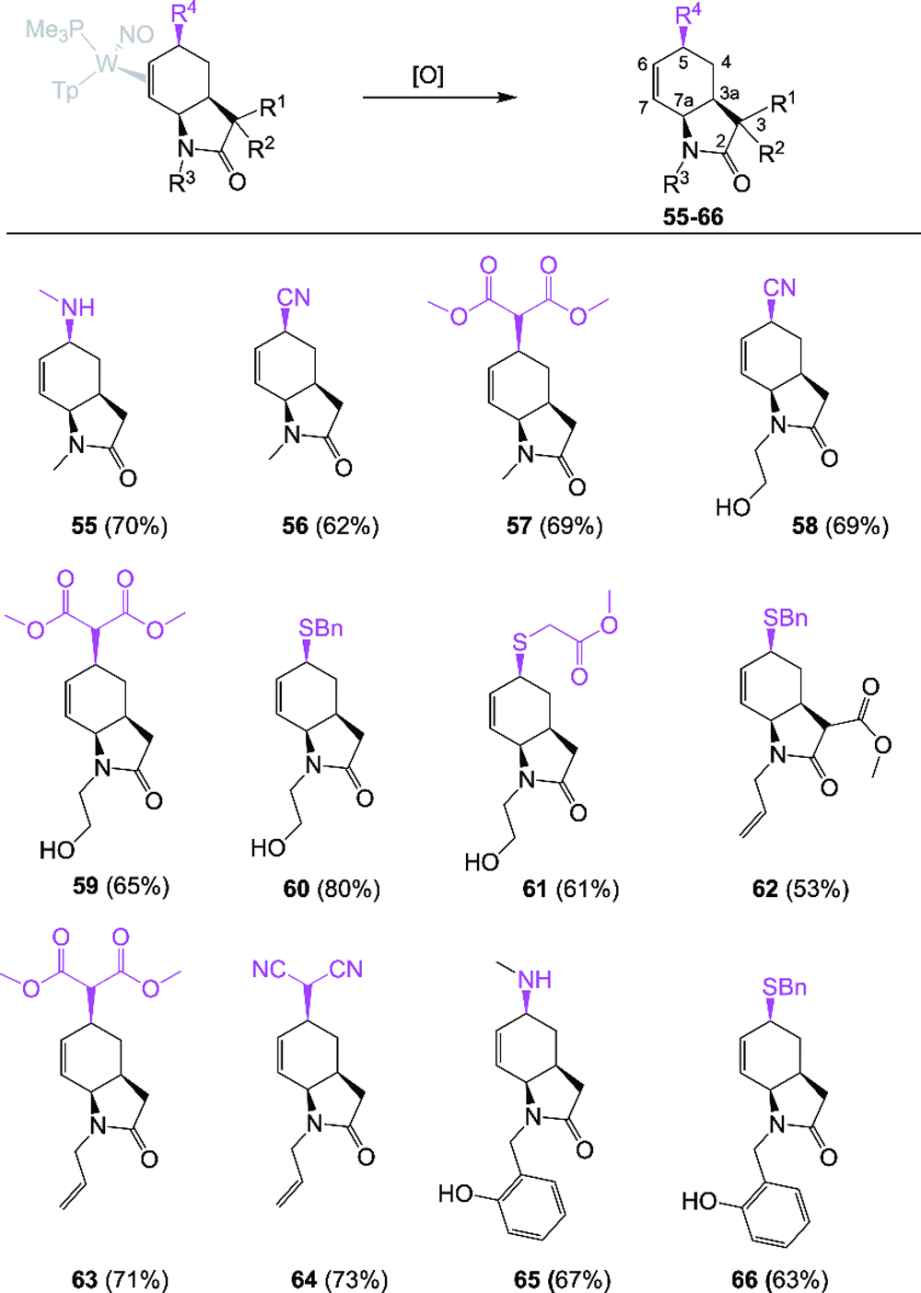
Liberation of organic tetrahydro-2-oxindoles **55**–**66** using various oxidants: [O] = DDQ (**60**–**62** and **66**); [O] = NOPF_6_ for all others. All compounds depicted are racemic mixtures.

### Extensions

As discussed earlier, the perhydroindole and perhydro-2-oxindole cores are widely represented in nature.^1–5^ The methodology outlined herein is useful, not so much as a tool to recreate these natural products but rather as a method to rapidly build small, diverse libraries of perhydroindoles and perhydrooxindoles as potential new lead compounds. Of the 12 tetrahydro-2-oxindoles prepared as examples of this methodology (**55**–**66**), nine (**57**, **58**, and **60**–**66**) meet the criteria of Lipinski’s rule of five^29^ for evaluating drug likeliness, as well as the criteria of Ghose et al.,^30^ Veber et al.,^31^ Egan et al.,^32^ and Muegge et al.^33^ In addition, six (**57**, **59**–**61**, **63**, and **65**) meet the qualification for leadlikeness popularized by Teague et al.^34^ and all but two (**65** and **66**) show no structural features connected to pan-assay interference compounds (PAINS).^35^ The methodology has high tolerance of functional groups including amines, thiols, phenols, alcohols, amides, and esters, with virtually no requirement for specialized protecting groups (beyond the metal itself). Additionally, this methodology does not require the use of halides or precious metals. To illustrate its potential to prepare diverse, biologically relevant compounds, we explored several additional ways this chemistry could be implemented in Figure 8. In the first example, the *N*-benzylated dihydro-2-oxindole is elaborated into a C5-aminated oxindole complex (**68**), which is then converted into an acrylamide-based covalent probe (**70**).^36^ Alternatively, the dihydrophenylsulfone complex **71** was prepared as an analogue to **3** (Figure 2) and cyclized to form a *cis*-bicyclic δ-lactam (**72** in Figure 8), similar to **8** (Figure 2). This compound was then elaborated into the acrylamide complex **74** (Figures 8 and 9) and then decomplexed to provide **75.**

**Figure 8:**
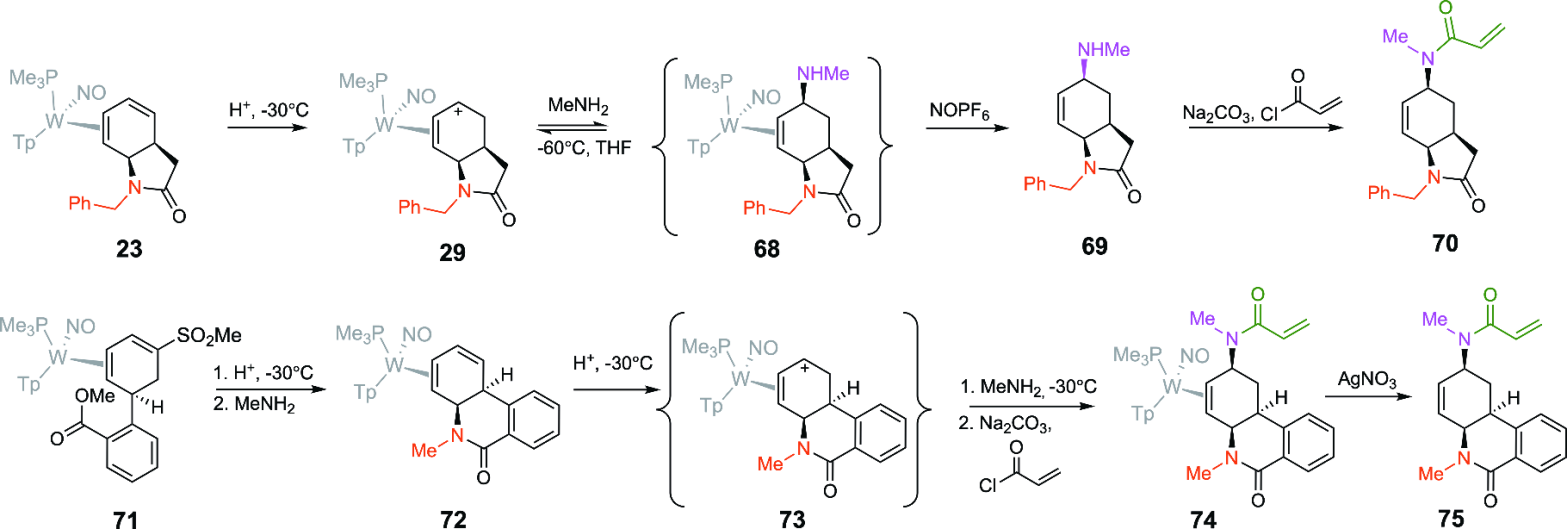
Several extensions to the diene lactam chemistry are shown in Figures 2–6. These include elaboration into acrylamide probes and development of the tetrahydrophenanthridinone core. All compounds depicted are racemic mixtures.

**Figure 9:**
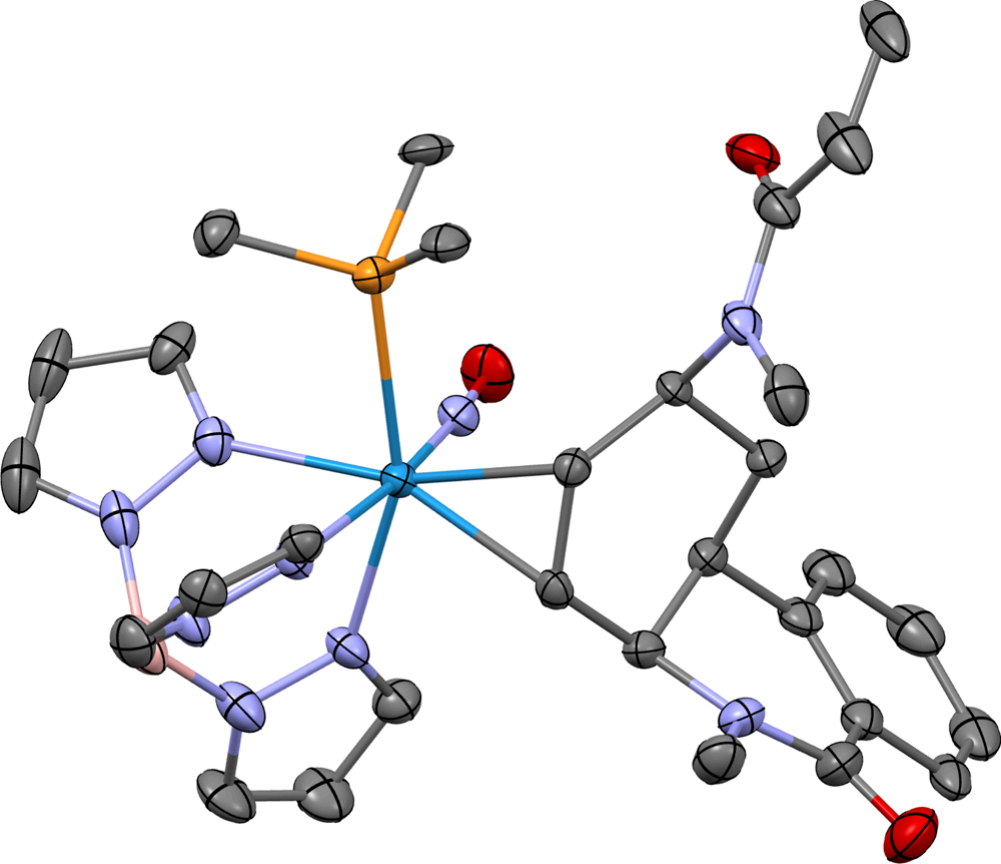
SC-XRD molecular structure of compound **74** represented with 50% ellipsoids.

Compounds **55**–**66** in Figure 7 and **70** and **75** in Figure 8 feature three stereogenic carbons originating from a benzene, whose configurations were determined directly by the metal complex. Although not explored in this study, the ability to enantioenrich WTp­(NO)­(PMe_3_)­(arene)^37^ provides a method for obtaining reliably enantioenriched organic products using this process.^22,23^

## Conclusions

This article presents a synthetic strategy for producing functionalized *cis*-tetrahydro-2-oxindoles using tungsten-based dearomatization.^38^ The method enables modular access to 1,2,5-functionalized semisaturated indolic compounds, which are important scaffolds in natural products and pharmaceuticals. The synthesis begins with coordination of a phenyl sulfone to the tungsten fragment {WTp­(NO)­(PMe_3_)}, followed by protonation, ester enolate, and amine addition to the coordinated phenyl ring, ultimately forming stable dihydro-2-oxindole complexes through γ-lactam construction and sulfinic acid elimination. Exposure to acid transforms the dihydro-2-oxindole complexes into η^2^-allyl salts, which are stable under an inert atmosphere and serve as intermediates for further functionalization. These η^2^-allyl complexes undergo selective nucleophilic addition with carbon, nitrogen, or sulfur nucleophiles to generate diverse 5-substituted tetrahydro-2-oxindole complexes. Oxidative decomplexation using NOPF_6_ or DDQ liberates the tetrahydro-2-oxindole organic ligands from the tungsten fragment in good yields with specific conditions optimized for amine and sulfur derivatives. The approach tolerates a wide range of functional groups without specialized protecting groups, avoids halides and precious metals, and efficiently constructs multiple stereocenters originating from the benzene ring. The methodology enables rapid generation of diverse, biologically relevant perhydroindole libraries, including covalent probes, demonstrating its potential utility for drug discovery.

## Supplementary Material





## Notes

Synthetic, spectroscopic, and electrochemical data (PDF)
Coordinates of calculated structures (XYZ)
