# Supplementary material for: Transition-Metal-Based Dearomatization Strategy for the Synthesis of Functionalized cis-Tetrahydro-2-Oxindoles
Source: Organometallics. 2025 Oct 28;44(21):2570–8. doi: 10.1021/acs.organomet.5c00371 (PMC12606712; doi:10.1021/acs.organomet.5c00371)
Supplement: Supplementary file 1 [file om5c00371_si_001.pdf]

A Transition-Metal Based Dearomatization Strategy for the Synthesis of Functionalized *cis*-Tetrahydro-2-Oxindoles

Paolo Siano[1‡], Louis A. Diment[1‡], Daniel J. Siela[1], Megan N. Ericson[1], Matt McGraw[1], Brian C. Song[1], Benjamin F. Livaudais[1], Spenser R. Simpson[1], Diane A. Dickie[1], and W. Dean Harman [1*]

Affiliation: [1]Department of Chemistry, University of Virginia; Charlottesville, VA 22904 U.S.A.

‡ Authors equally contributed

*Corresponding author. Email: wdh5z@virginia.edu

Table of Contents

**NMR Spectra:**	S2
**Supplementary Methods:**	S124
**DFT Analysis:**	S168
**Crystallographic Data:**	S169
**Supplementary References:**	S174

Compounds 1, 2, 3a, 3b, 3c, 10, 11 and 72 have been previously reported, along with SC-XRD data for 1, 3a, 3b, 3c, 10, 11 and 72[1,2].

NMR Data:

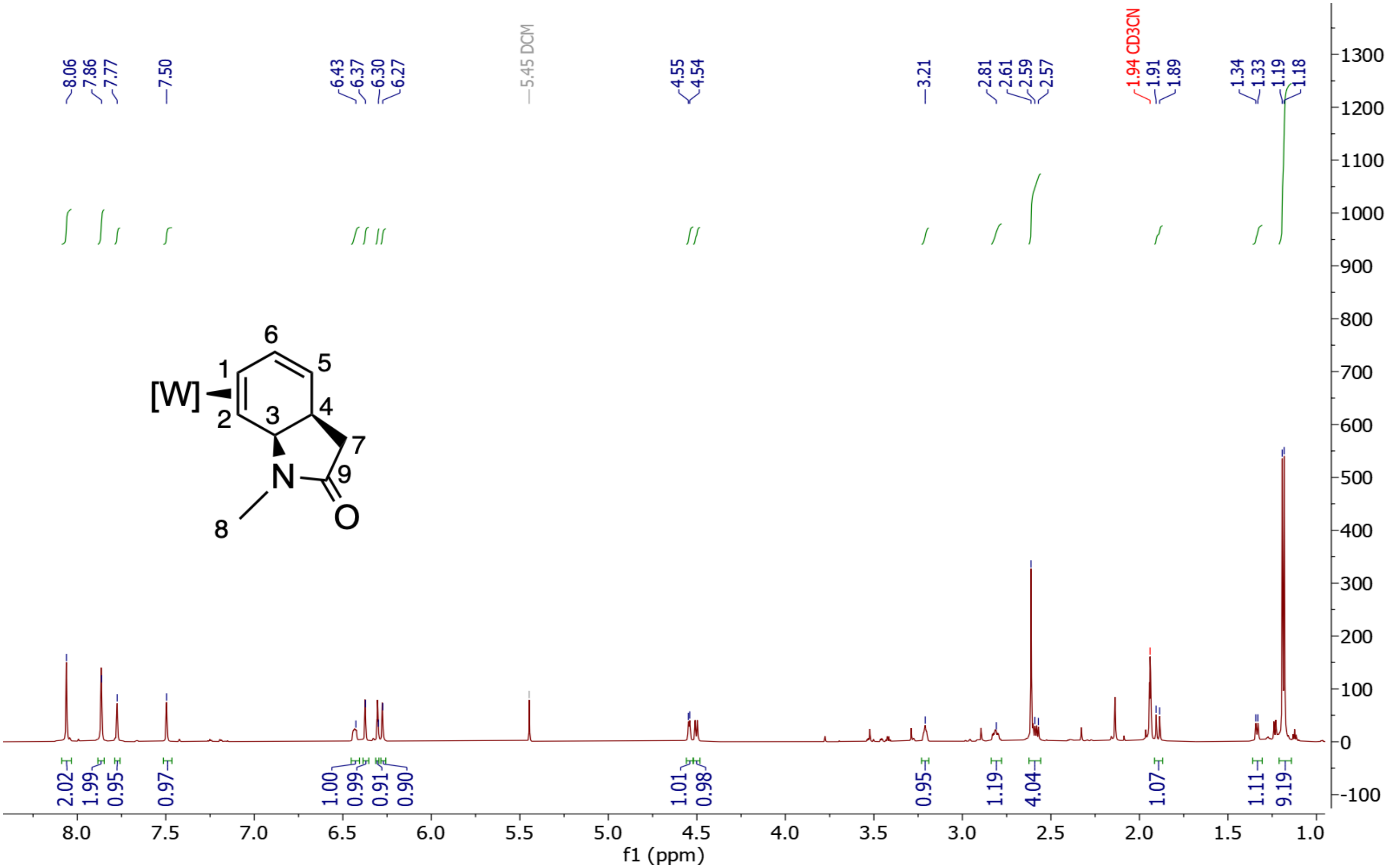

Figure S1: ^{1}H-NMR (CD_3CN) of Compound 9.

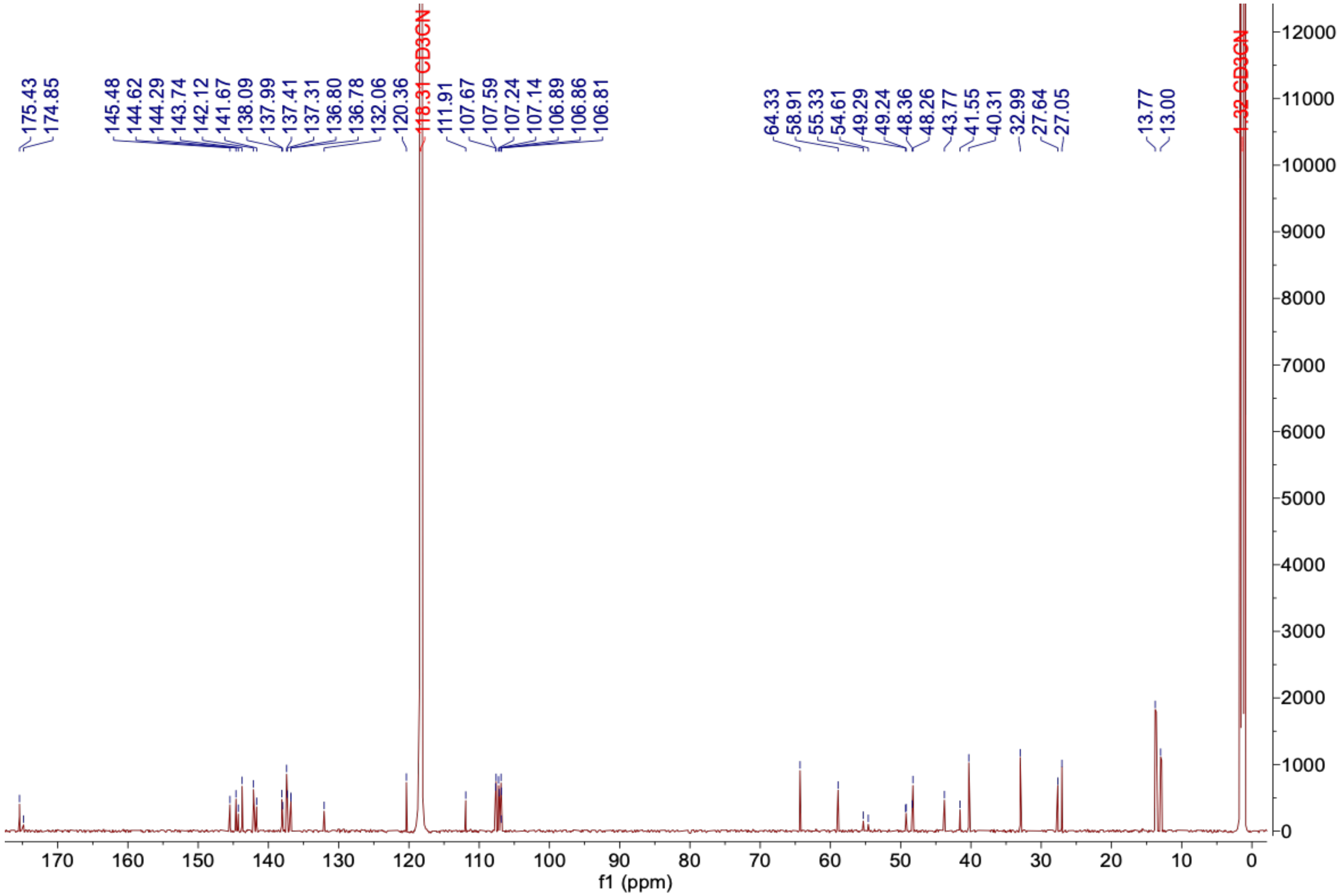

Figure S2: ^{13}C-NMR (CD_3CN) of Compound 9.
Compound ring-walks during the 2D NMR experiment.

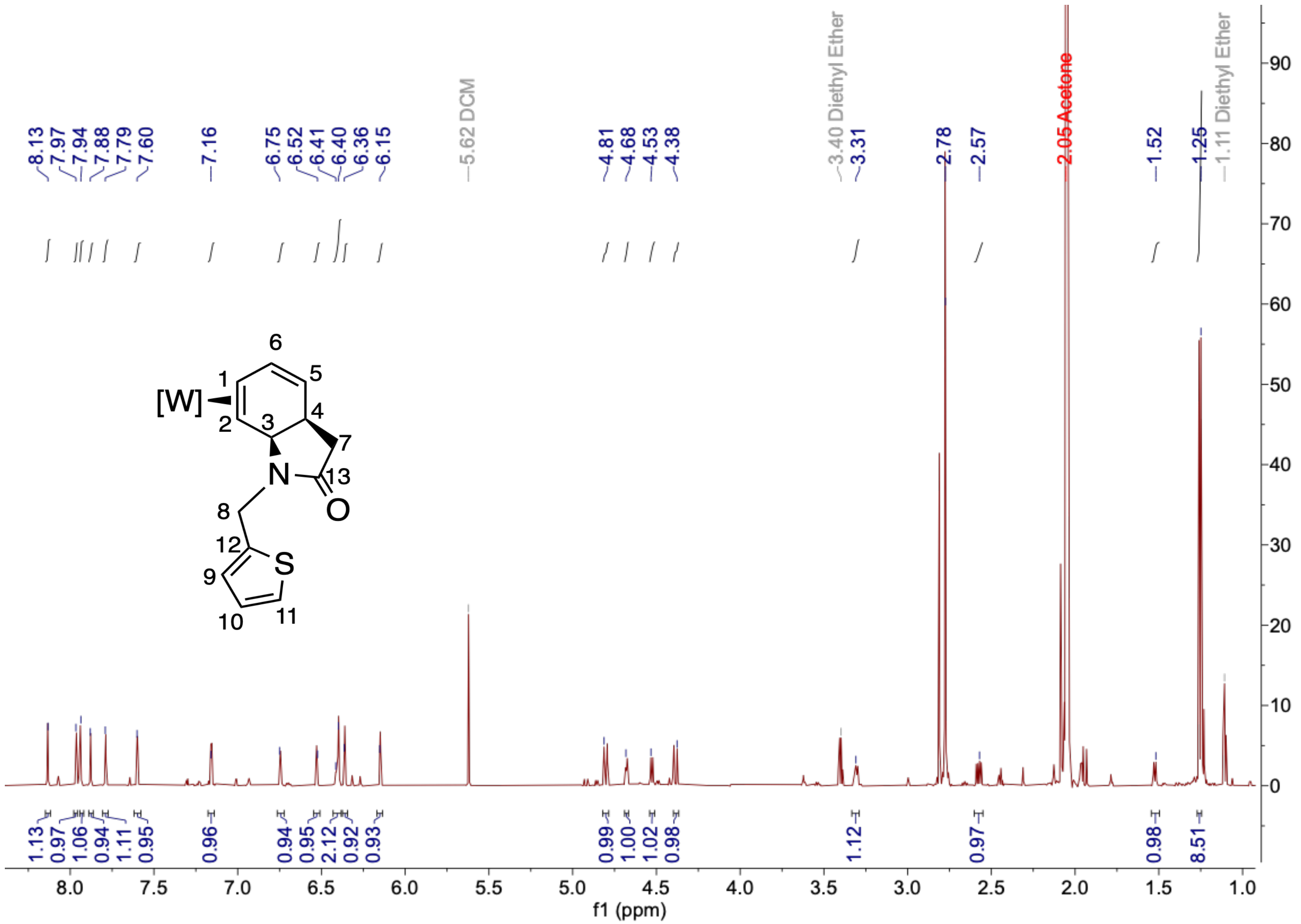

Figure S3: ^{1}H-NMR (($(CD_3)_2CO$)) of Compound 12.
Compound ring-walks during the 2D NMR experiment.

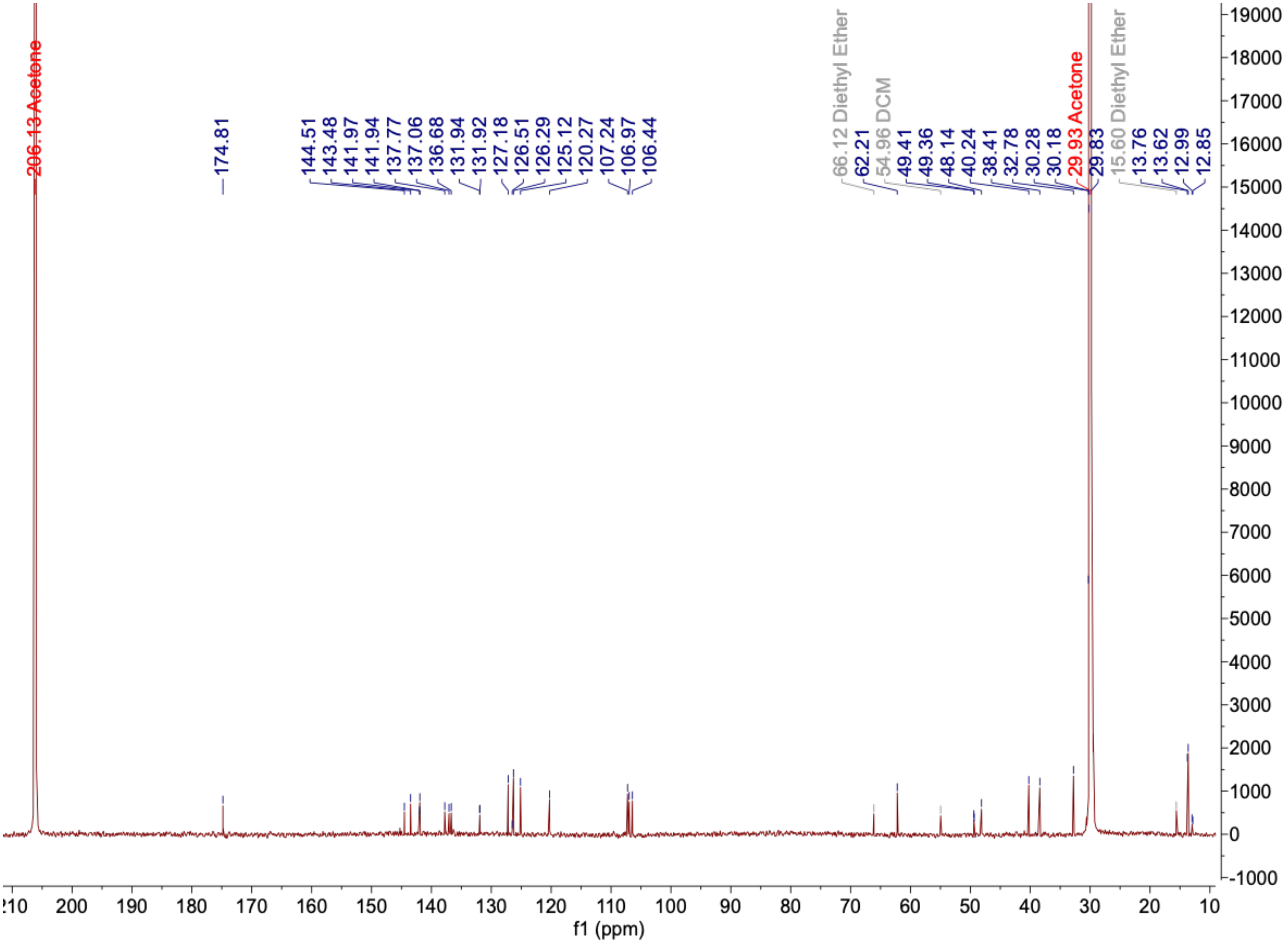

Figure S4: ^{13}C-NMR ($(CD_3)_2CO$) of Compound 12.

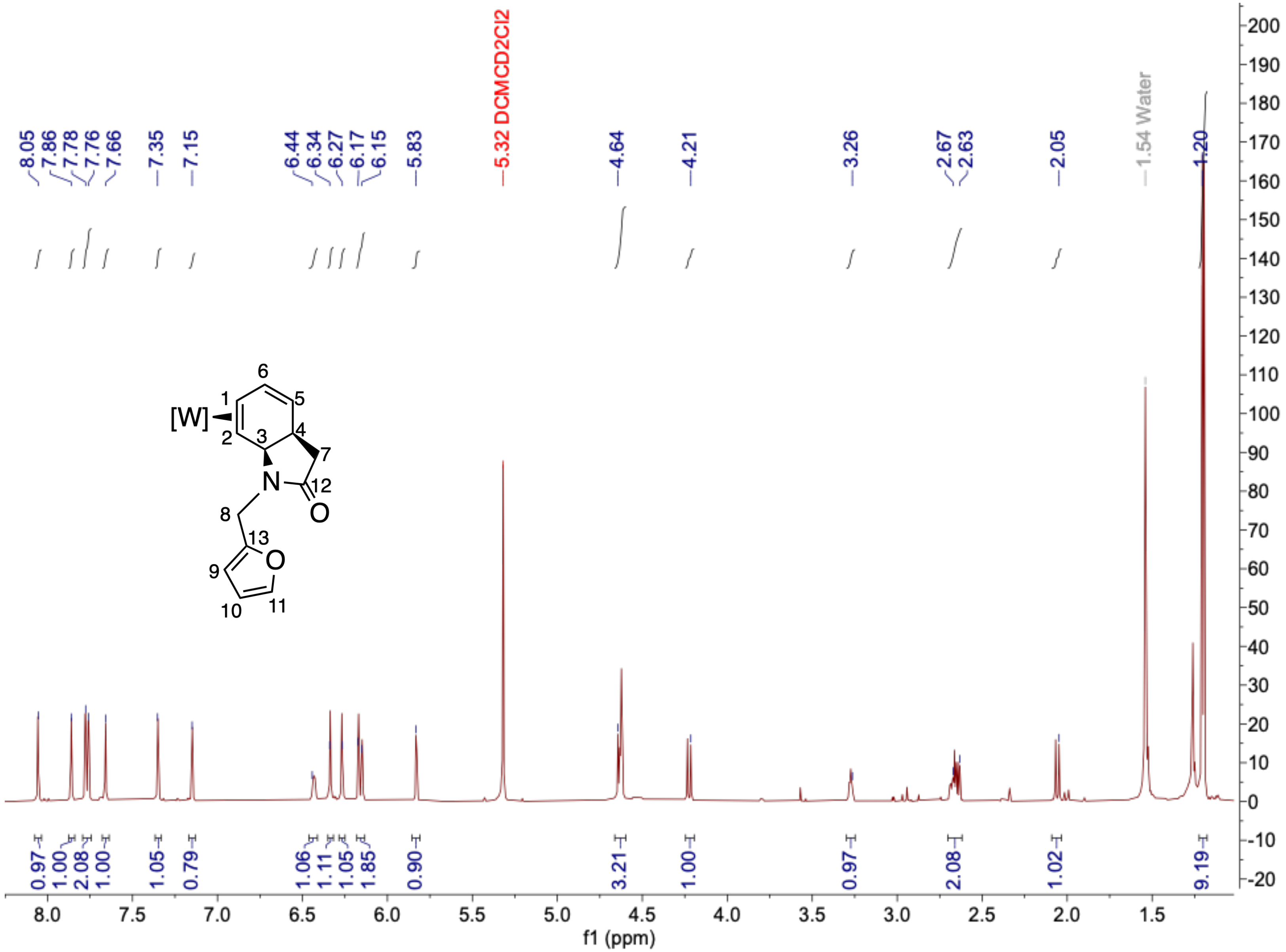

Figure S5: ^{1}H-NMR (CD_2Cl_2) of Compound 13.

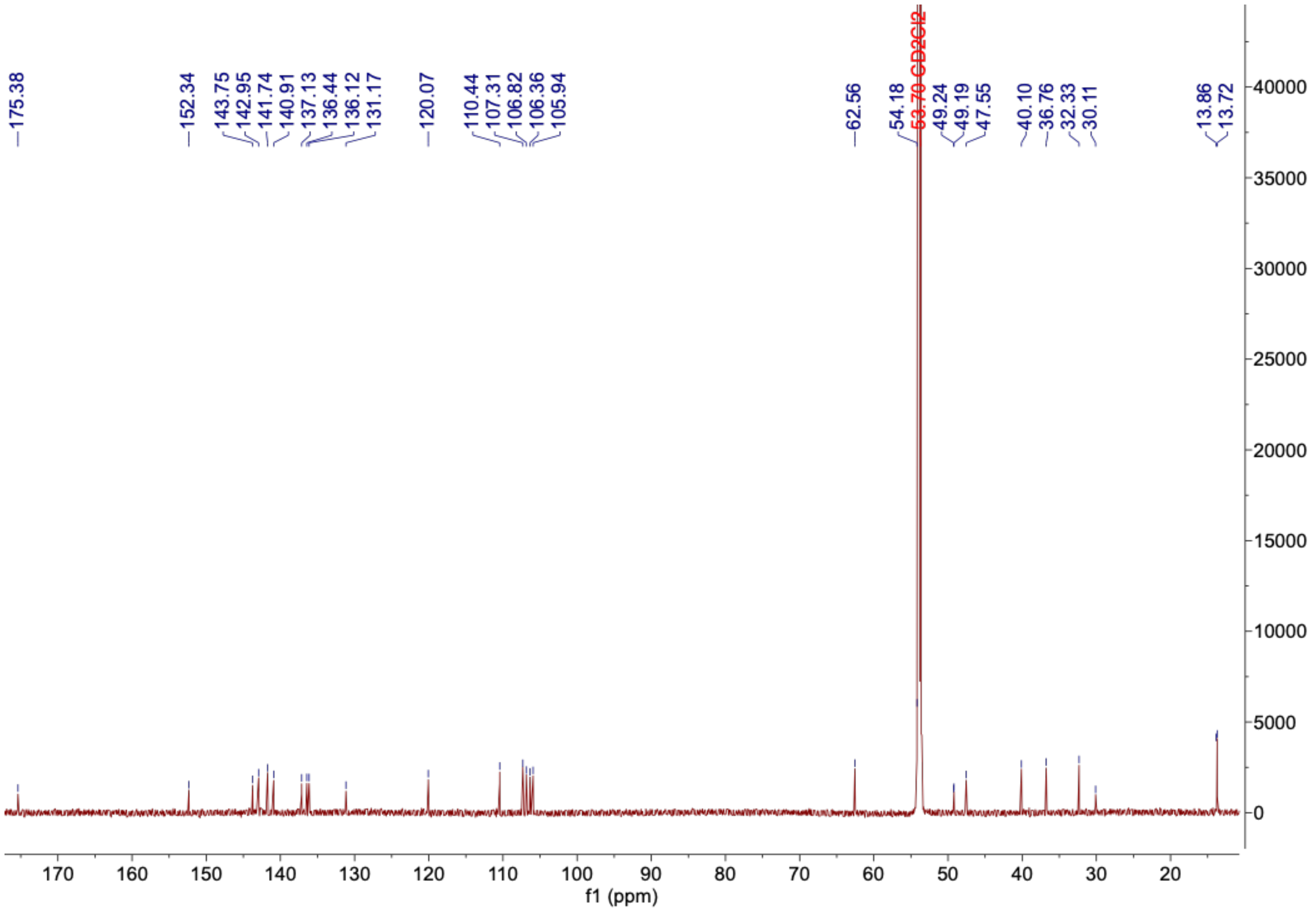

Figure S6: ^{13}C-NMR (CD_2Cl_2) of Compound 13.

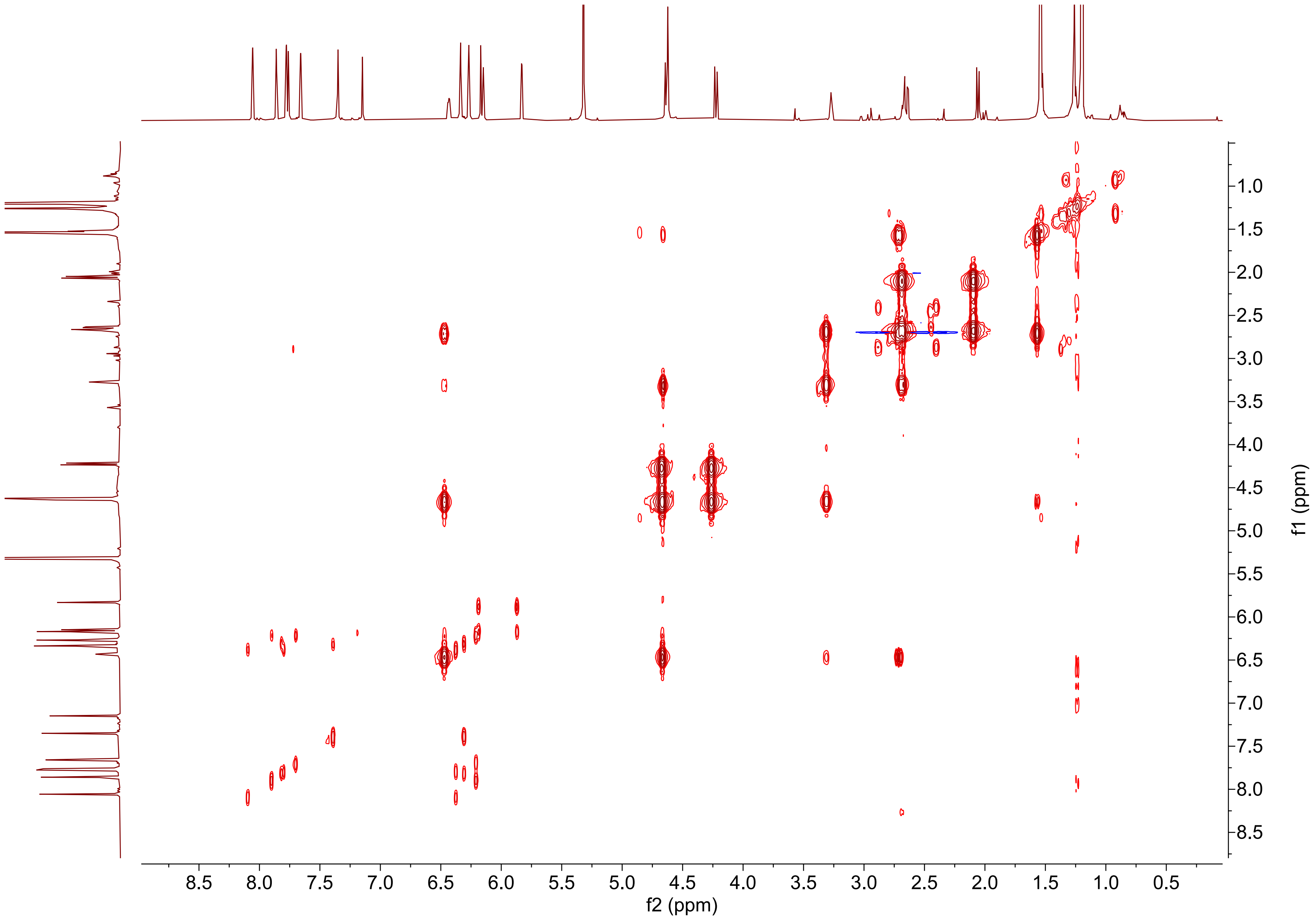

Figure S7: ^{1}H-^{1}H COSY (CD_2Cl_2) of Compound 13.

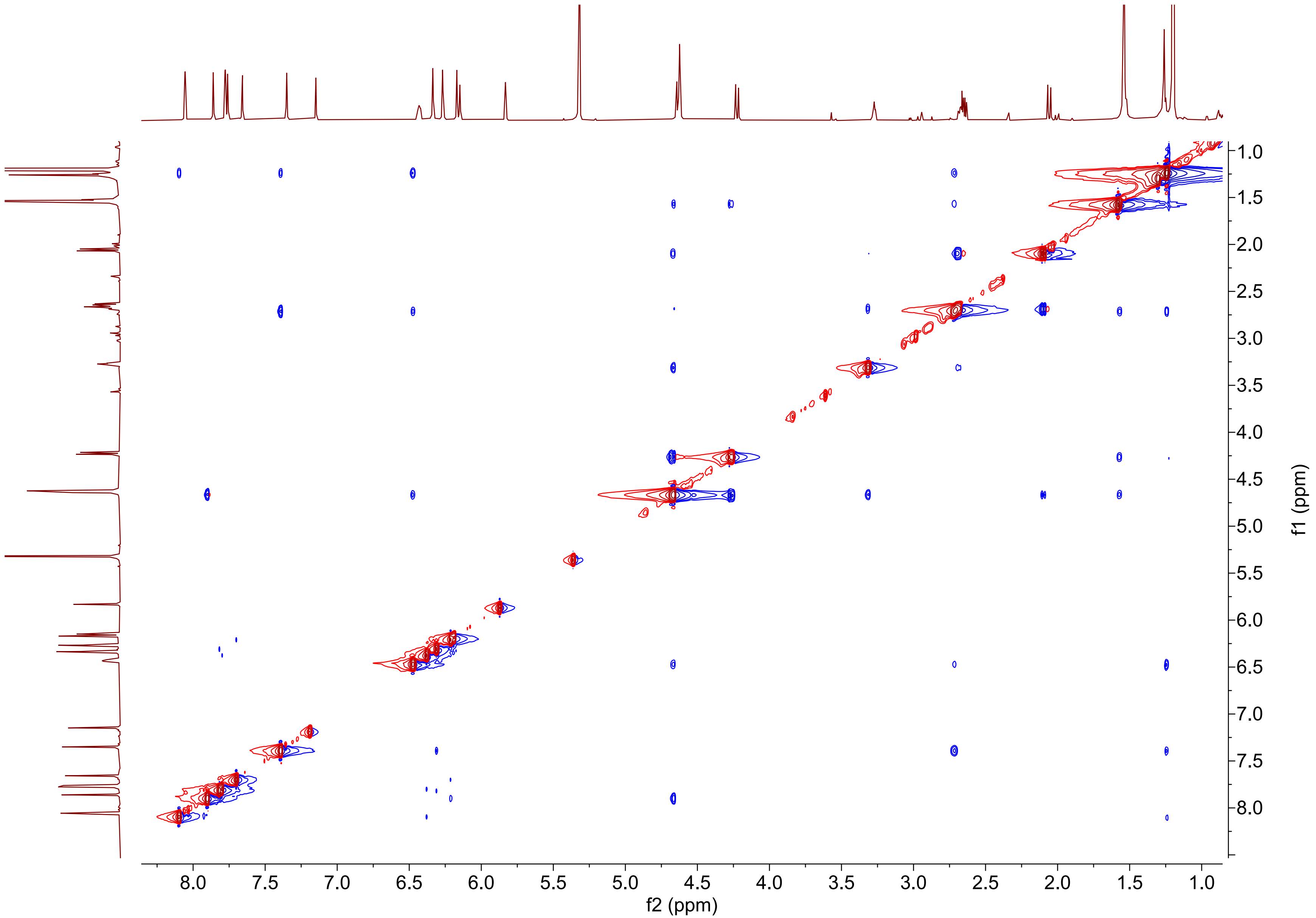

Figure S8: ^{1}H-^{1}H NOESY (CD_2Cl_2) of Compound 13.

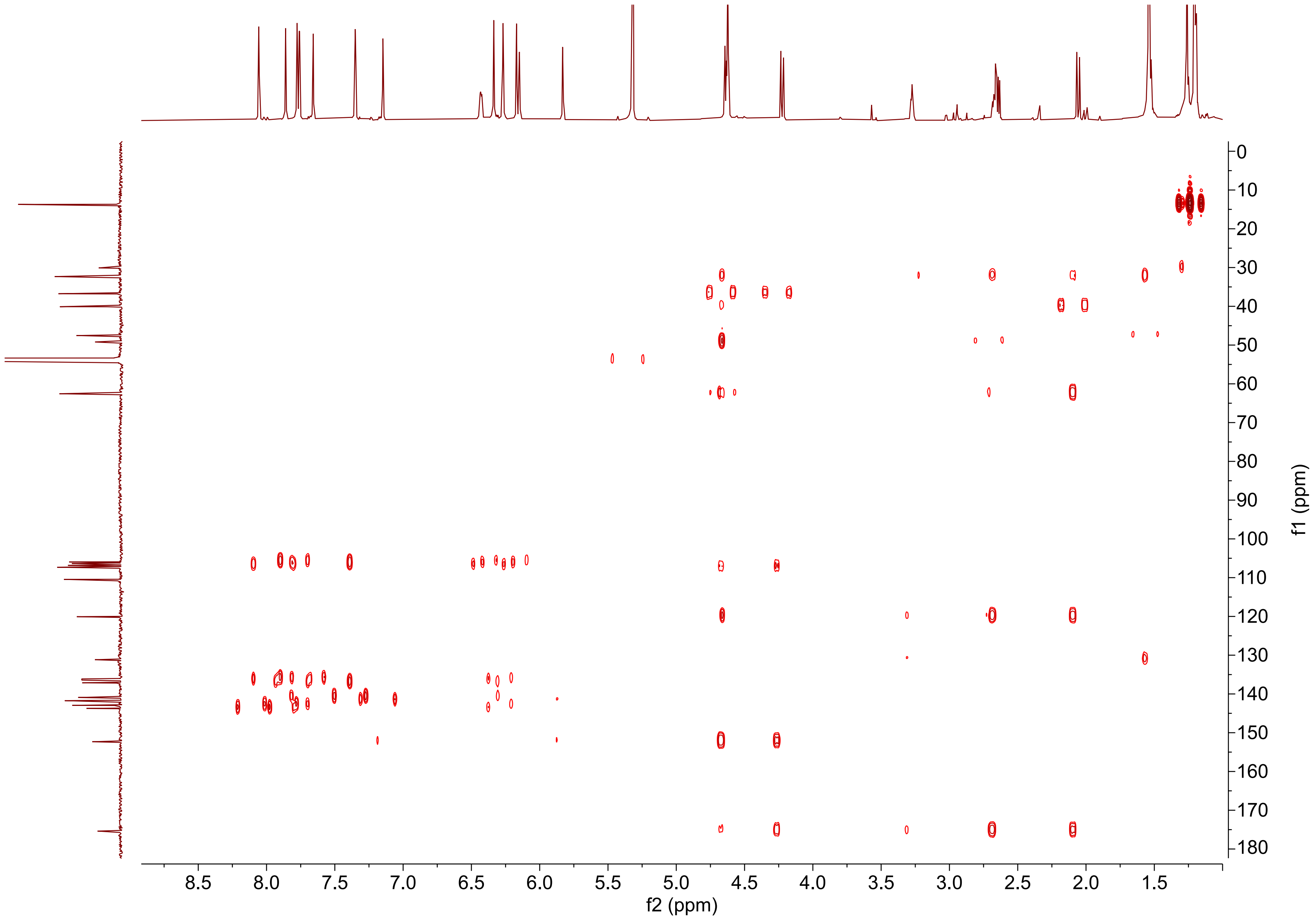

Figure S9: ^{1}H-^{13}C HMBC (CD_2Cl_2) of Compound 13.

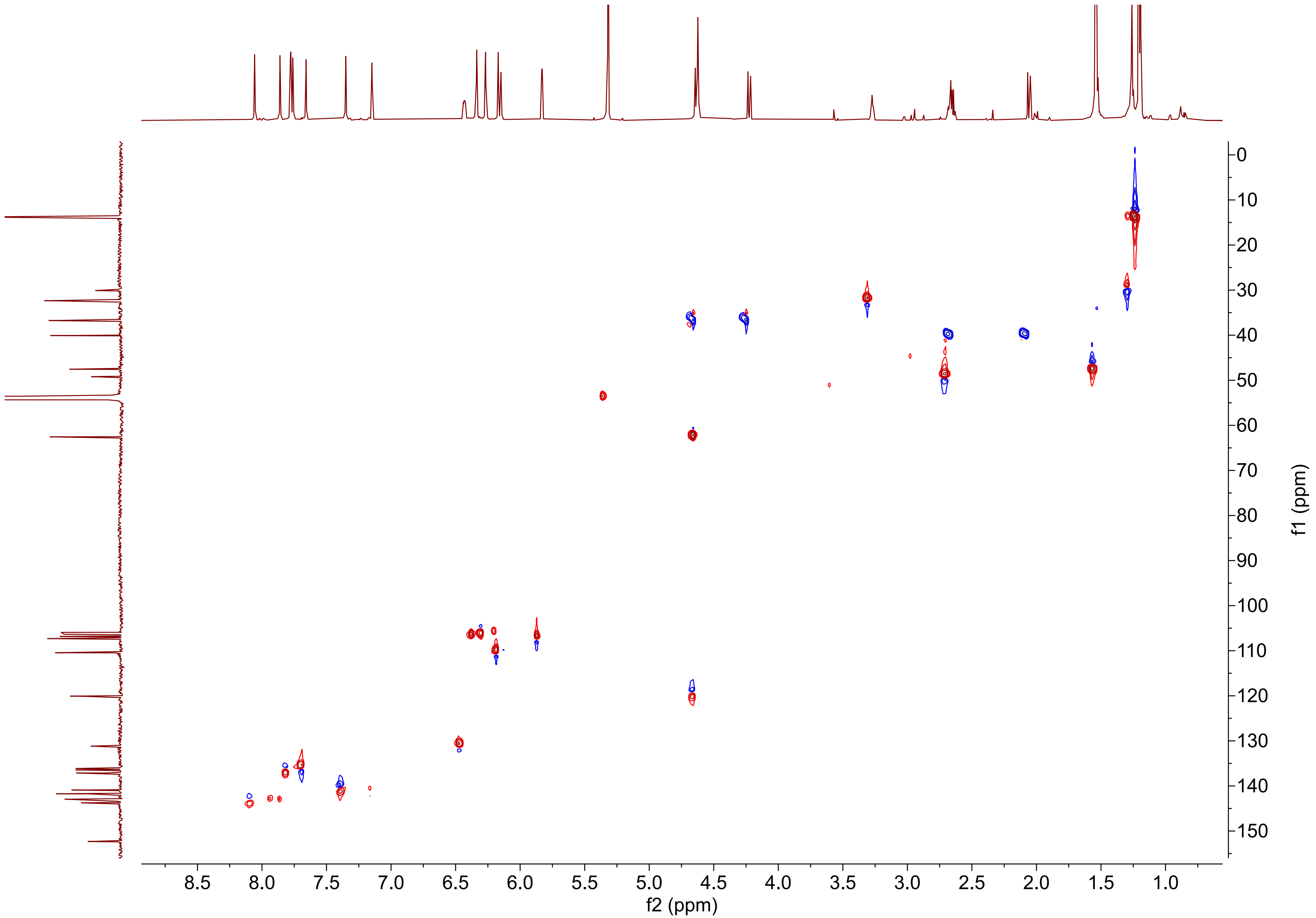

Figure S10: ^{1}H-^{13}C HSQC (CD_2Cl_2) of Compound 13.

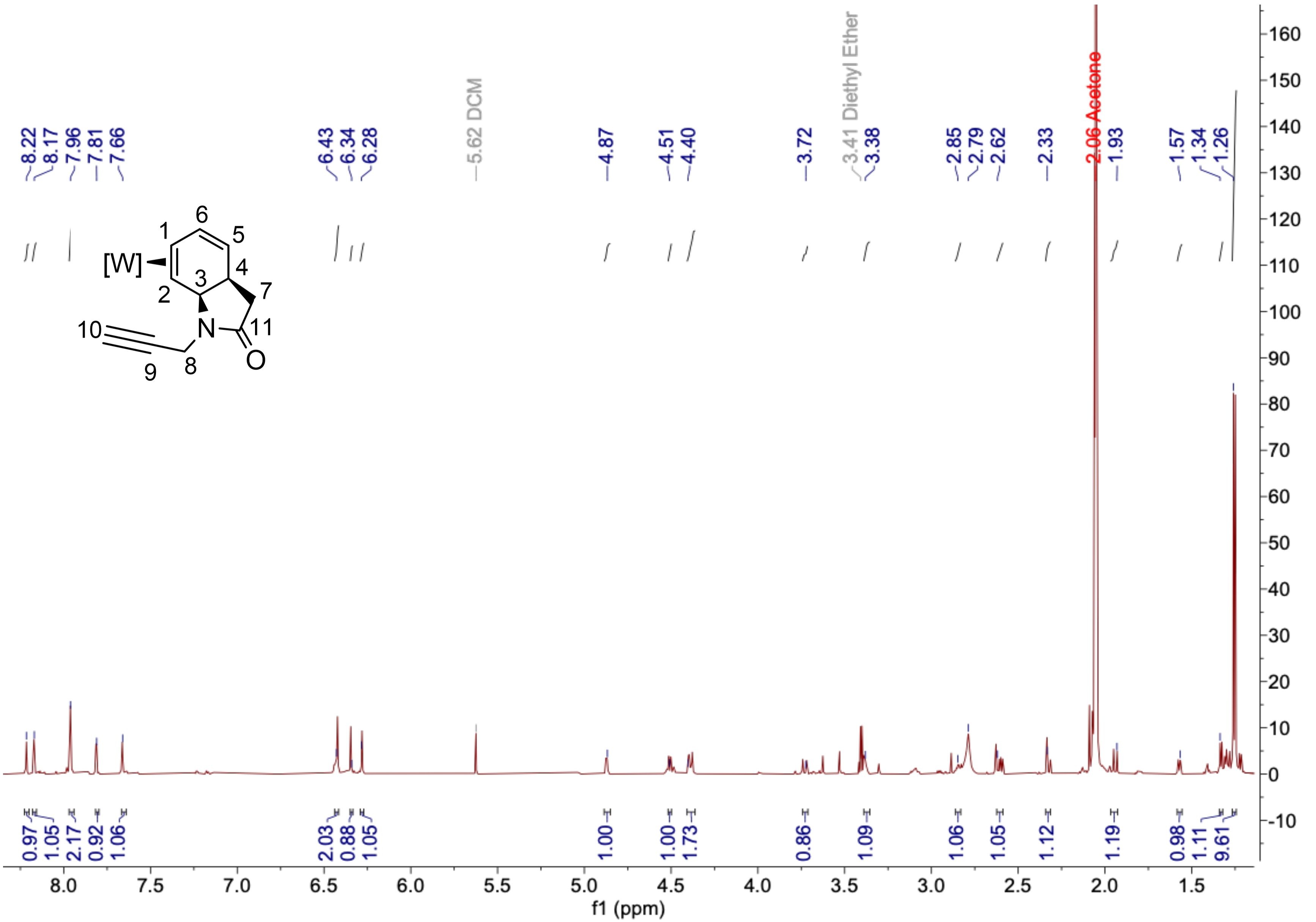

Figure S11: ^{1}H-NMR (($(CD_3)_2CO$)) of Compound 14.

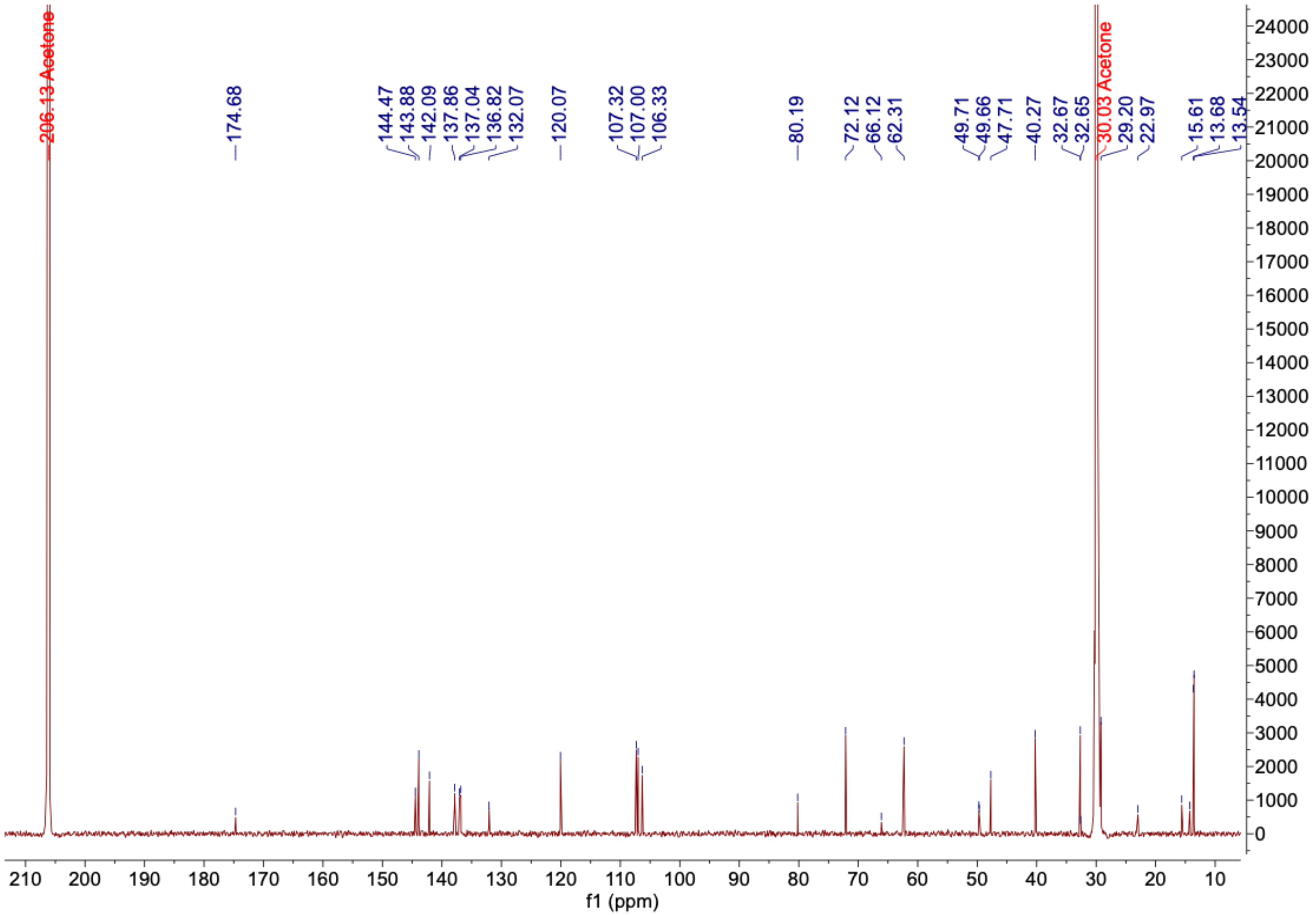

Figure S12: ^{13}C-NMR ($(CD_3)_2CO$) of Compound 14.

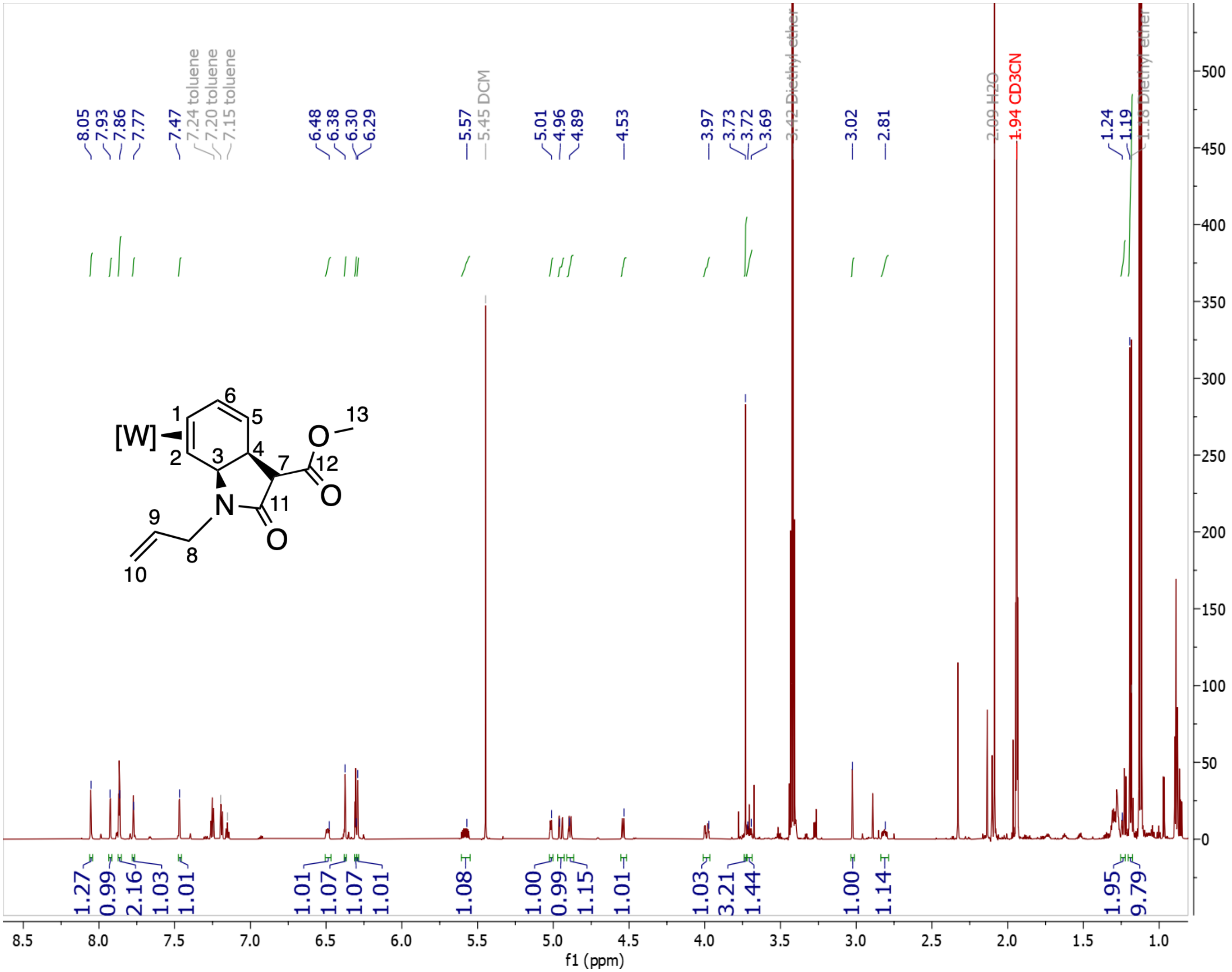

Figure S13: ^{1}H-NMR (CD_3CN) of Compound 15.

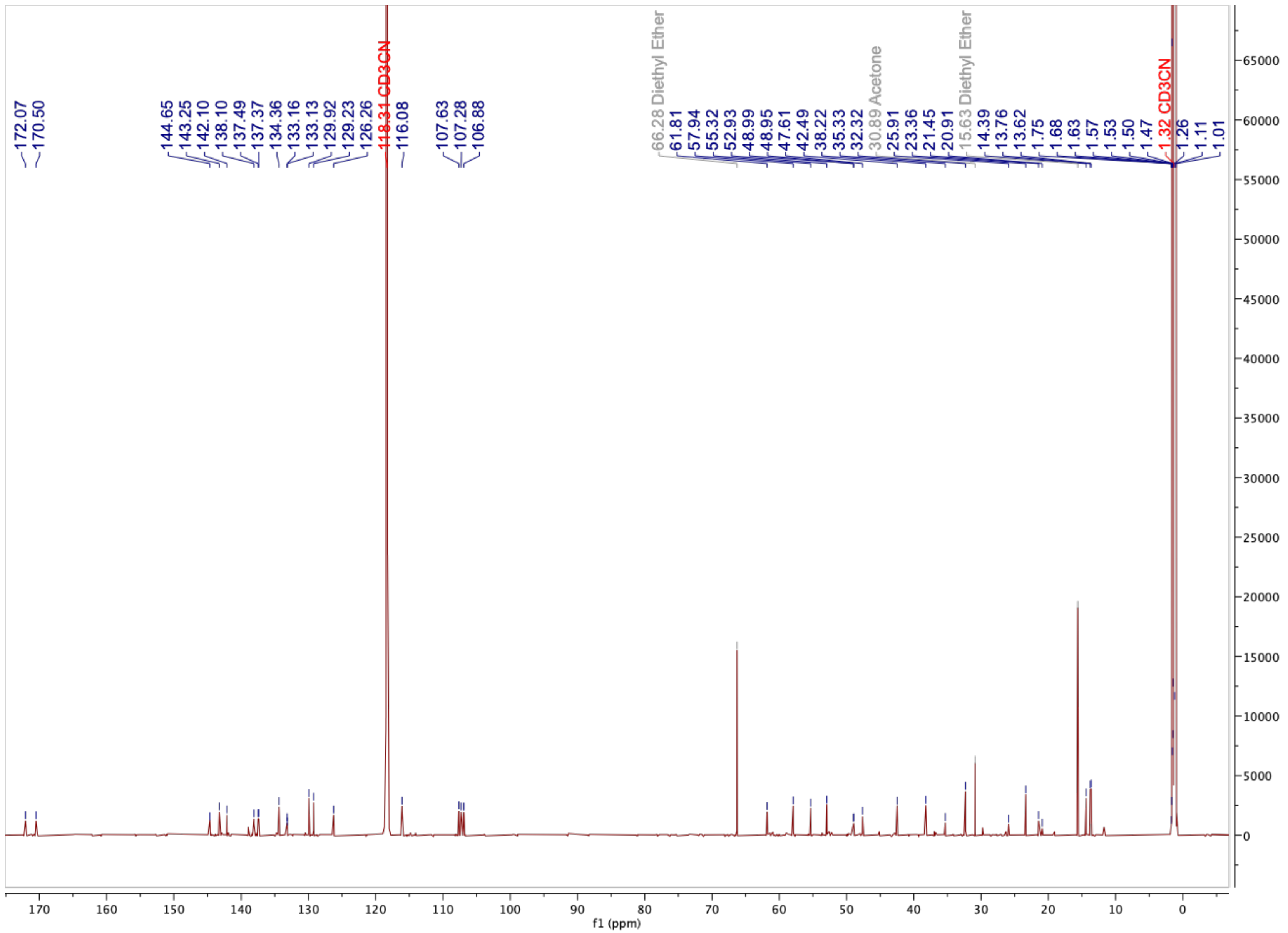

Figure S14: ^{13}C-NMR (CD_3CN) of Compound 15.

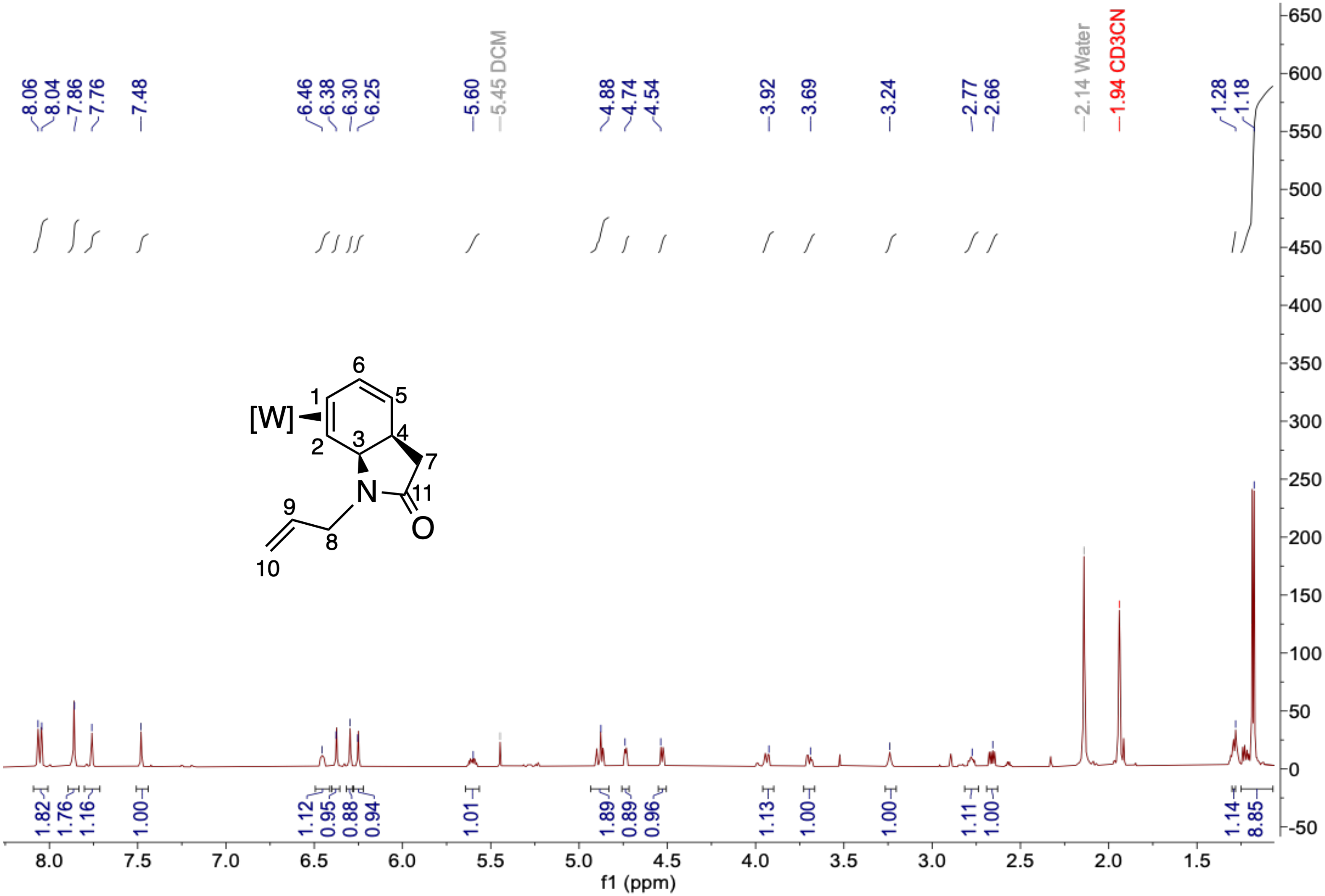

Figure S15: ^{1}H-NMR (CD_3CN) of Compound 16.

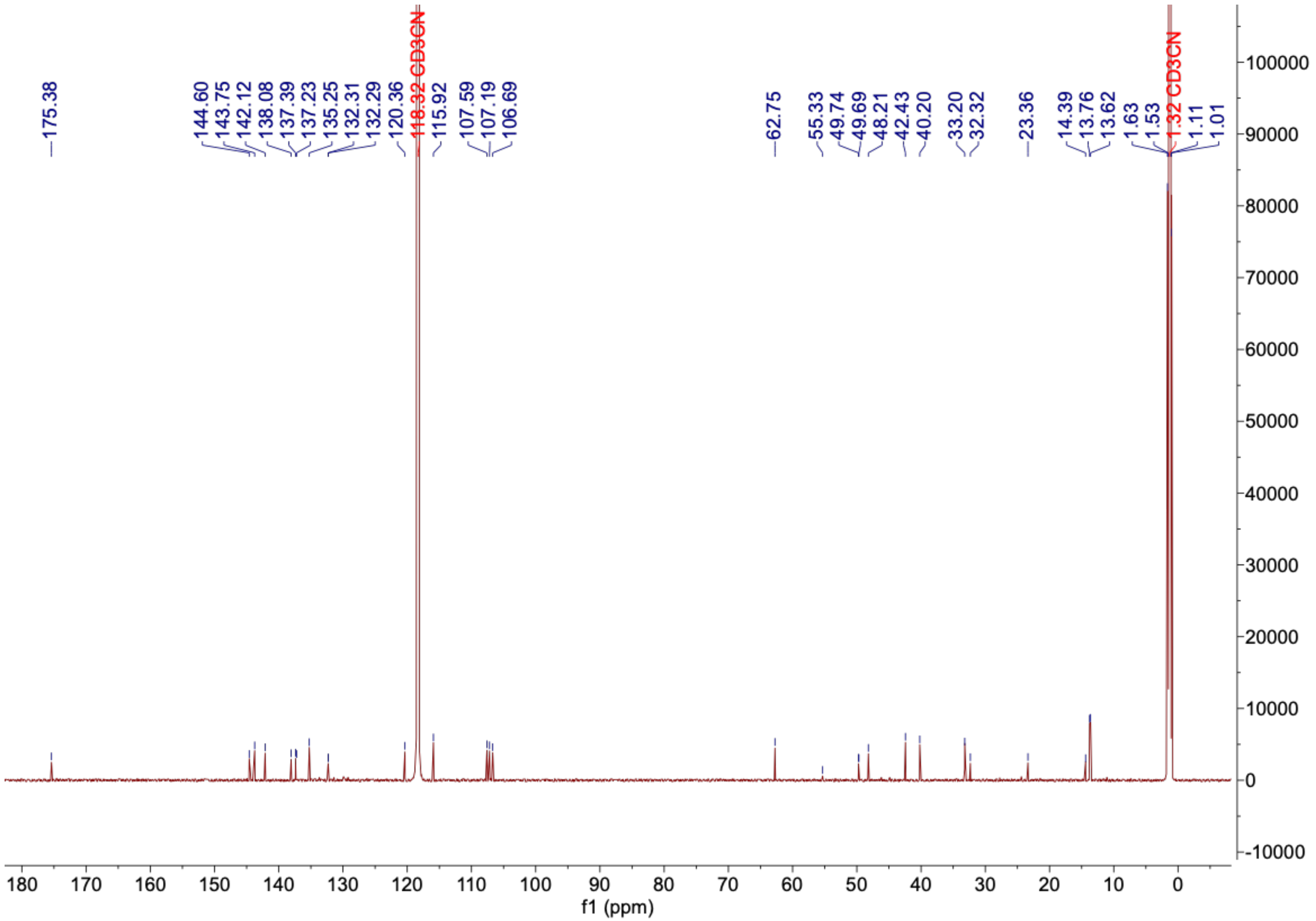

Figure S16: ^{13}C-NMR (CD_3CN) of Compound 16.

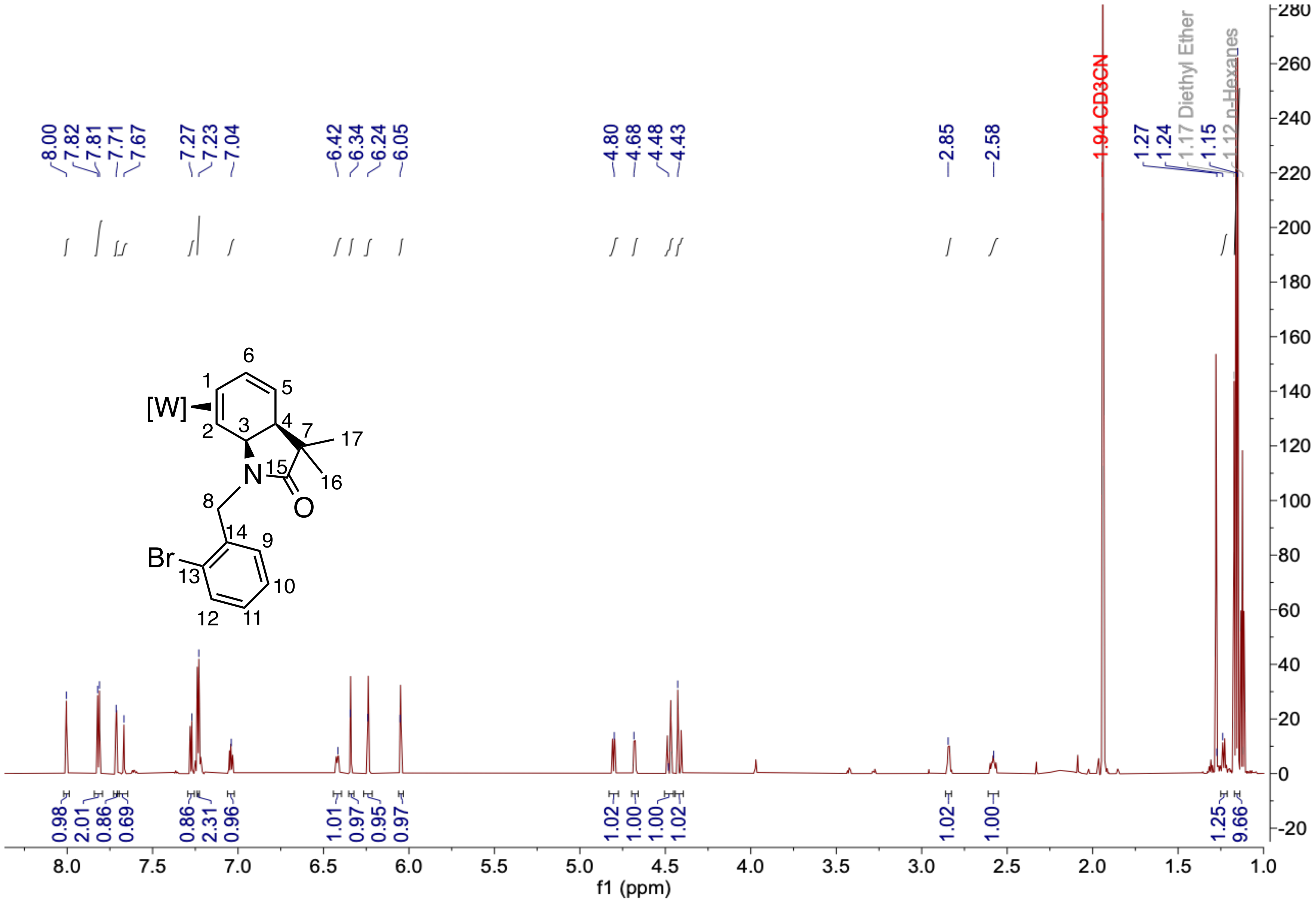

Figure S17: ^{1}H-NMR (CD_3CN) of Compound 17.

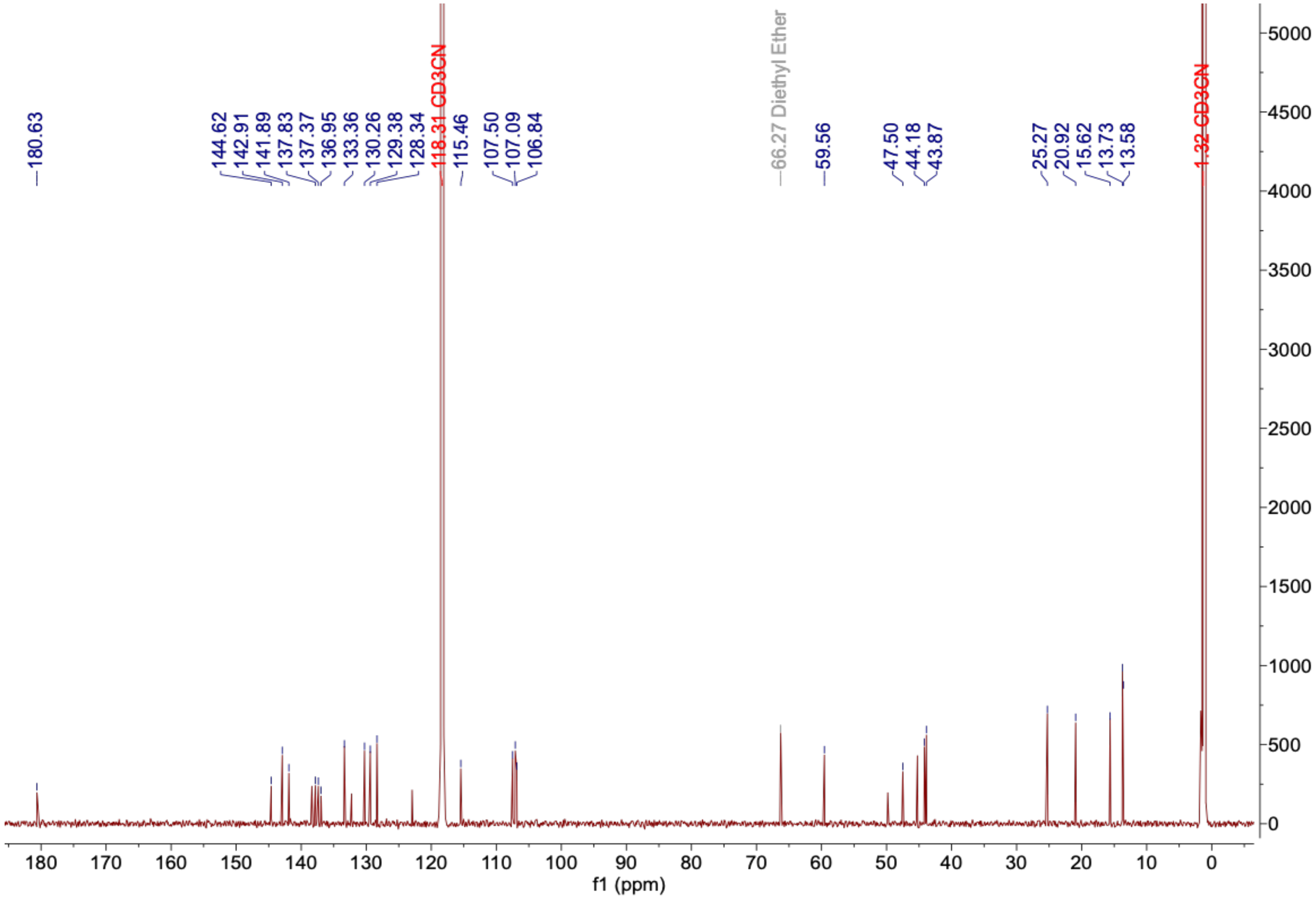

Figure S18: ^{13}C-NMR (CD_3CN) of Compound 17.

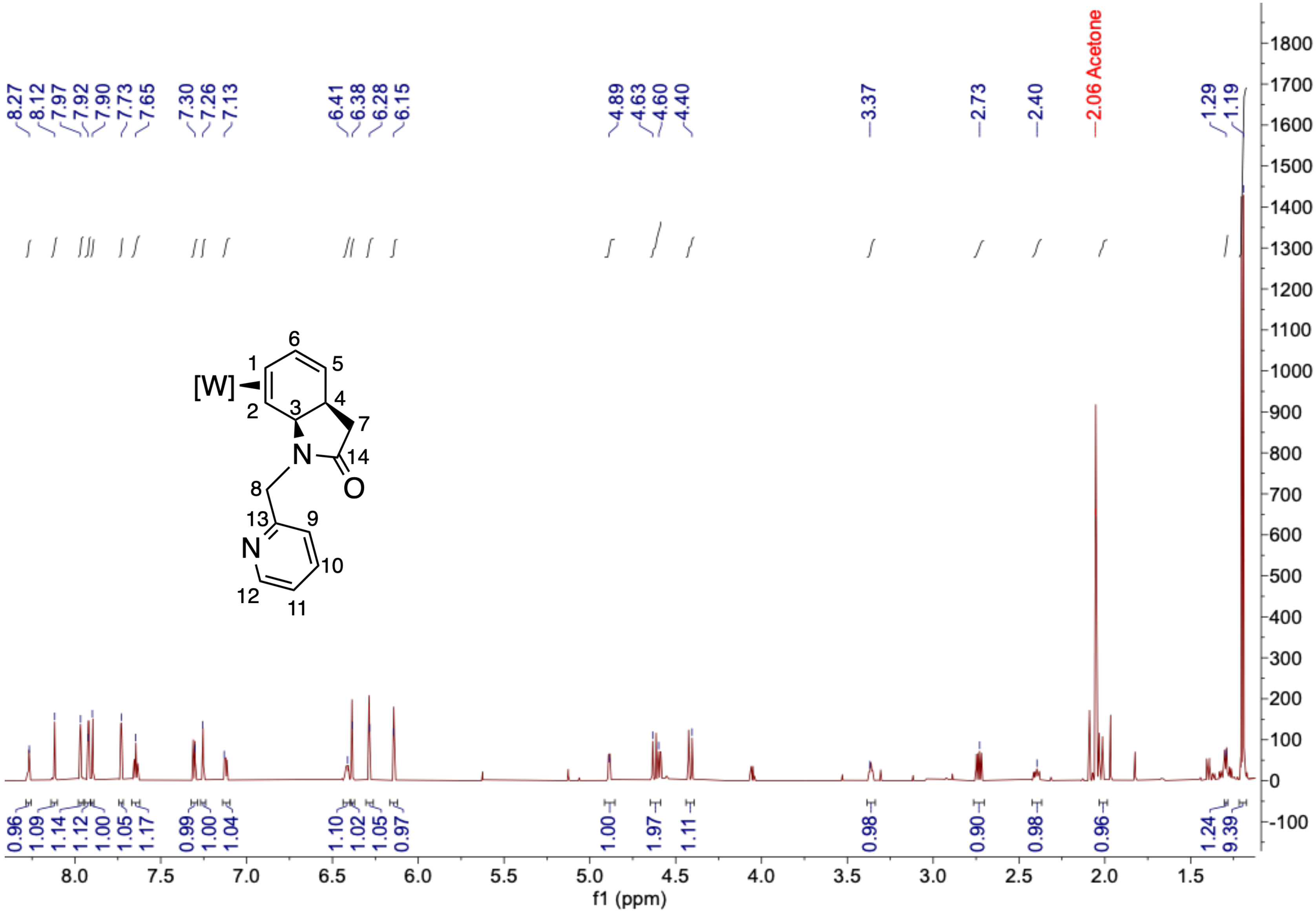

Figure S19: ^{1}H-NMR ($(CD_3)_2CO$) of Compound 18.

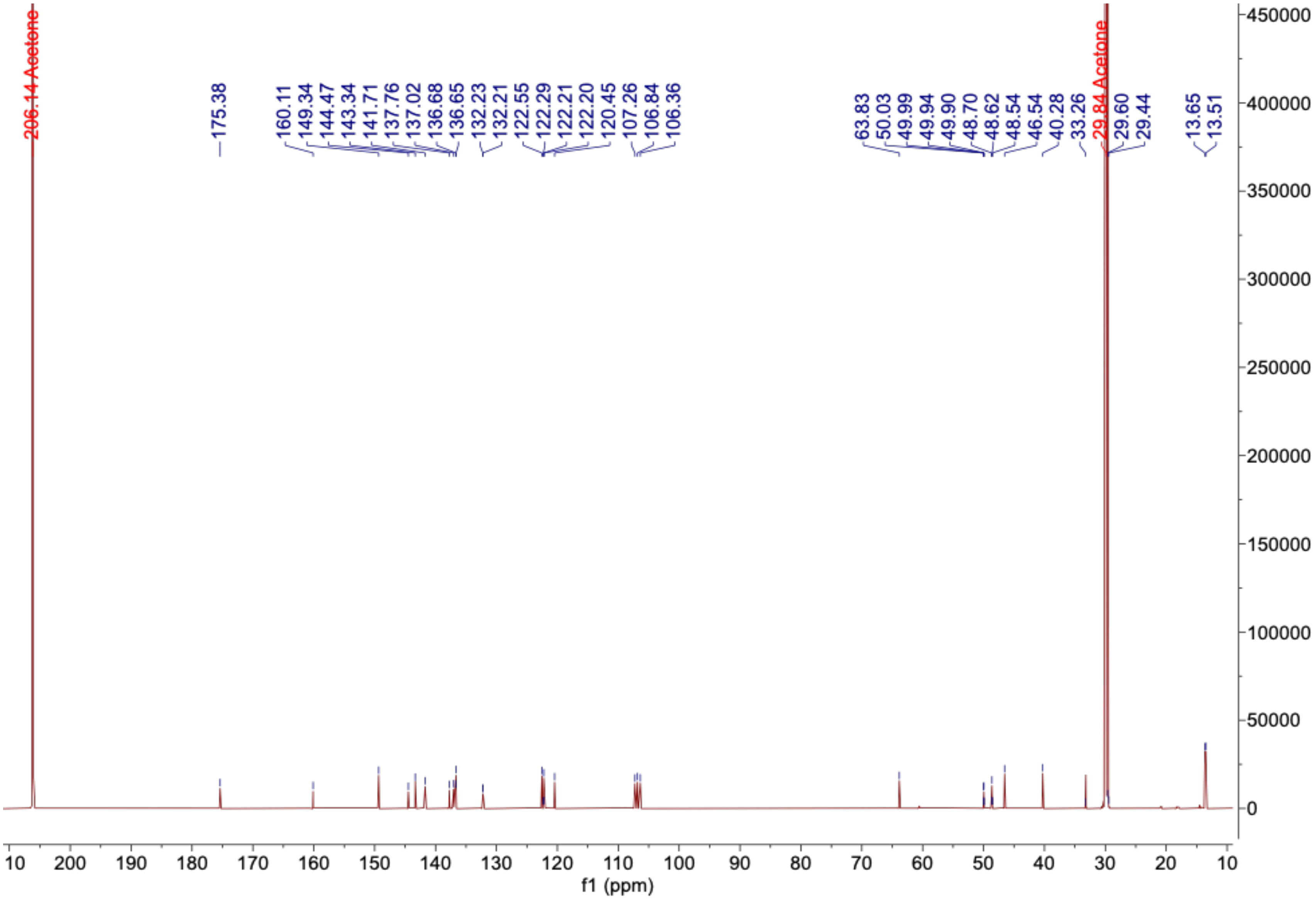

Figure S20: ^{13}C-NMR ($(CD_3)_2CO$) of Compound 18.

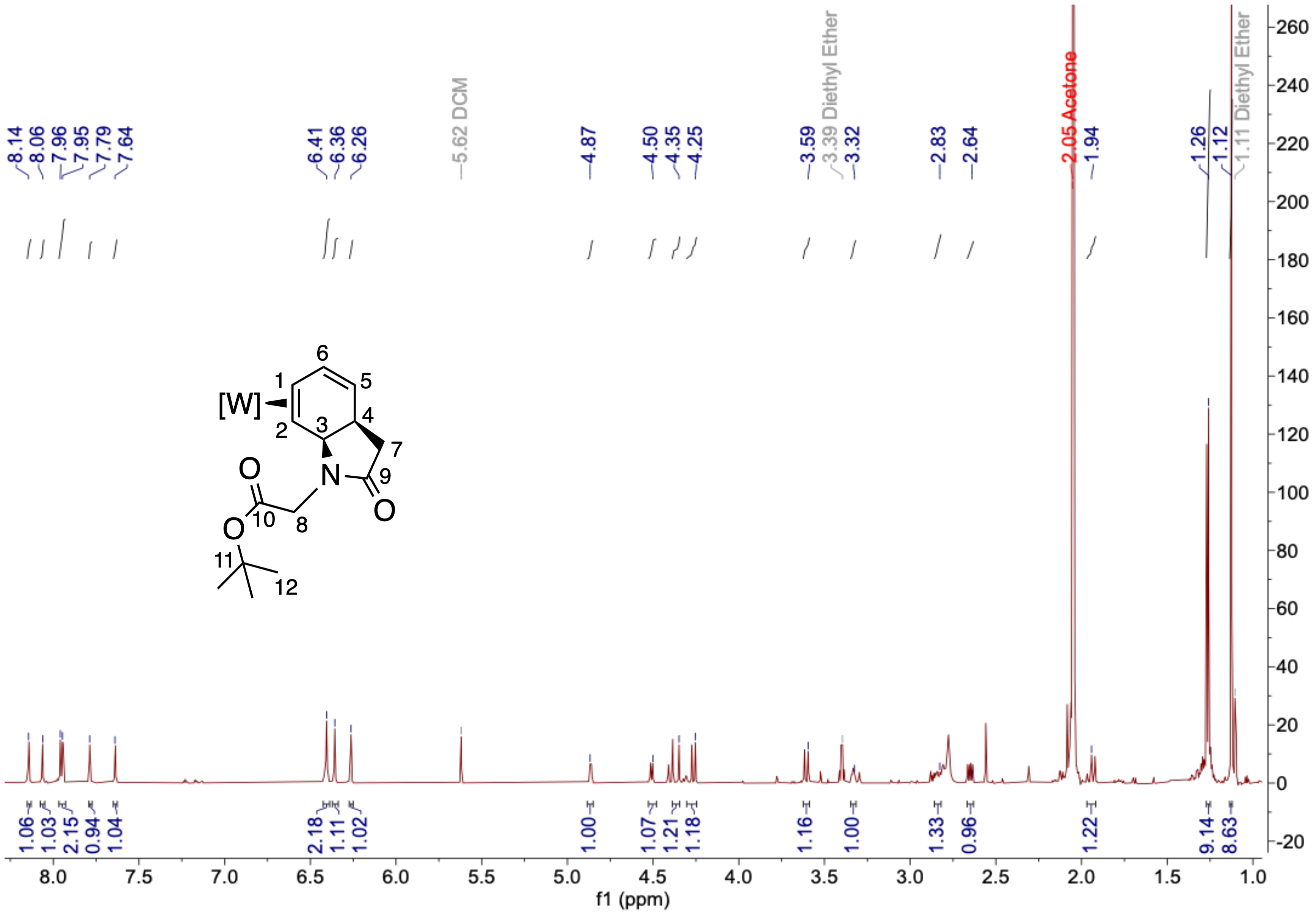

Figure S21: ^{1}H-NMR ($(CD_3)_2CO$) of Compound 19.

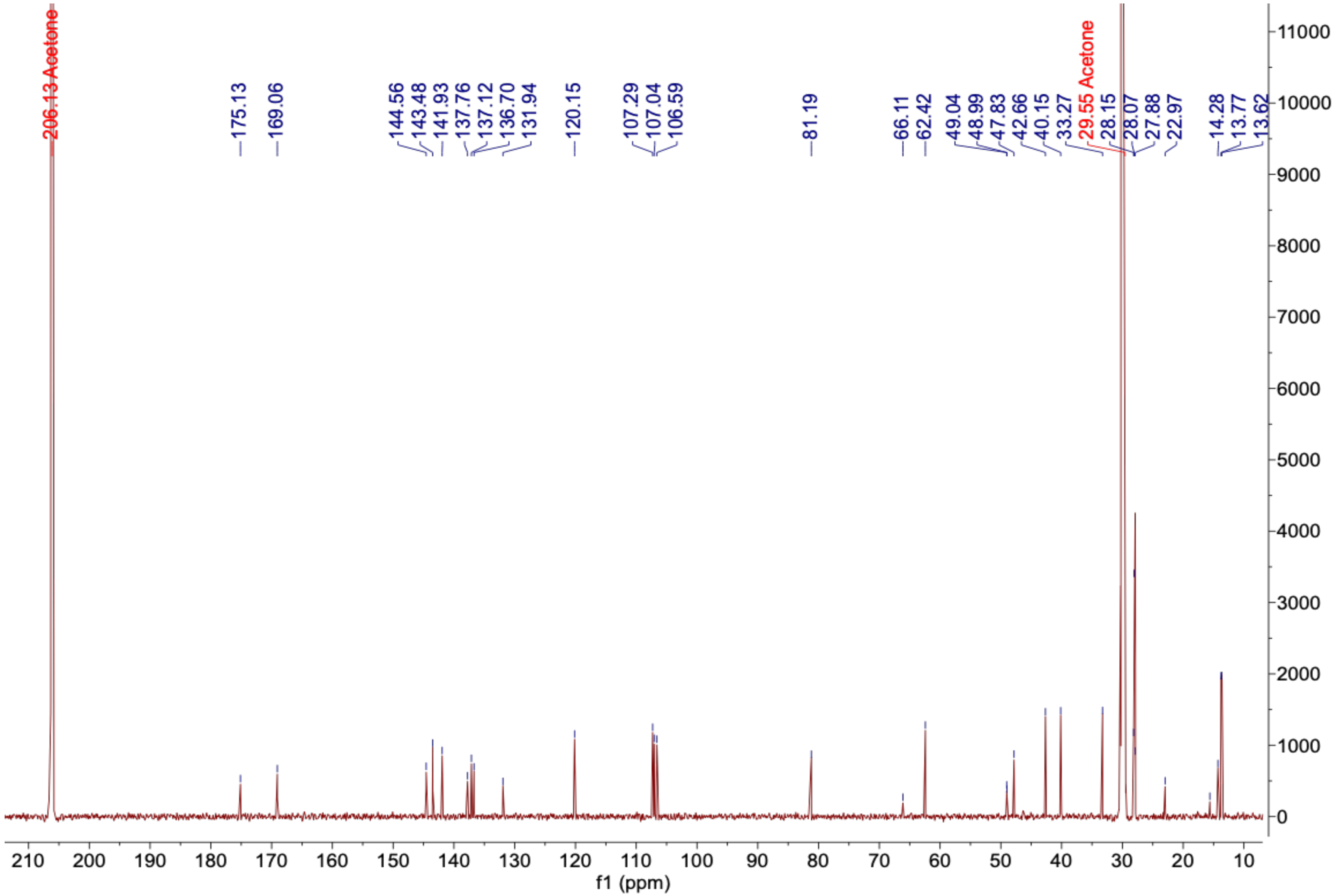

Figure S22: ^{13}C-NMR ($(CD_3)_2CO$) of Compound 19.

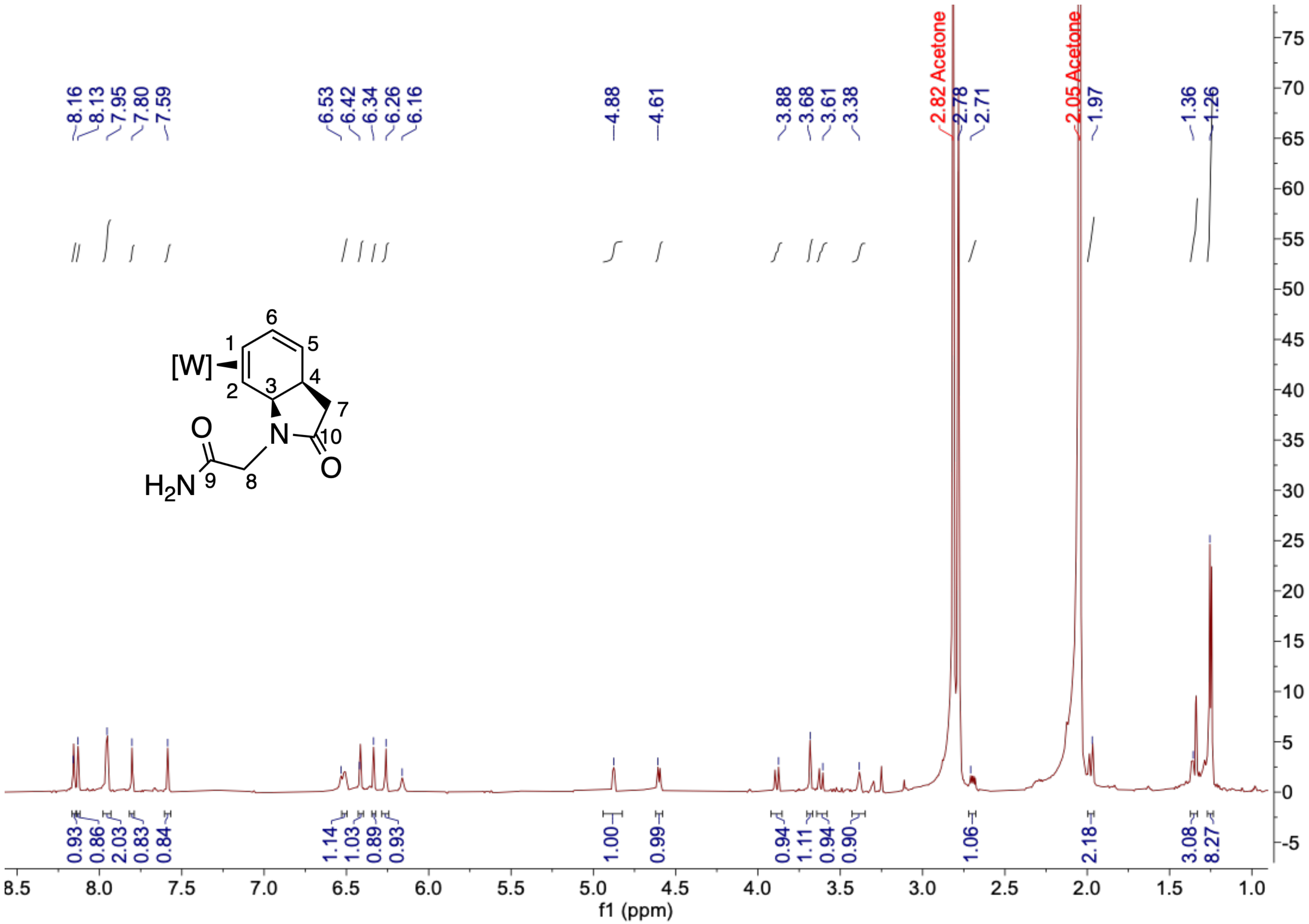

Figure S23: ^{1}H-NMR ($(CD_3)_2CO$) of Compound 20.

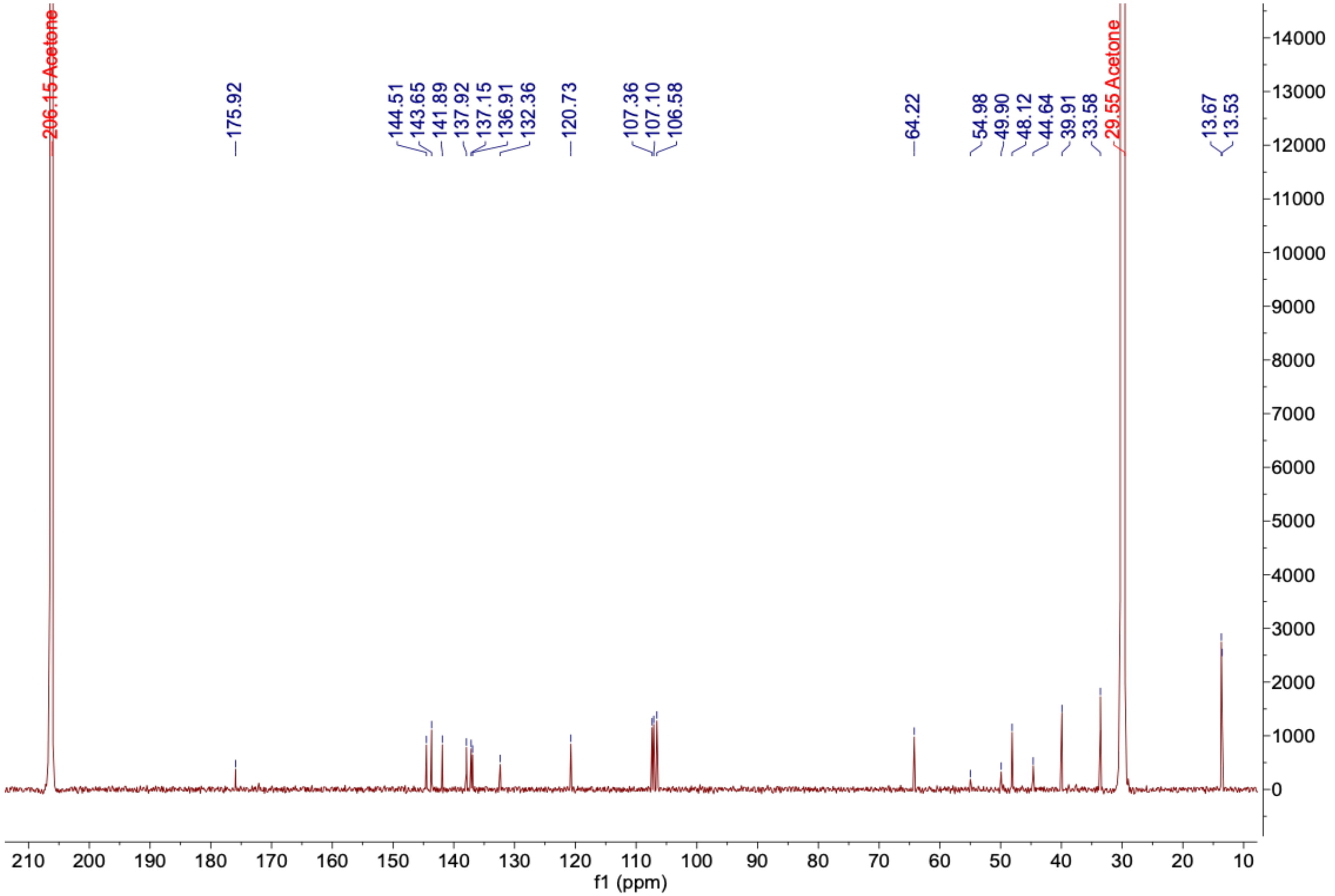

Figure S24: ^{13}C-NMR (($CD_3)_2CO$) of Compound 20.

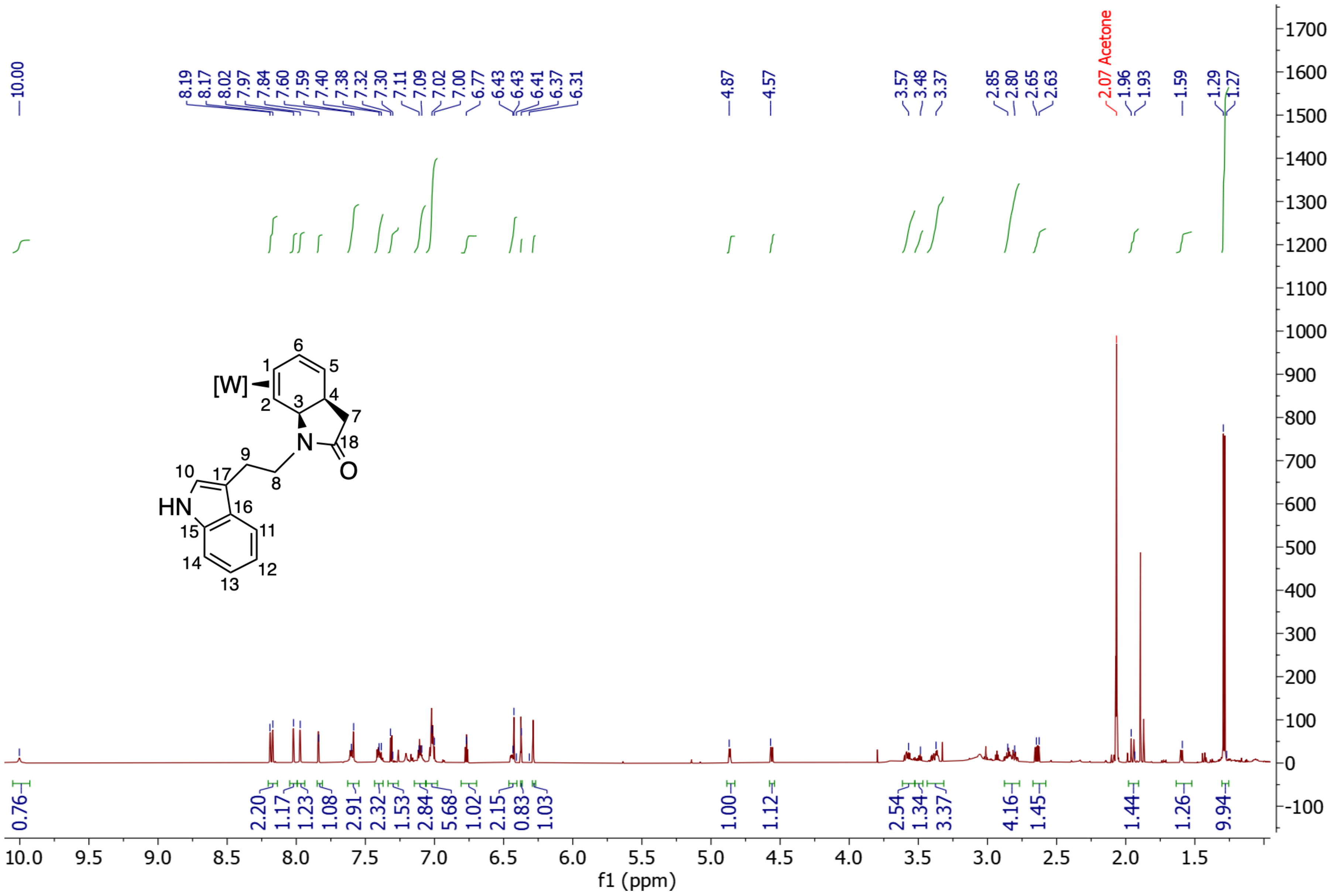

Figure S25: ^{1}H-NMR ($(CD_3)_2CO$) of Compound 21.

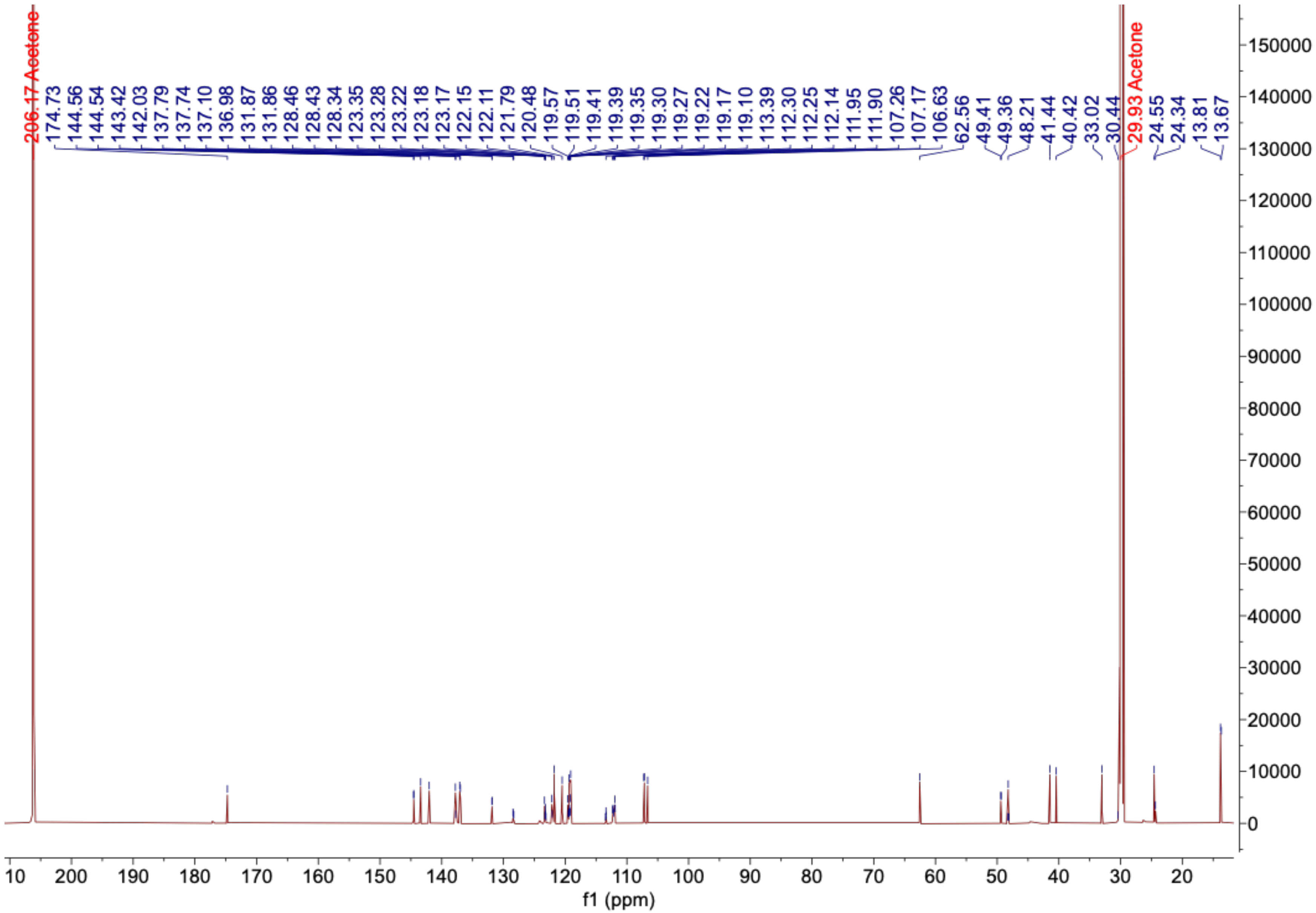

Figure S26: ^{13}C-NMR ($(CD_3)_2CO$) of Compound 21.

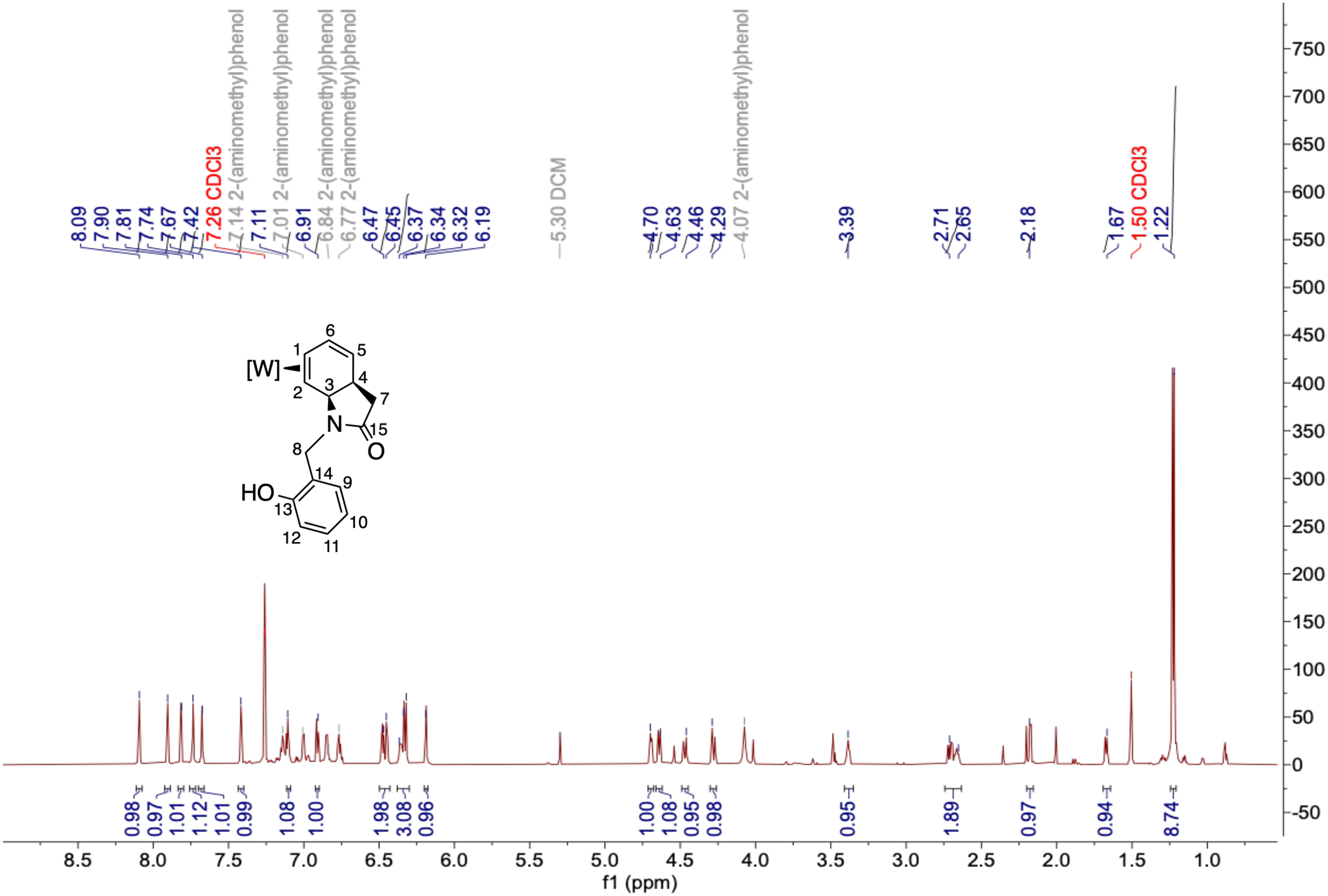

Figure S27: ^{1}H-NMR ($CDCl_3$) of Compound 22.

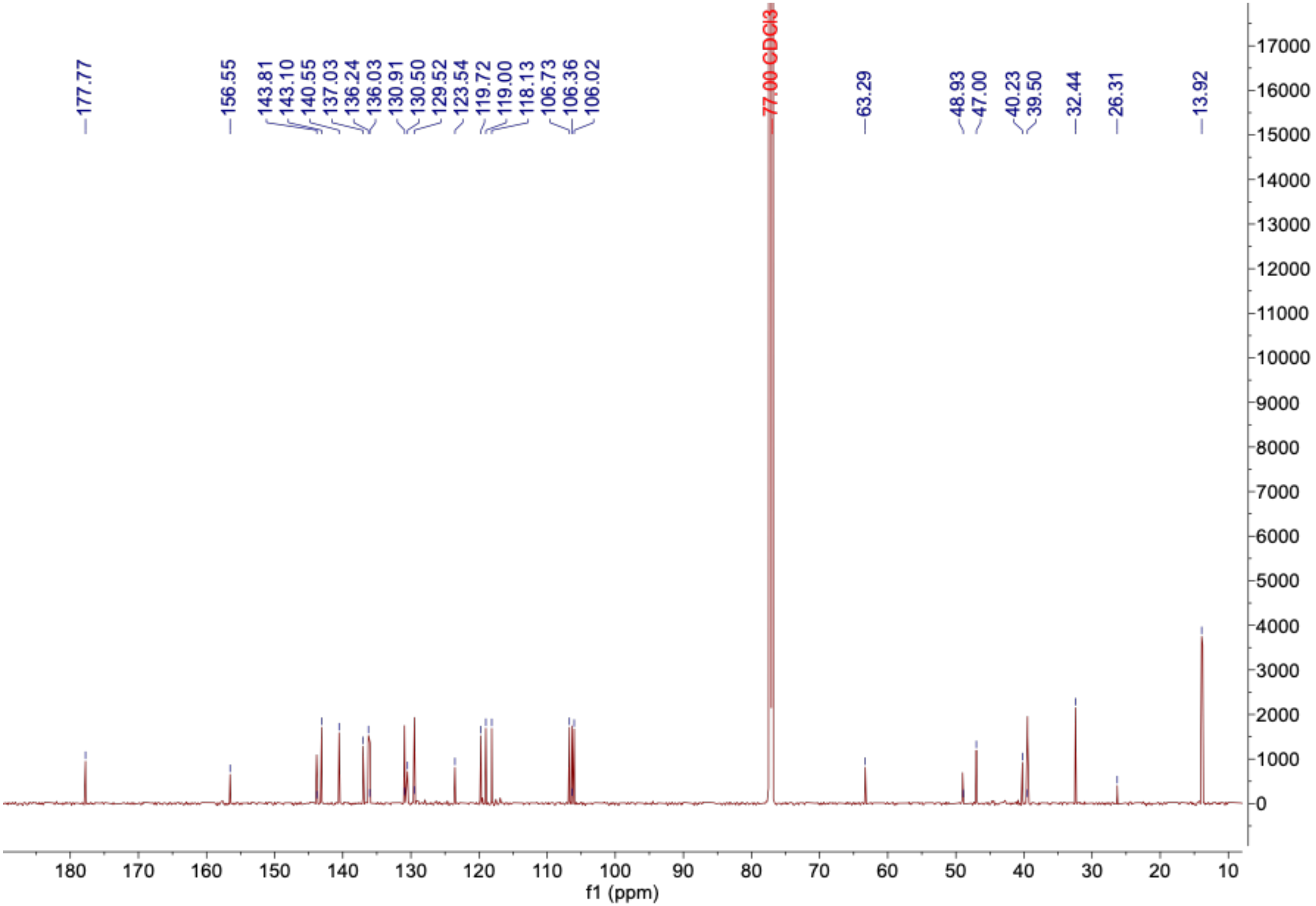

Figure S28: ^{13}C-NMR ($CDCl_3$) of Compound 22.

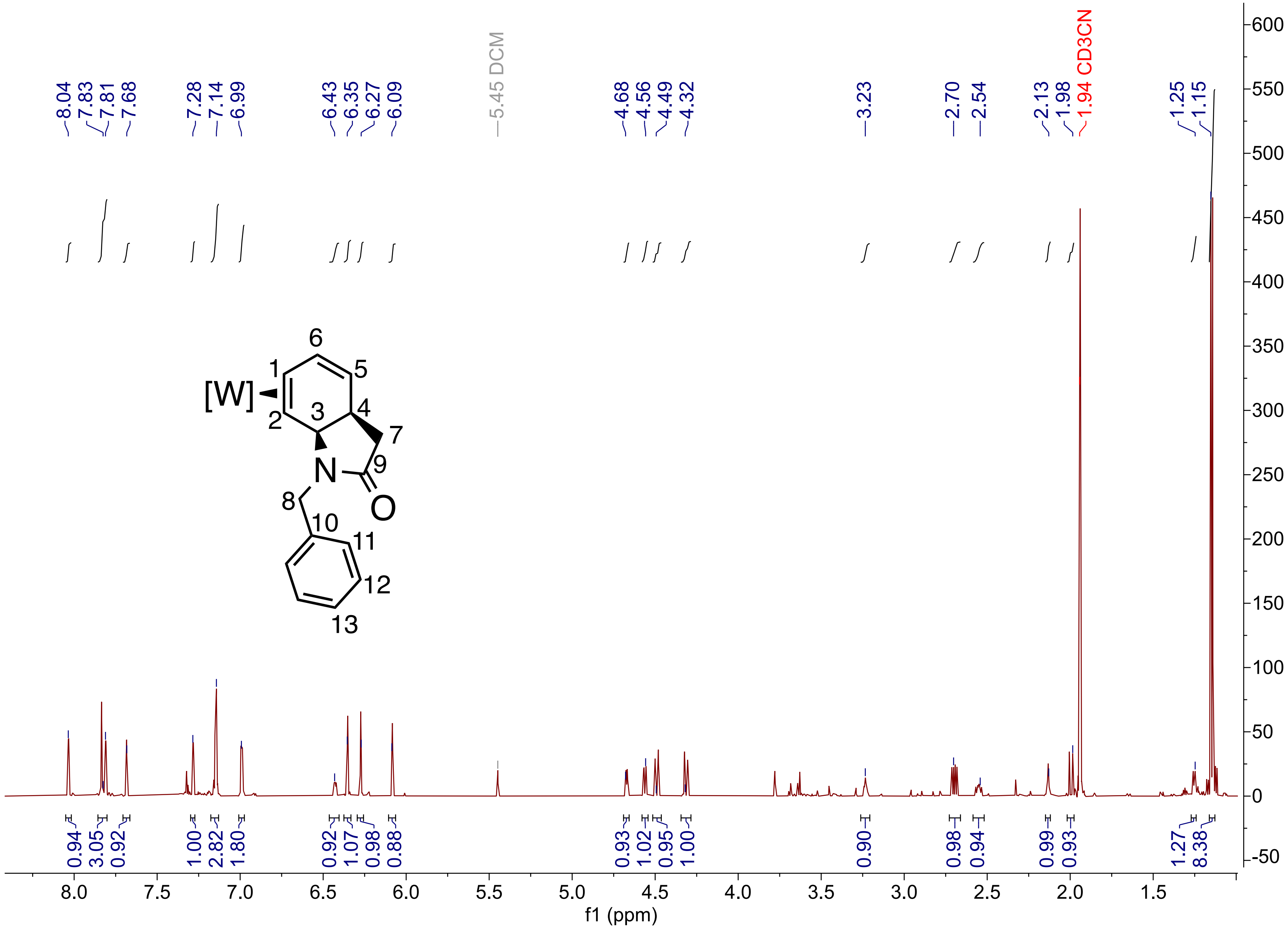

Figure S29: ^{1}H-NMR (CD_3CN) of Compound 23.

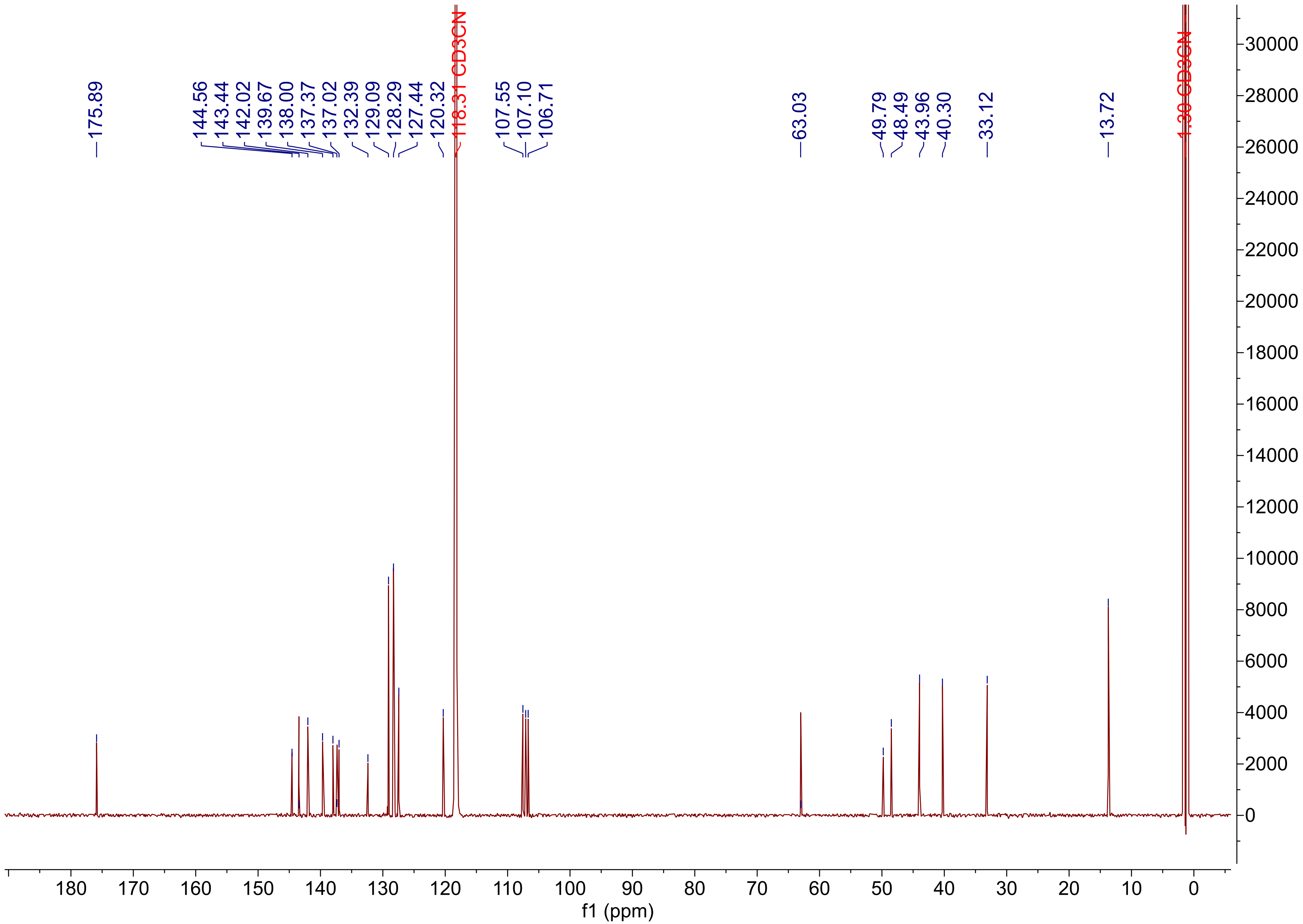

Figure S30: ^{13}C-NMR (CD_3CN) of Compound 23.

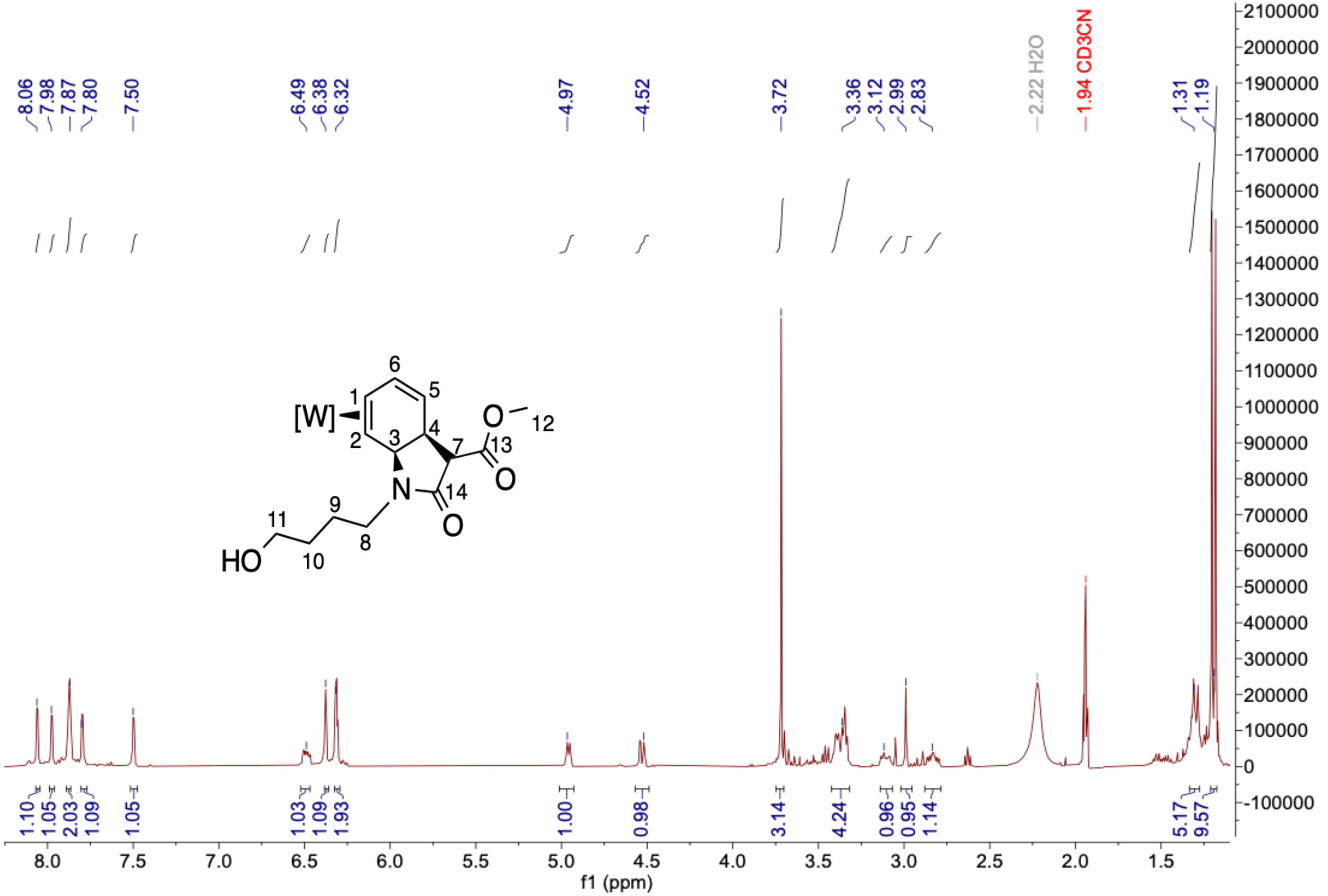

Figure S31: ^{1}H-NMR (CD_3CN) of Compound 24.

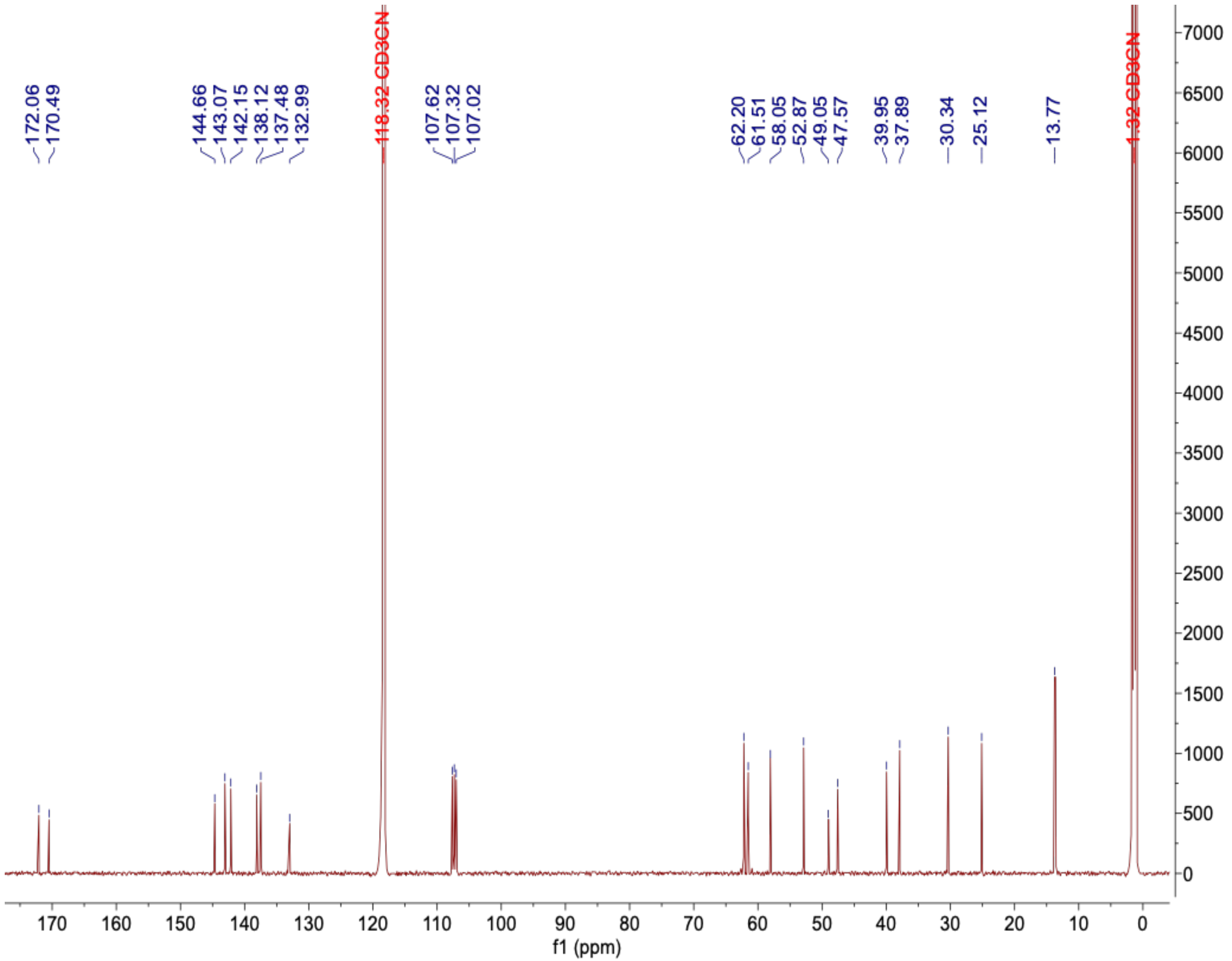

Figure S32: ^{13}C-NMR (CD_3CN) of Compound 24.

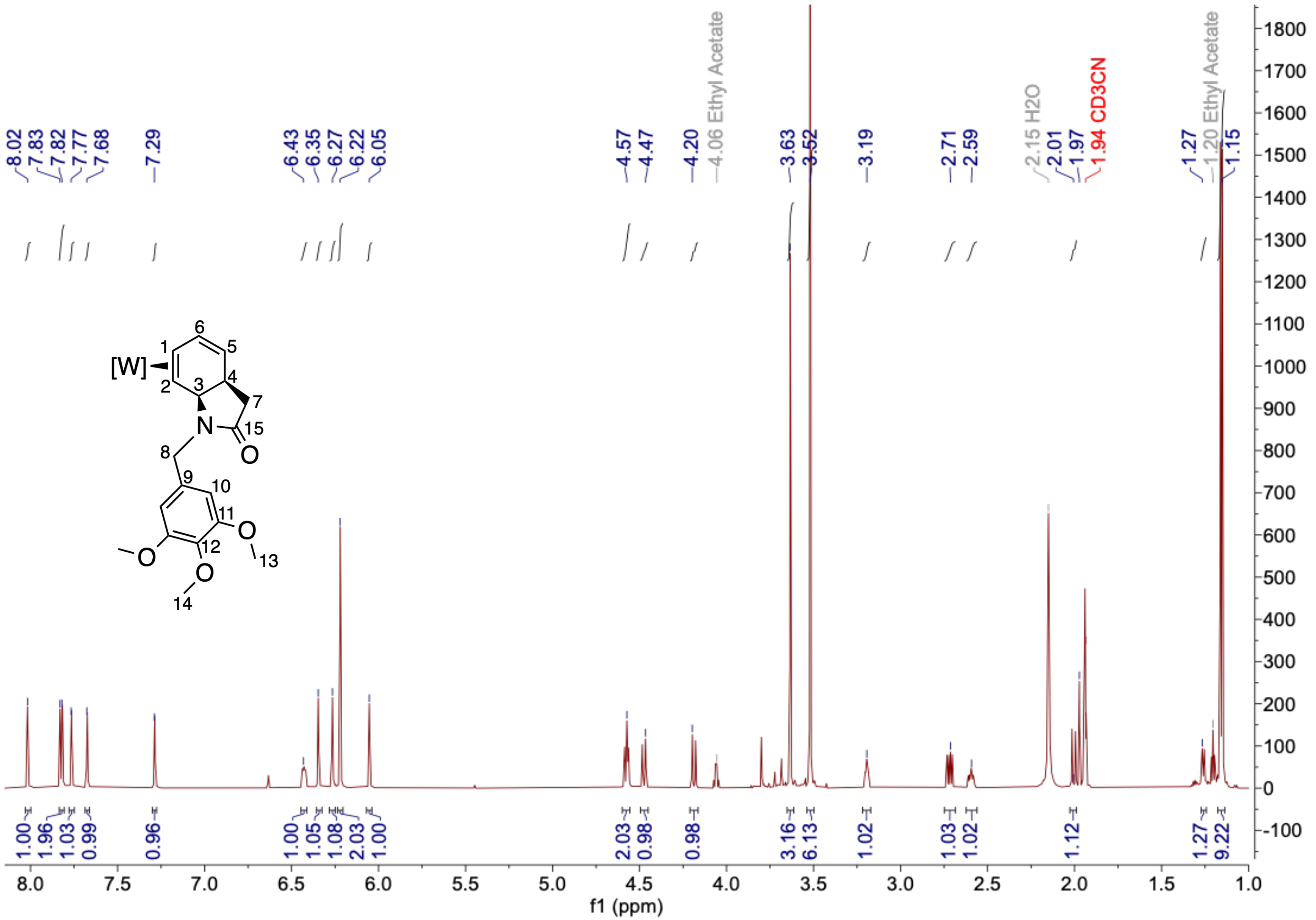

Figure S33: ^{1}H-NMR (CD_3CN) of Compound 25.

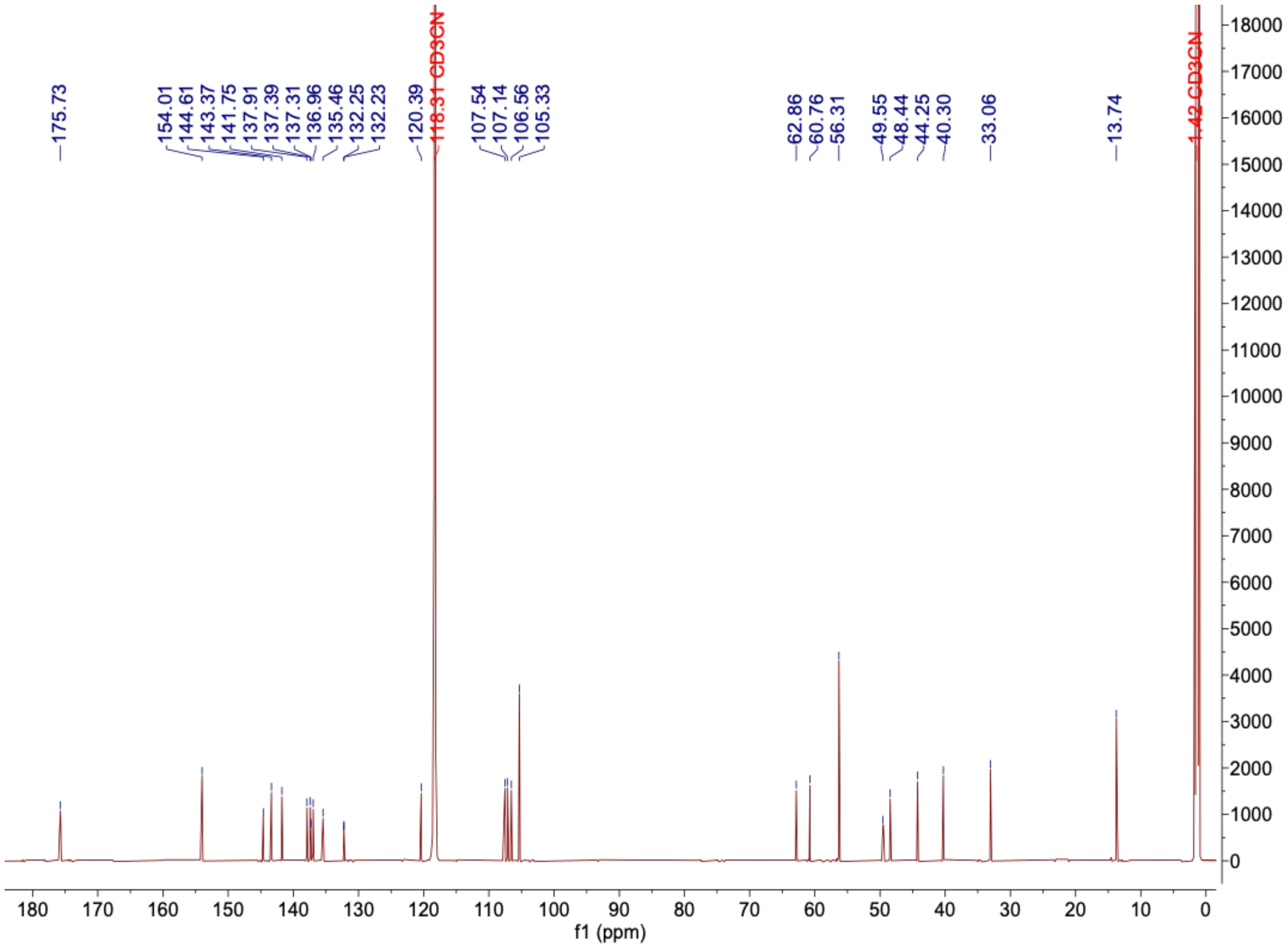

Figure S34: ^{13}C-NMR (CD_3CN) of Compound 25.

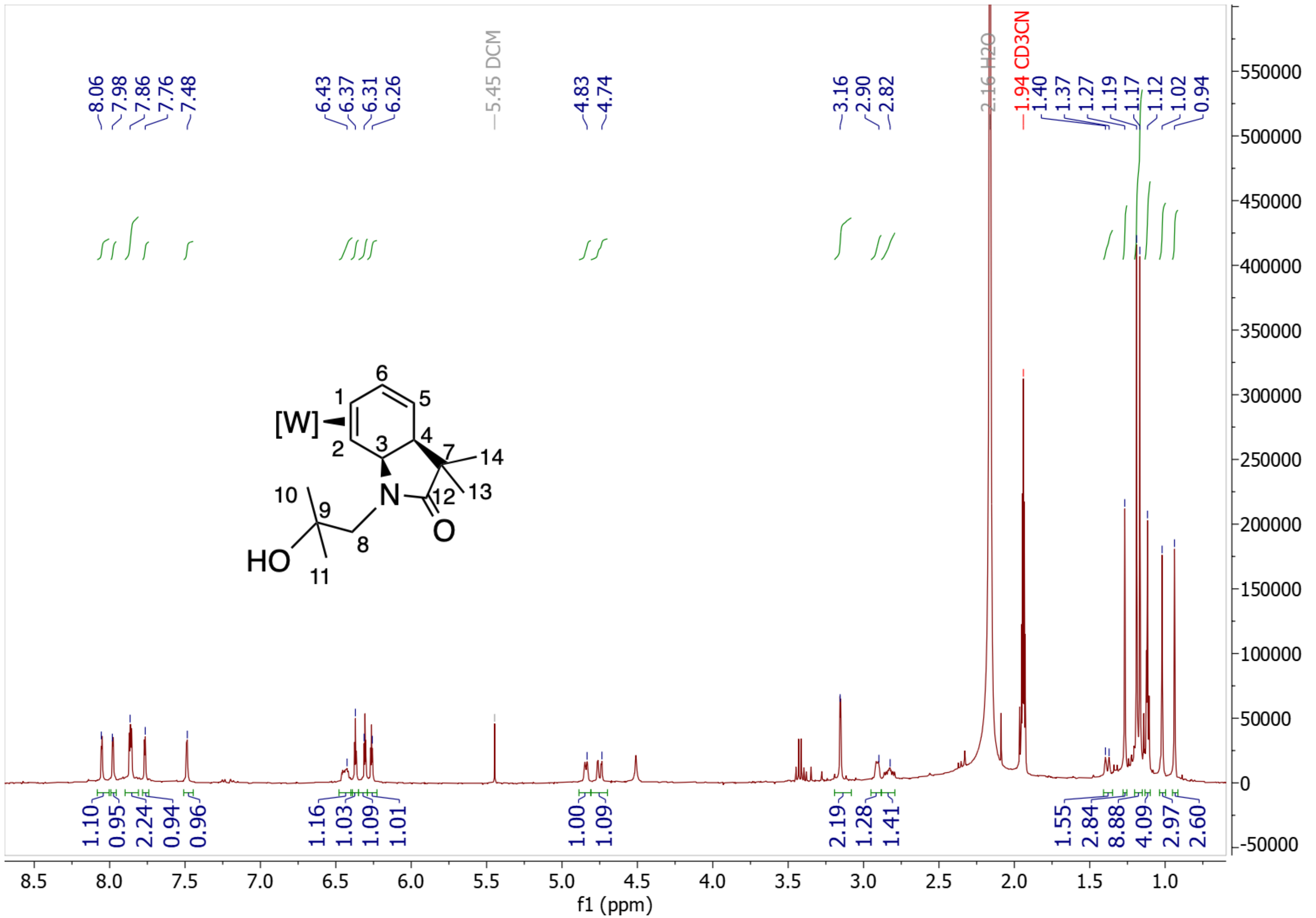

Figure S35: ^{1}H-NMR (CD_3CN) of Compound 26.

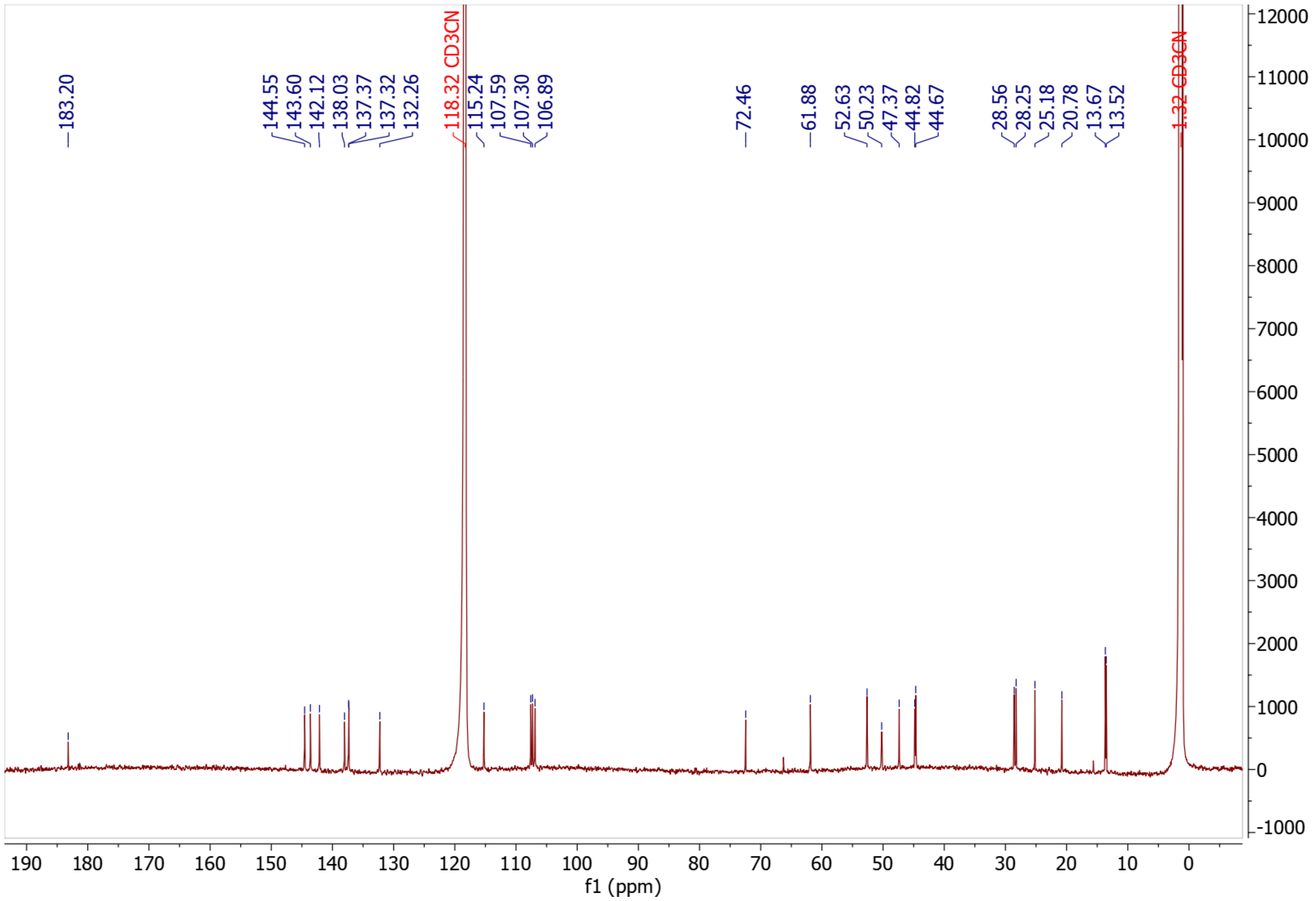

Figure S36: ^{13}C-NMR (CD_3CN) of Compound 26.

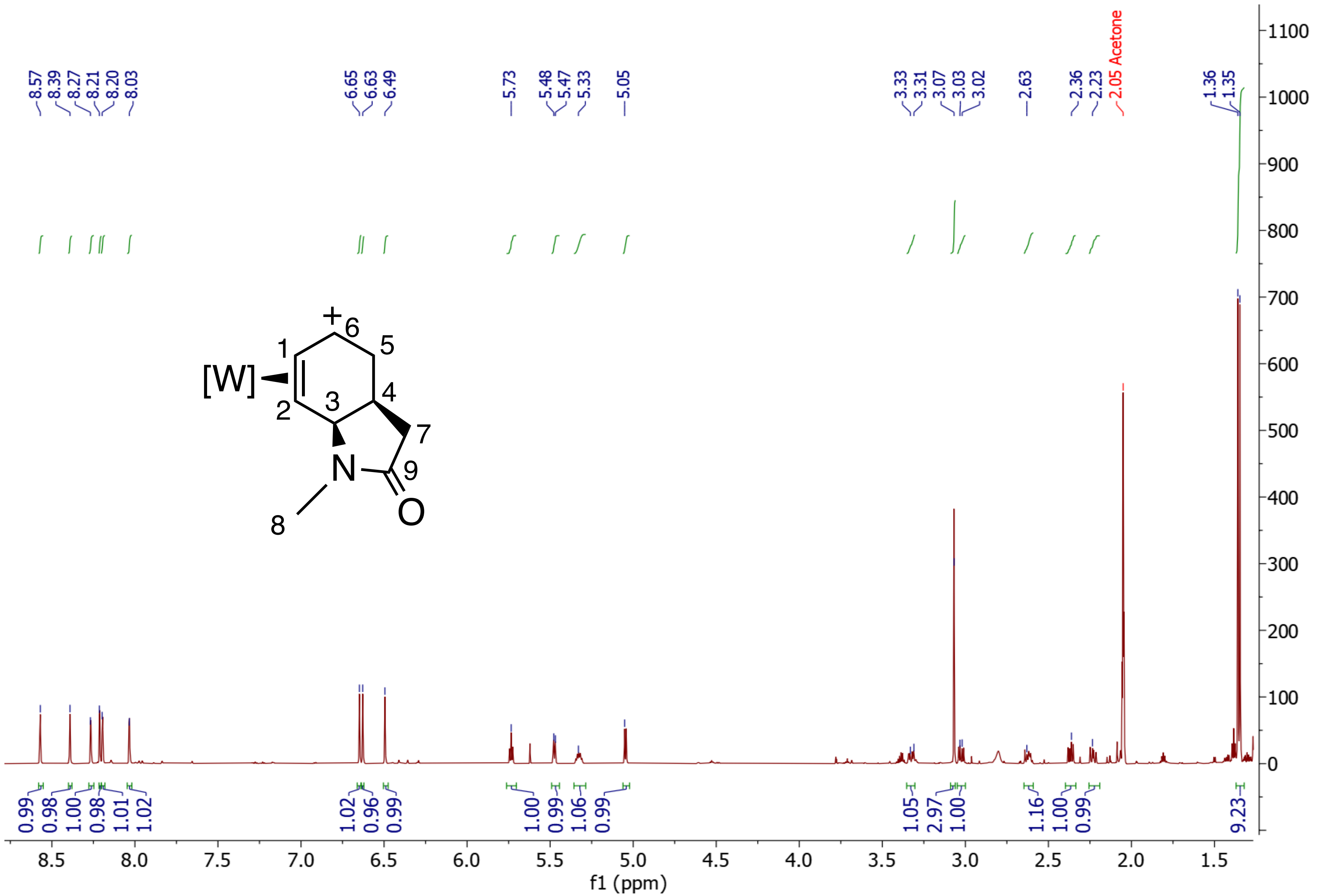

Figure S37: ^{1}H-NMR ($(CD_3)_2CO$) of Compound 27.

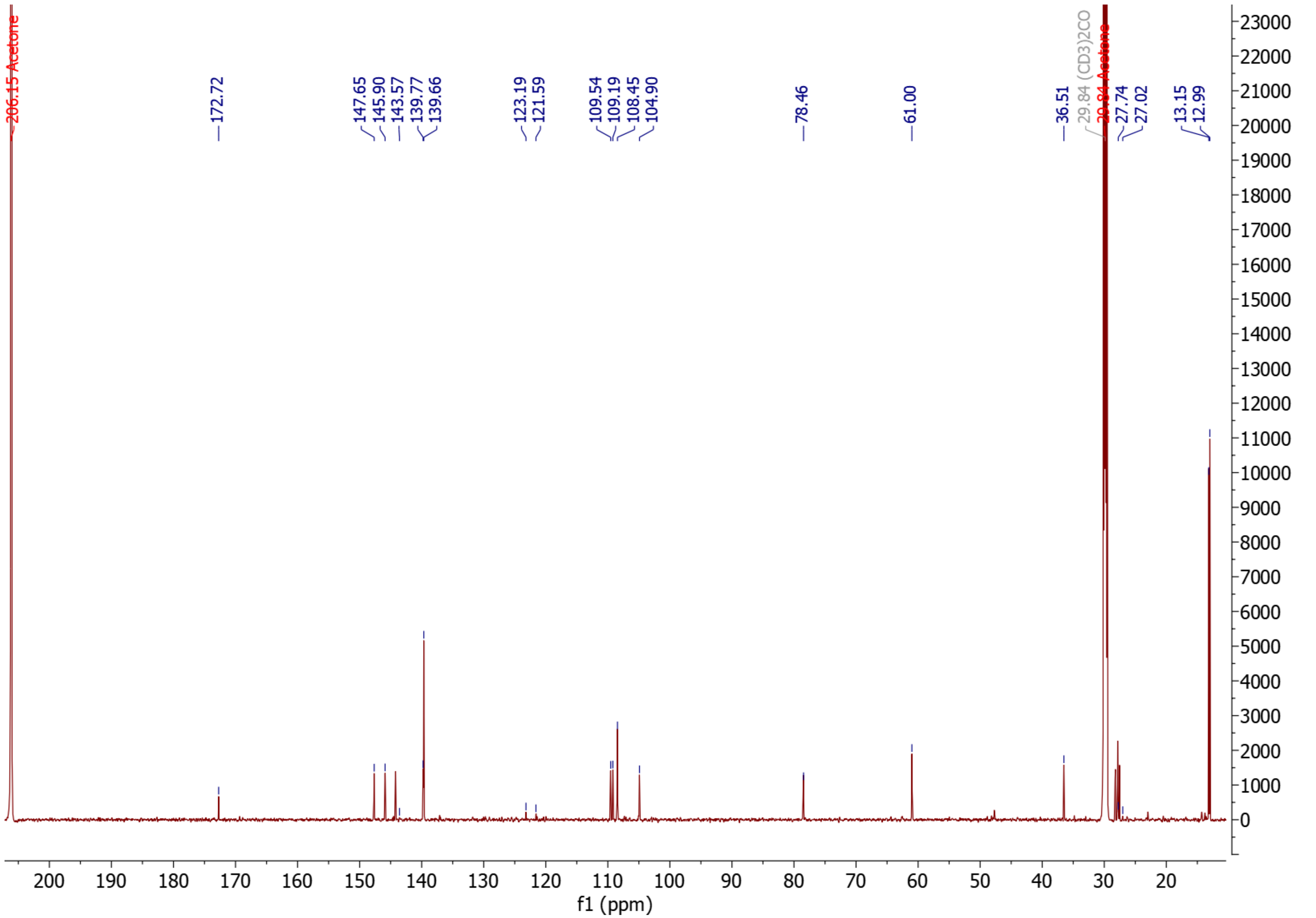

Figure S38: ^{13}C-NMR ($(CD_3)_2CO$) of Compound 27.

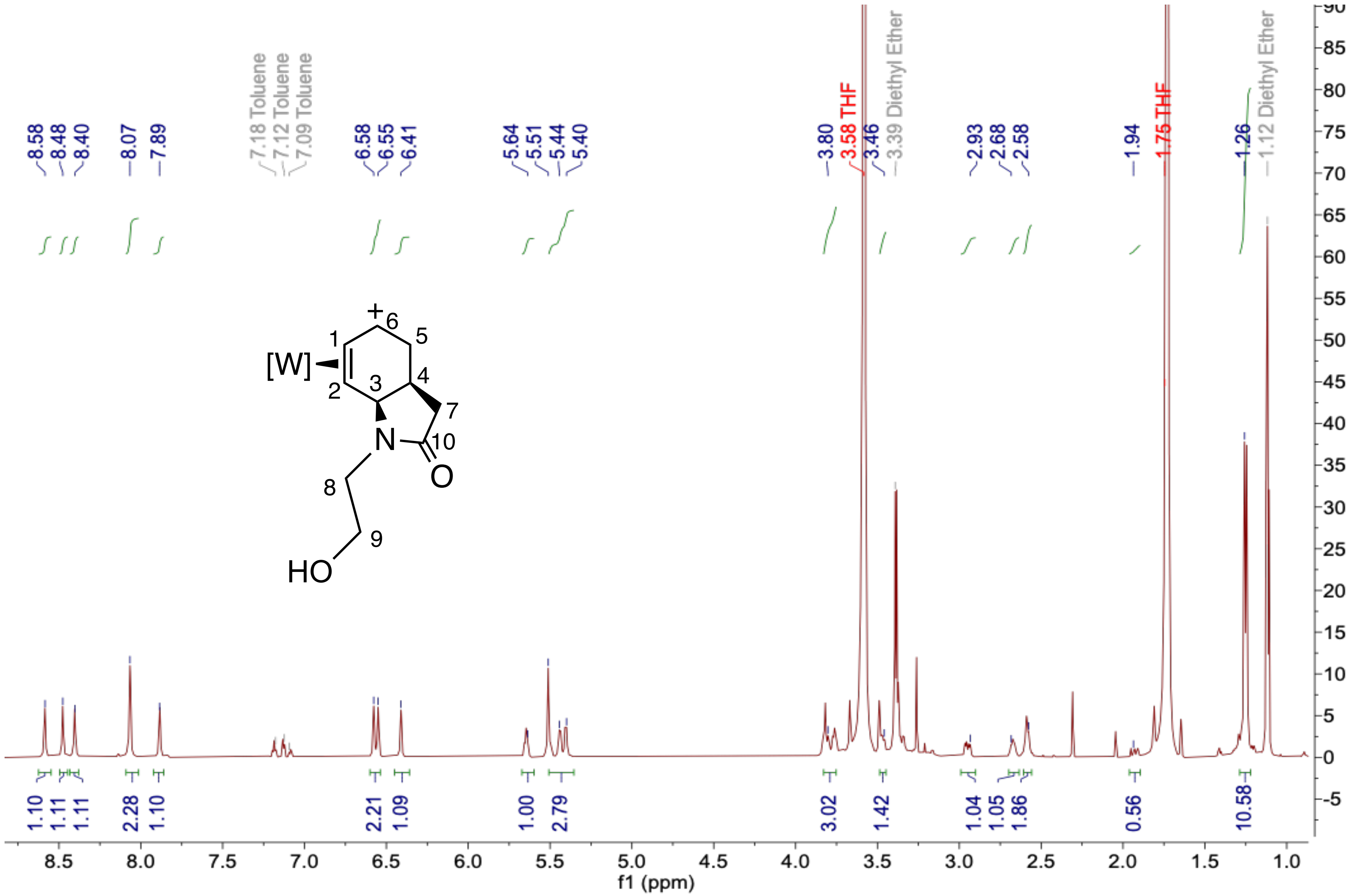

Figure S39: ^{1}H-NMR ($(CD_2)_4O$) of Compound 28.
Complex is unstable in solution. Partial characterization.

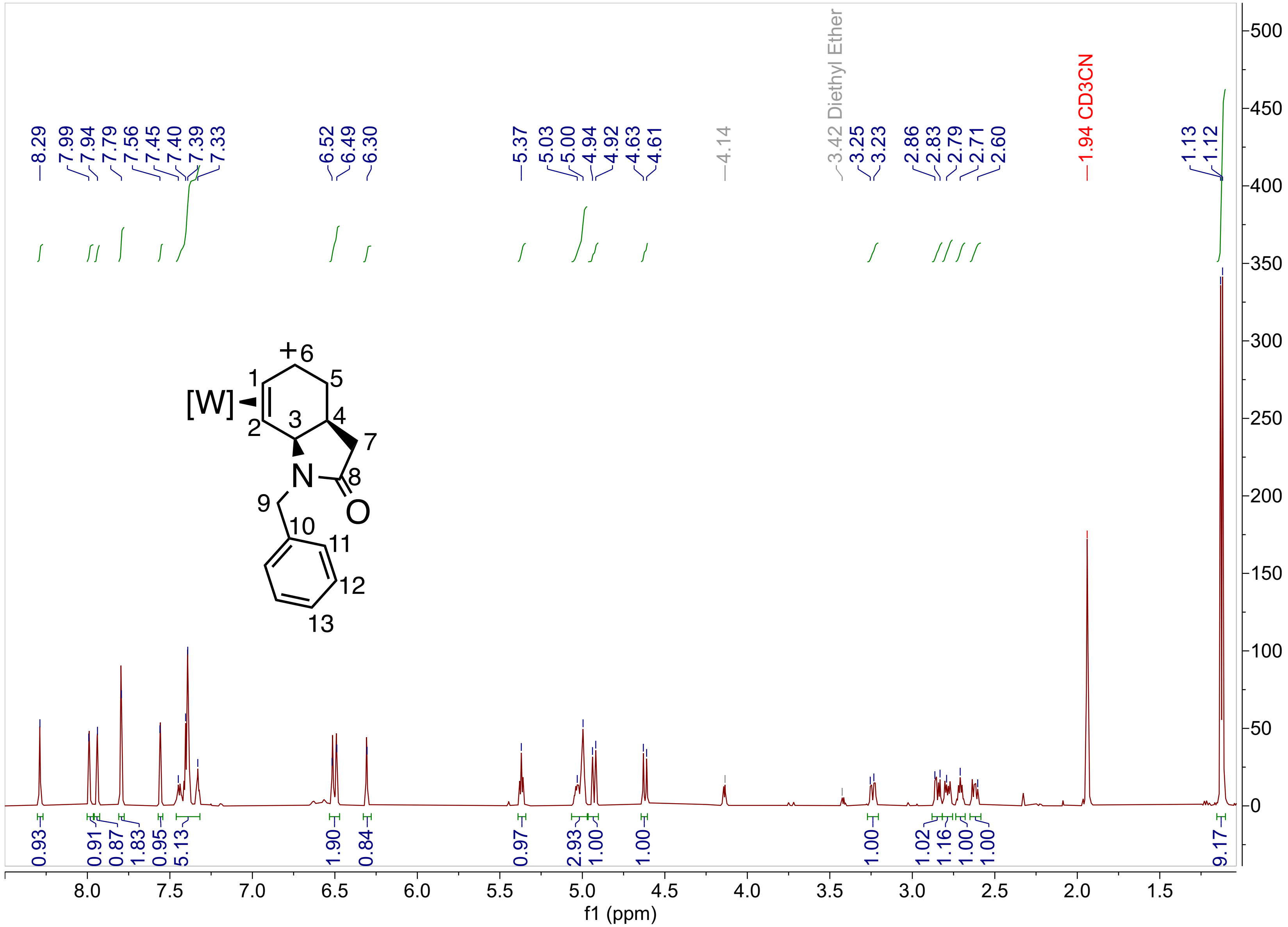

Figure S40: ^{1}H-NMR (CD_3CN) of Compound 29.

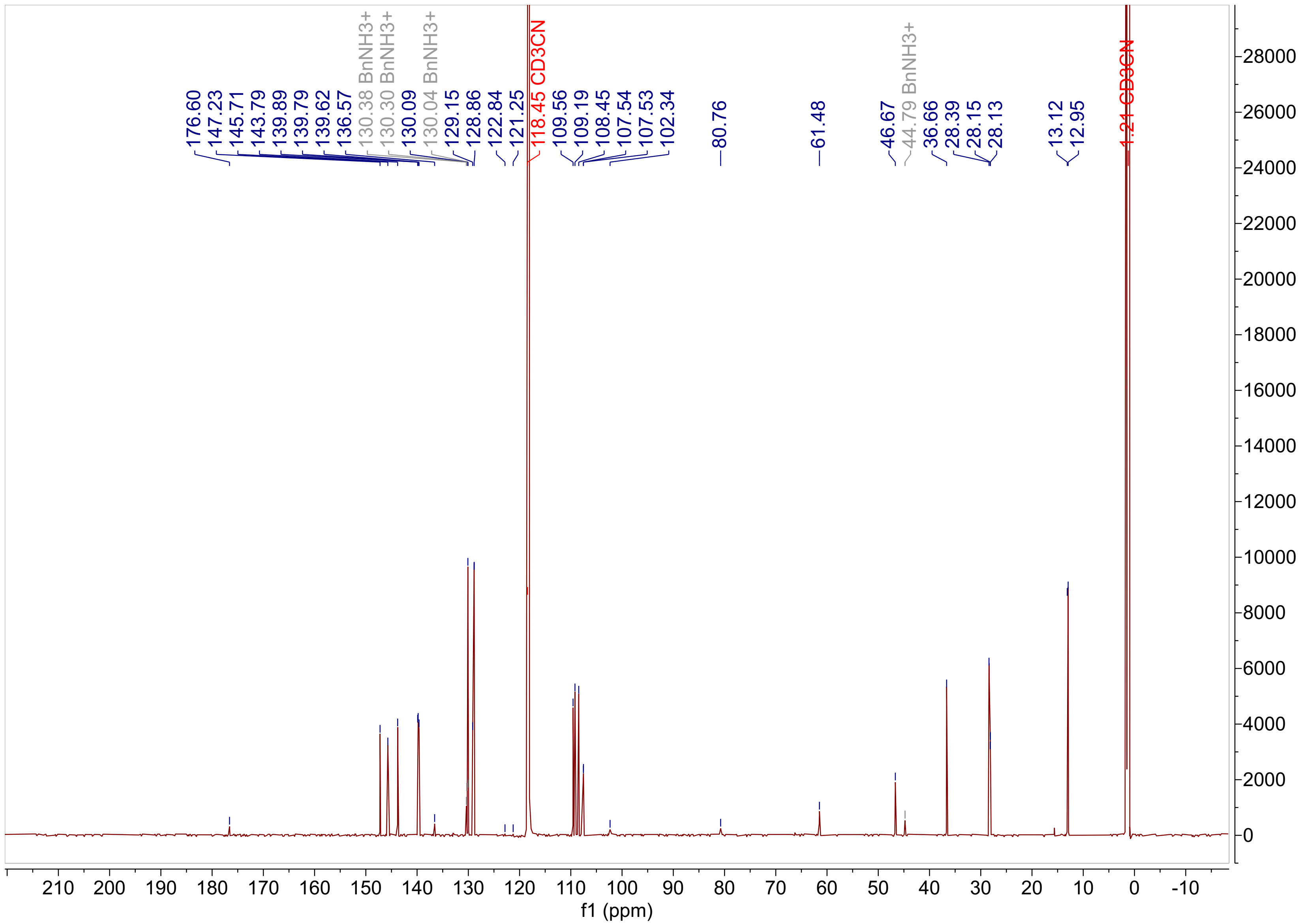

Figure S41: ^{13}C-NMR (CD_3CN) of Compound 29.

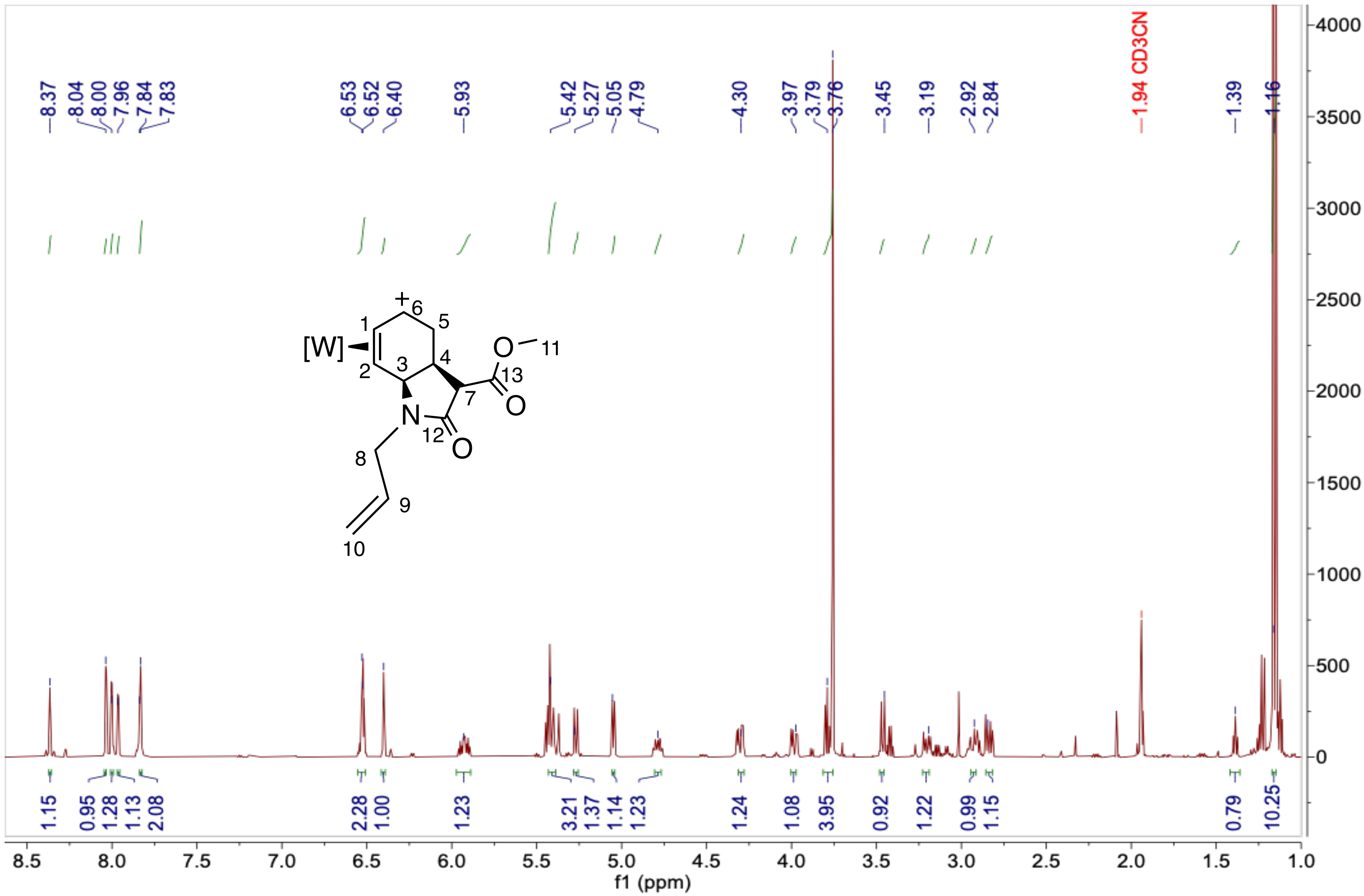

Figure S42: ^{1}H-NMR (CD_3CN) of Compound 30.
Complex is unstable in solution. Partial characterization.

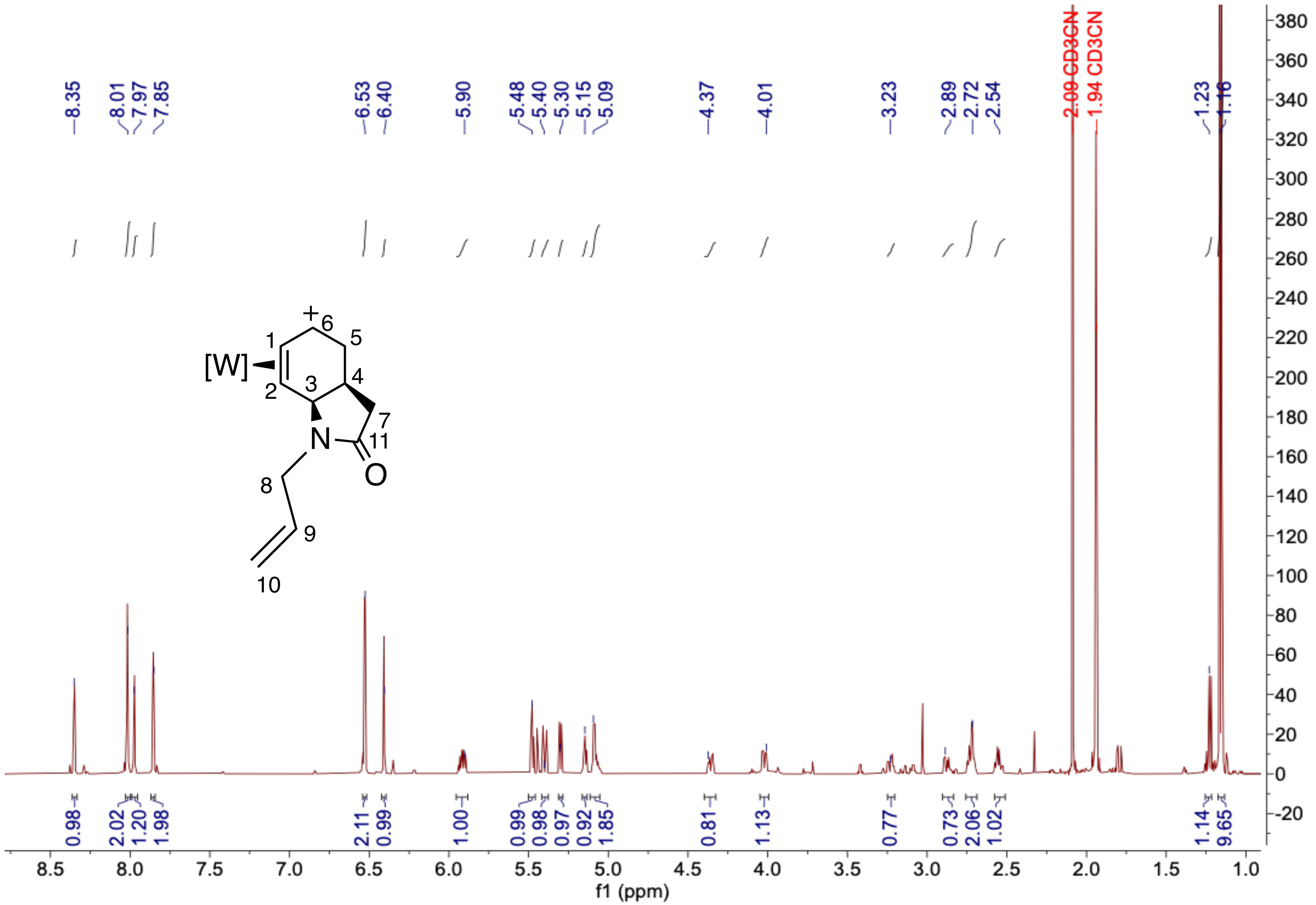

Figure S43: ^{1}H-NMR (CD_3CN) of Compound 31.
Complex is unstable in solution. Partial Characterization.

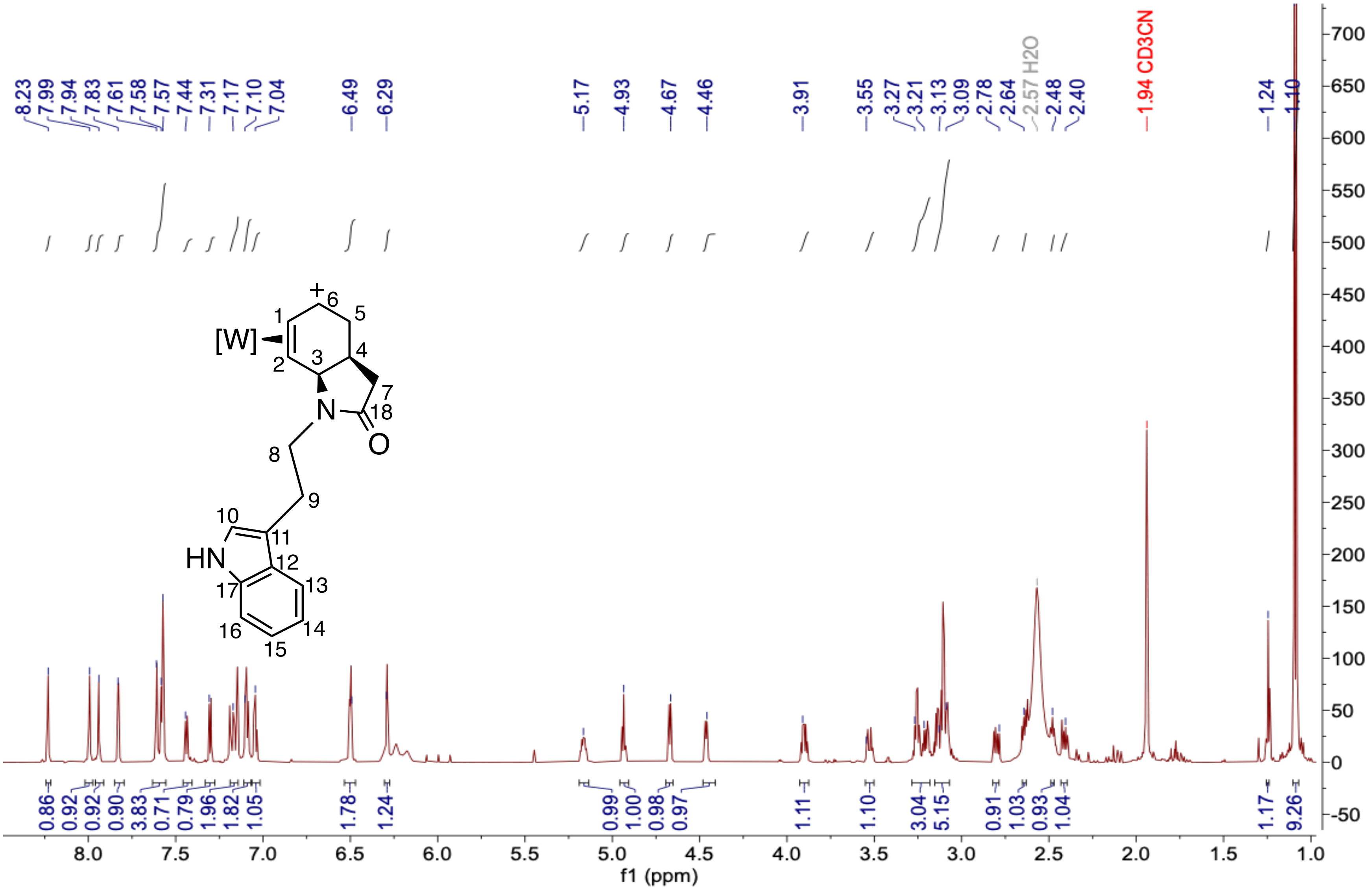

Figure S44: ^{1}H-NMR (CD_3CN) of Compound 32.
Complex is unstable in solution. Partial characterization.

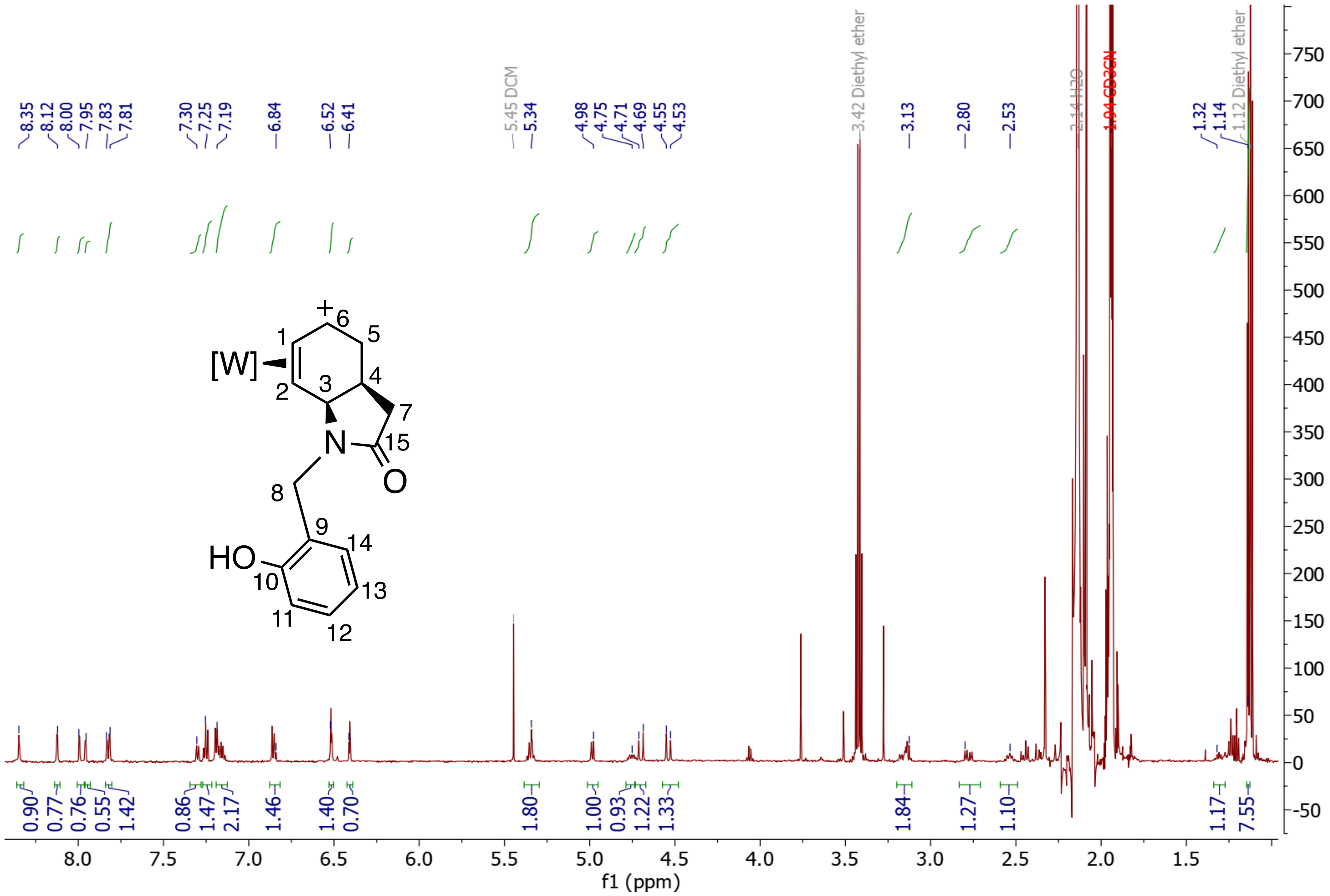

Figure S45: ^{1}H-NMR (CD_3CN) of Compound 33
Complex is unstable in solution. Partial characterization.

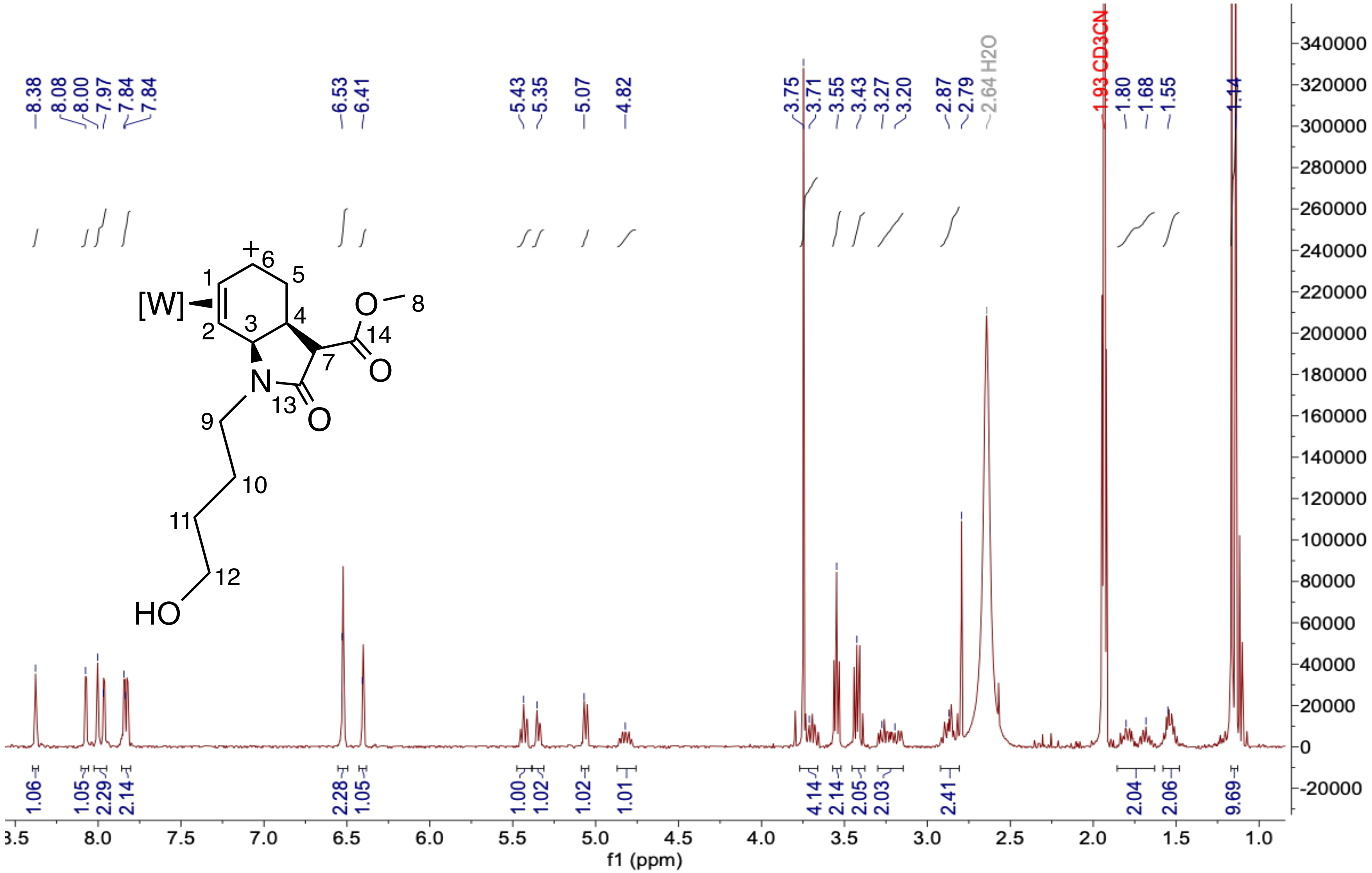

Figure S46: ^{1}H-NMR (CD_3CN) of Compound 34.
Complex is unstable in solution. Partial characterization.

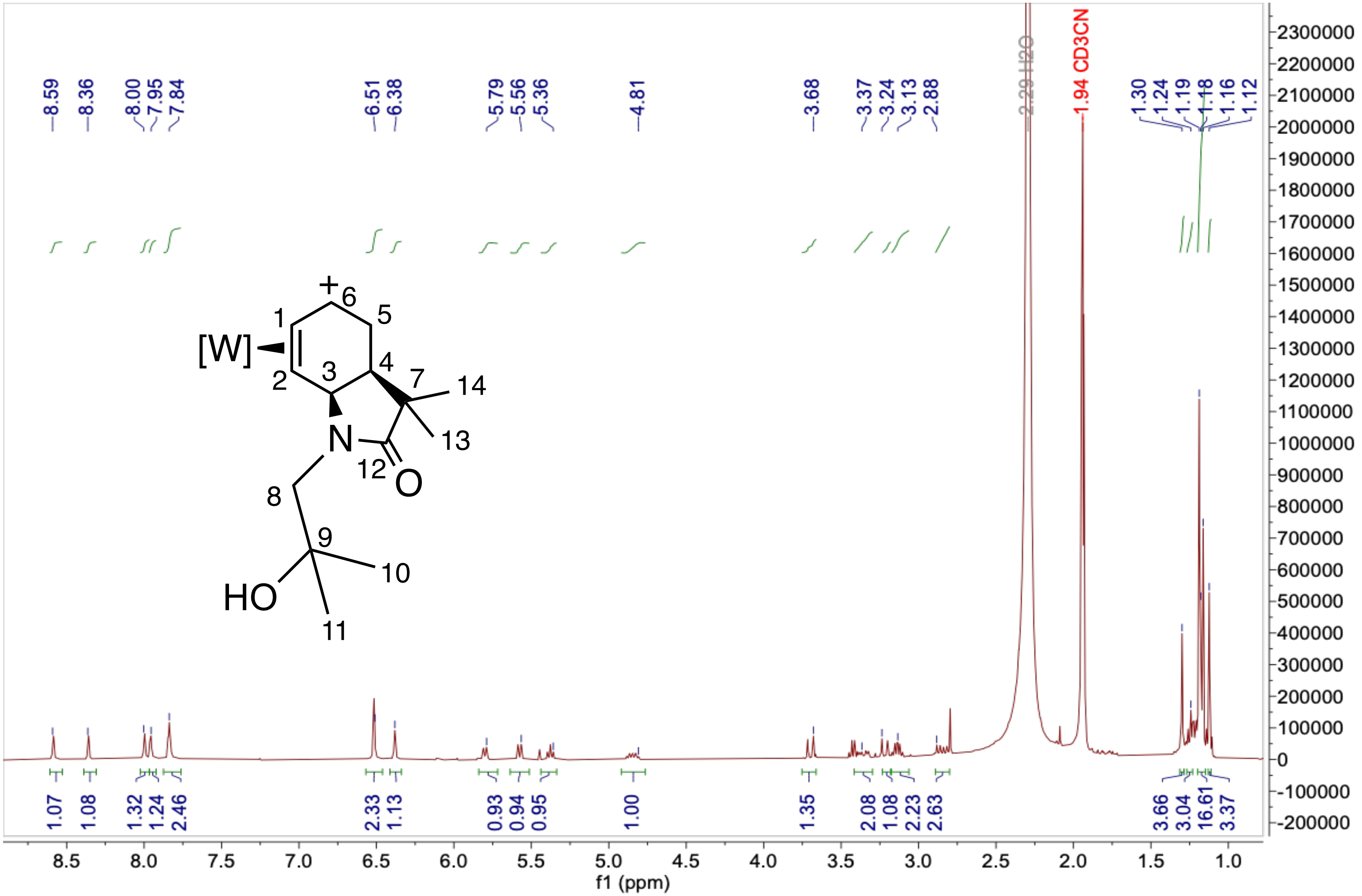

Figure S47: ^{1}H-NMR (CD_3CN) of Compound 35.
Complex is unstable in solution. Partial characterization.

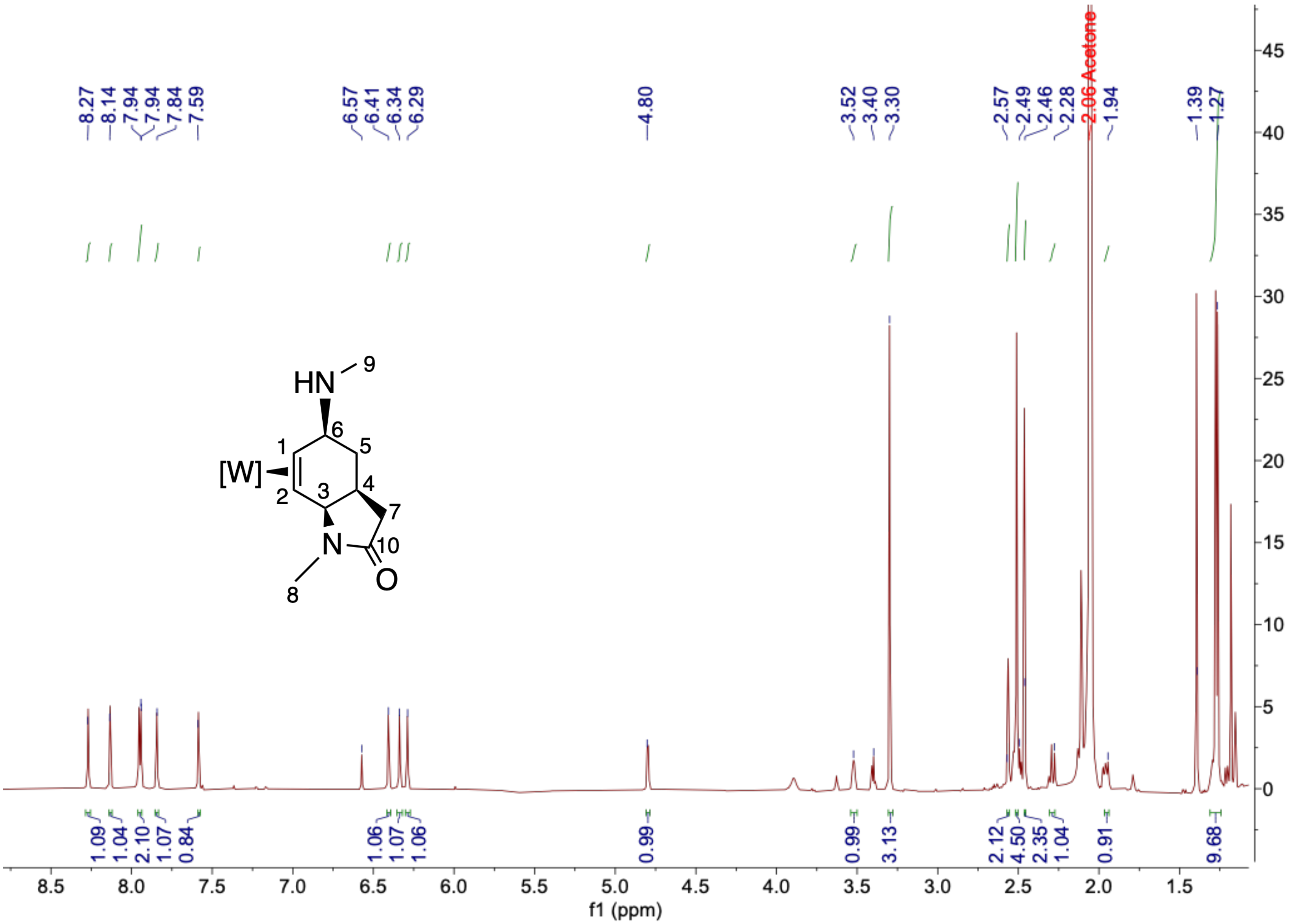

Figure S48: ^{1}H-NMR ($(CD_3)_2CO$) of Compound 36.

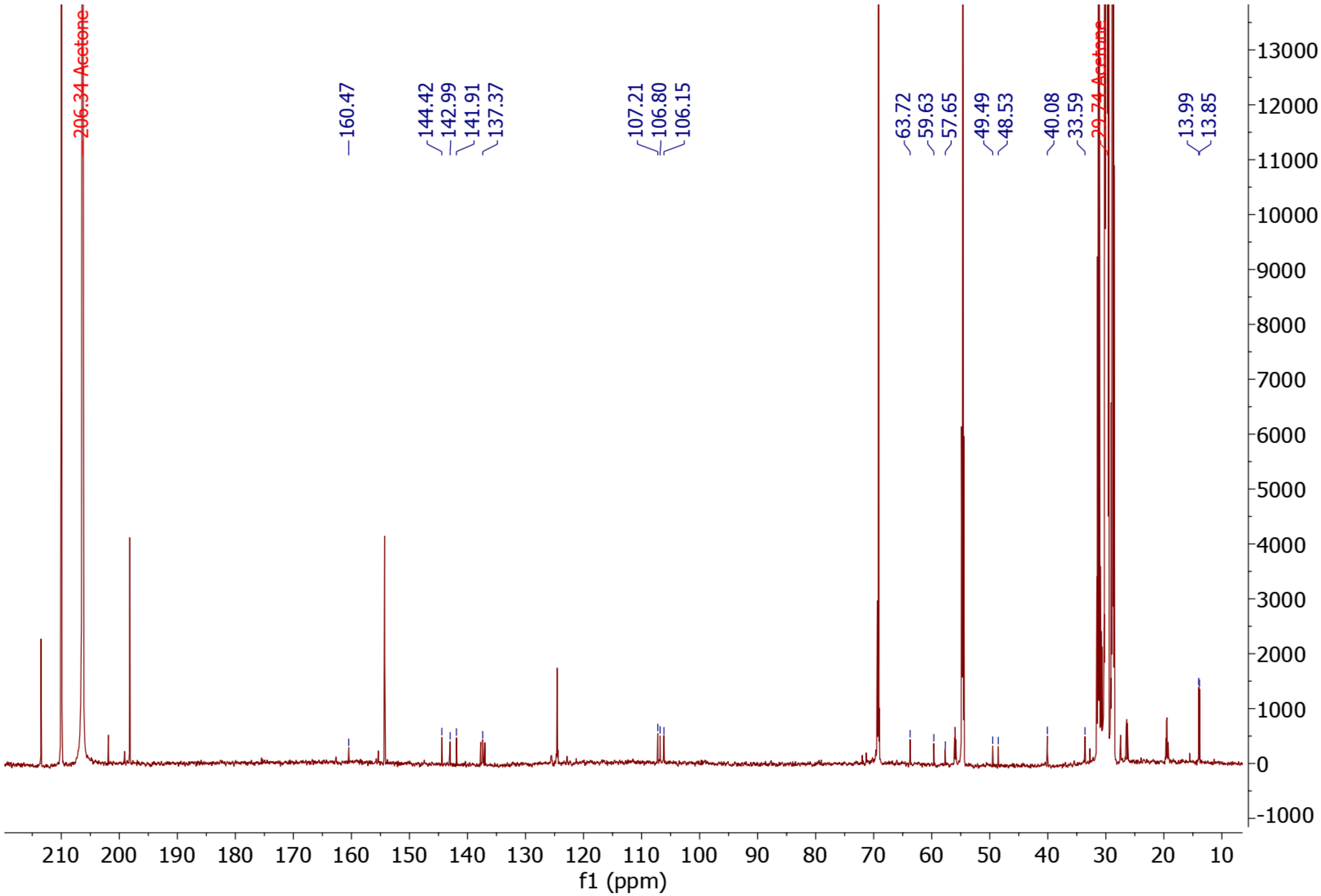

Figure S49: ^{13}C-NMR ($(CD_3)_2CO$) of Compound 36. Complex undergoes decomposition during 2D NMR experiment.

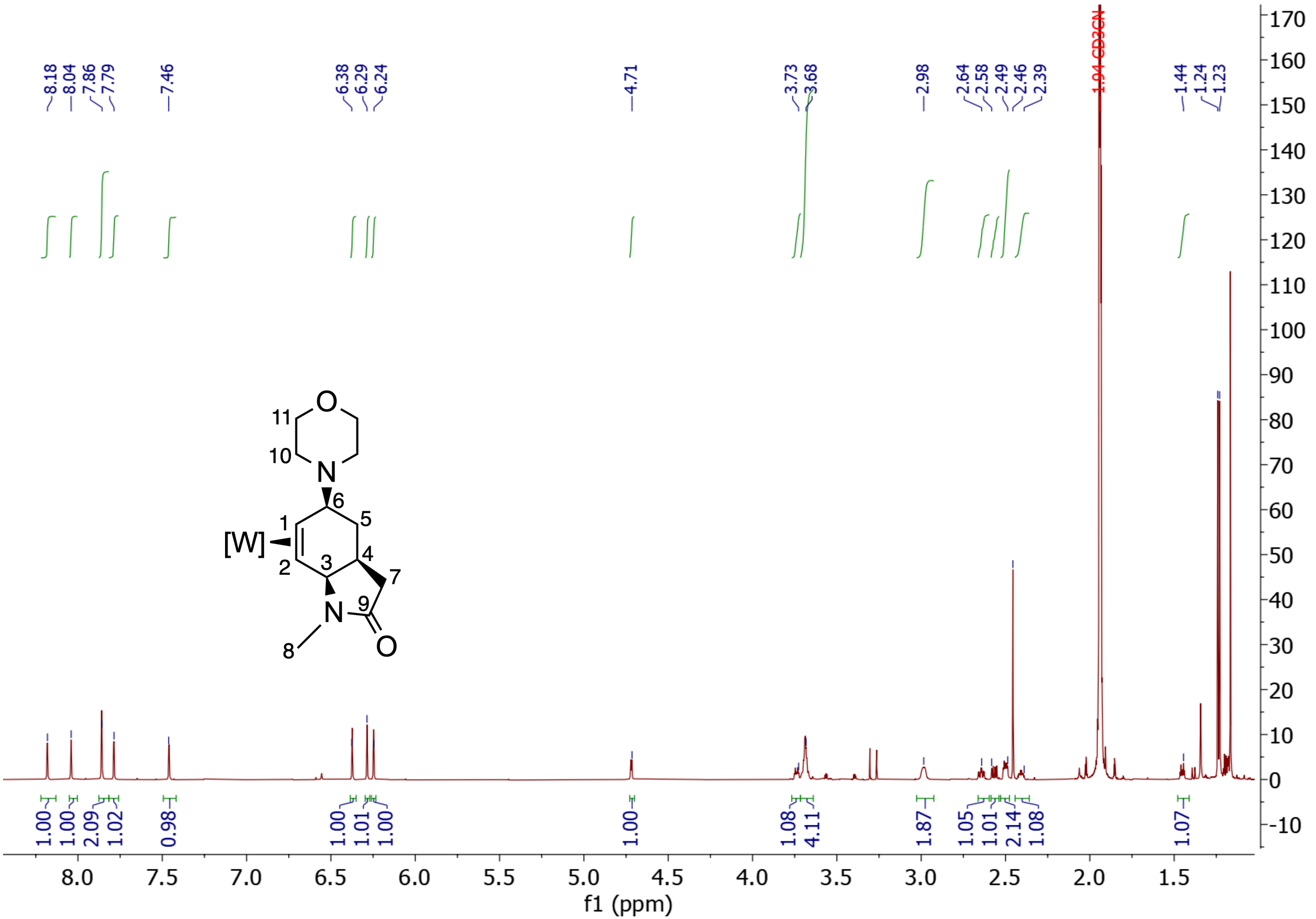

Figure S50: ^{1}H-NMR (CD_3CN) of Compound 37.

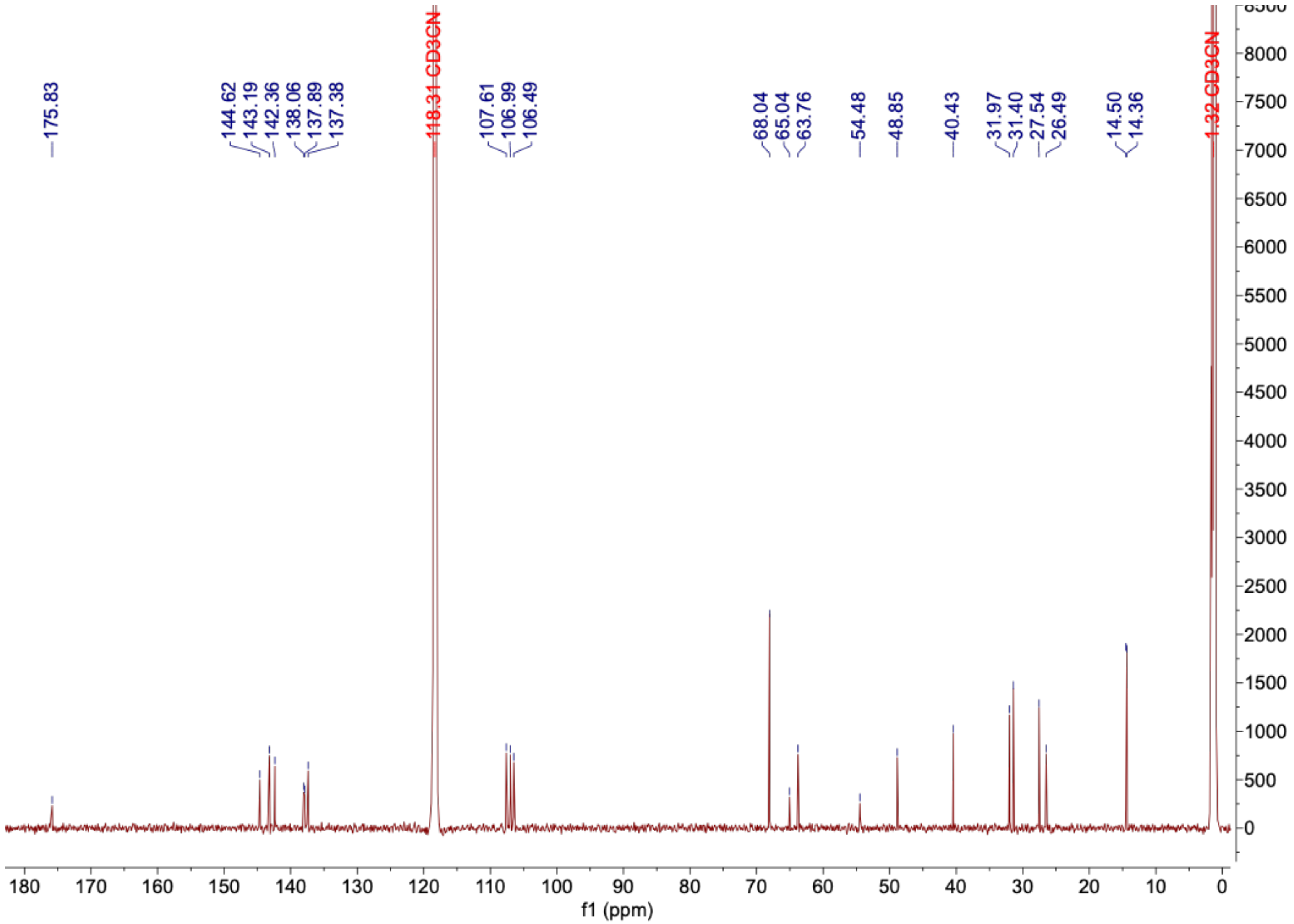

Figure S51: ^{13}C-NMR (CD_3CN) of Compound 37.

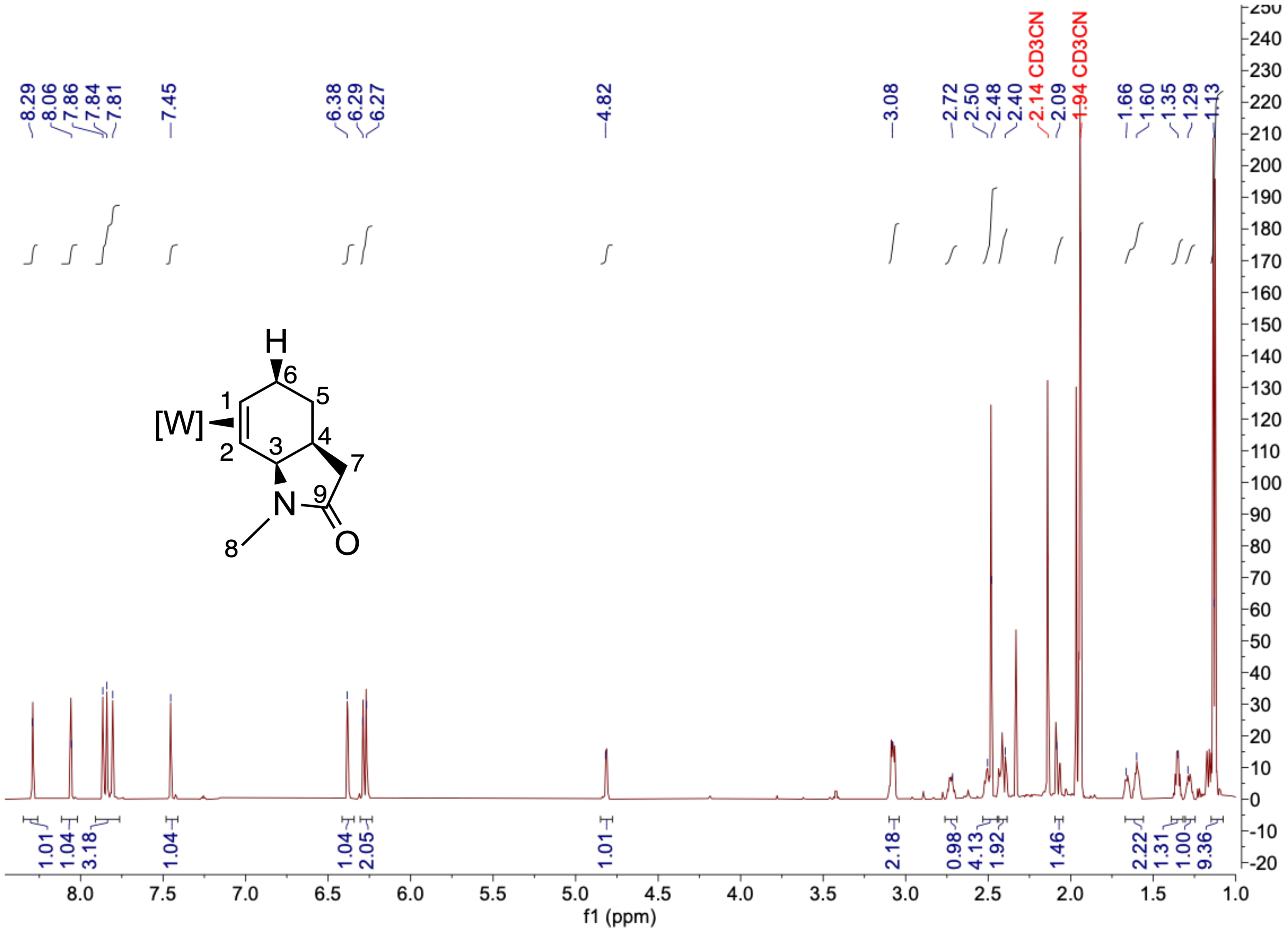

Figure S52: ^{1}H-NMR (CD_3CN) of Compound 38.

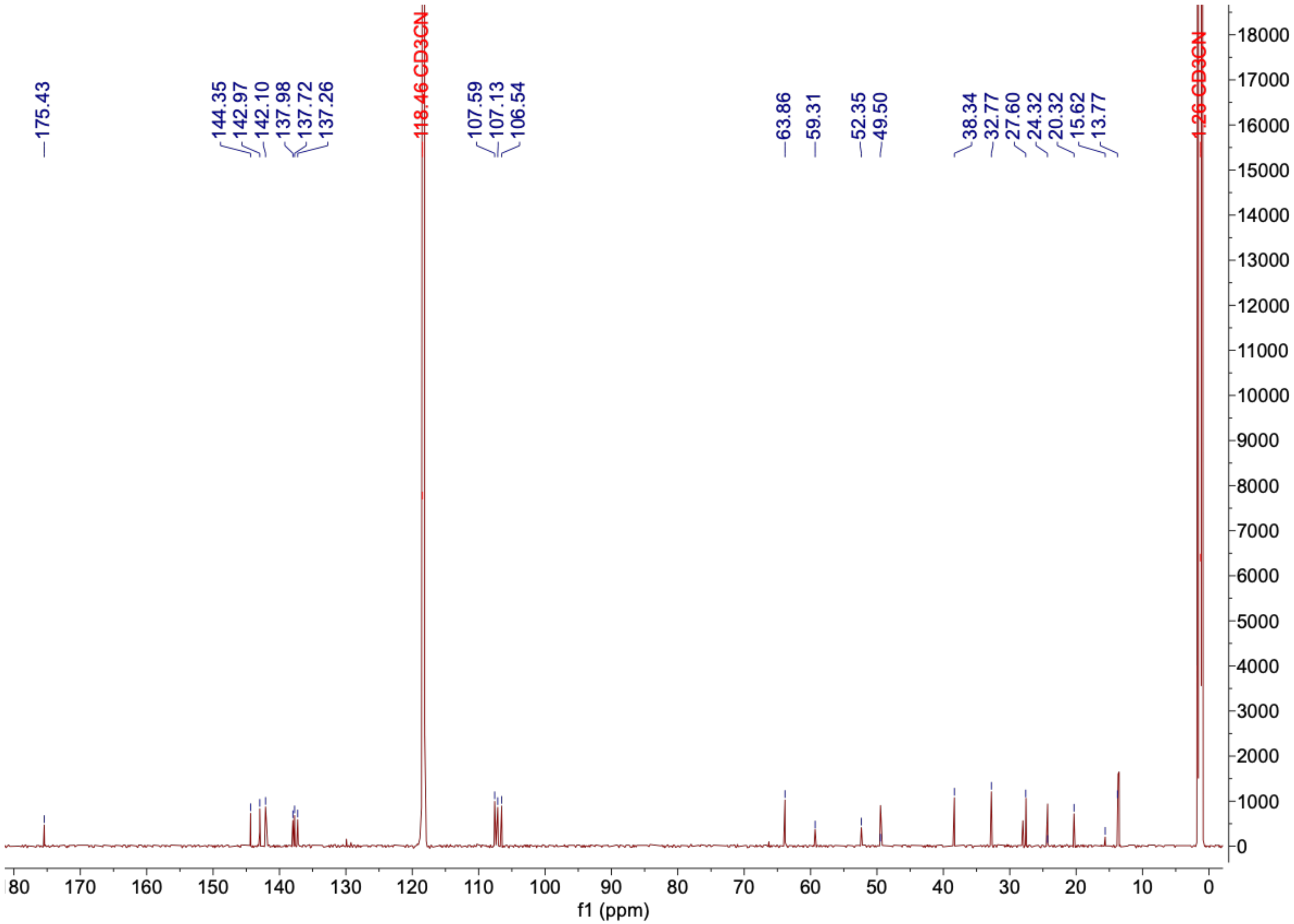

Figure S53: ^{13}C-NMR (CD_3CN) of Compound 38.

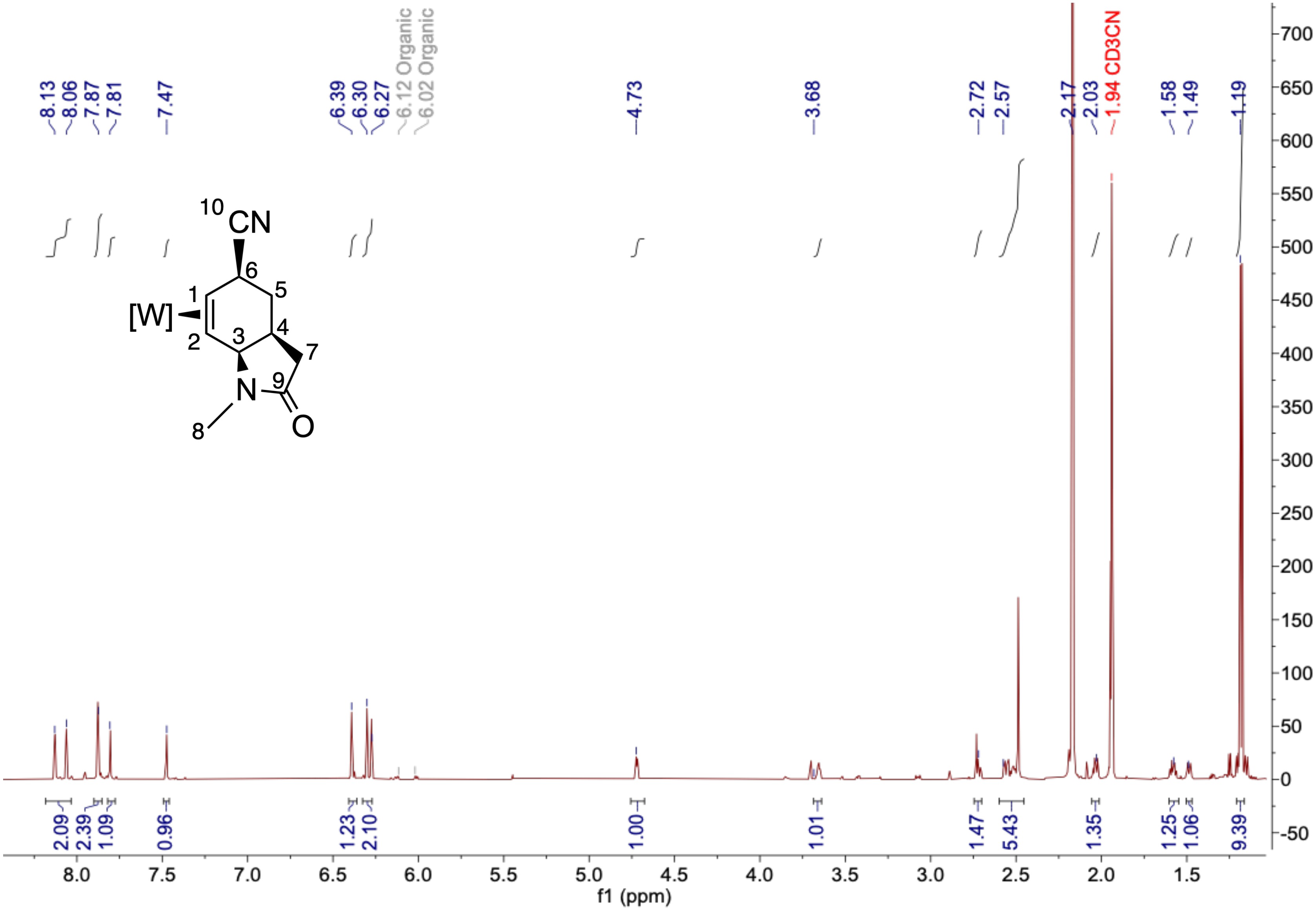

Figure S54: ^{1}H-NMR (CD_3CN) of Compound 39. Whilst in solution, the metal started to decomplex liberating the free organic.

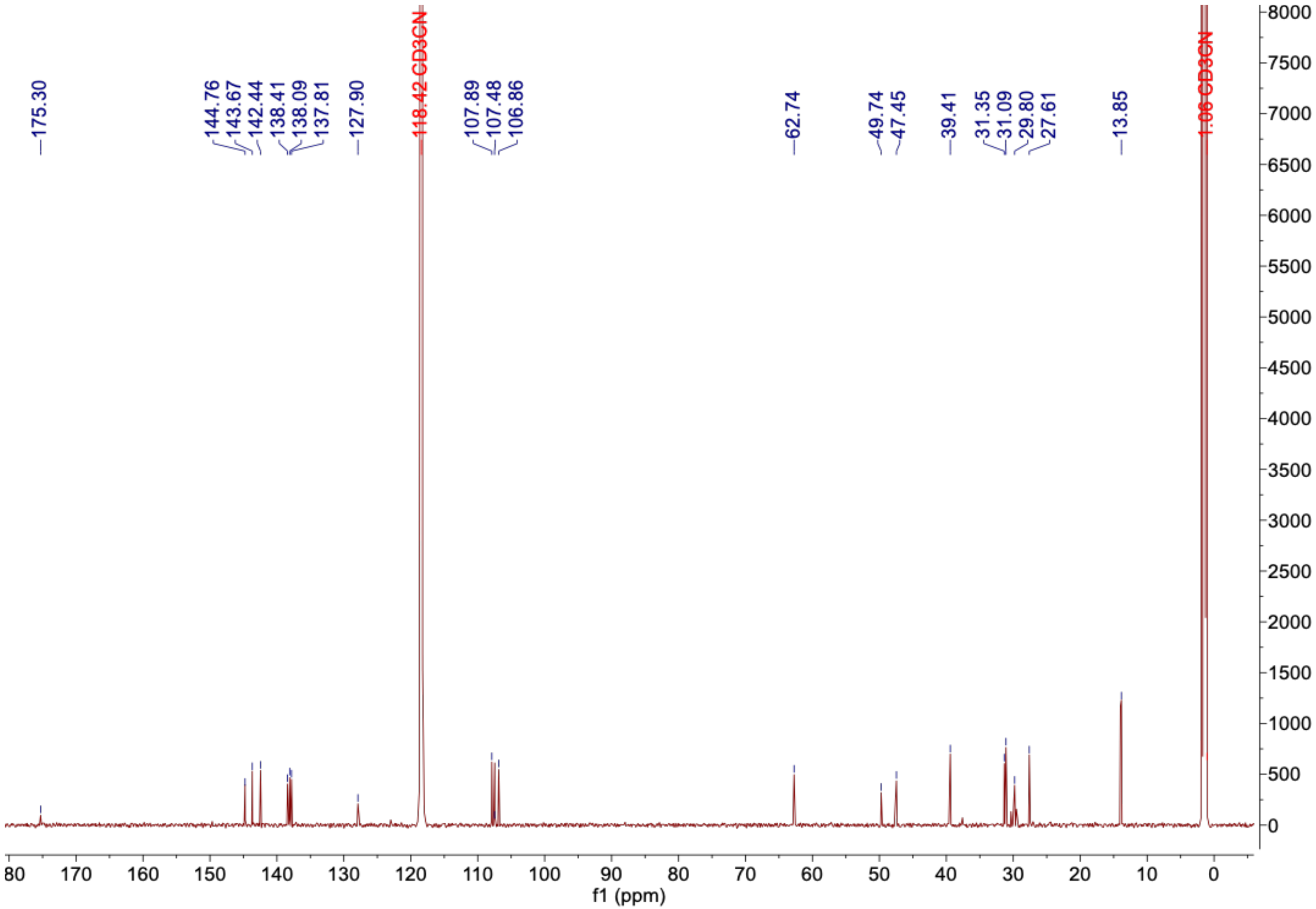

Figure S55: ^{13}C-NMR (CD_3CN) of Compound 39.

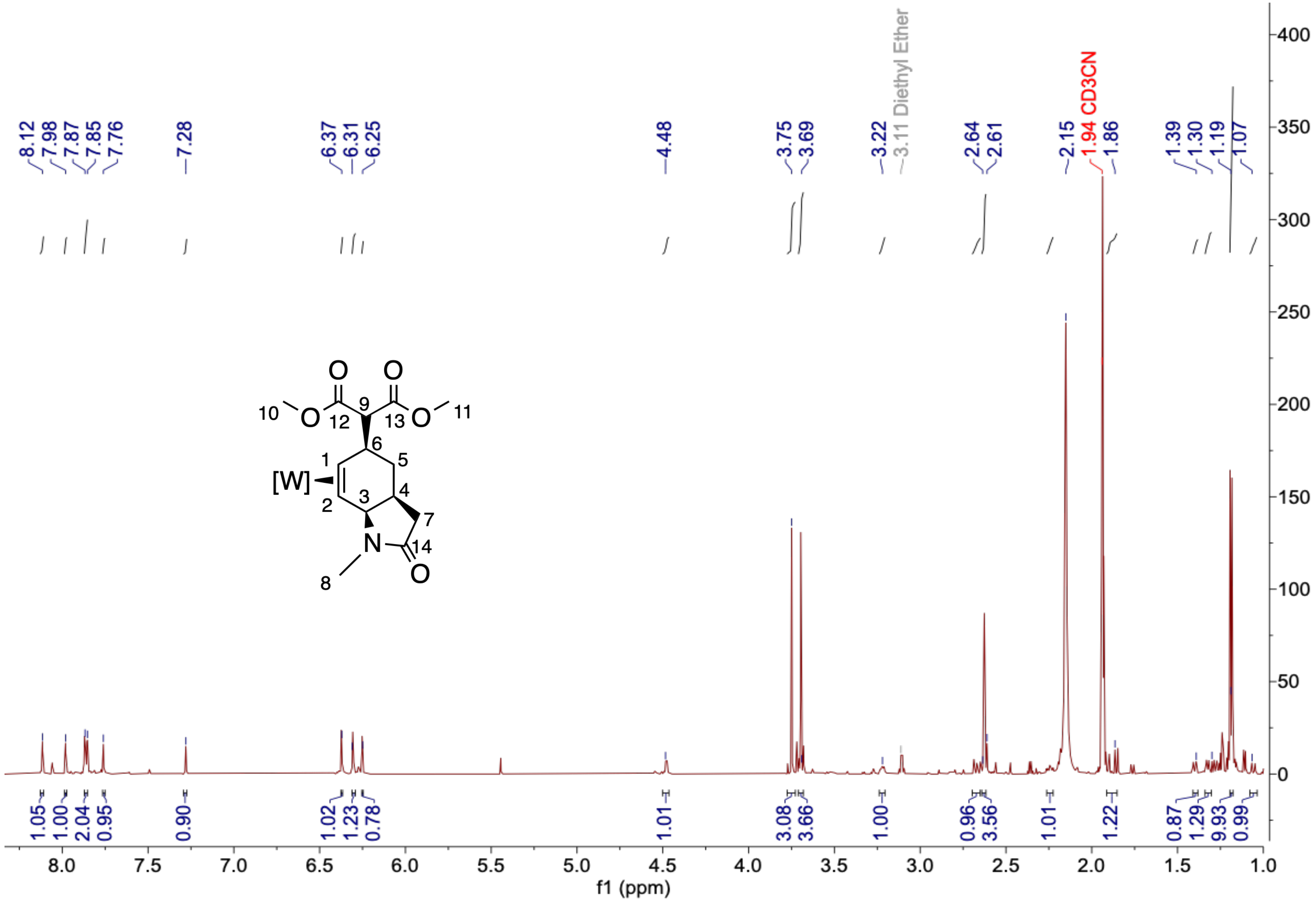

Figure S56: ^{1}H-NMR (CD_3CN) of Compound 40.

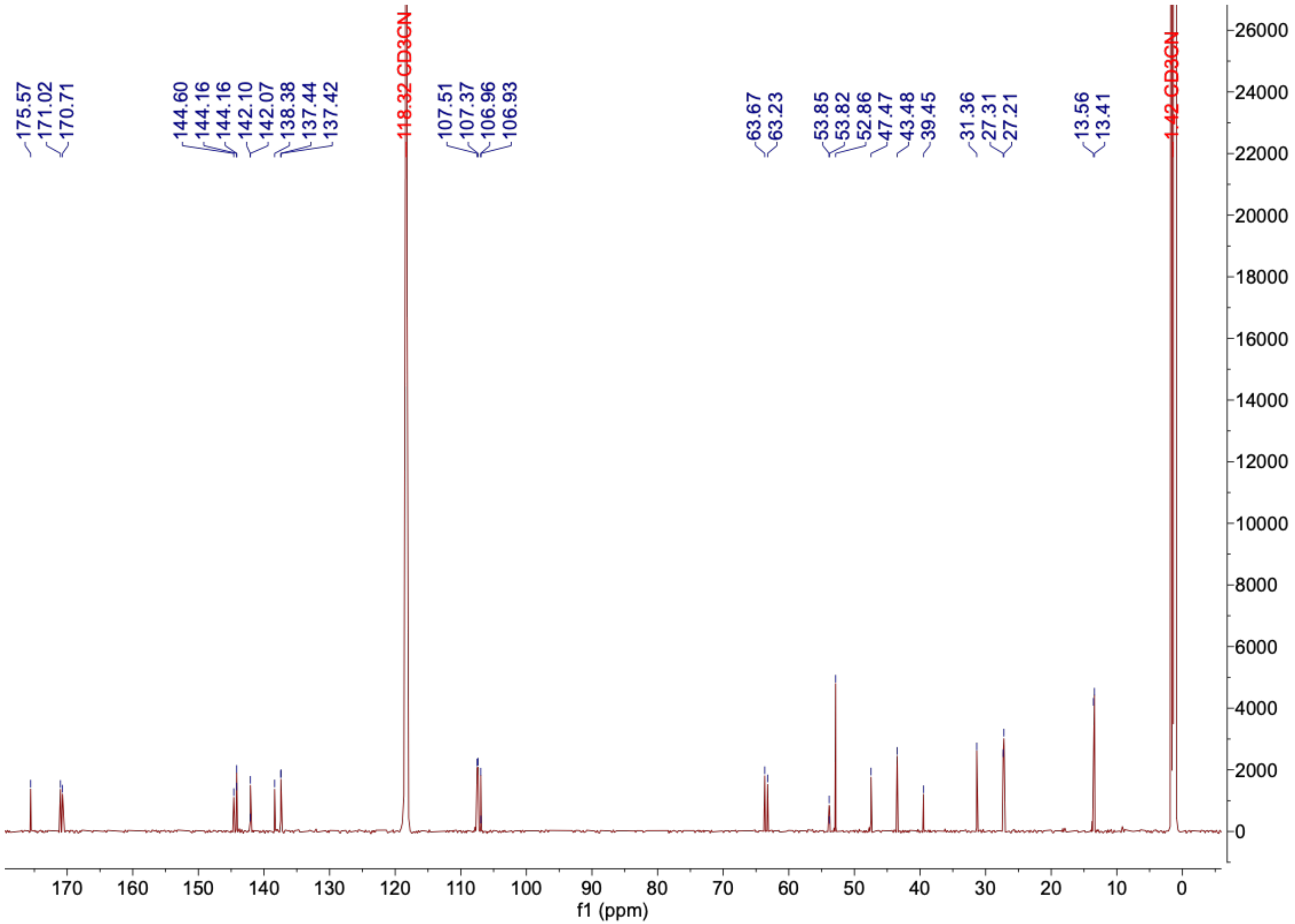

Figure S57: ^{13}C-NMR (CD_3CN) of Compound 40.

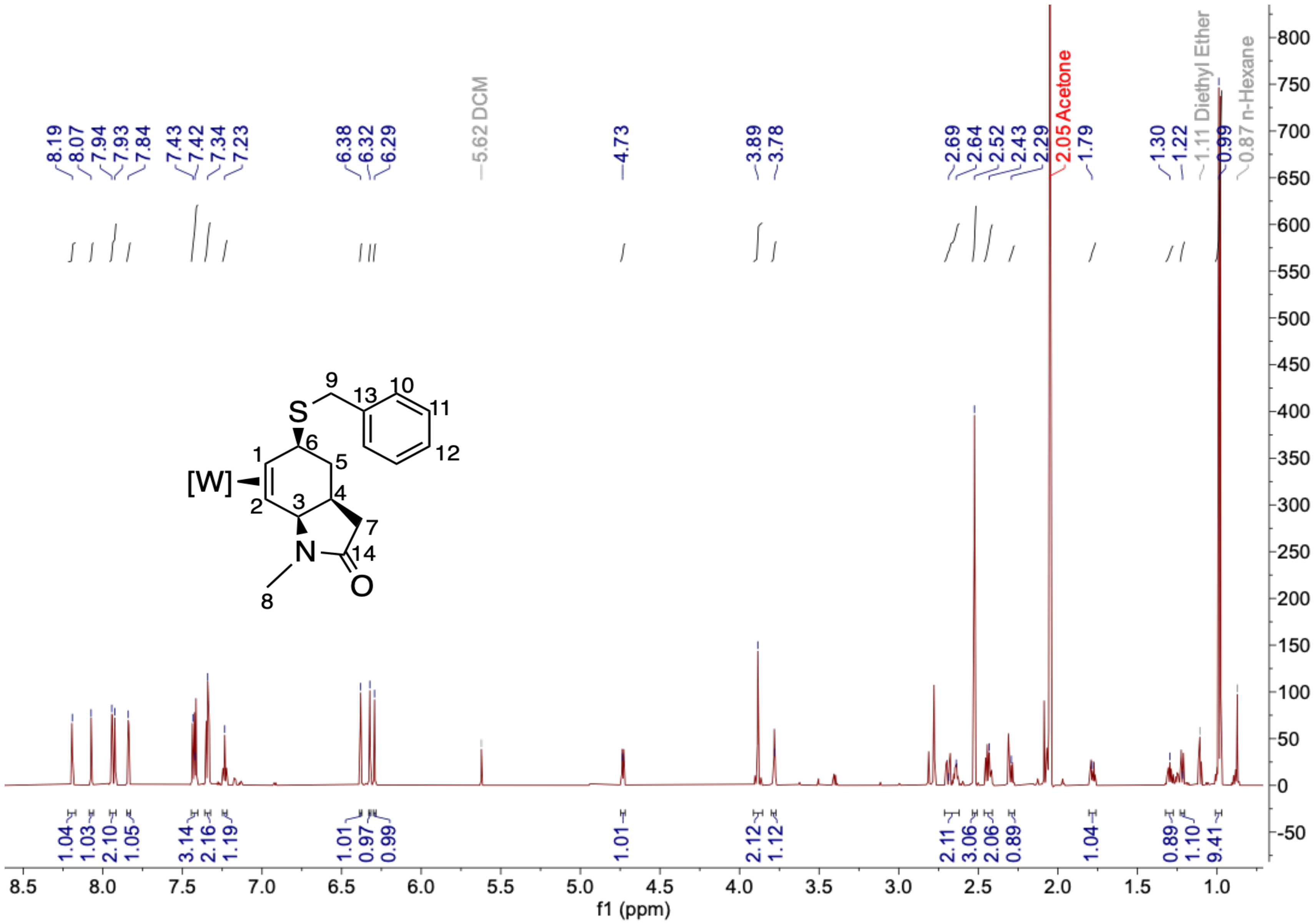

Figure S58: ^{1}H-NMR ($(CD_3)_2CO$) of Compound 41.

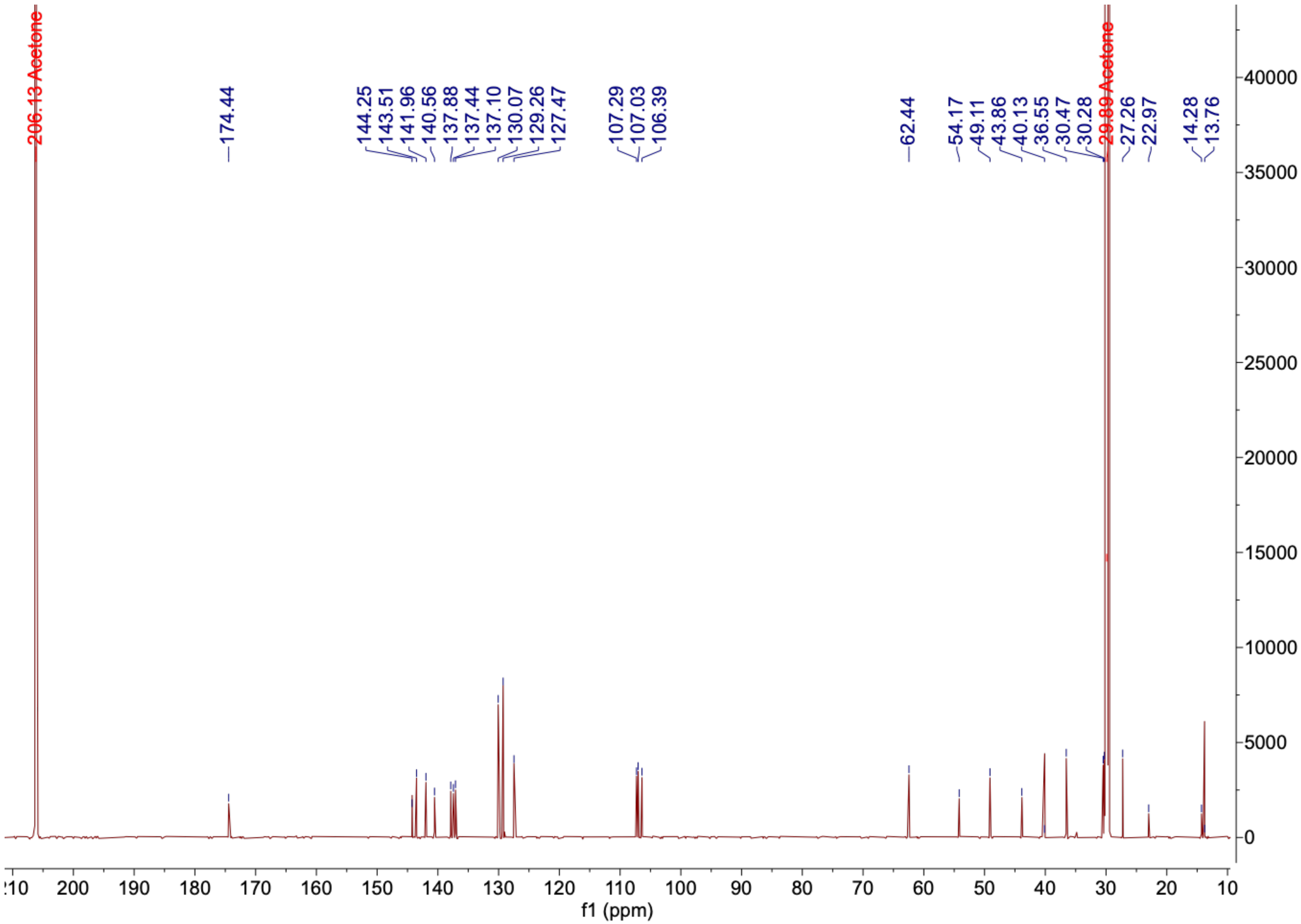

Figure S59: ^{13}C-NMR ($(CD_3)_2CO$) of Compound 41.

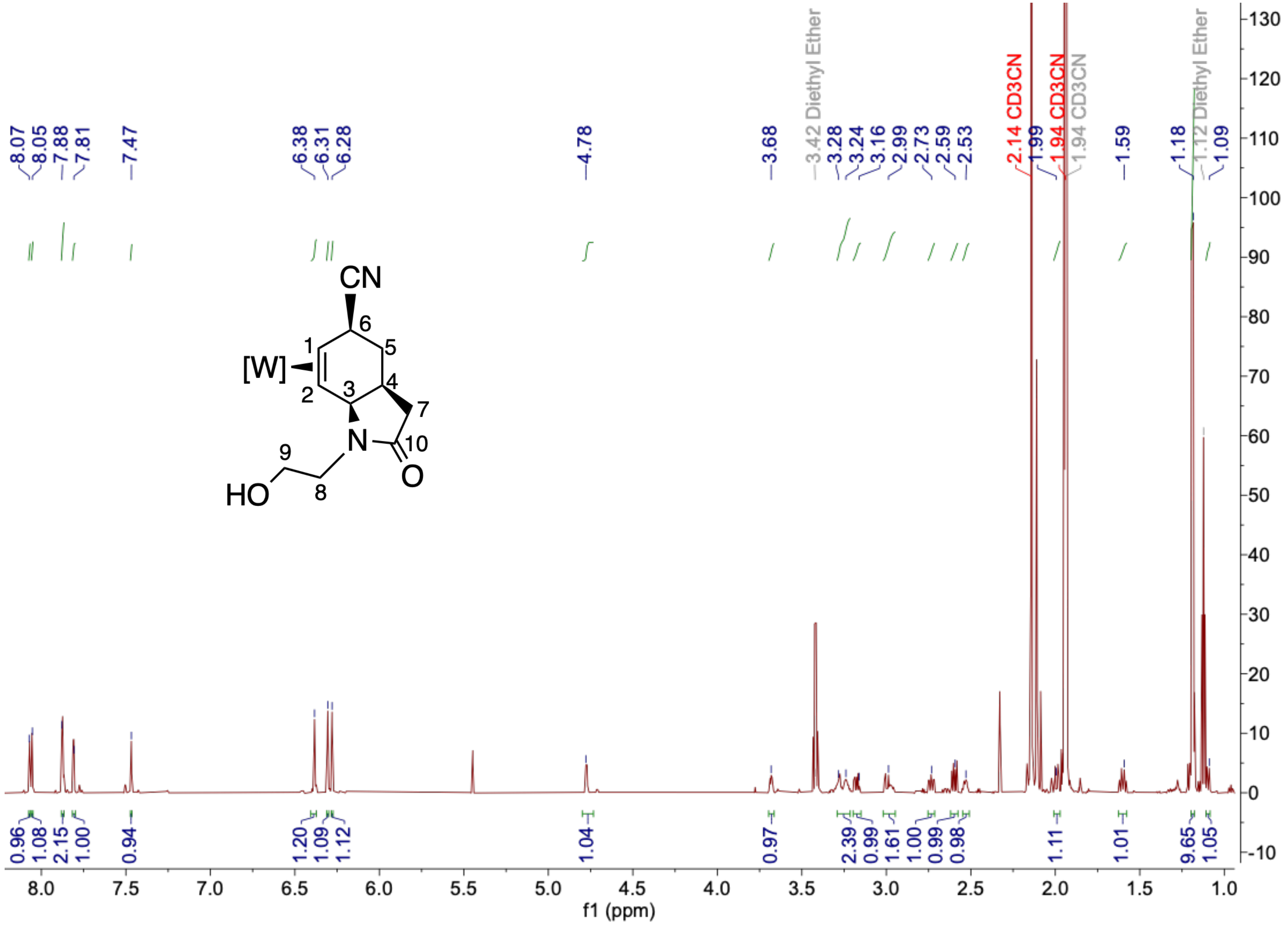

Figure S60: ^{1}H-NMR (CD_3CN) of Compound 42.

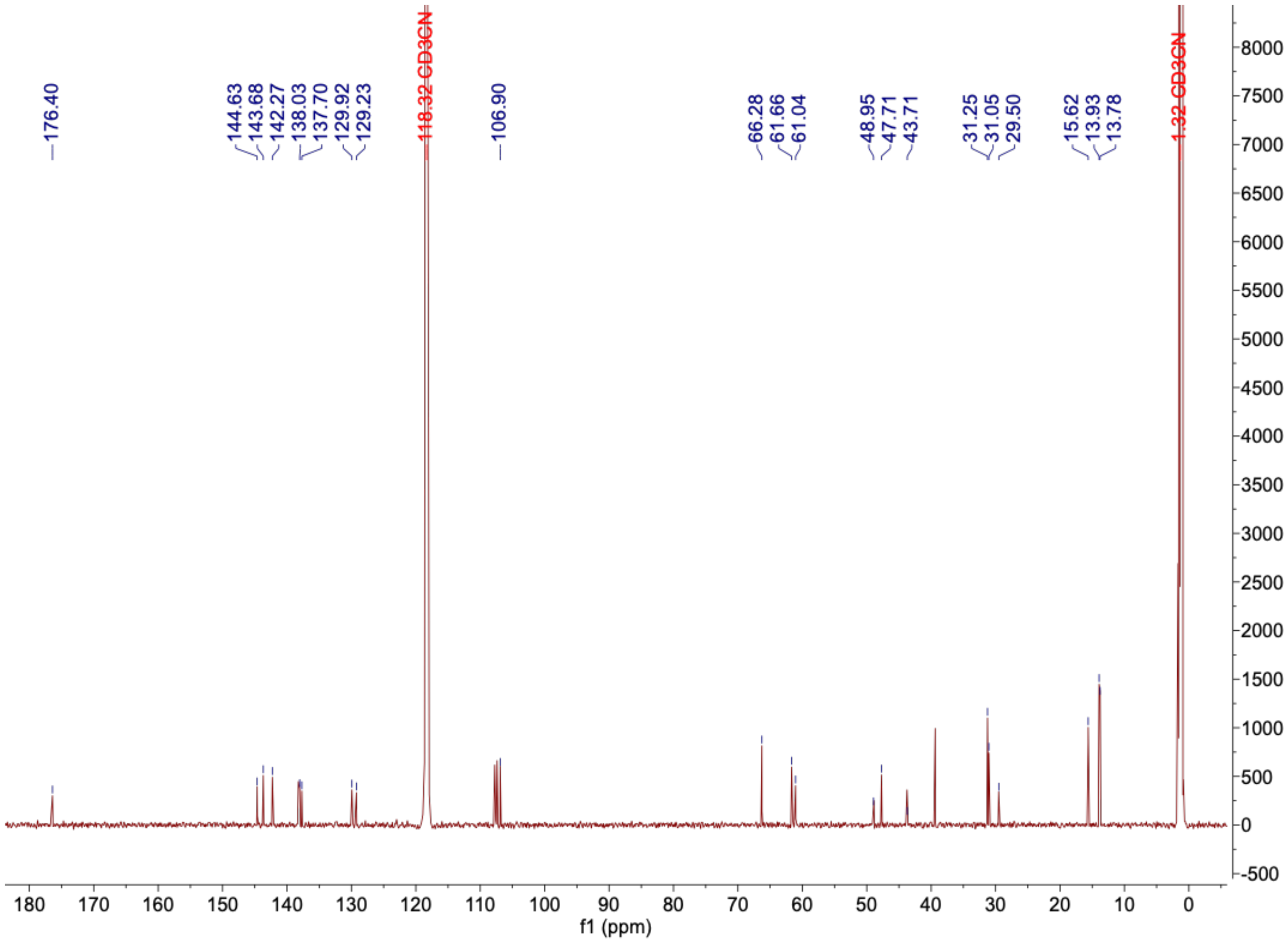

Figure S61: ^{13}C-NMR (CD_3CN) of Compound 42.

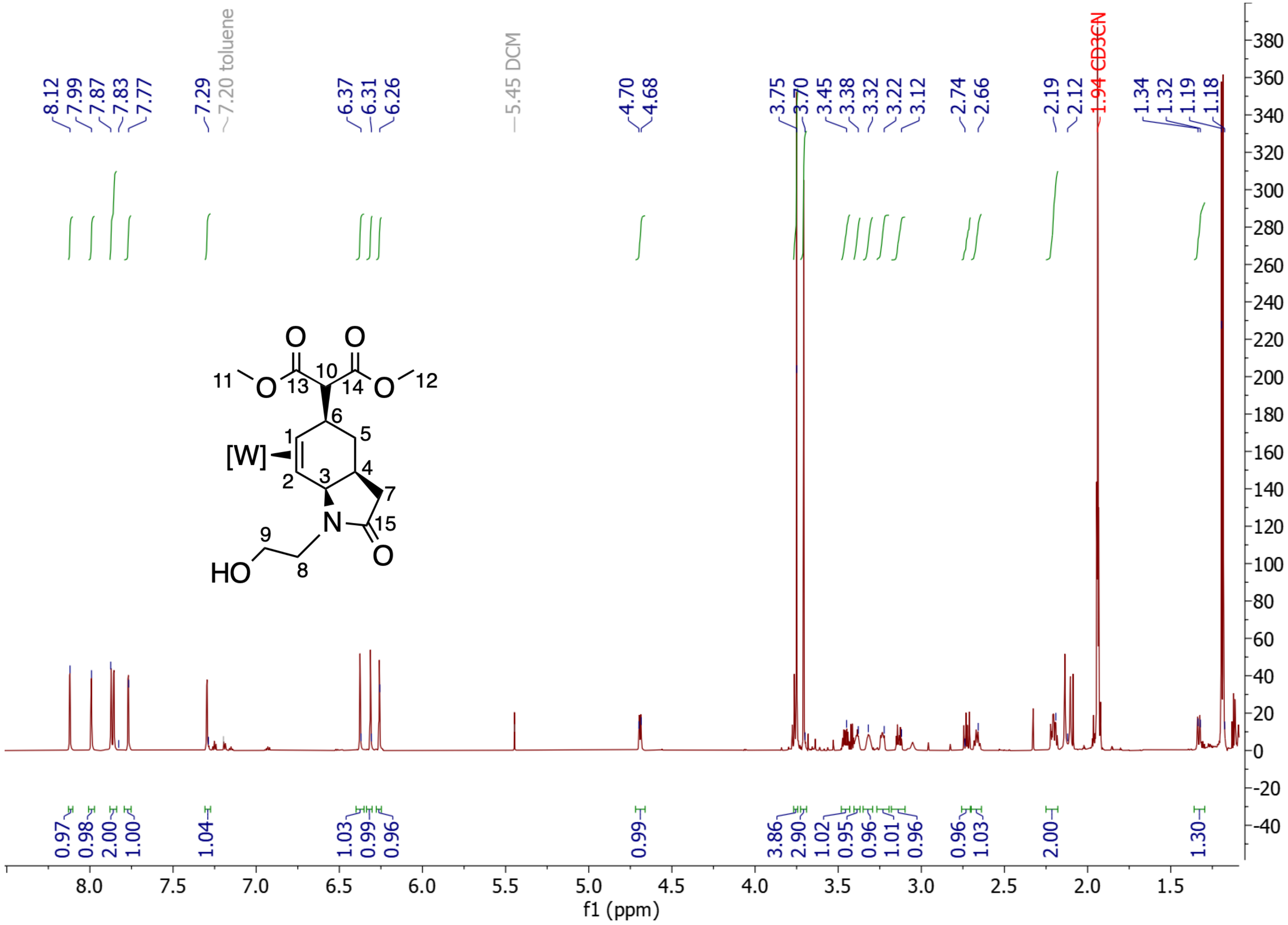

Figure S62: ^{1}H-NMR (CD_3CN) of Compound 43.

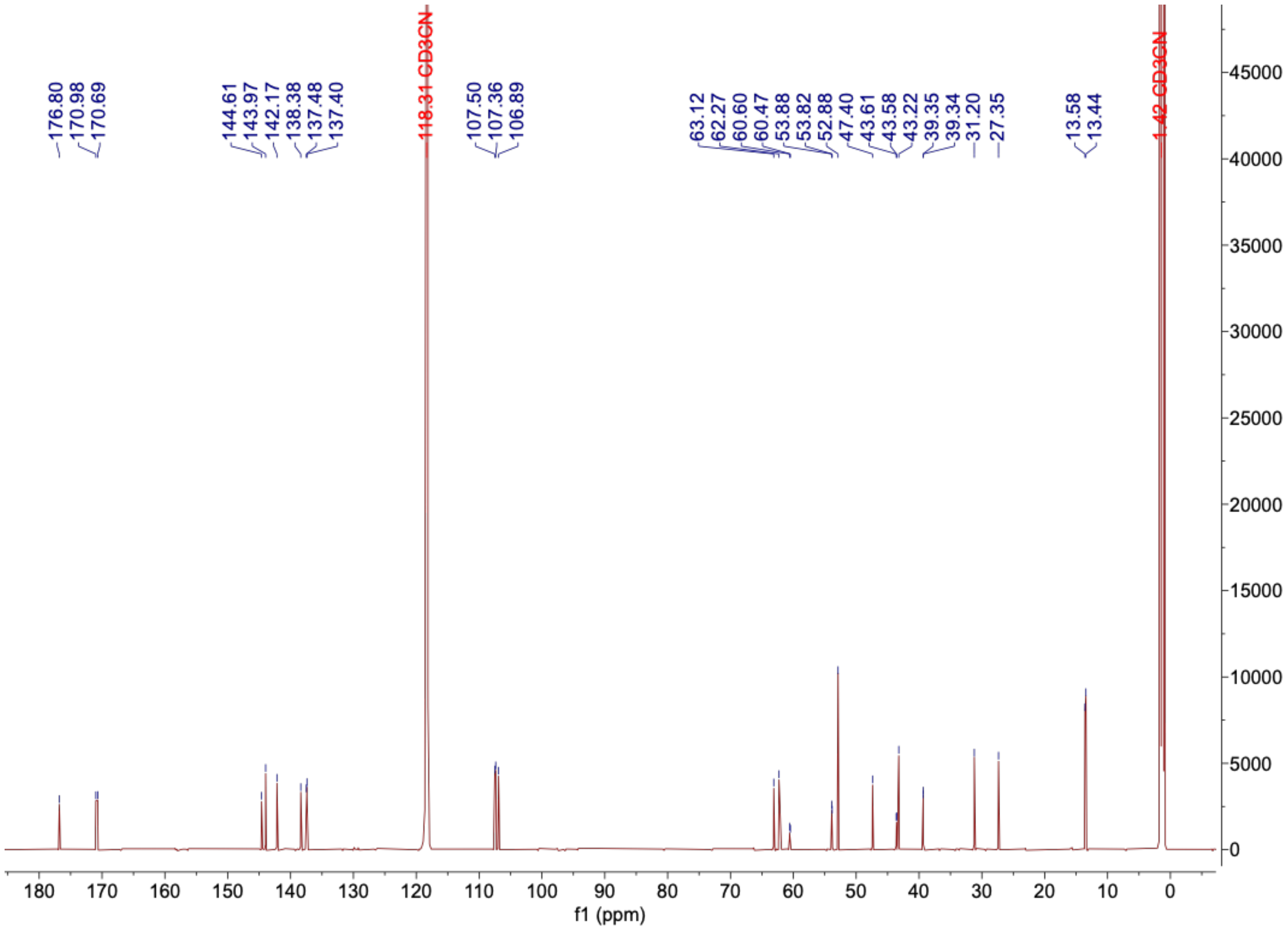

Figure S63: ^{13}C-NMR (CD_3CN) of Compound 43.

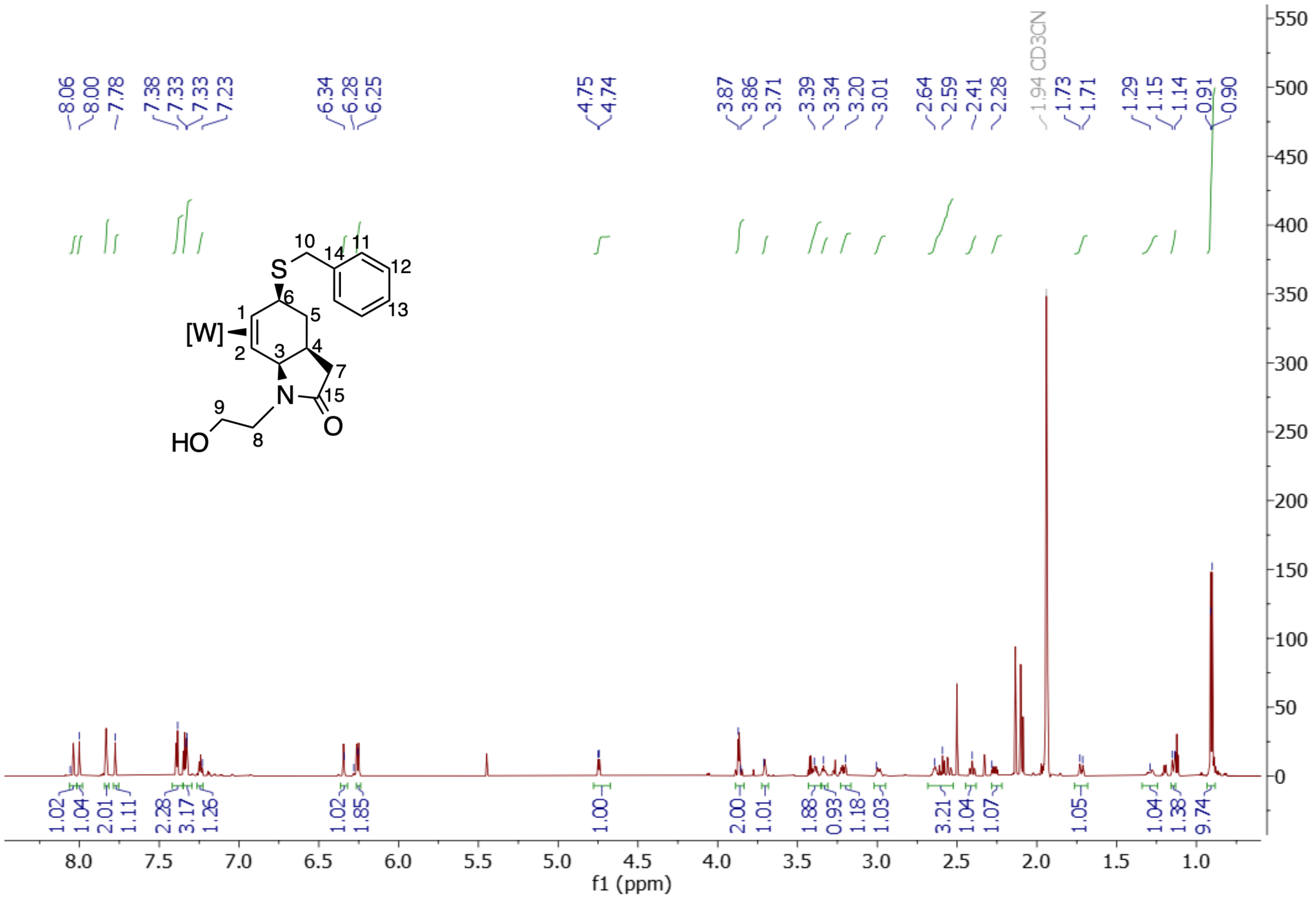

Figure S64: ^{1}H-NMR (CD_3CN) of Compound 44.

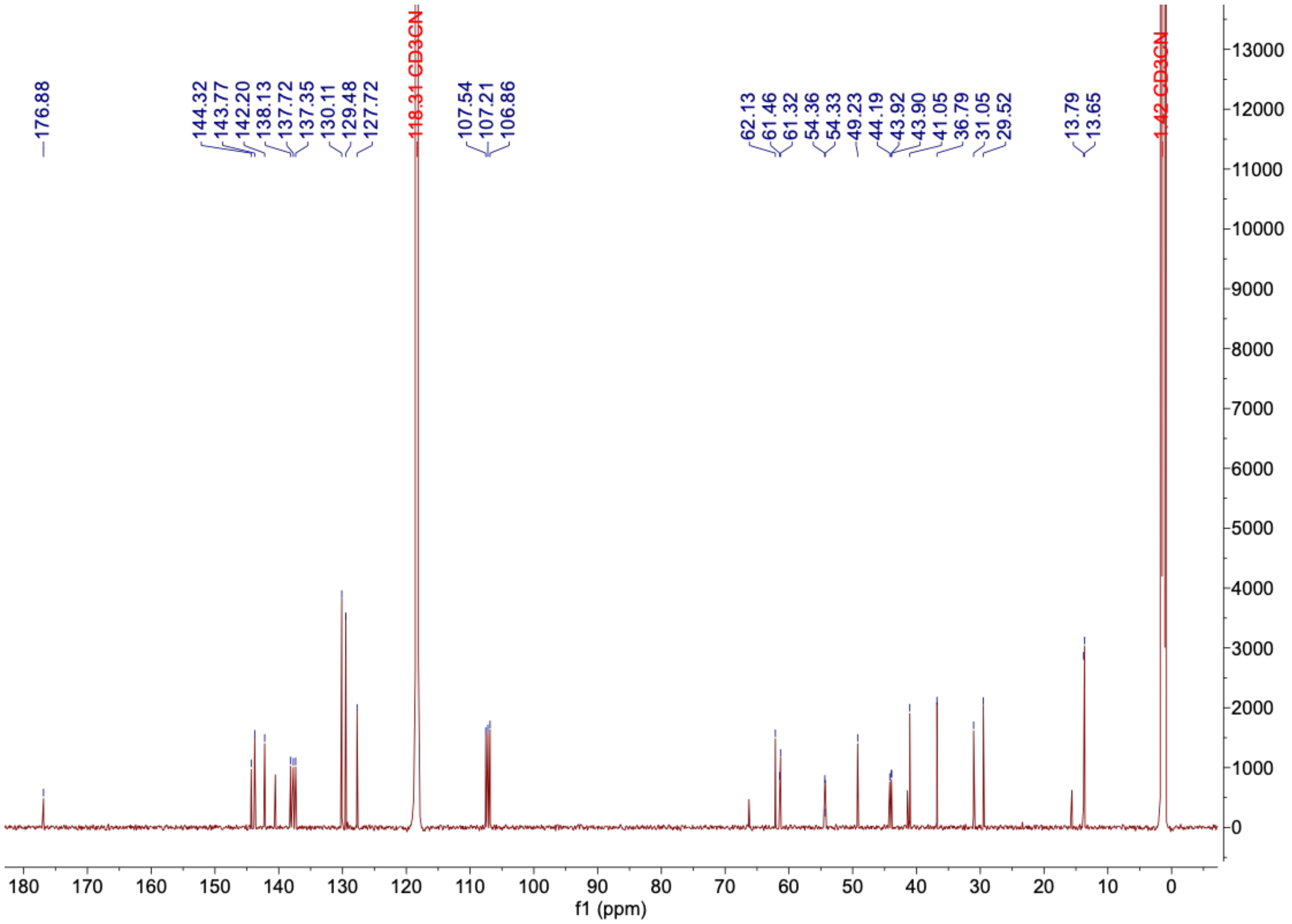

Figure S65: ^{13}C-NMR (CD_3CN) of Compound 44.

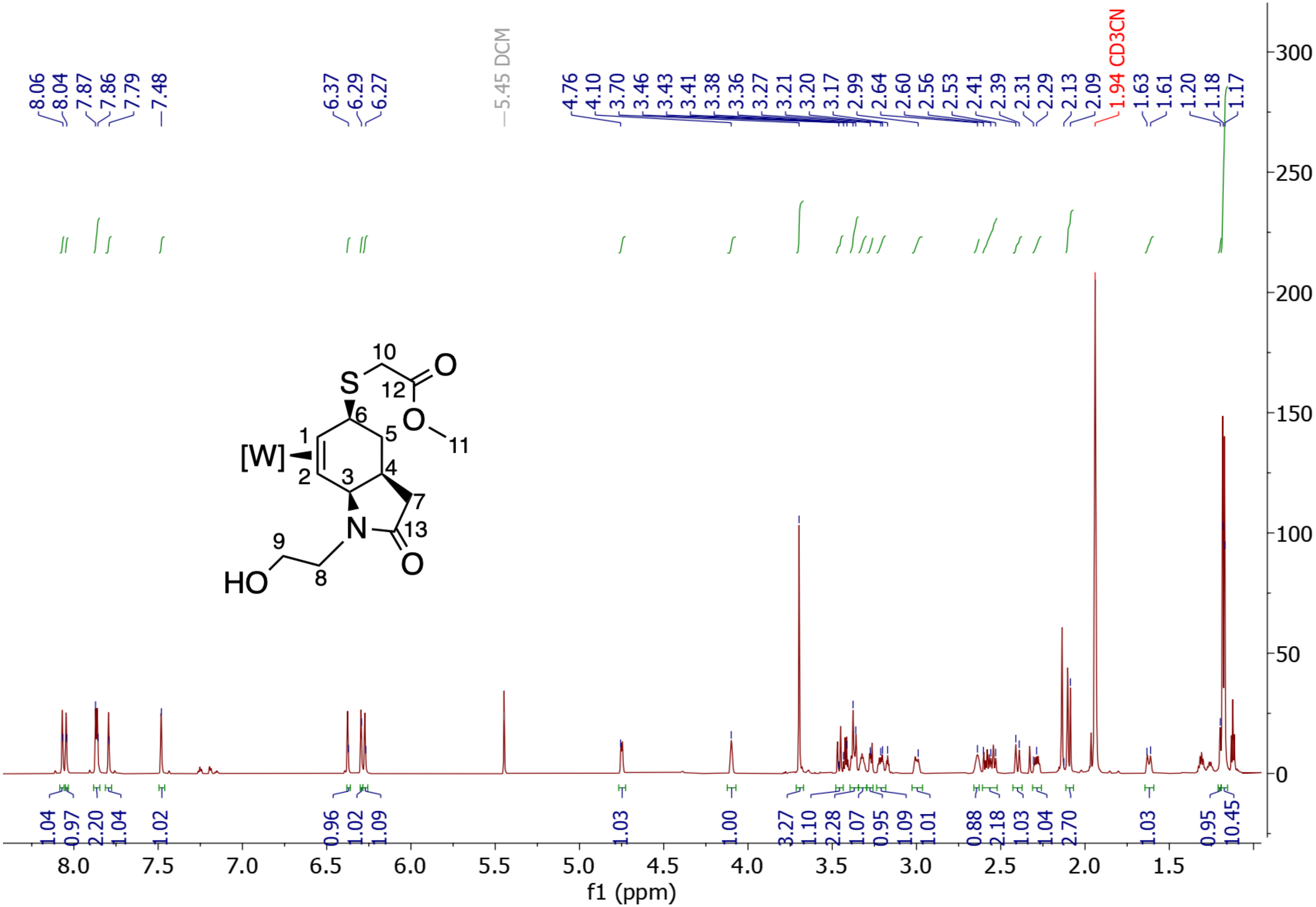

Figure S66: ^{1}H-NMR (CD_3CN) of Compound 45.

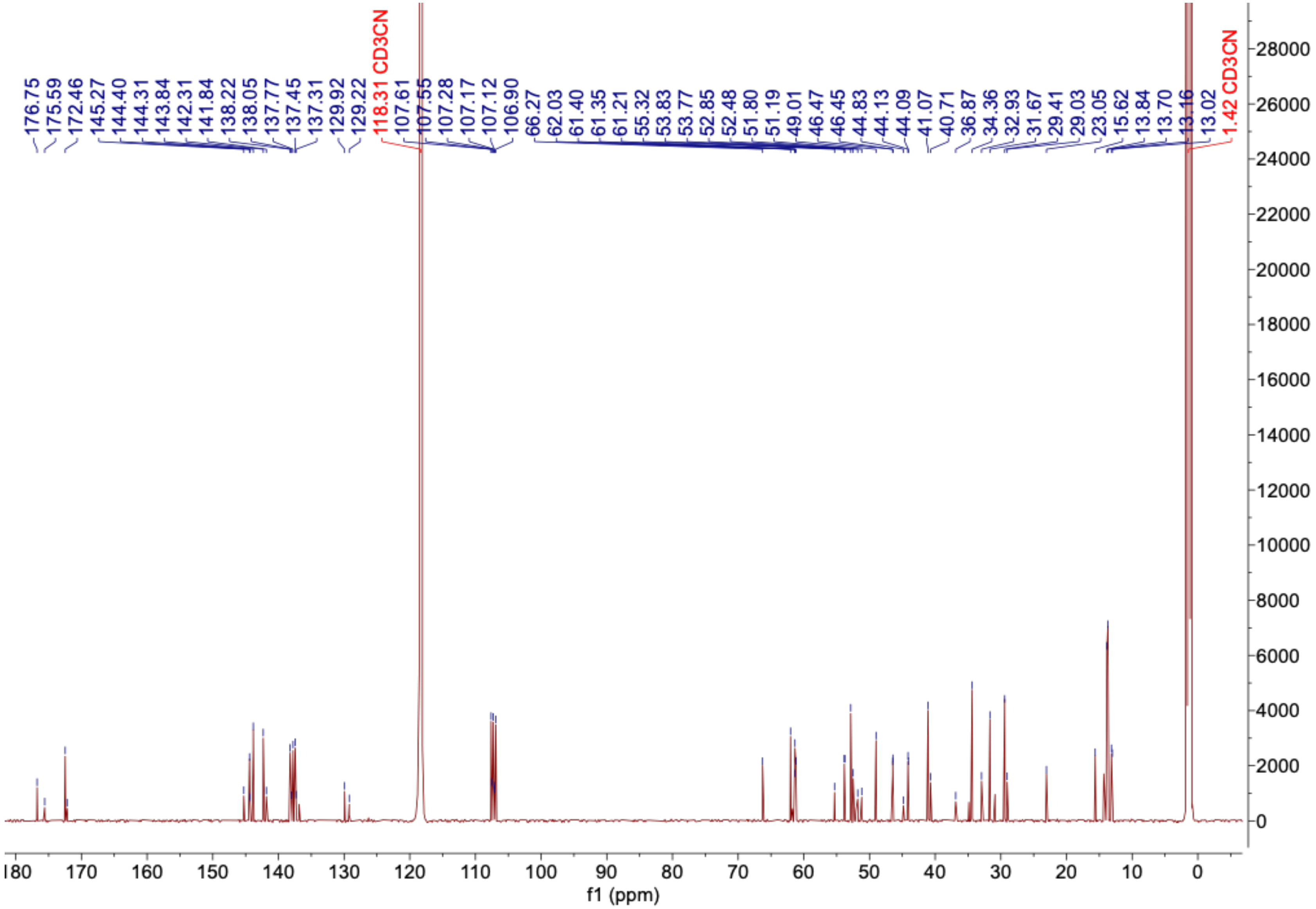

Figure S67: ^{13}C-NMR (CD_3CN) of Compound 45.
While in solution the other isomer was also generated.

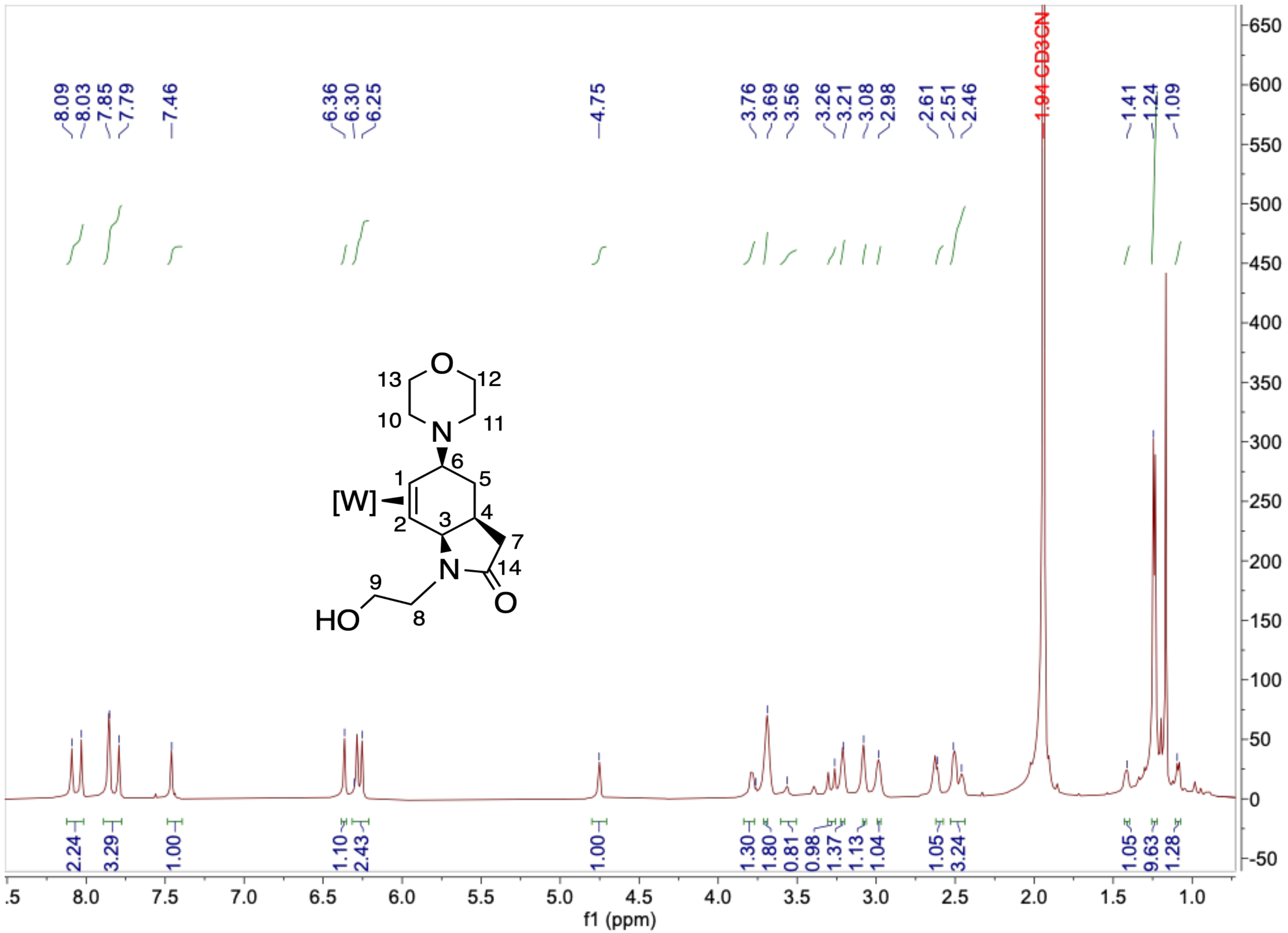

Figure S68: ¹H-NMR (CD_3CN) of Compound 46.

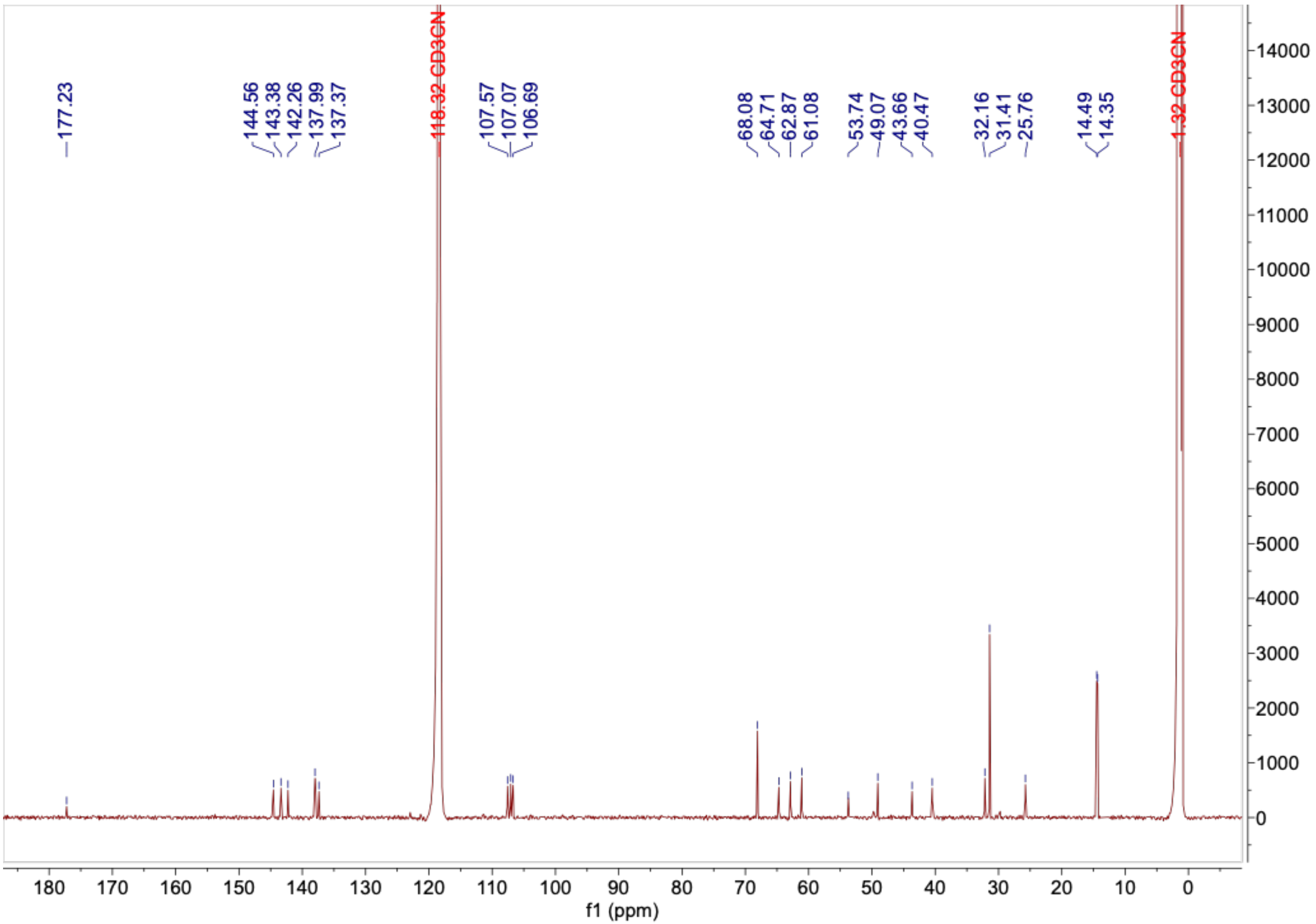

Figure S69: ^{13}C-NMR (CD_3CN) of Compound 46.

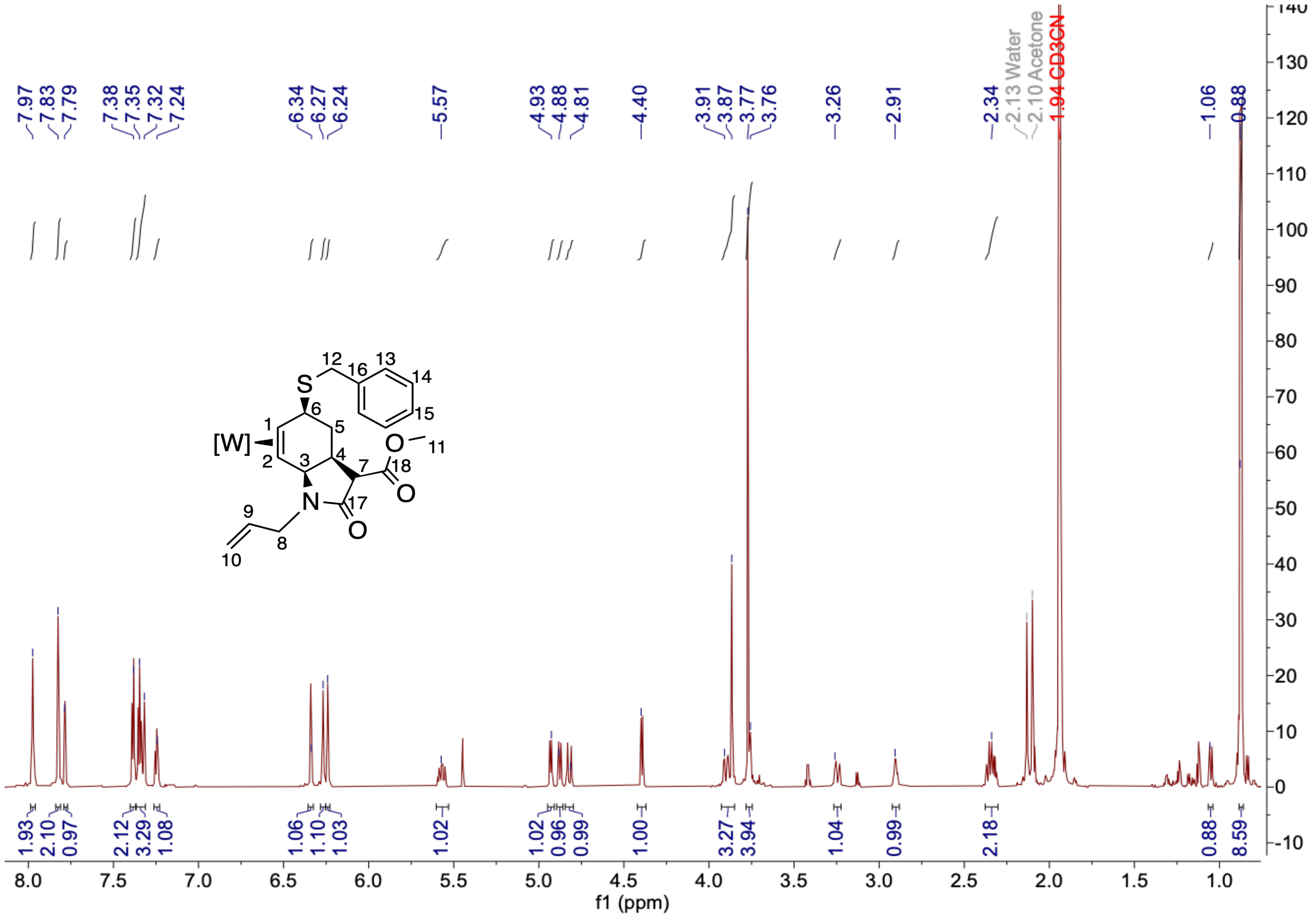

Figure S70: ^{1}H-NMR (CD_3CN) of Compound 47.

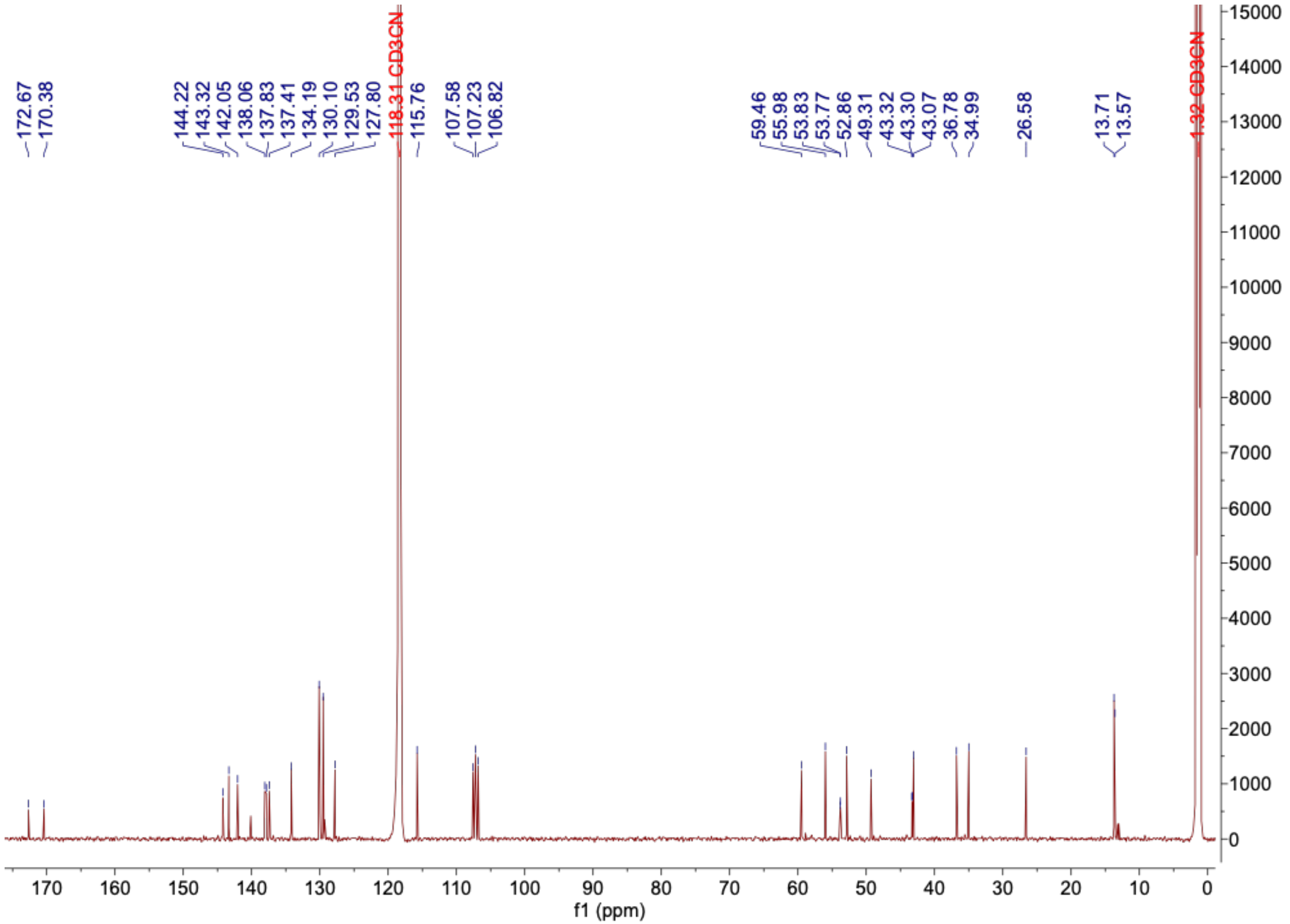

Figure S71: ^{13}C-NMR (CD_3CN) of Compound 47.

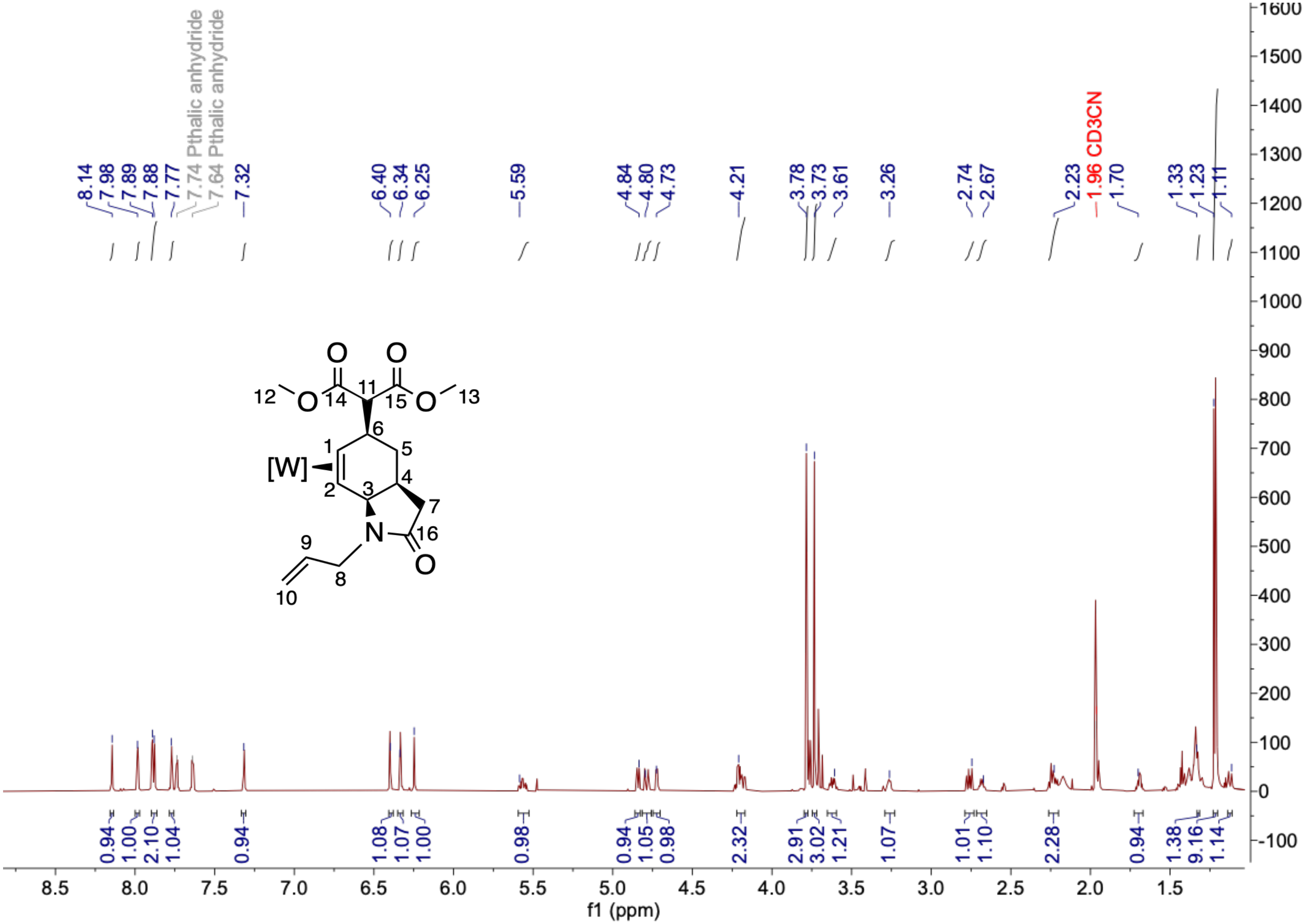

Figure S72: ^{1}H-NMR (CD_3CN) of Compound 48.

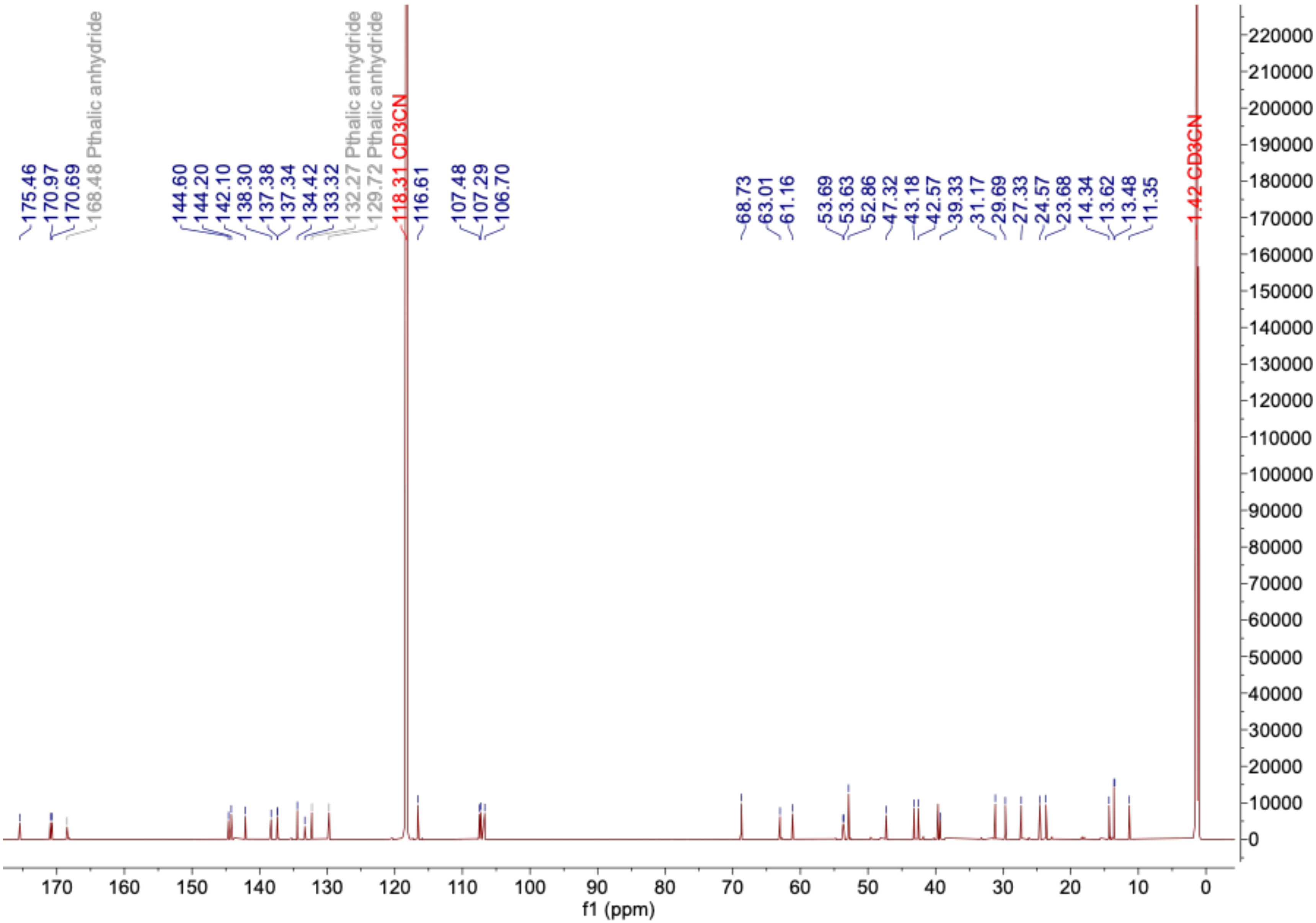

Figure S73: ^{13}C-NMR (CD_3CN) of Compound 48.

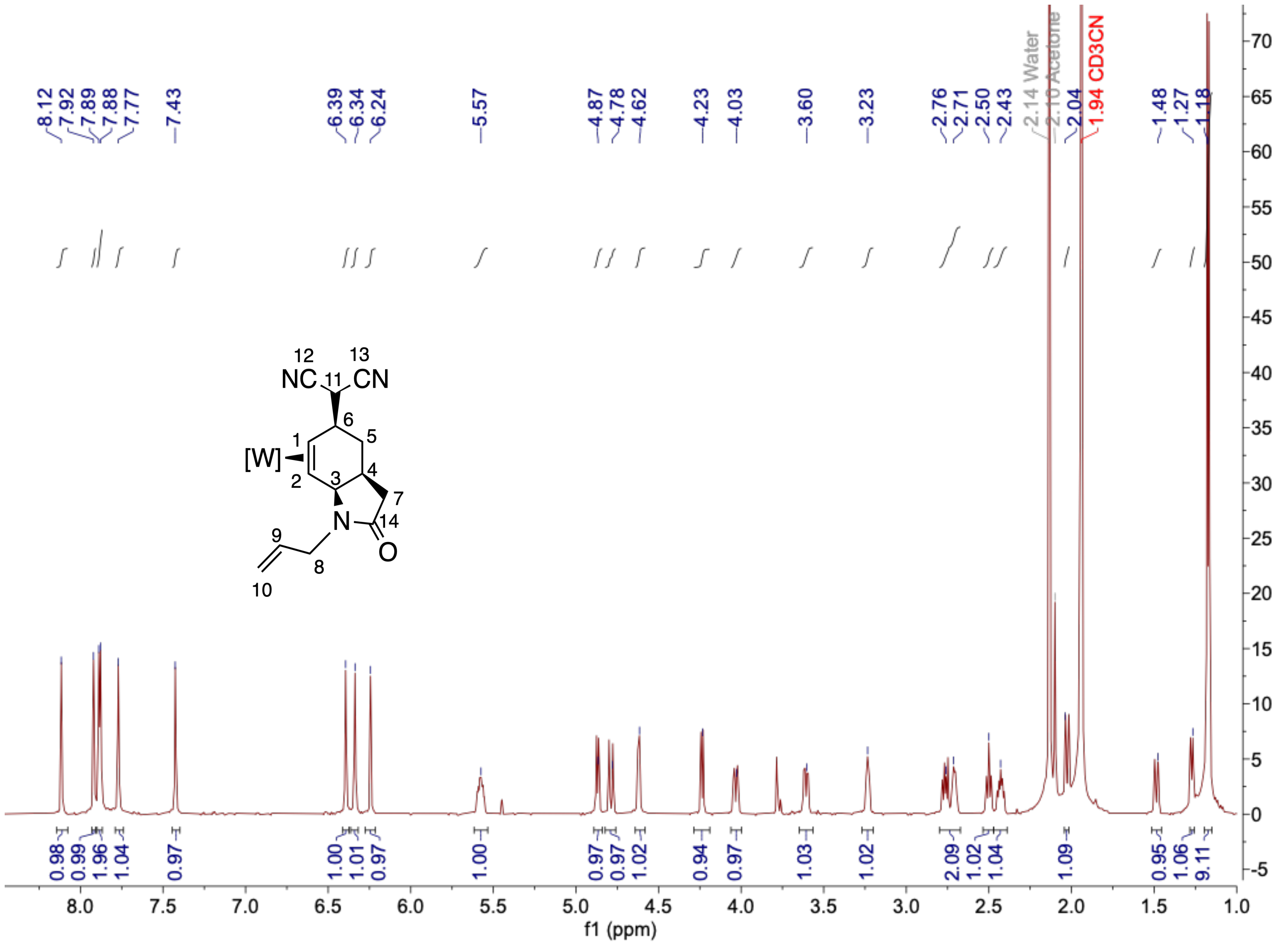

Figure S74: ^{1}H-NMR (CD_3CN) of Compound 49.

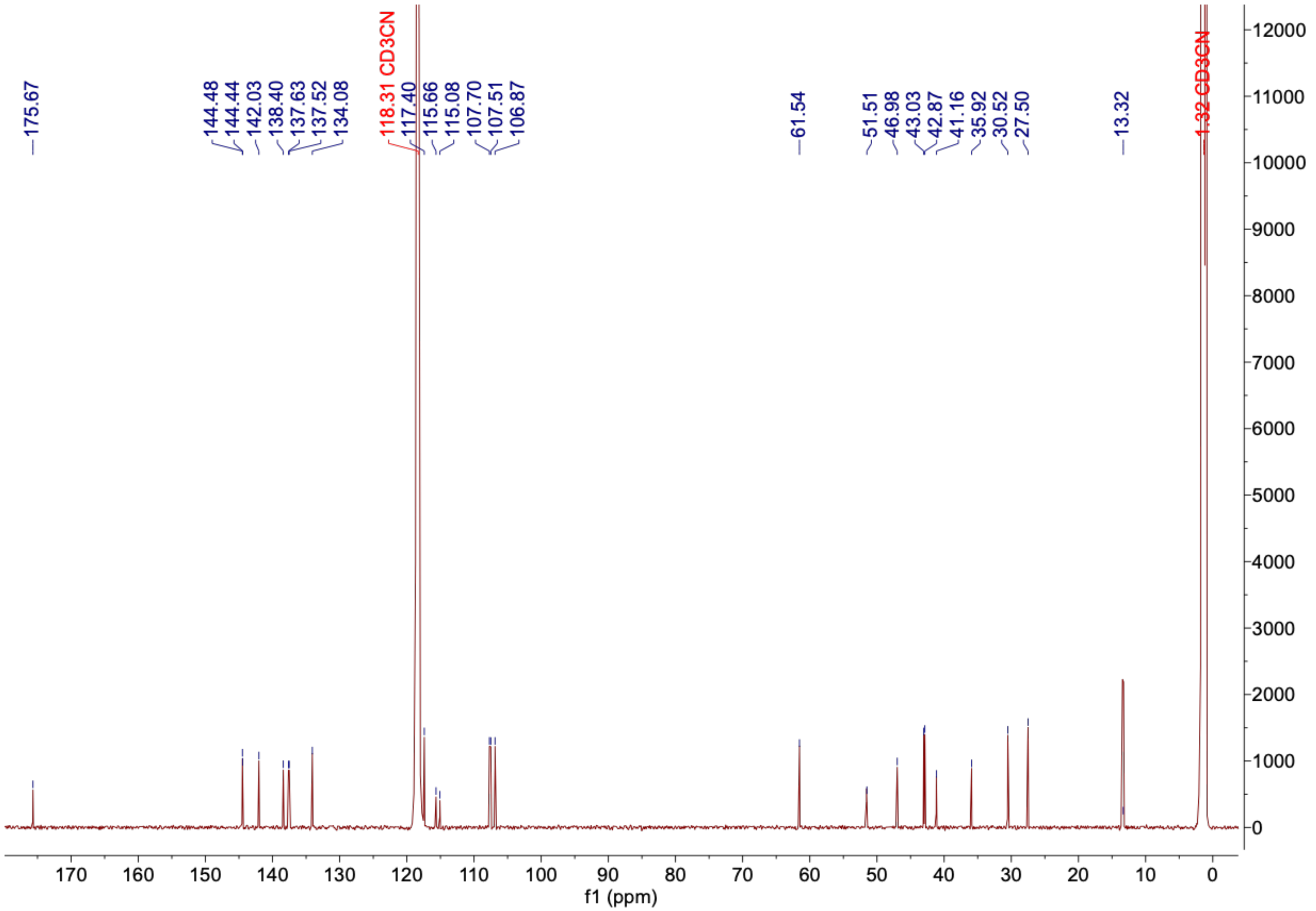

Figure S75: ^{13}C-NMR (CD_3CN) of Compound 49.

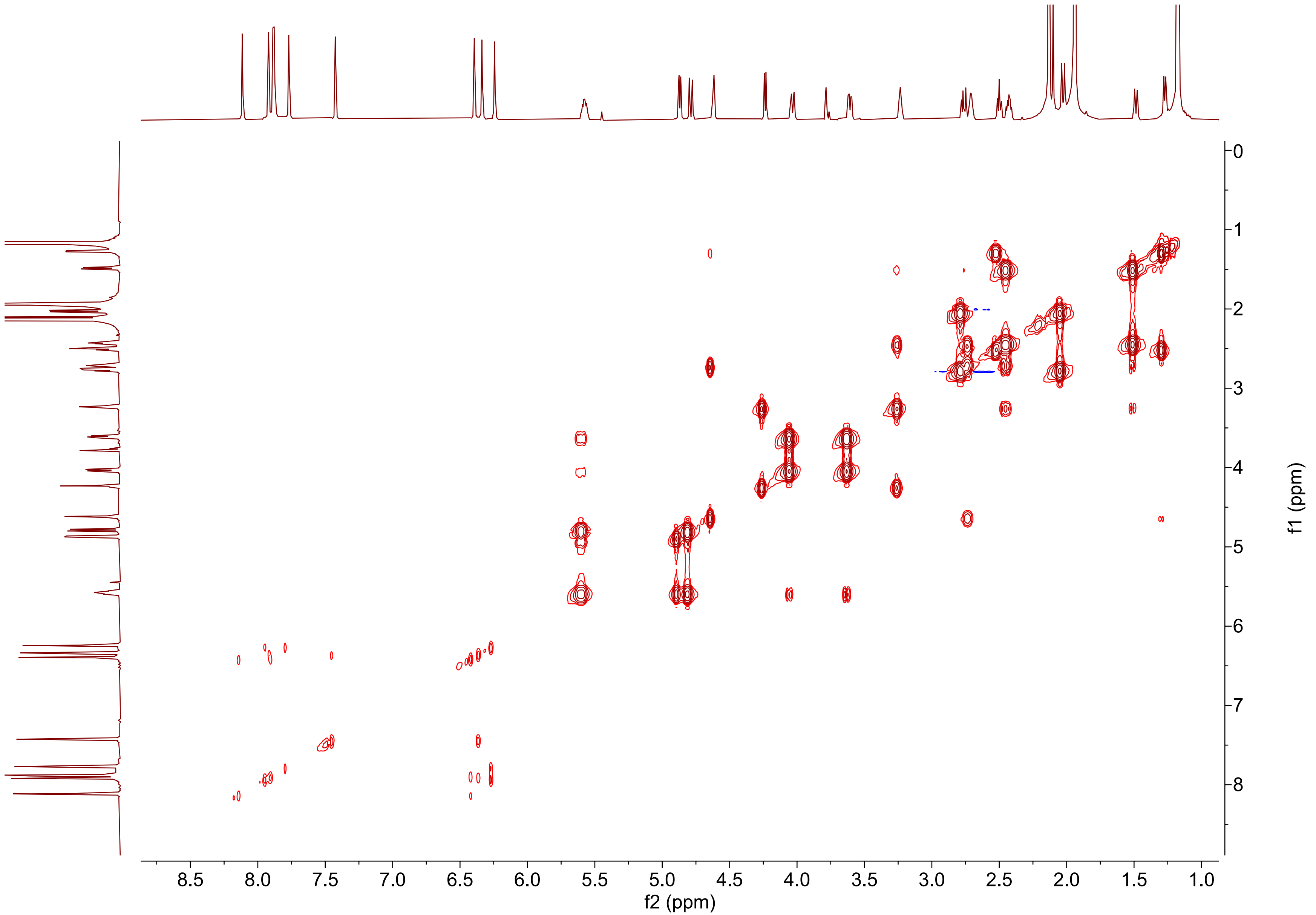

Figure S76: ^{1}H-^{1}H COSY (CD_3CN) of Compound 49.

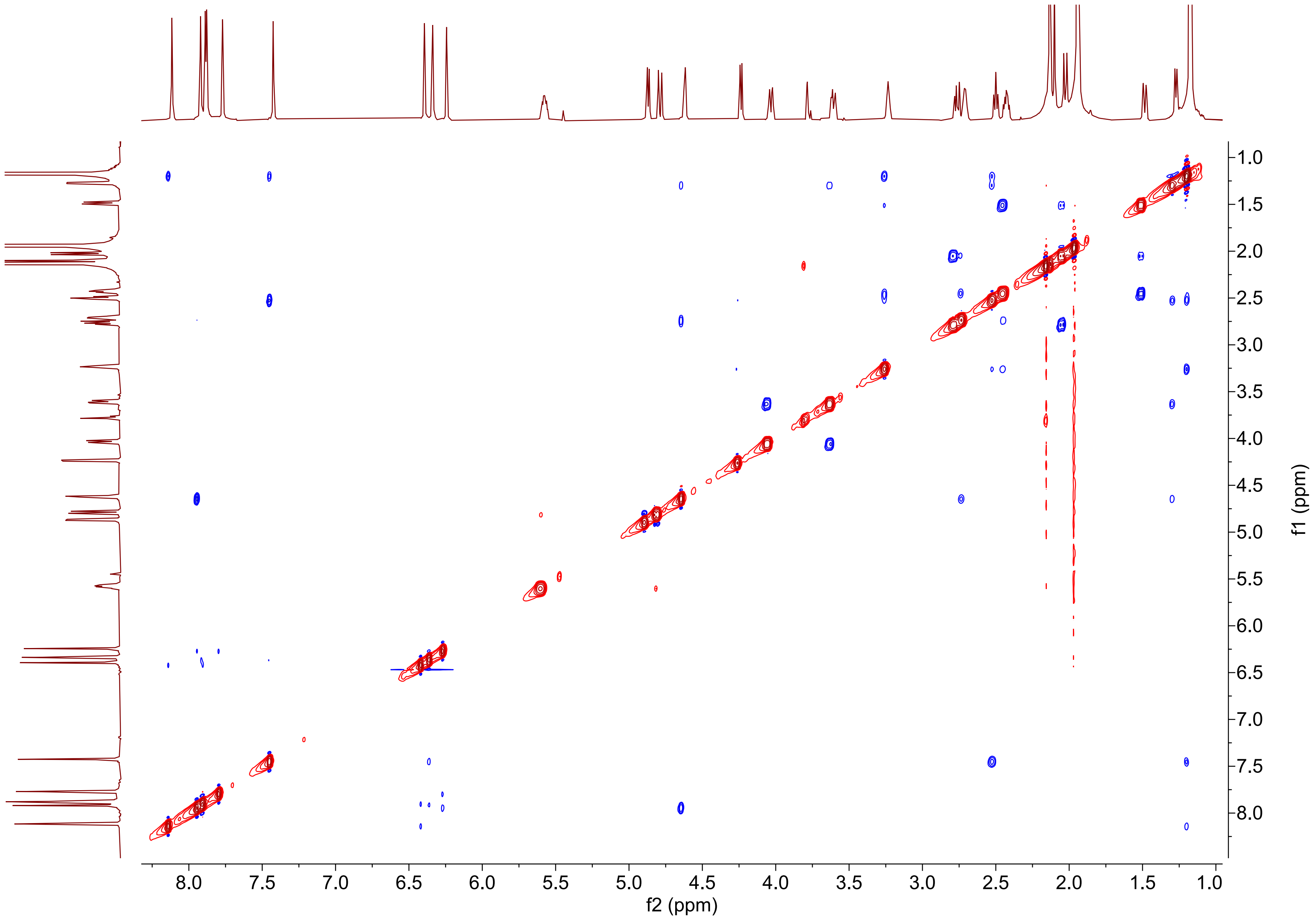

Figure S77: ^{1}H-^{1}H NOSEY (CD_3CN) of Compound 49.

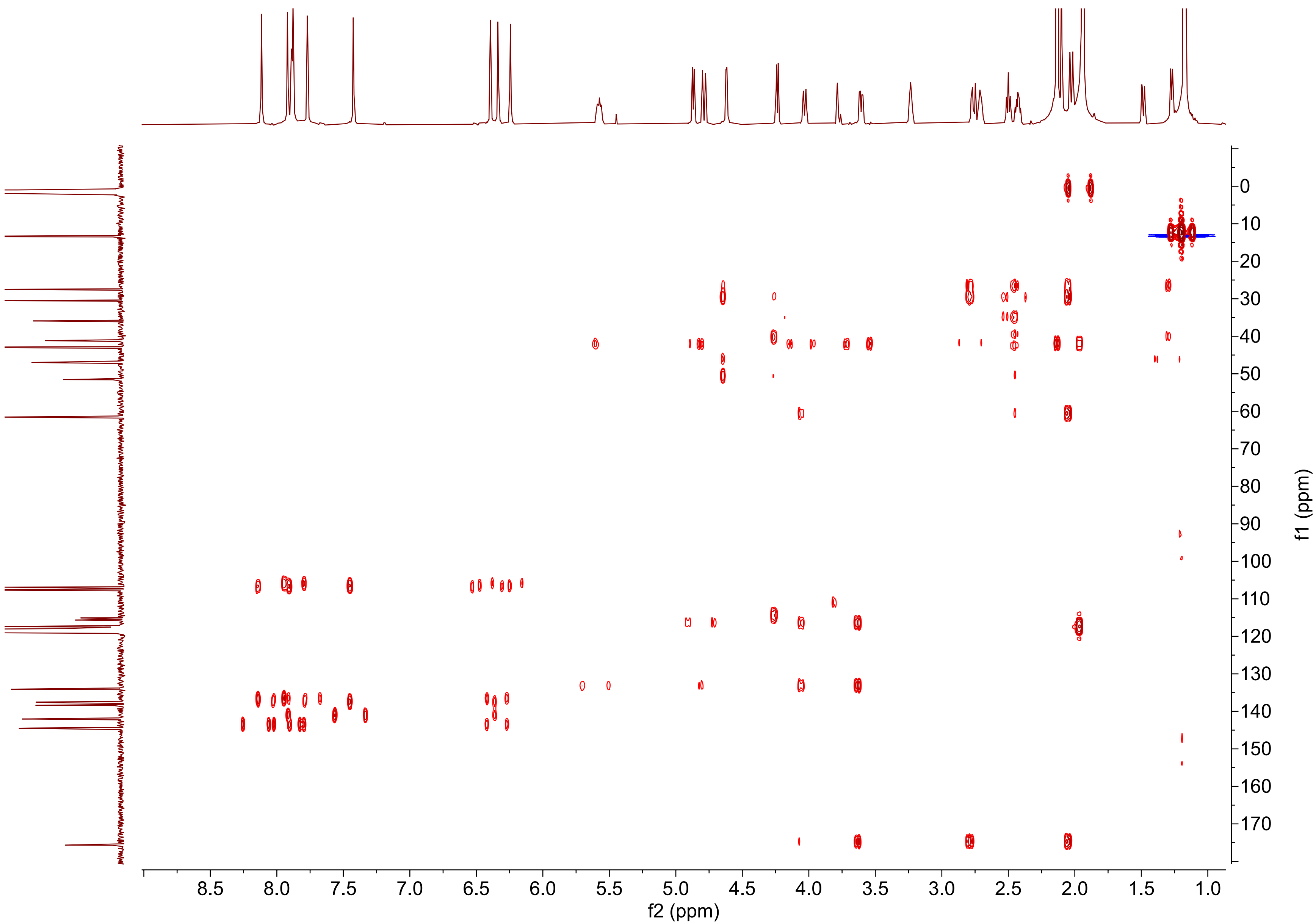

Figure S78: ^{1}H-^{13}C HMBC (CD_3CN) of Compound 49.

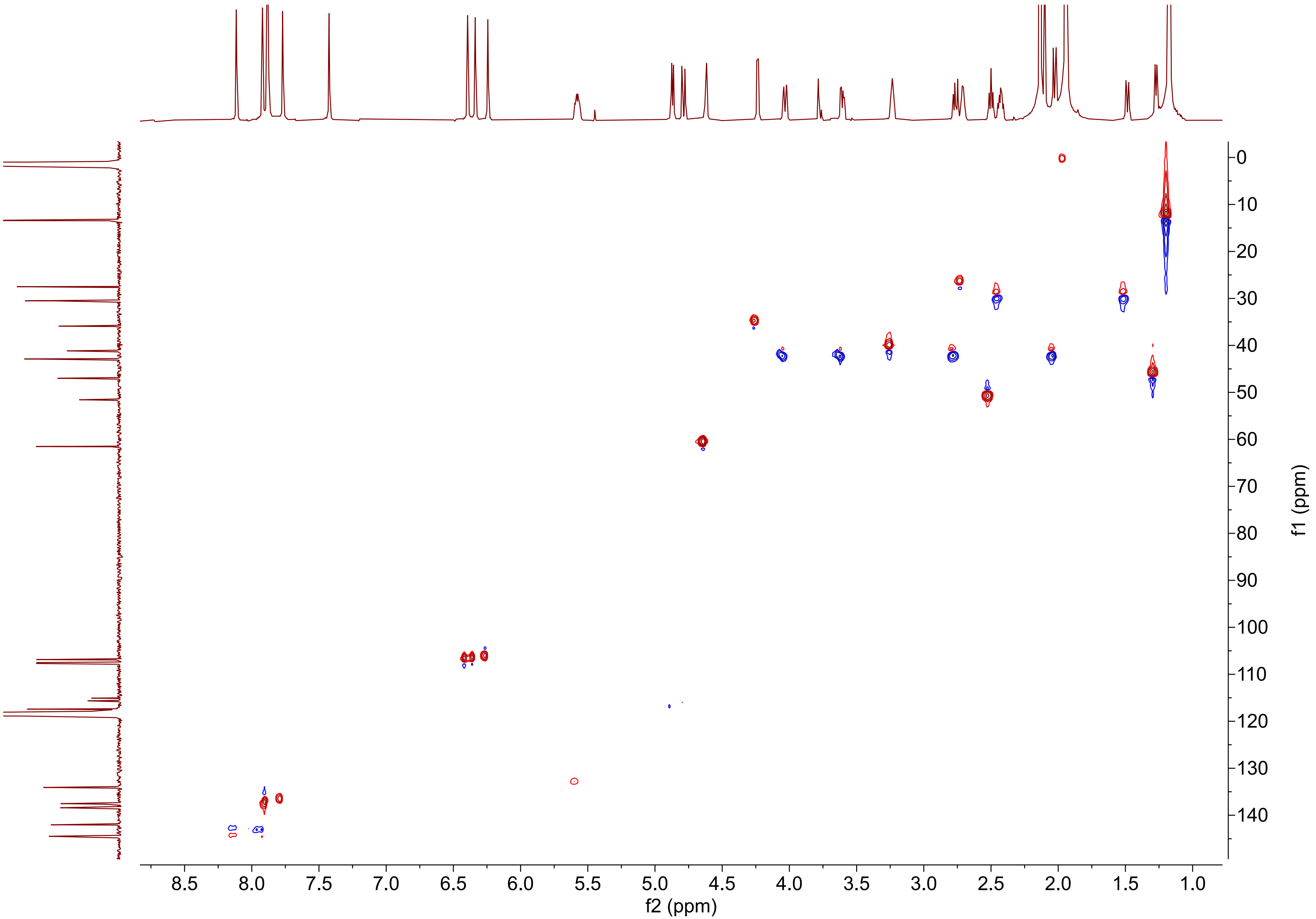

Figure S79: ^{1}H-^{13}C HSQC (CD_3CN) of Compound 49.

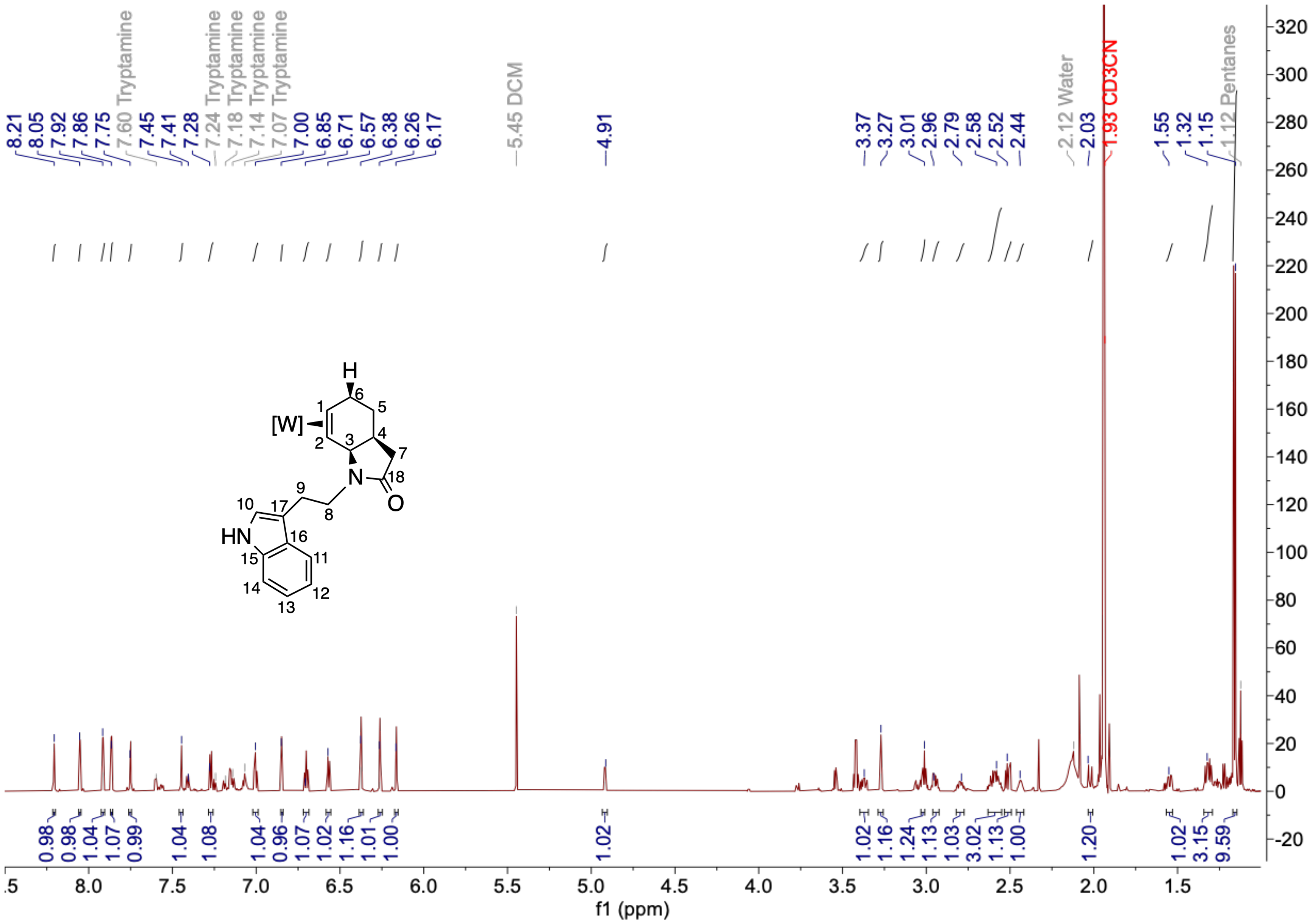

Figure S80: ^{1}H-NMR (CD_3CN) of Compound 50.

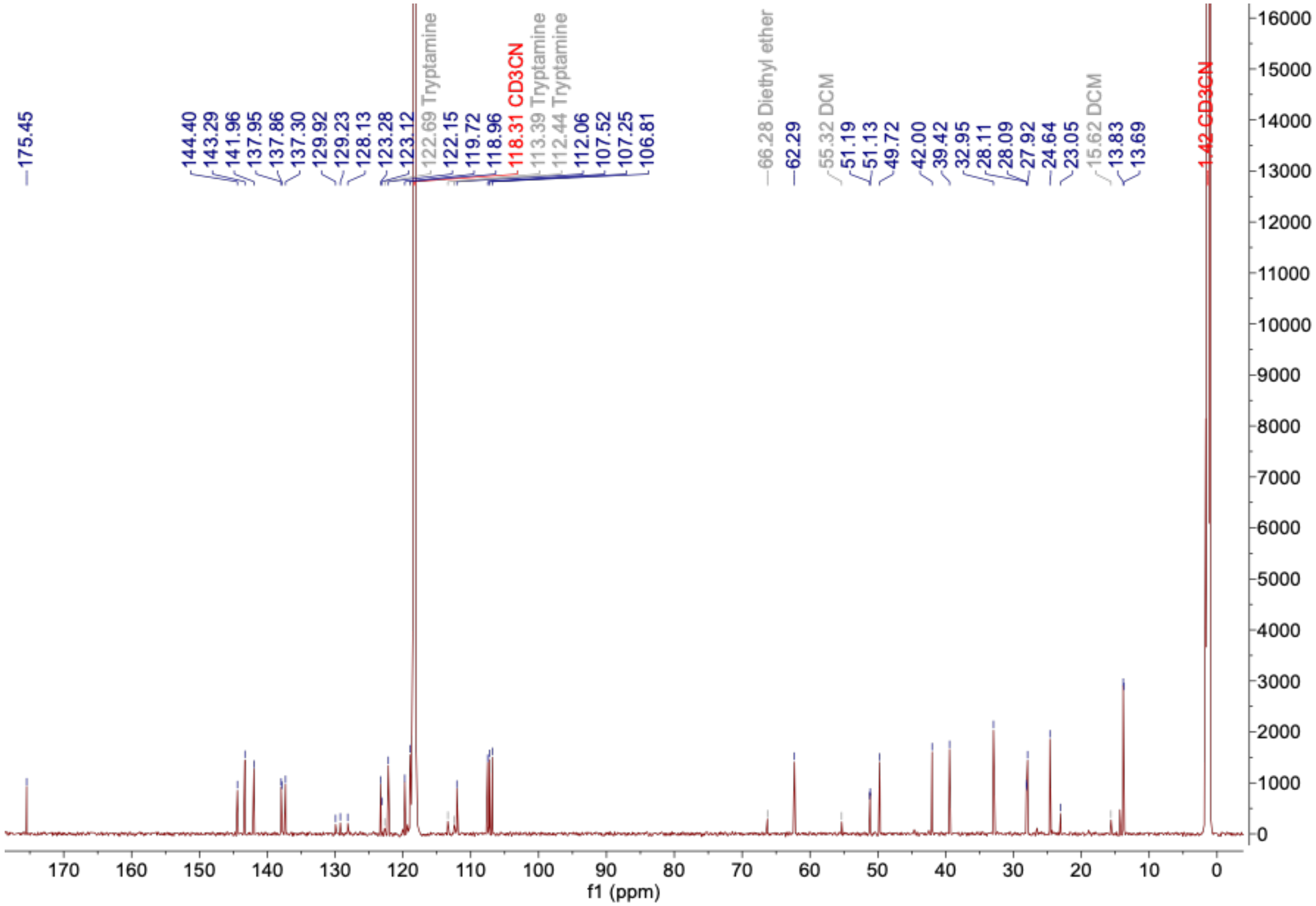

Figure S81: ^{13}C-NMR (CD_3CN) of Compound 50.

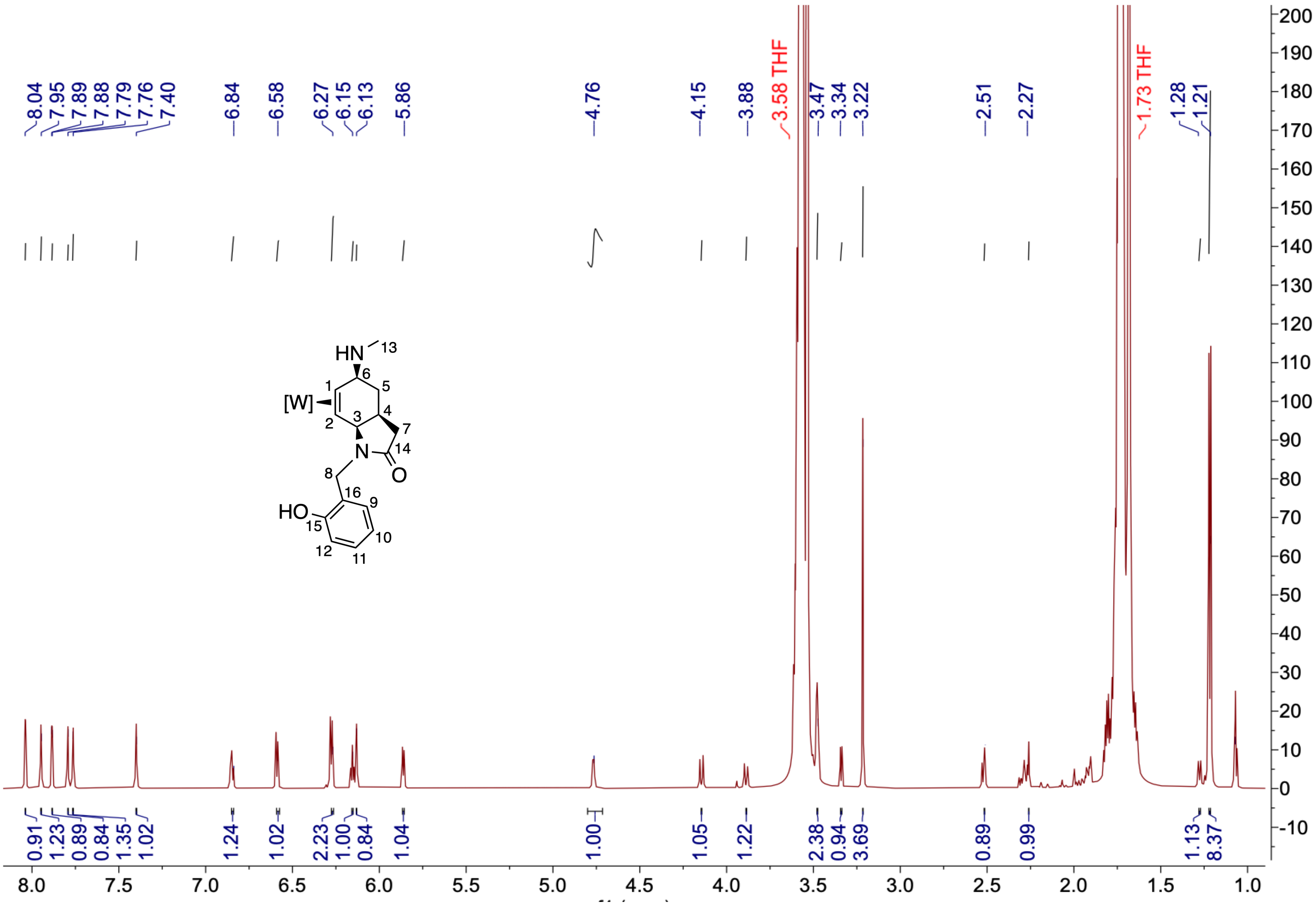

Figure S82: ^{1}H-NMR ($(CD_2)_4O$) of Compound 51.

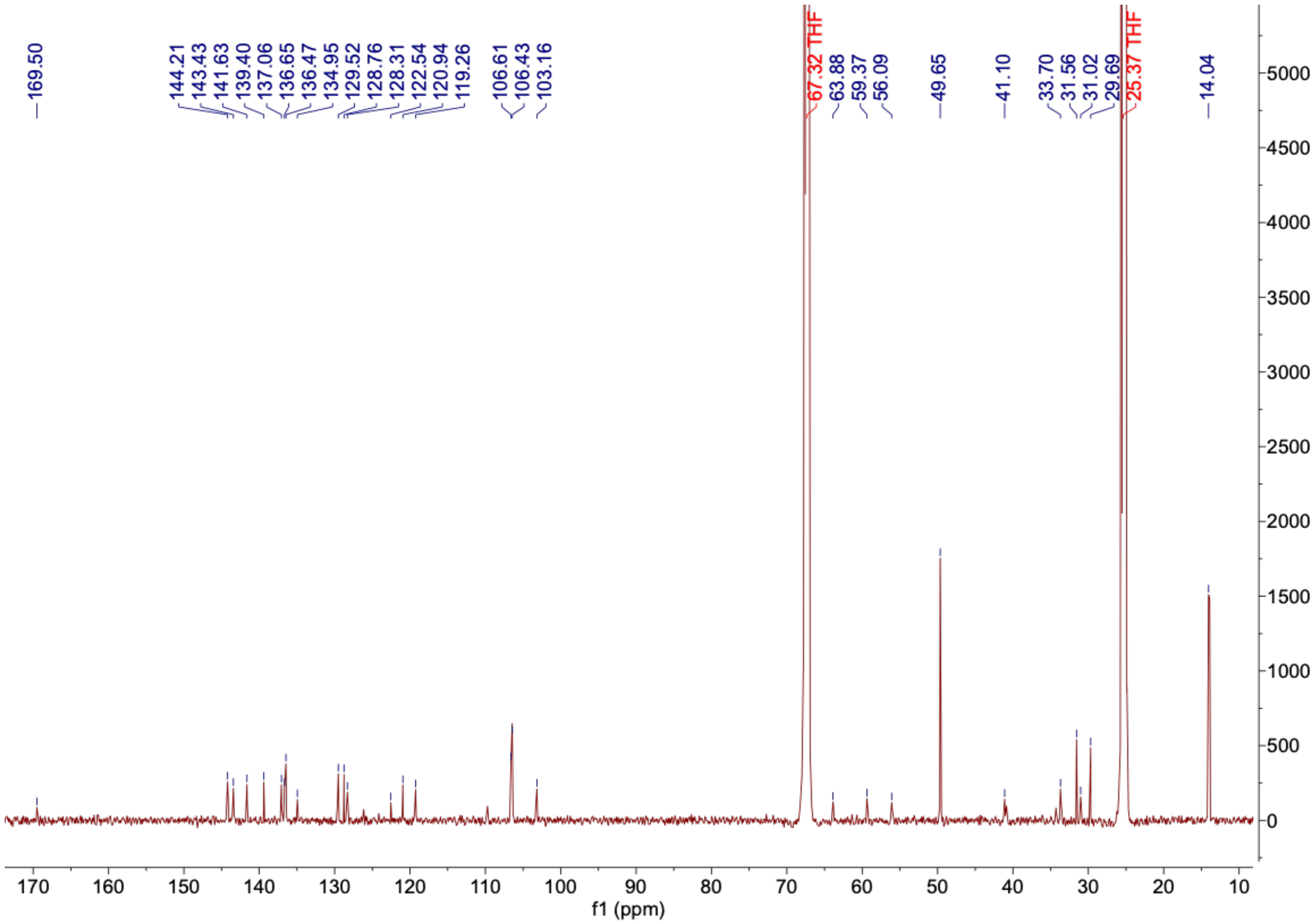

Figure S83: ^{13}C-NMR ($(CD_2)_4O$) of Compound 51.

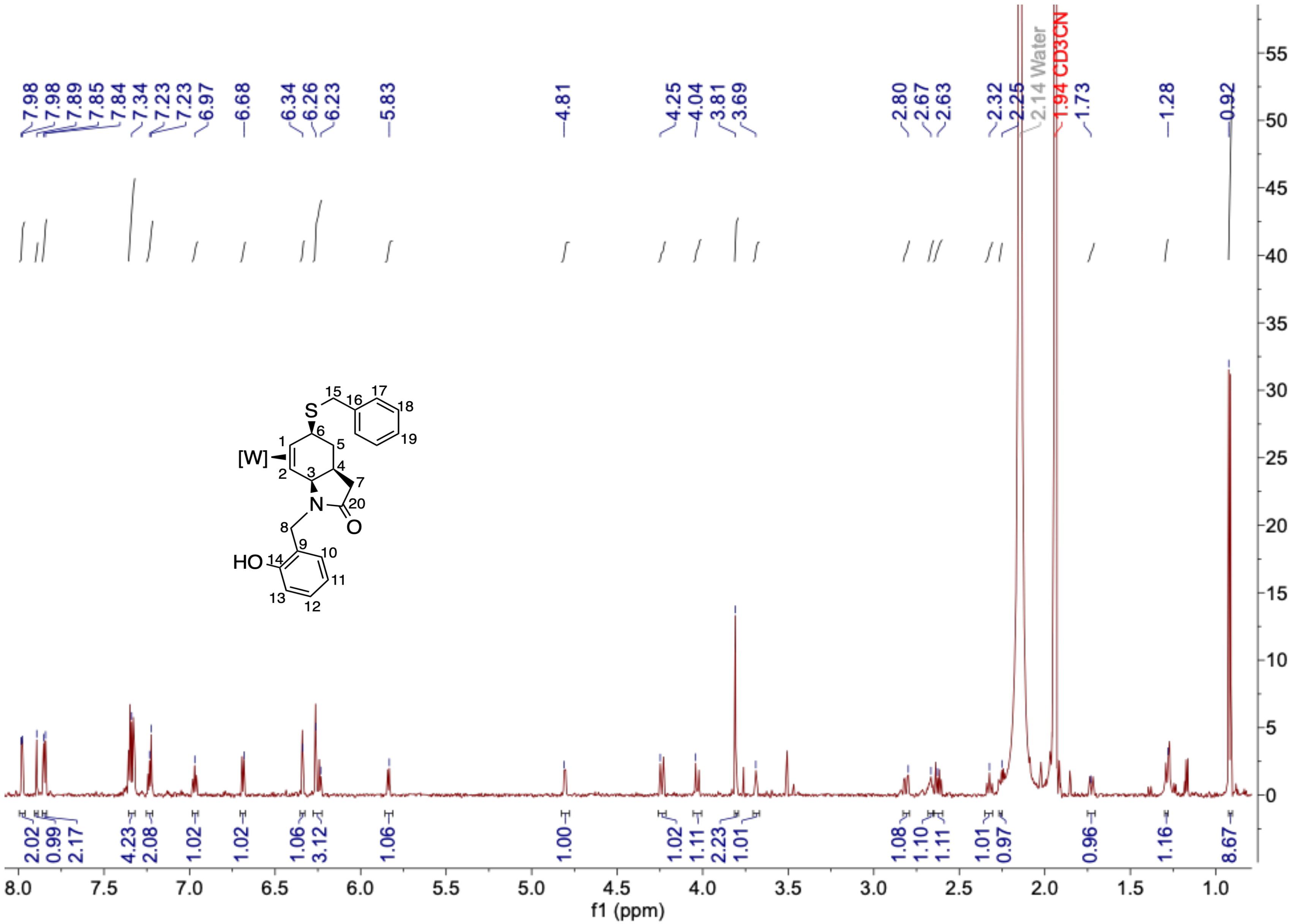

Figure S84: ^{1}H-NMR (CD_3CN) of Compound 52.

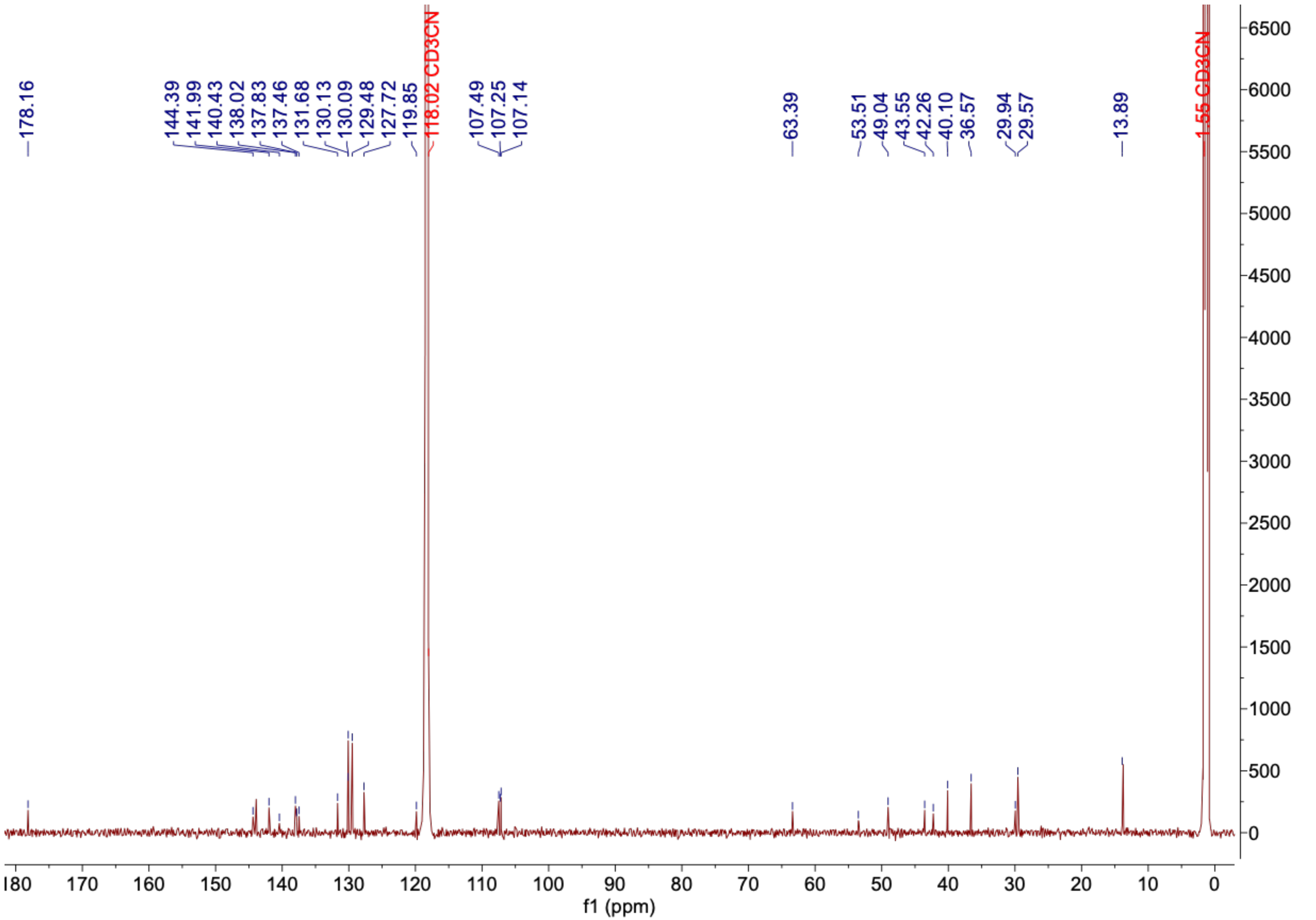

Figure S85: ^{13}C-NMR (CD_3CN) of Compound 52.

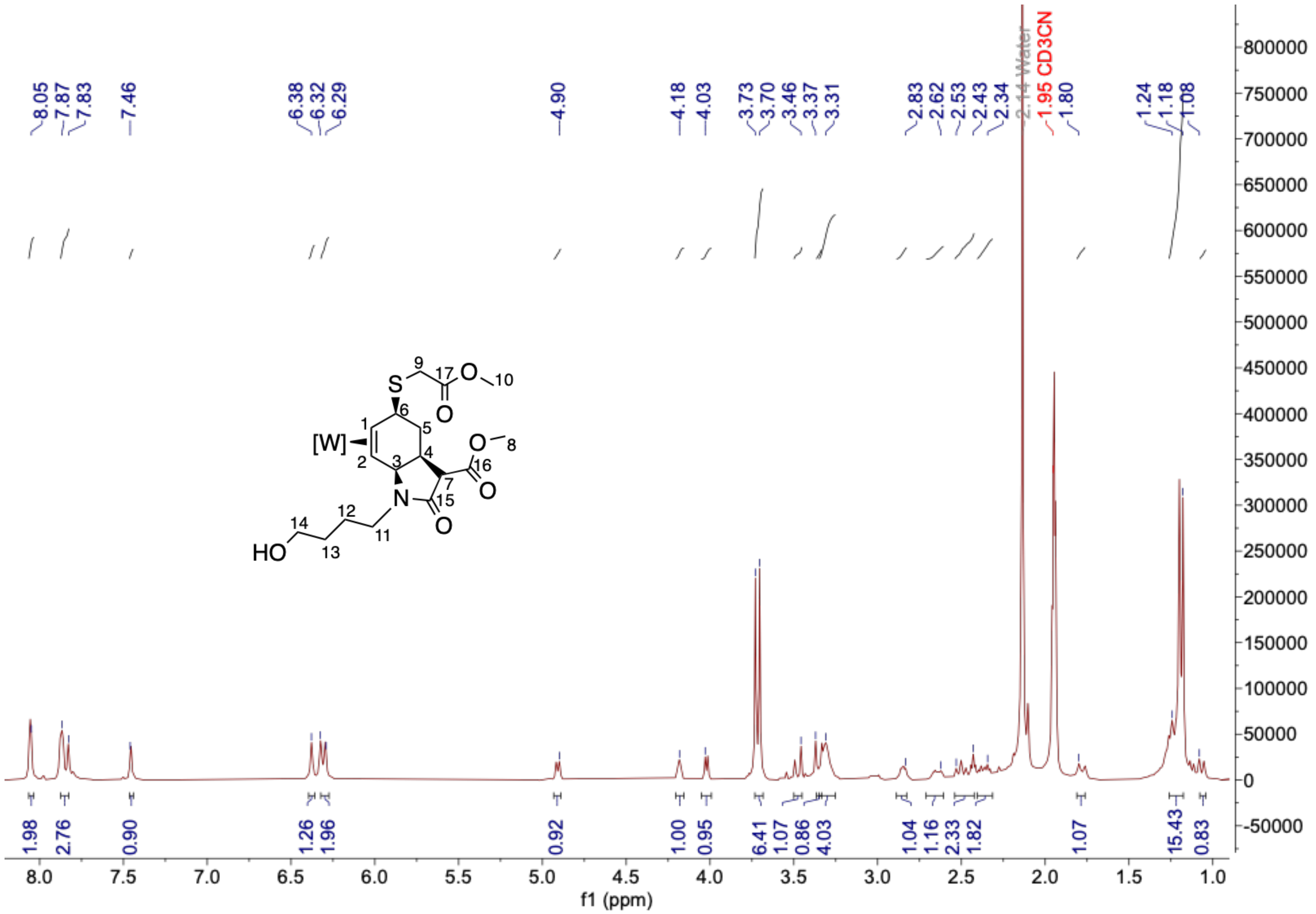

Figure S86: ^{1}H-NMR (CD_3CN) of Compound 53.

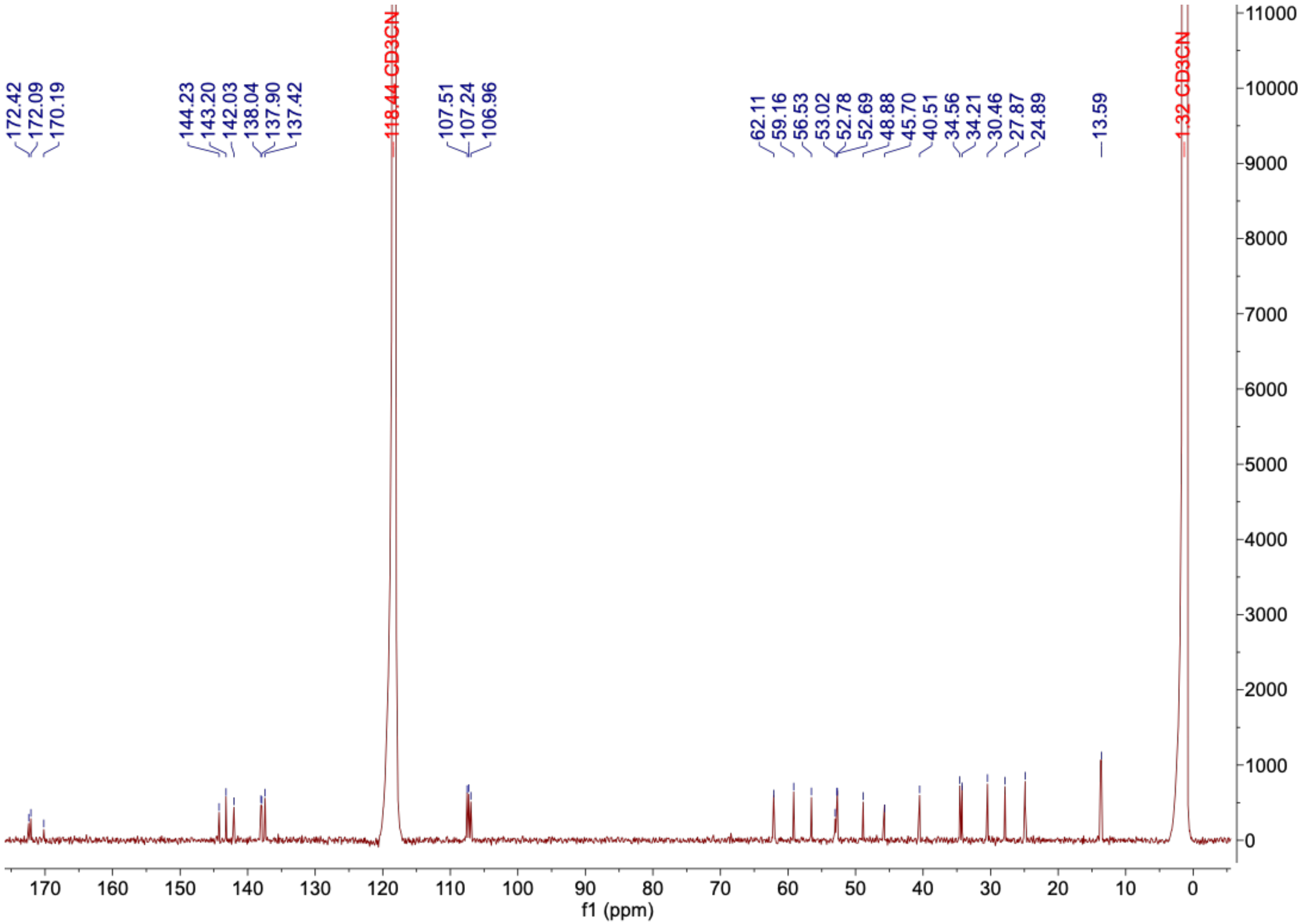

Figure S87: ^{13}C-NMR (CD_3CN) of Compound 53.

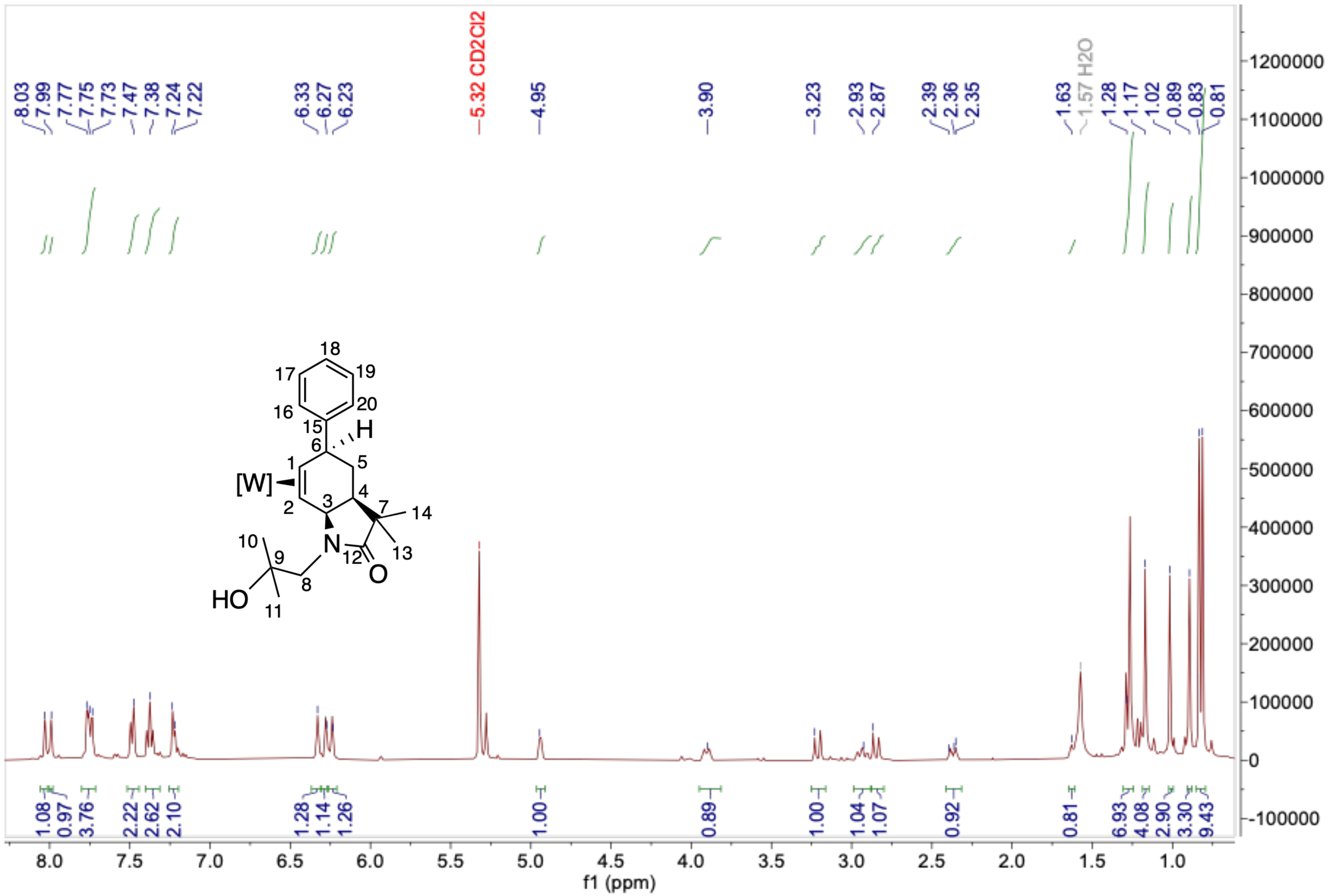

Figure S88: ^{1}H-NMR (CD_2Cl_2) of Compound 54.

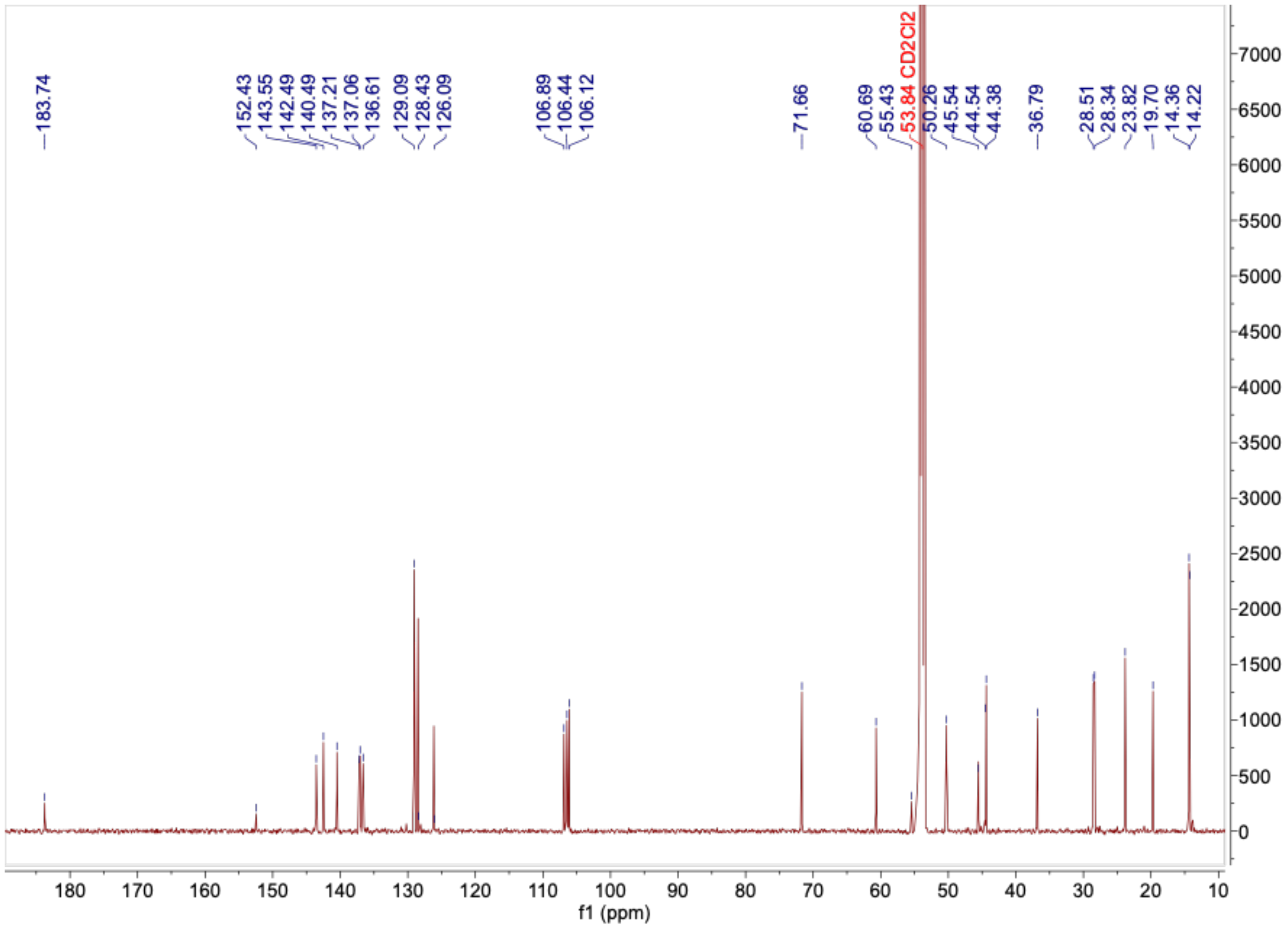

Figure S89: ^{13}C-NMR (CD_2Cl_2) of Compound 54.

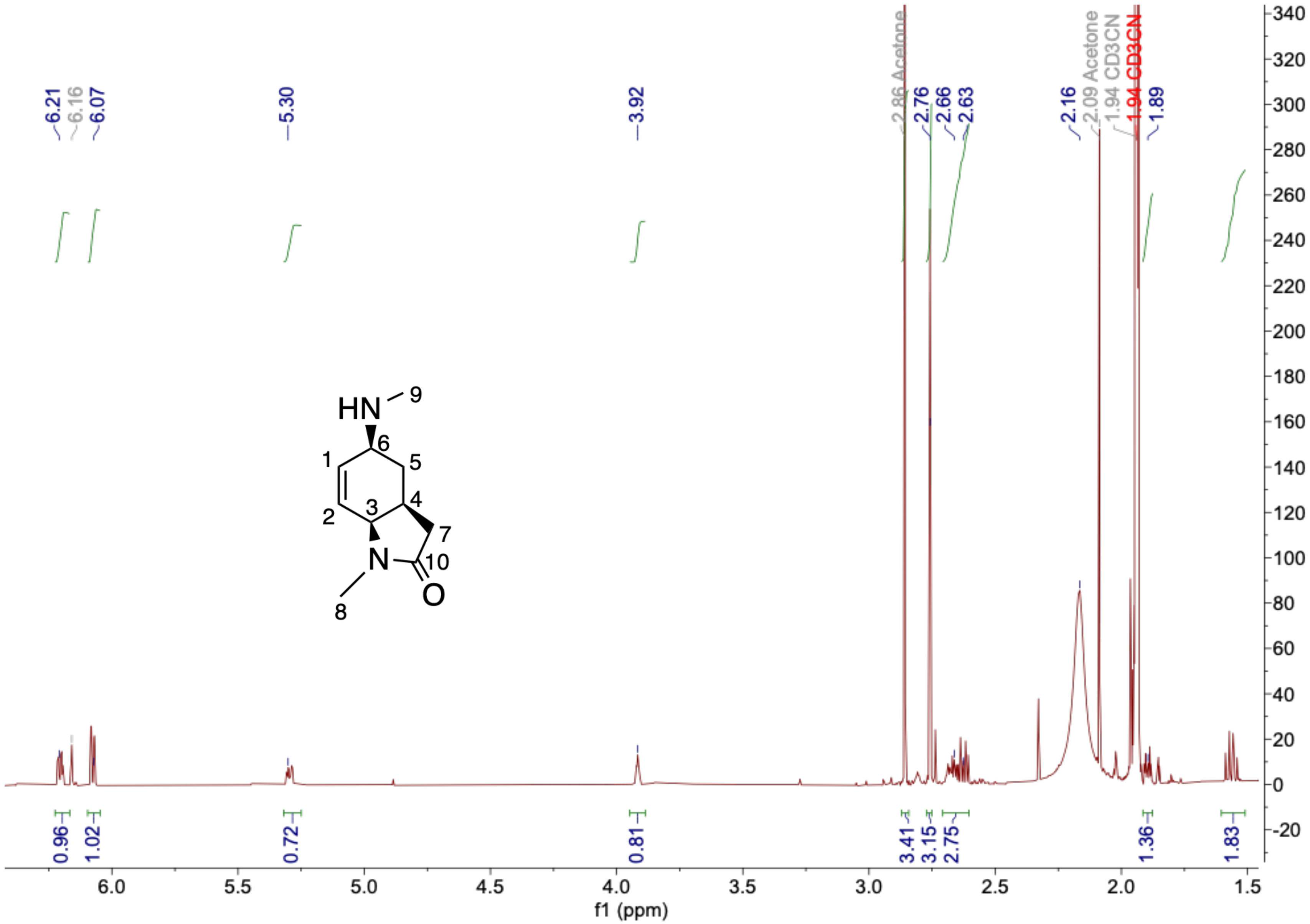

Figure S90: ^{1}H-NMR (CD_3CN) of Compound 55.

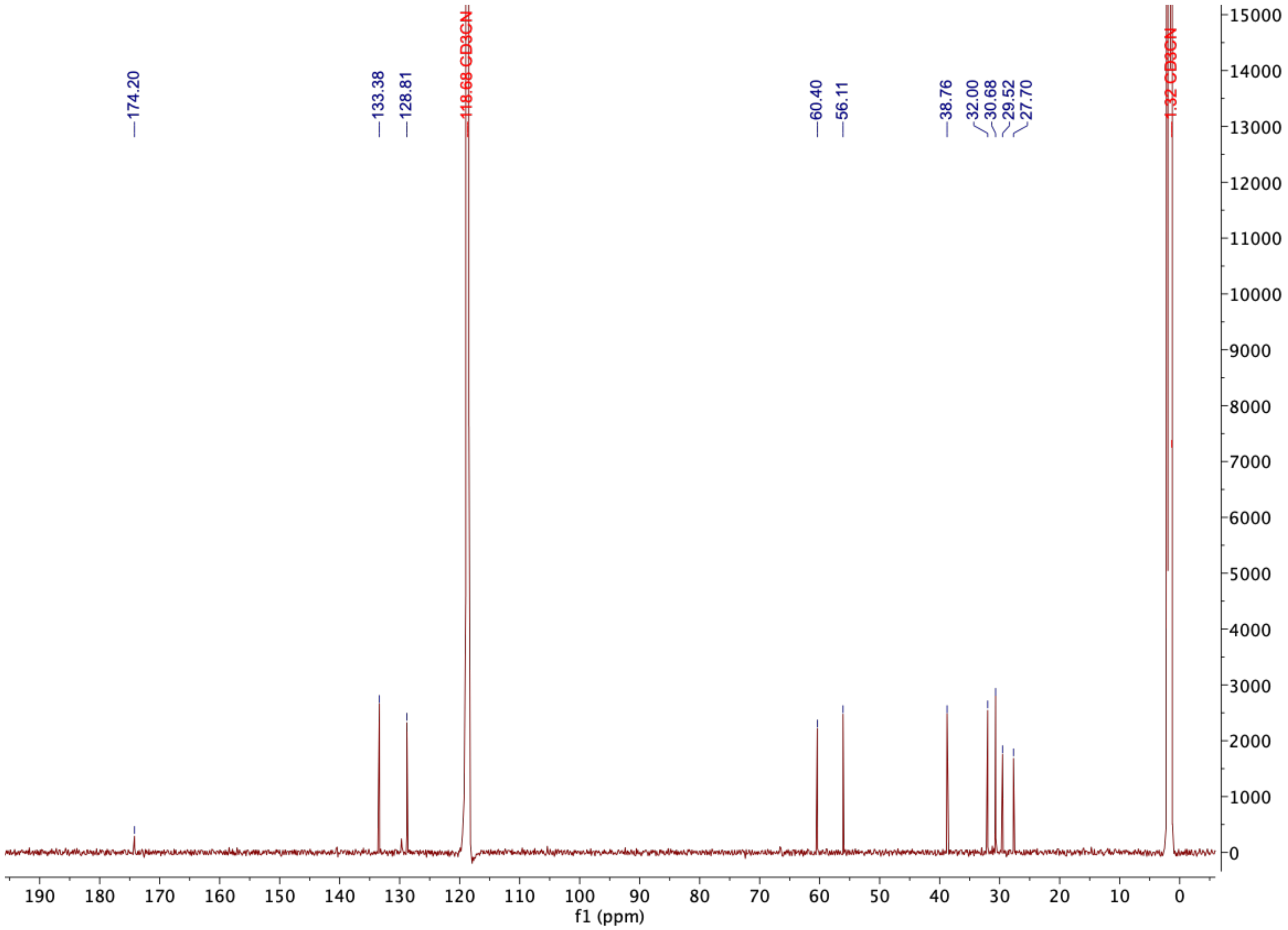

Figure S91: ^{13}C-NMR (CD_3CN) of Compound 55.

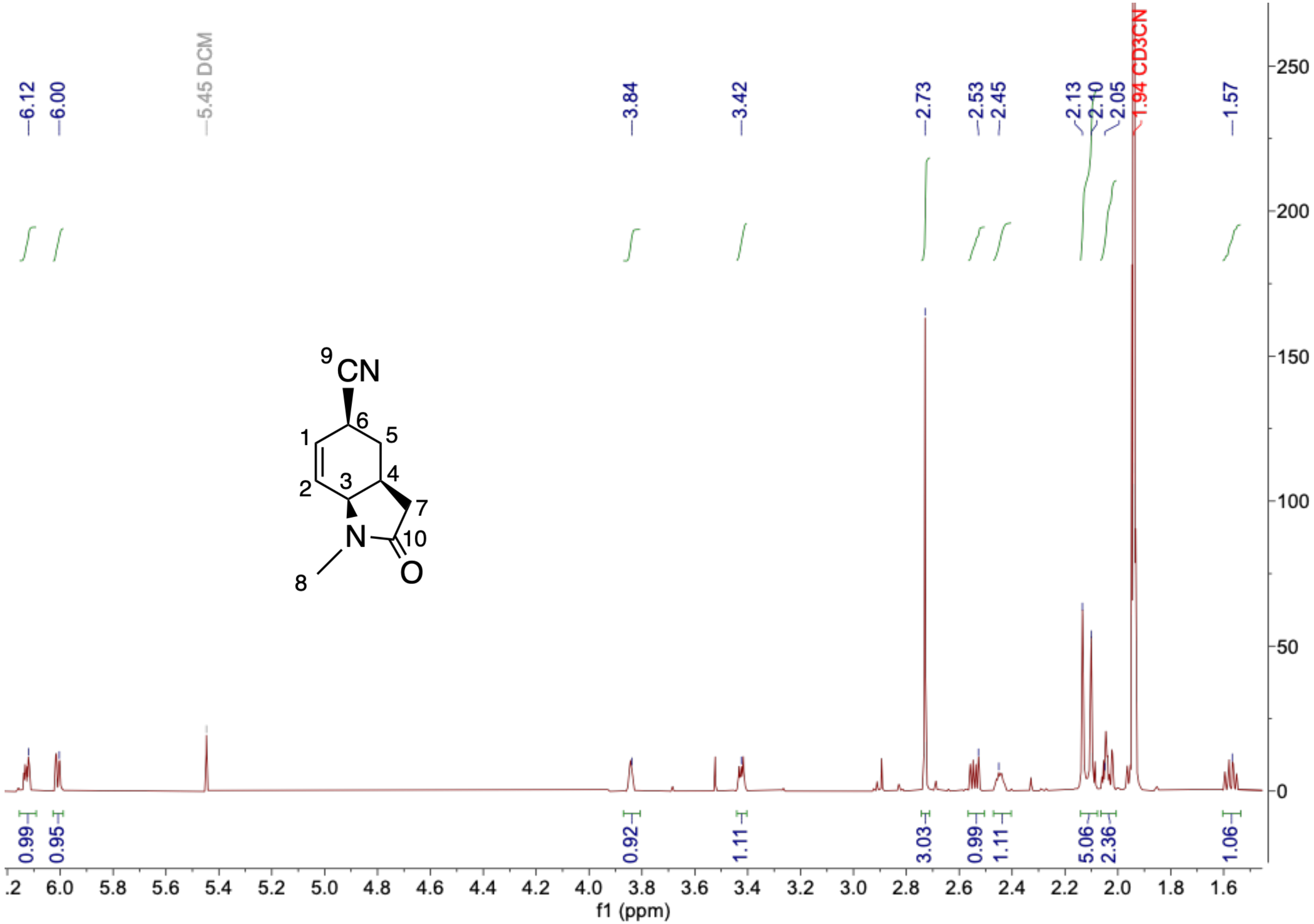

Figure S92: ^{1}H-NMR (CD_3CN) of Compound 56.

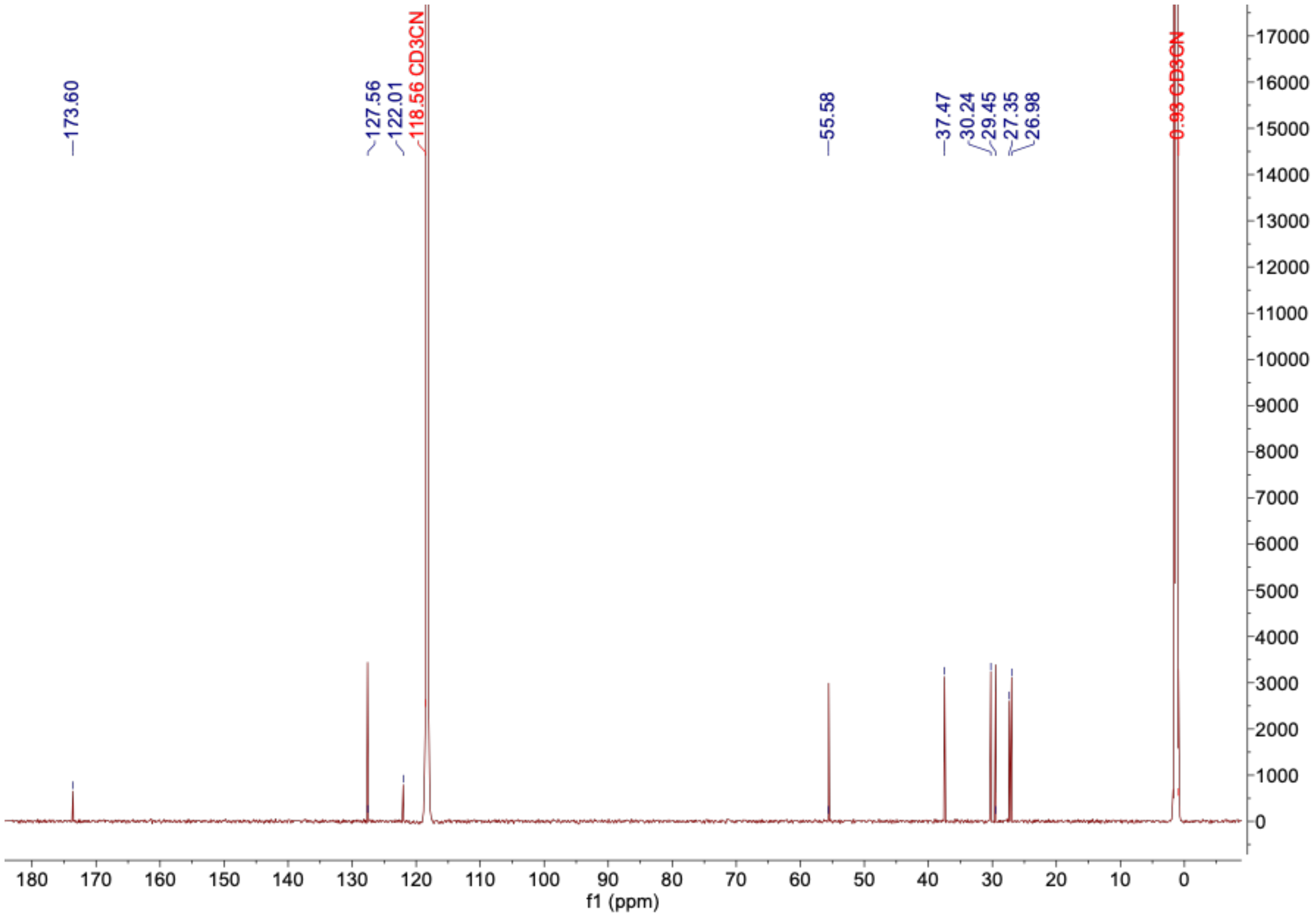

Figure S93: ^{13}C-NMR (CD_3CN) of Compound 56.

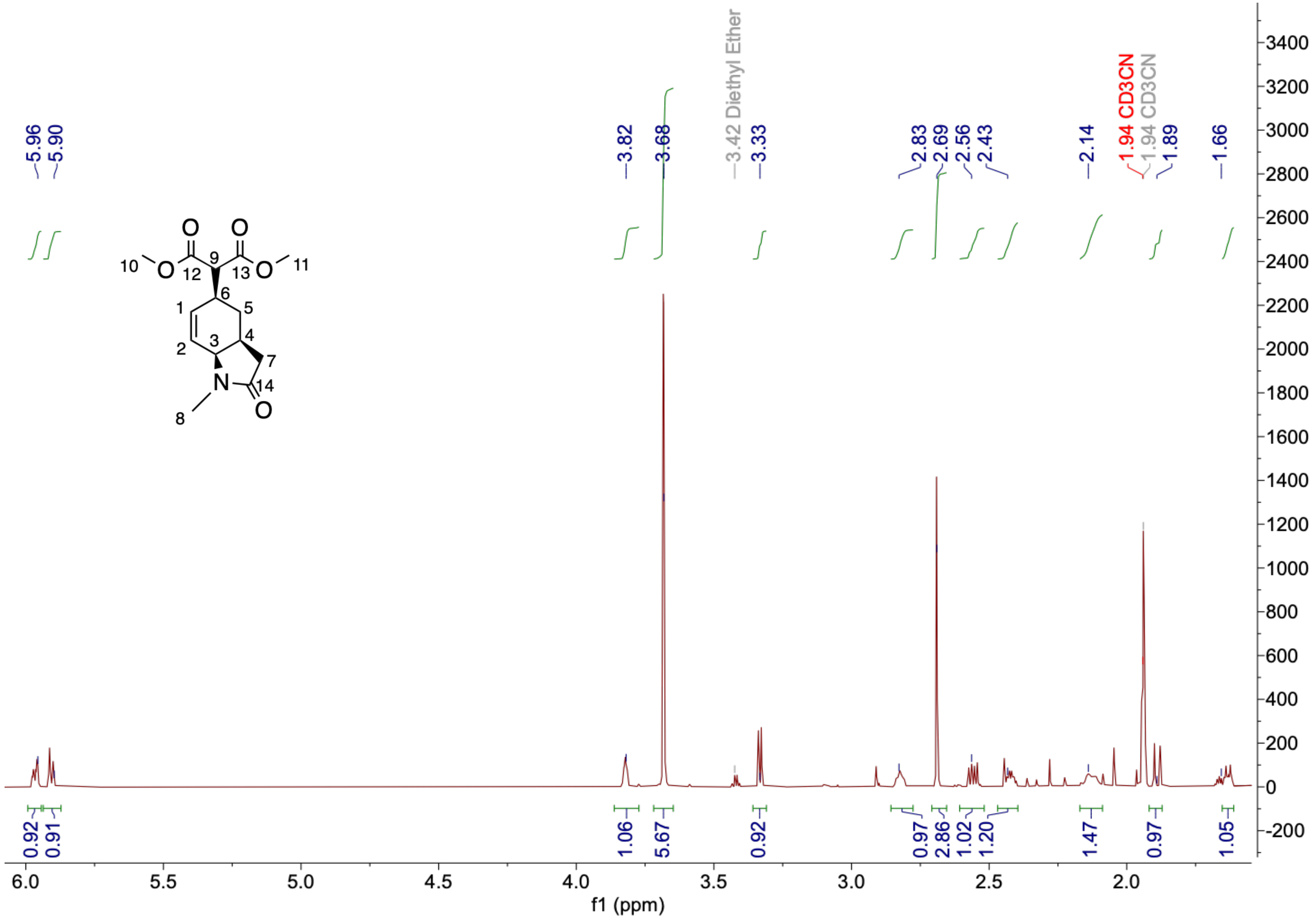

Figure S94: ^{1}H-NMR (CD_3CN) of Compound 57.

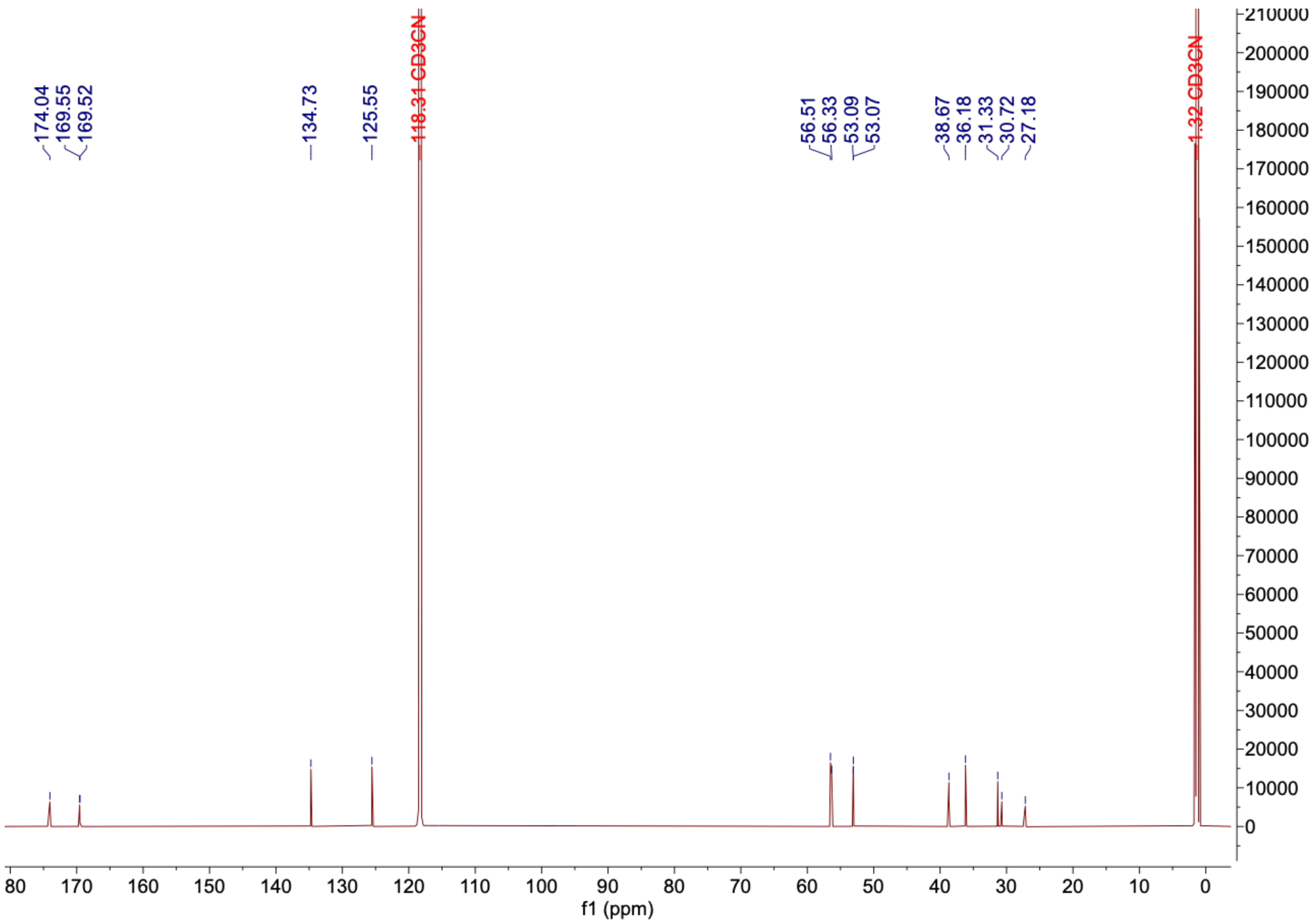

Figure S95: ^{13}C-NMR (CD_3CN) of Compound 57.

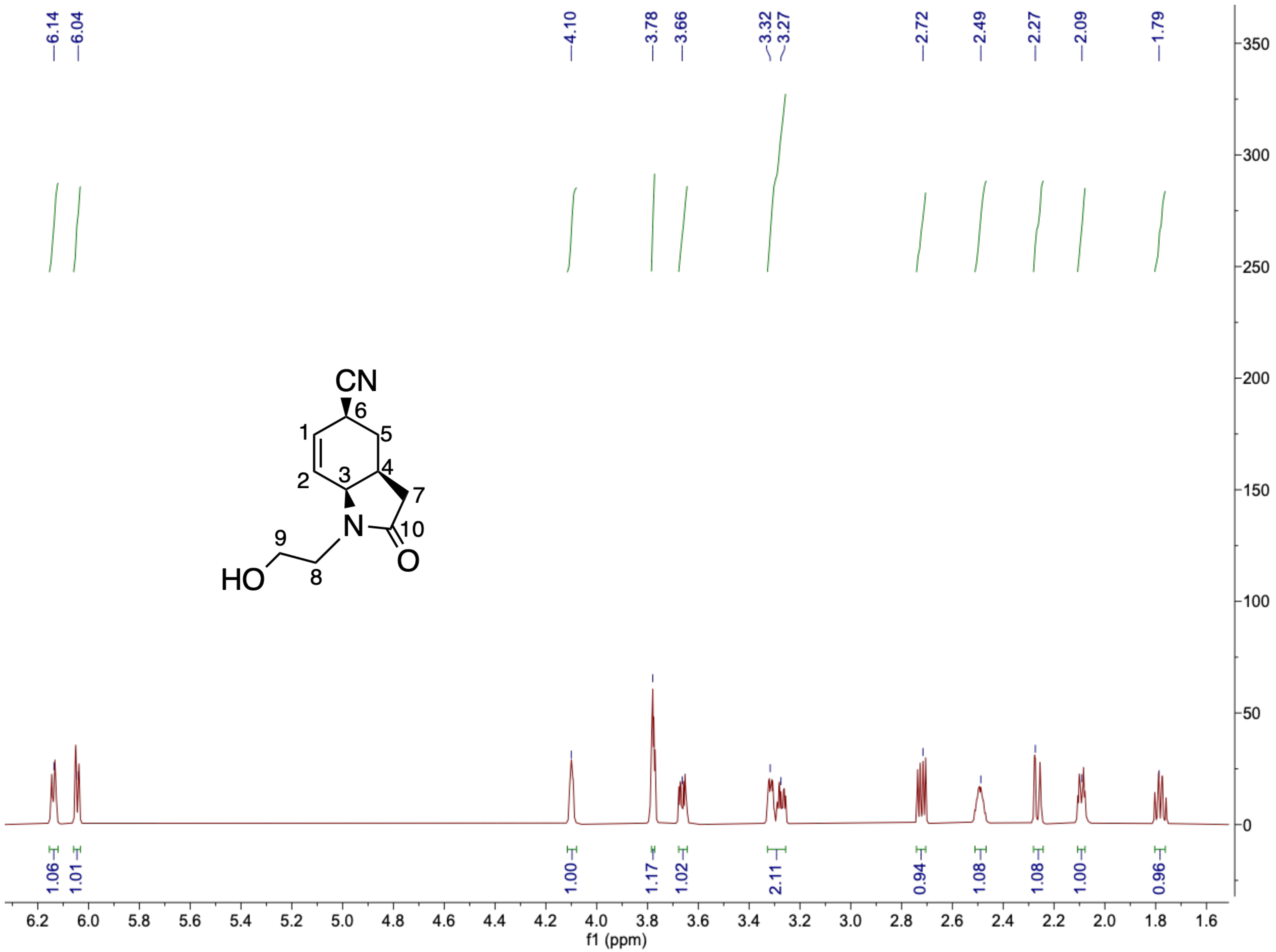

Figure S96: ^{1}H-NMR ($CDCl_3$) of Compound 58.

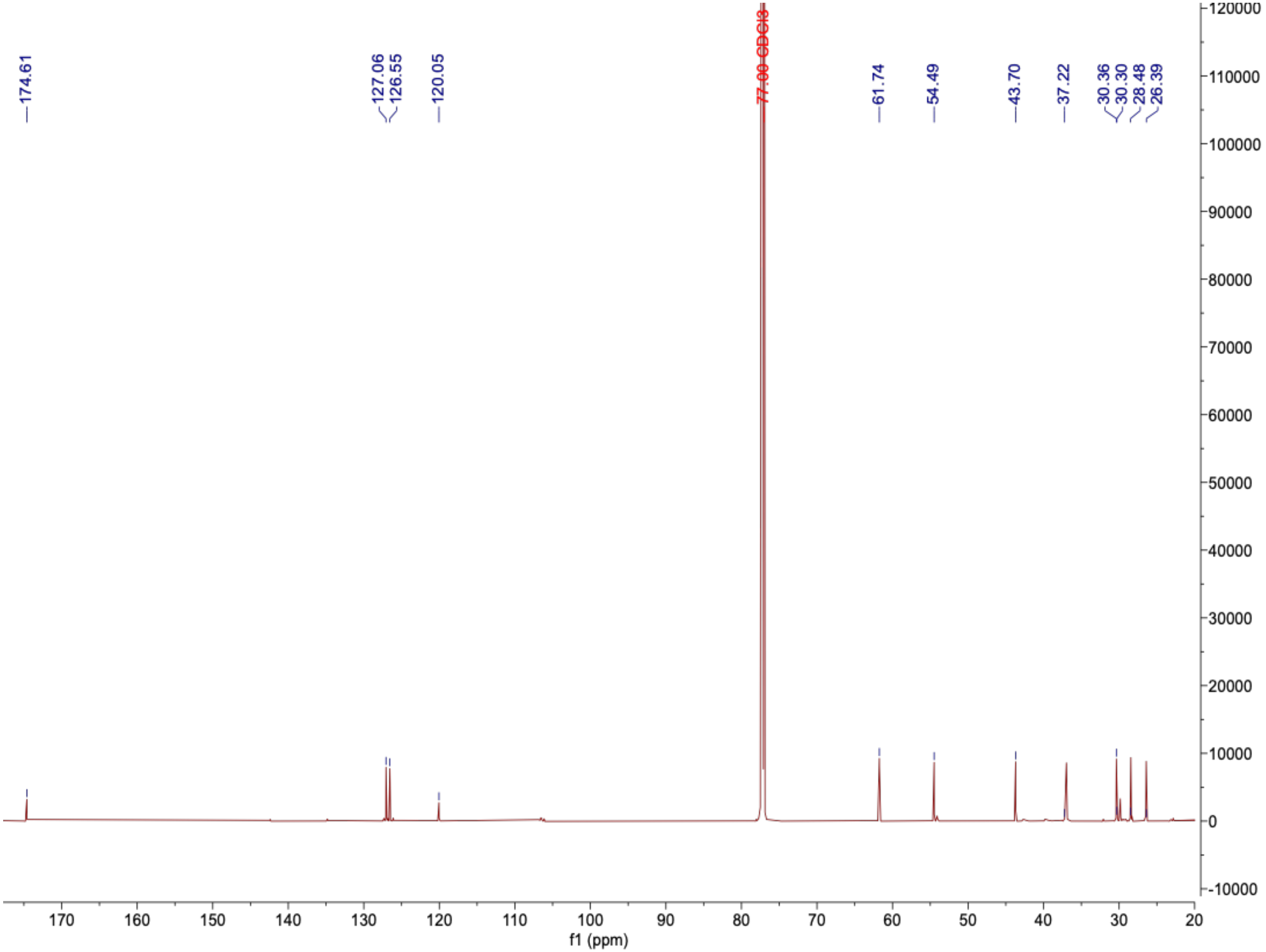

Figure S97: ^{13}C-NMR ($CDCl_3$) of Compound 58.

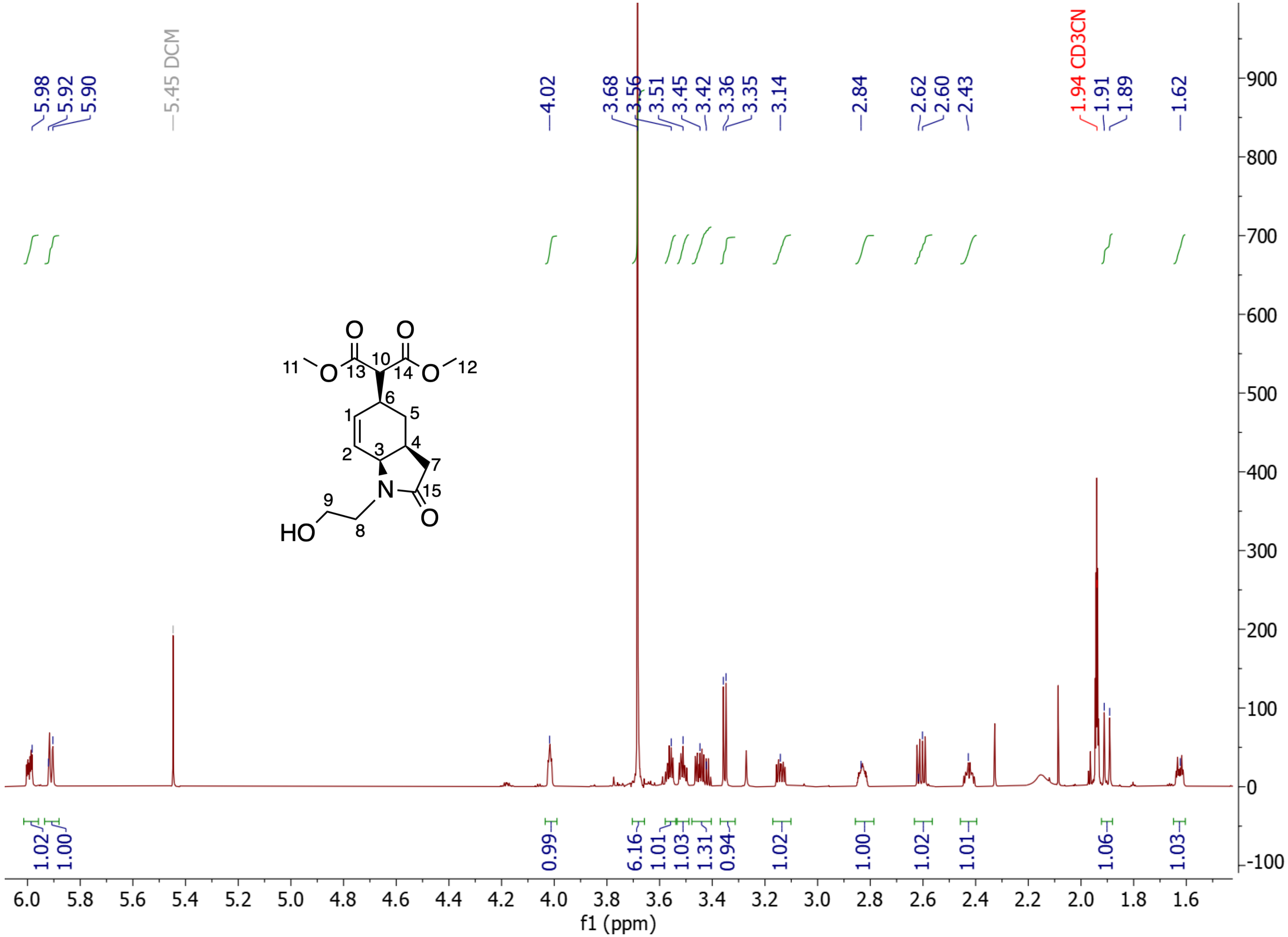

Figure S98: ^{1}H-NMR (CD_3CN) of Compound 59.

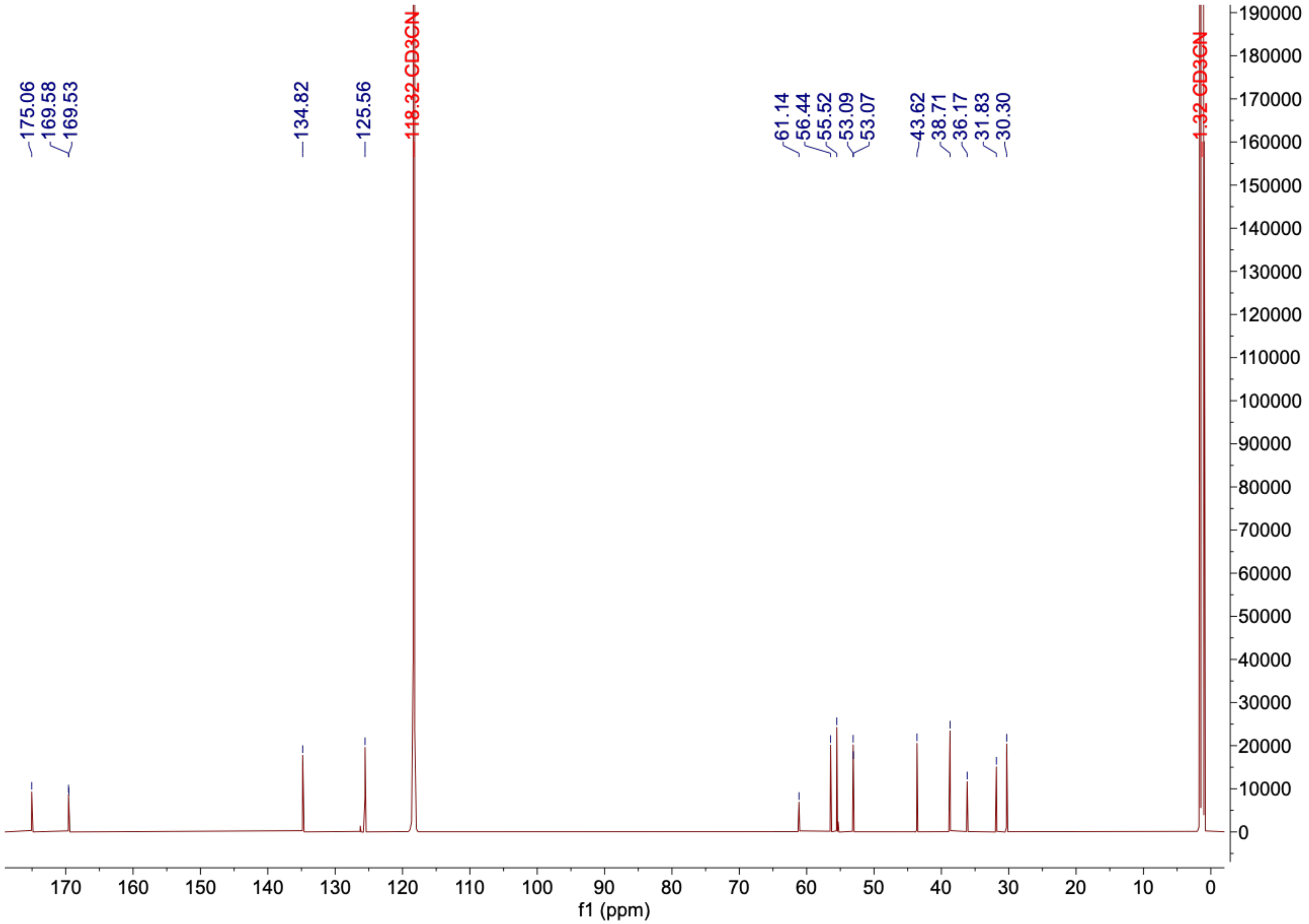

Figure S99: ^{13}C-NMR (CD_3CN) of Compound 59.

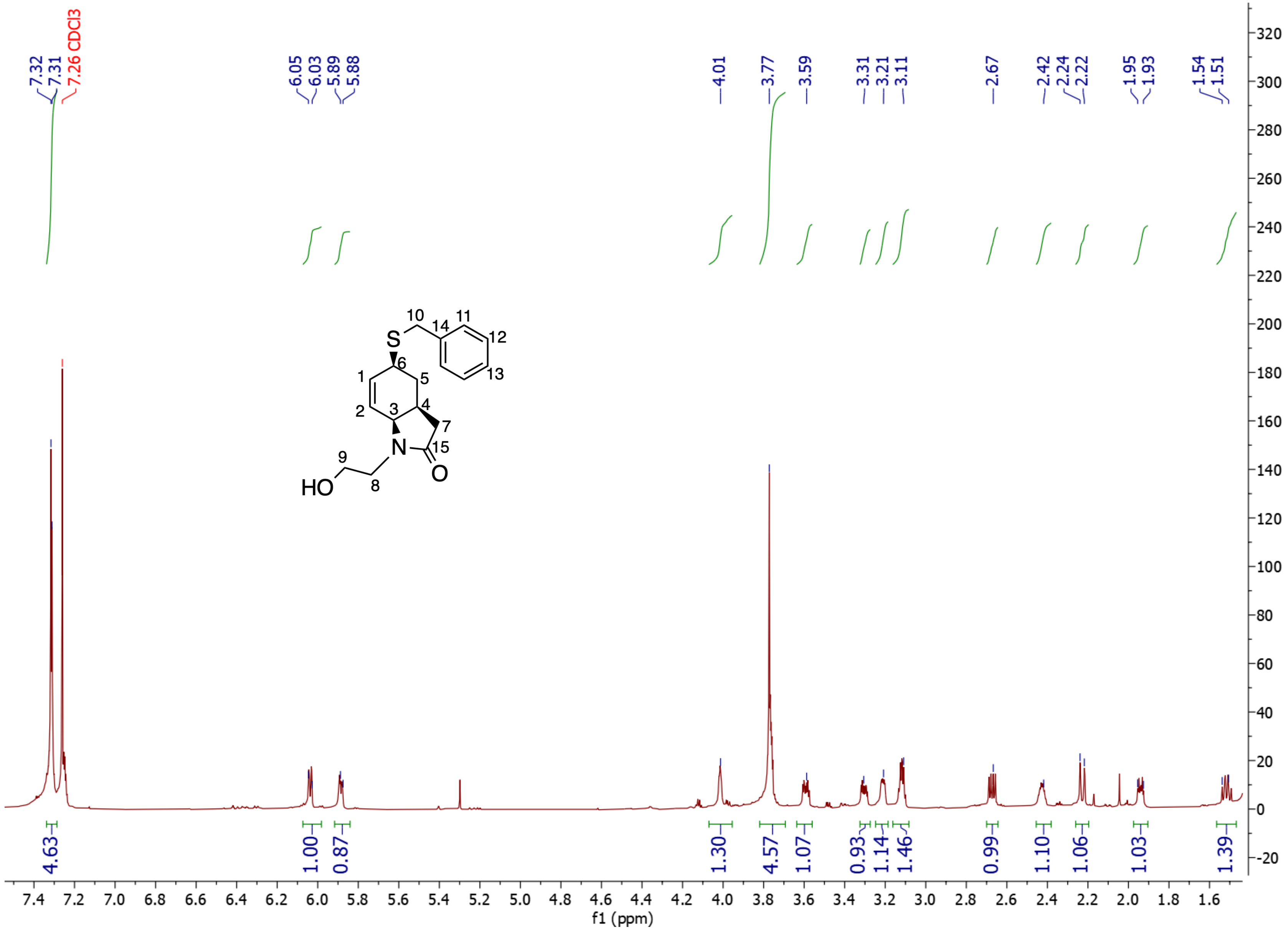

Figure S100: ^{1}H-NMR ($CDCl_3$) of Compound 60.

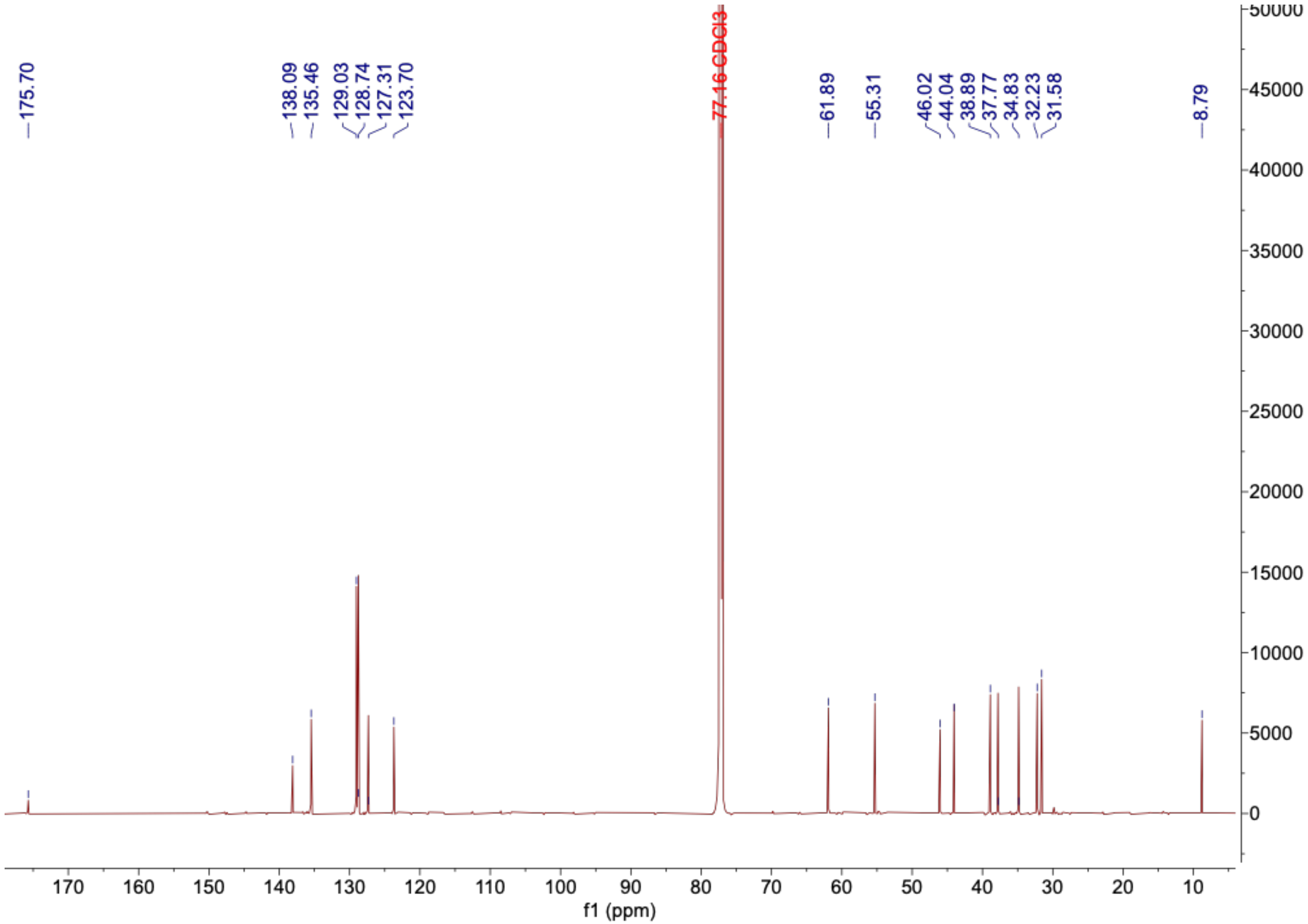

Figure S101: ^{13}C-NMR ($CDCl_3$) of Compound 60.

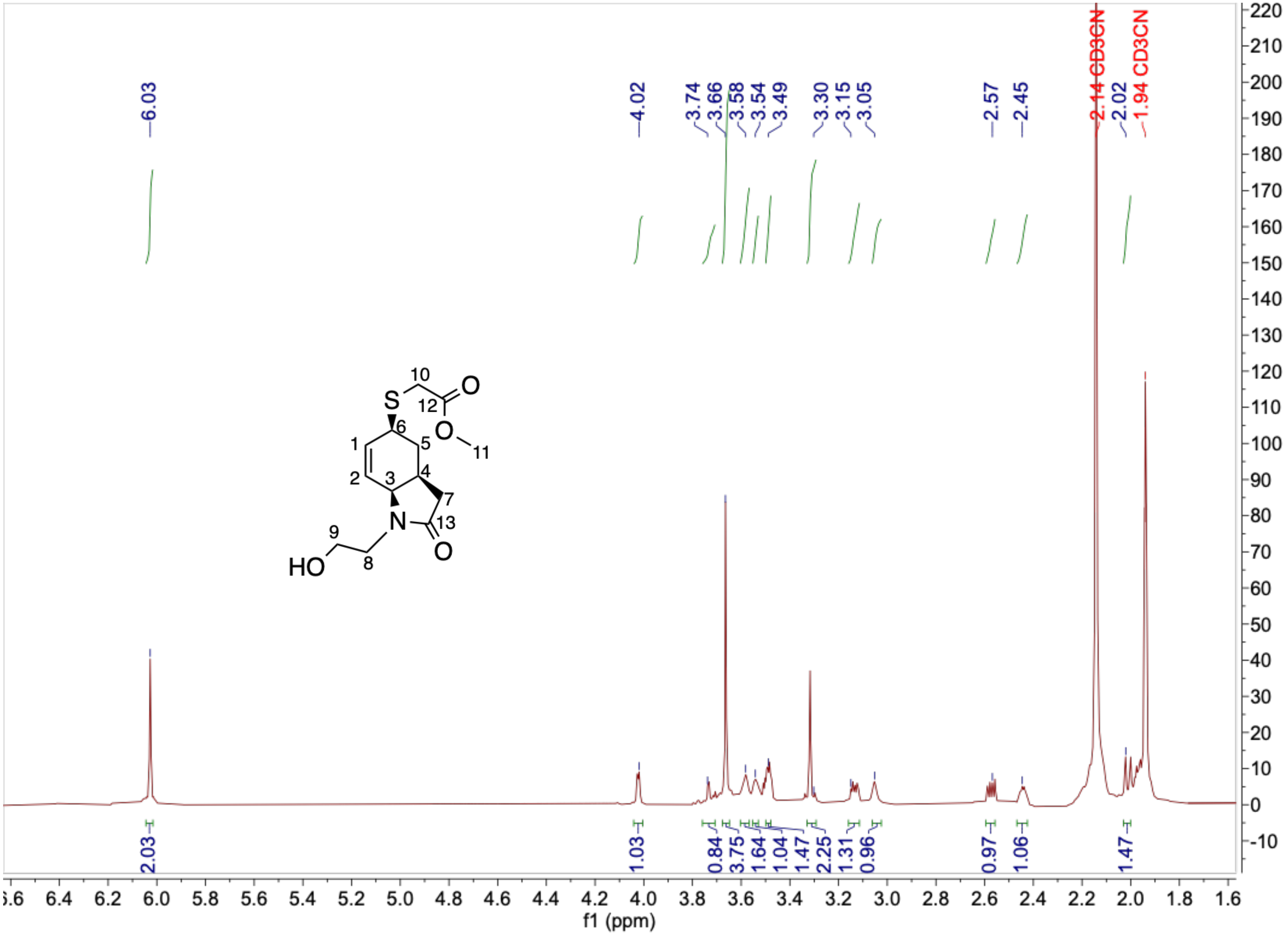

Figure S102: ^{1}H-NMR (CD_3CN) of Compound 61.

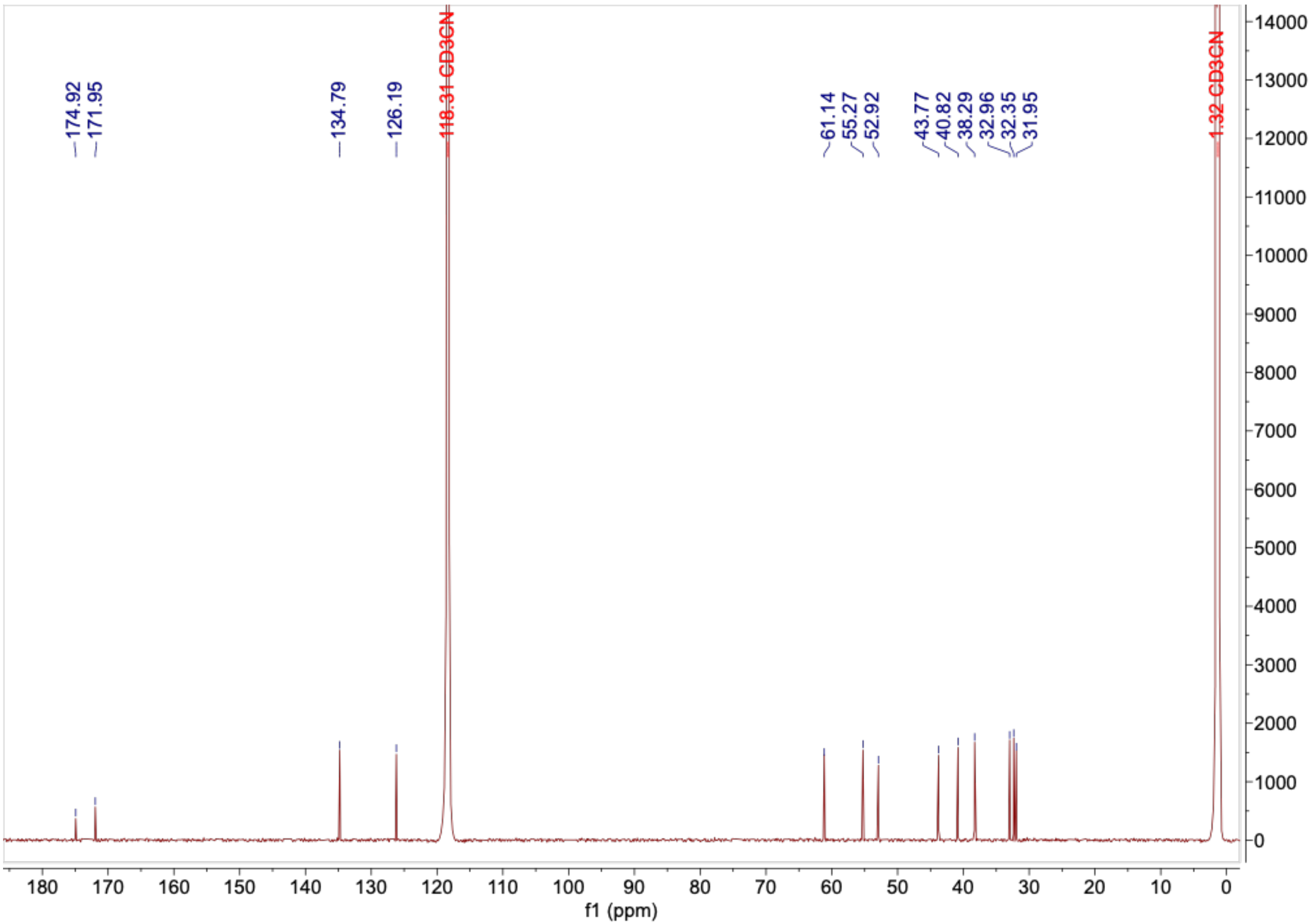

Figure S103: ^{13}C-NMR (CD_3CN) of Compound 61.

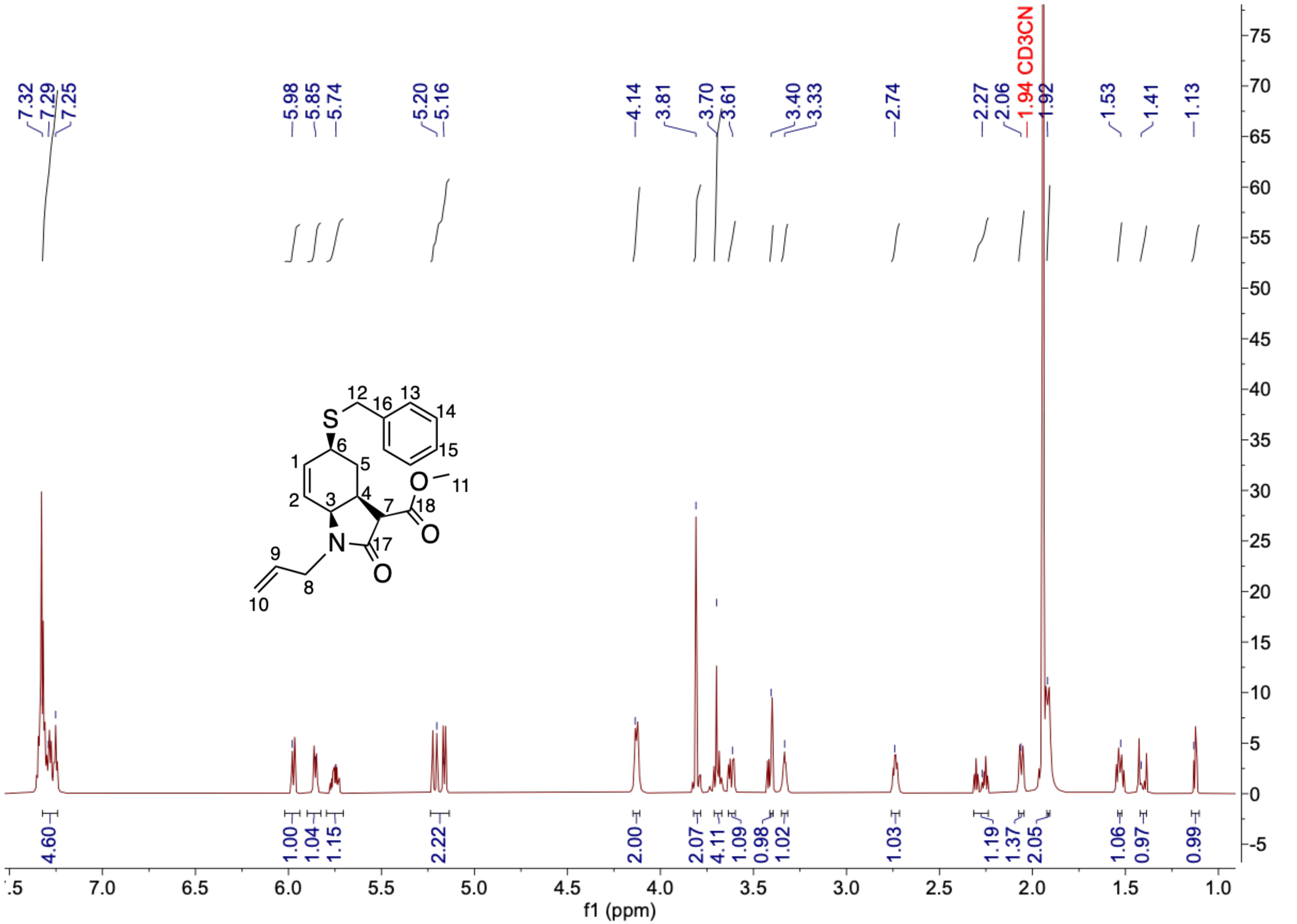

Figure S104: ^{1}H-NMR (CD_3CN) of Compound 62.

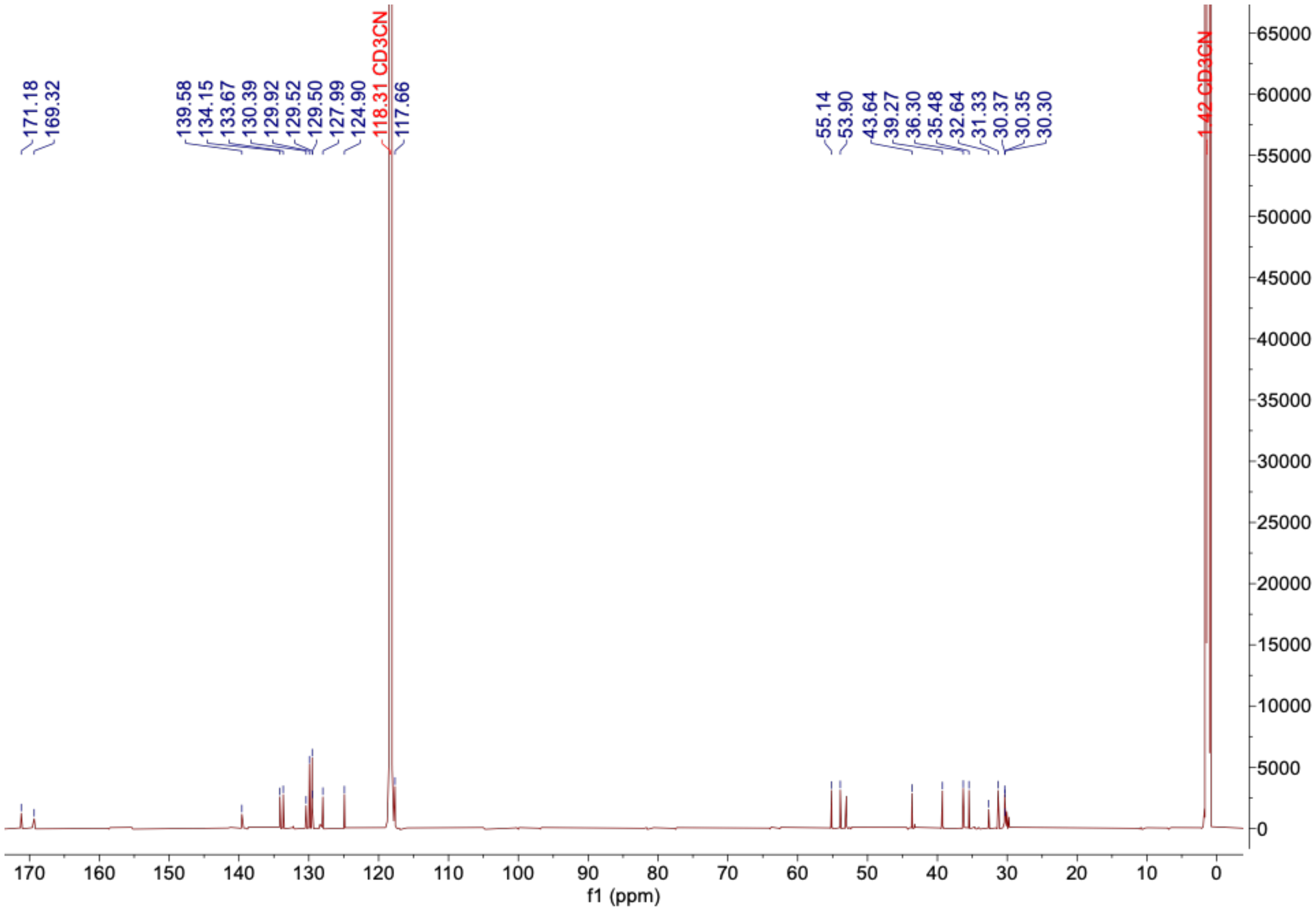

Figure S105: ^{13}C-NMR (CD_3CN) of Compound 62.

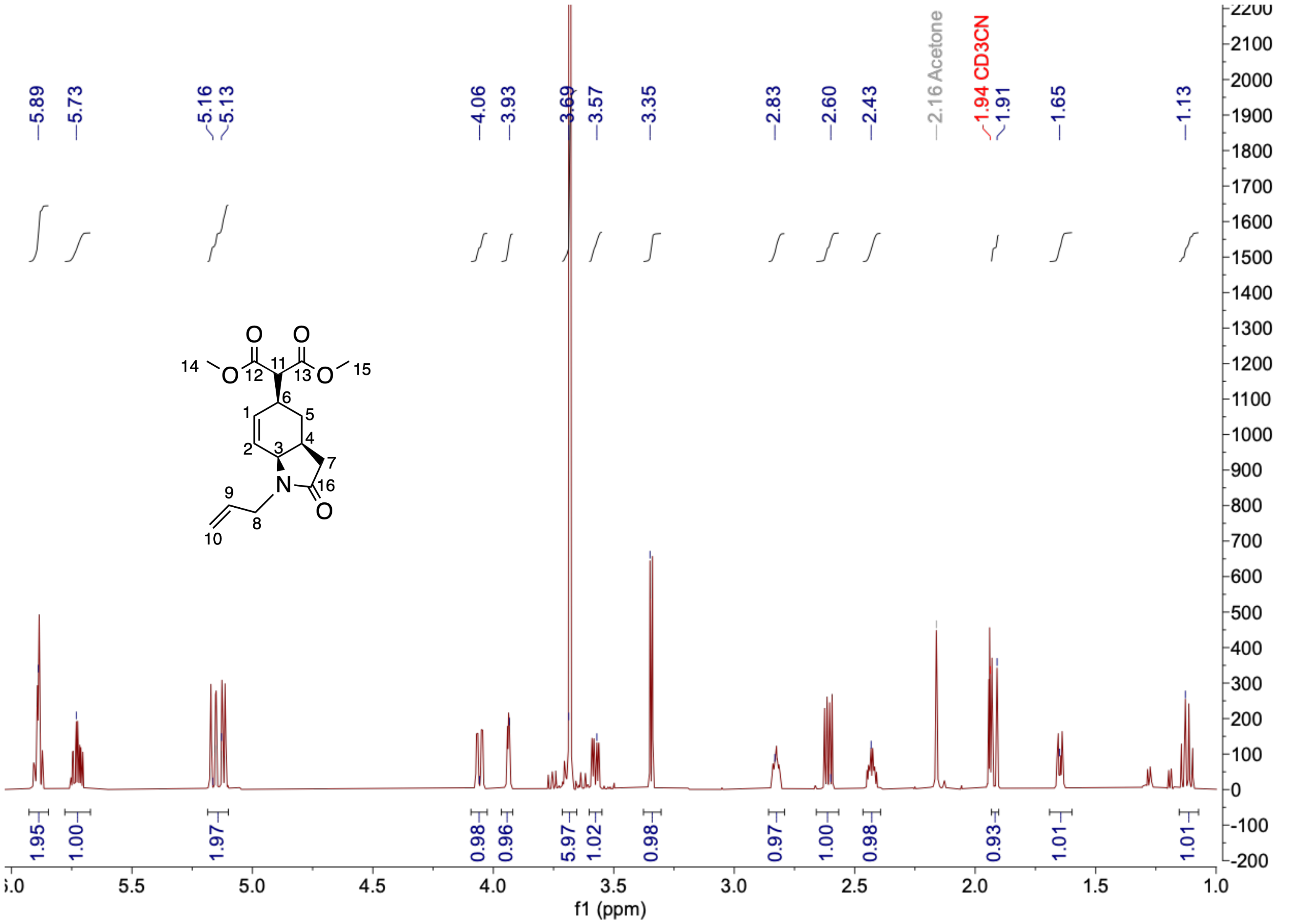

Figure S106: ^{1}H-NMR (CD_3CN) of Compound 63.

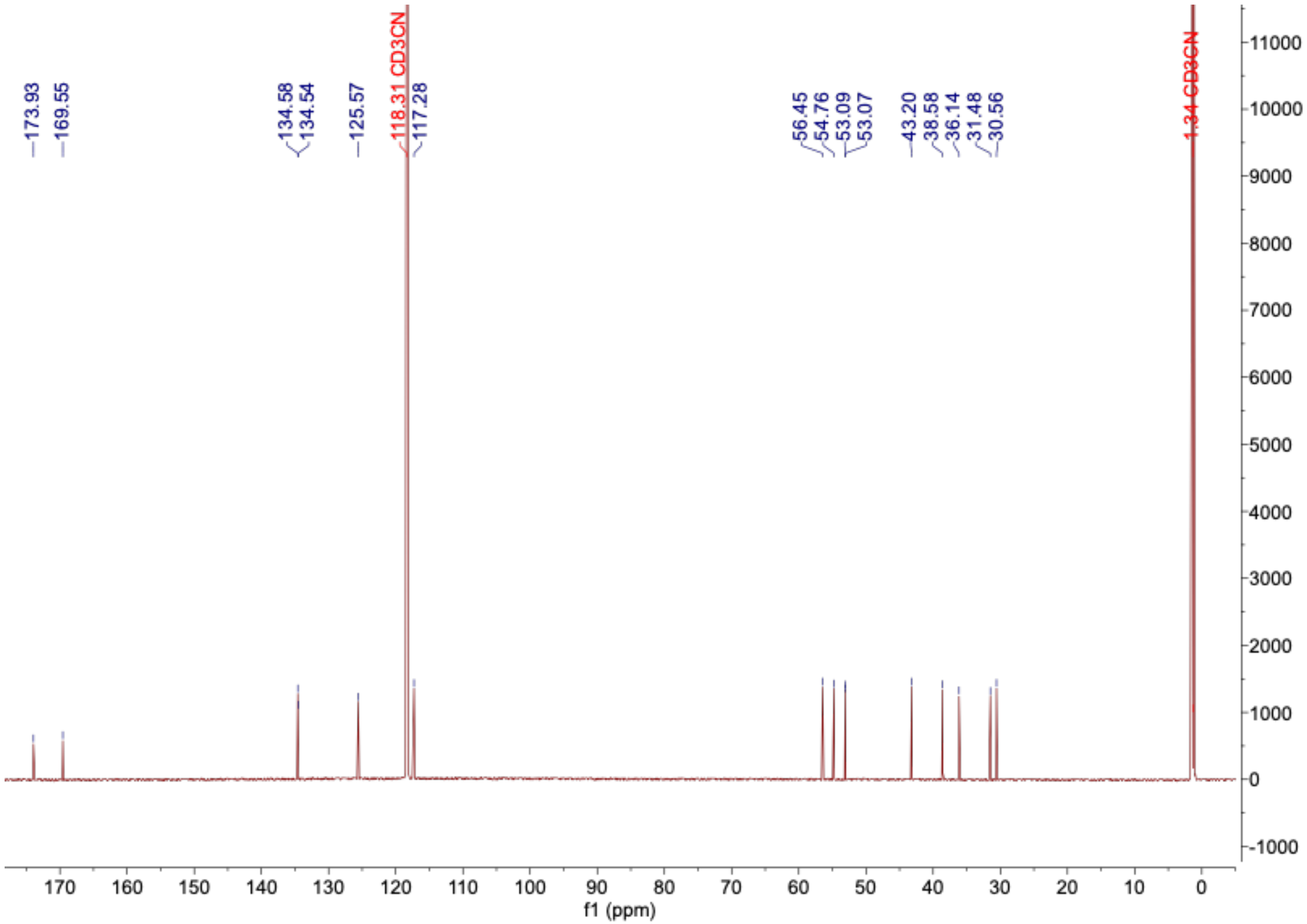

Figure S107: ^{13}C-NMR (CD_3CN) of Compound 63.

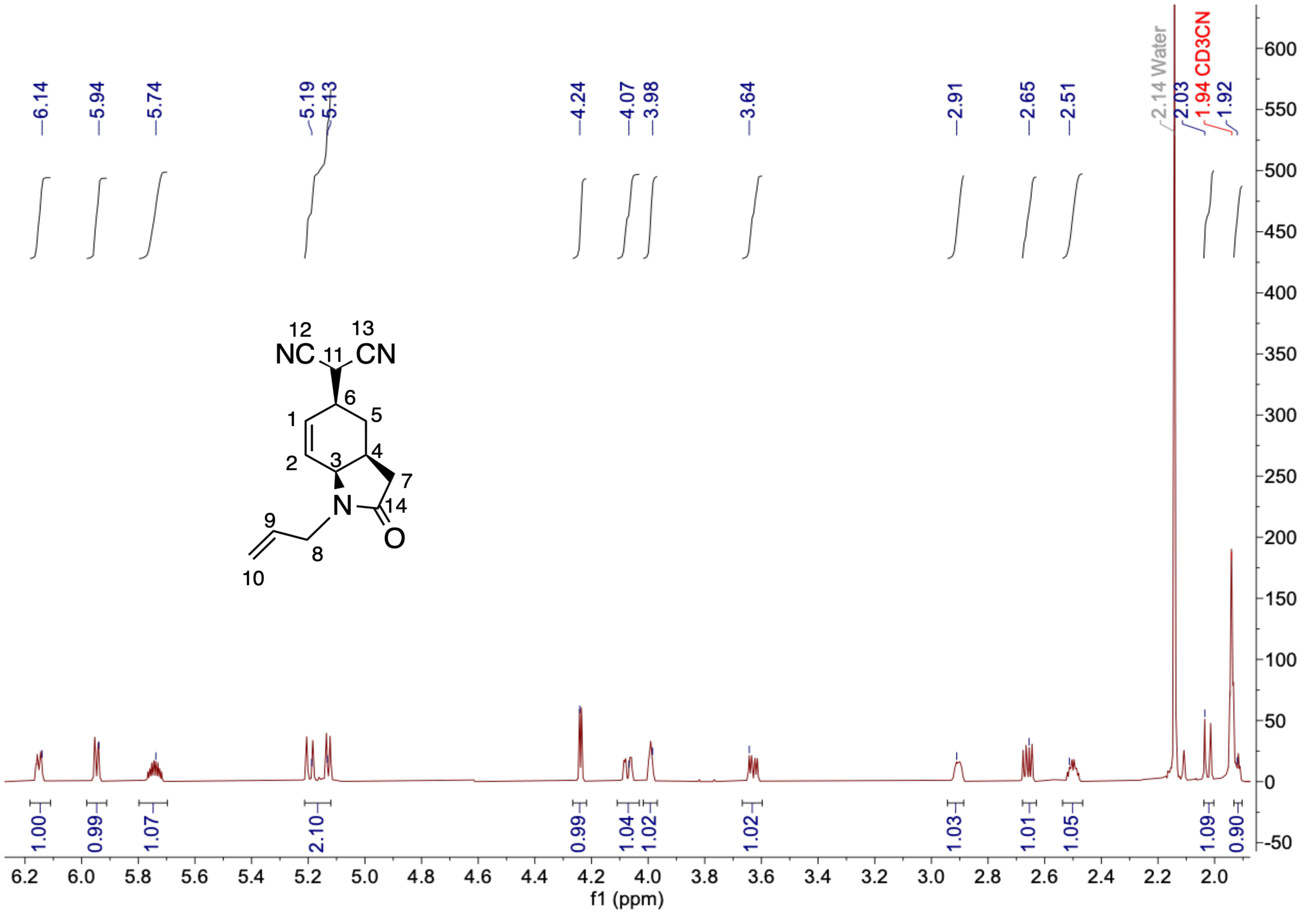

Figure S108: ^{1}H-NMR (CD_3CN) of Compound 64.

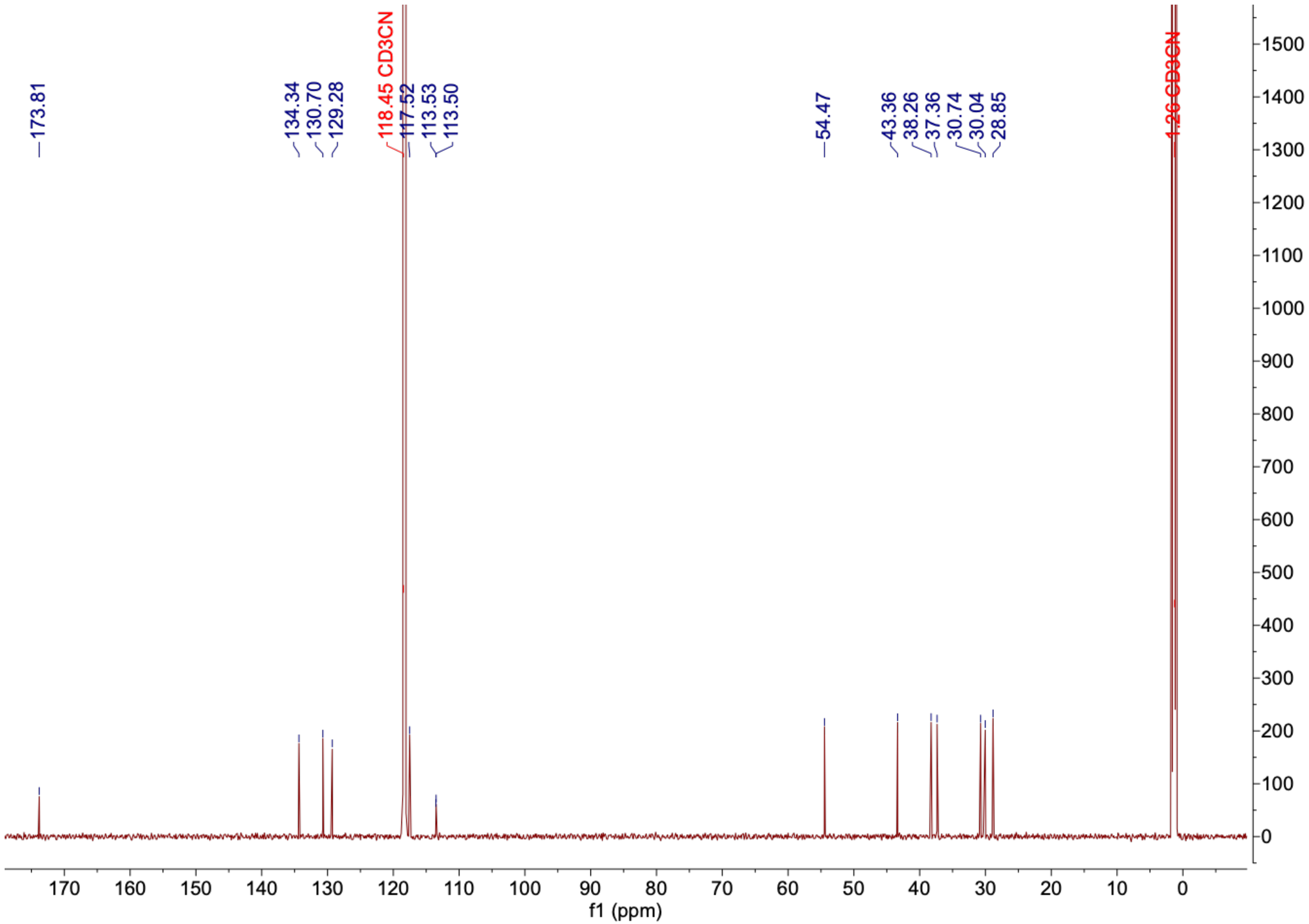

Figure S109: ^{13}C-NMR (CD_3CN) of Compound 64.

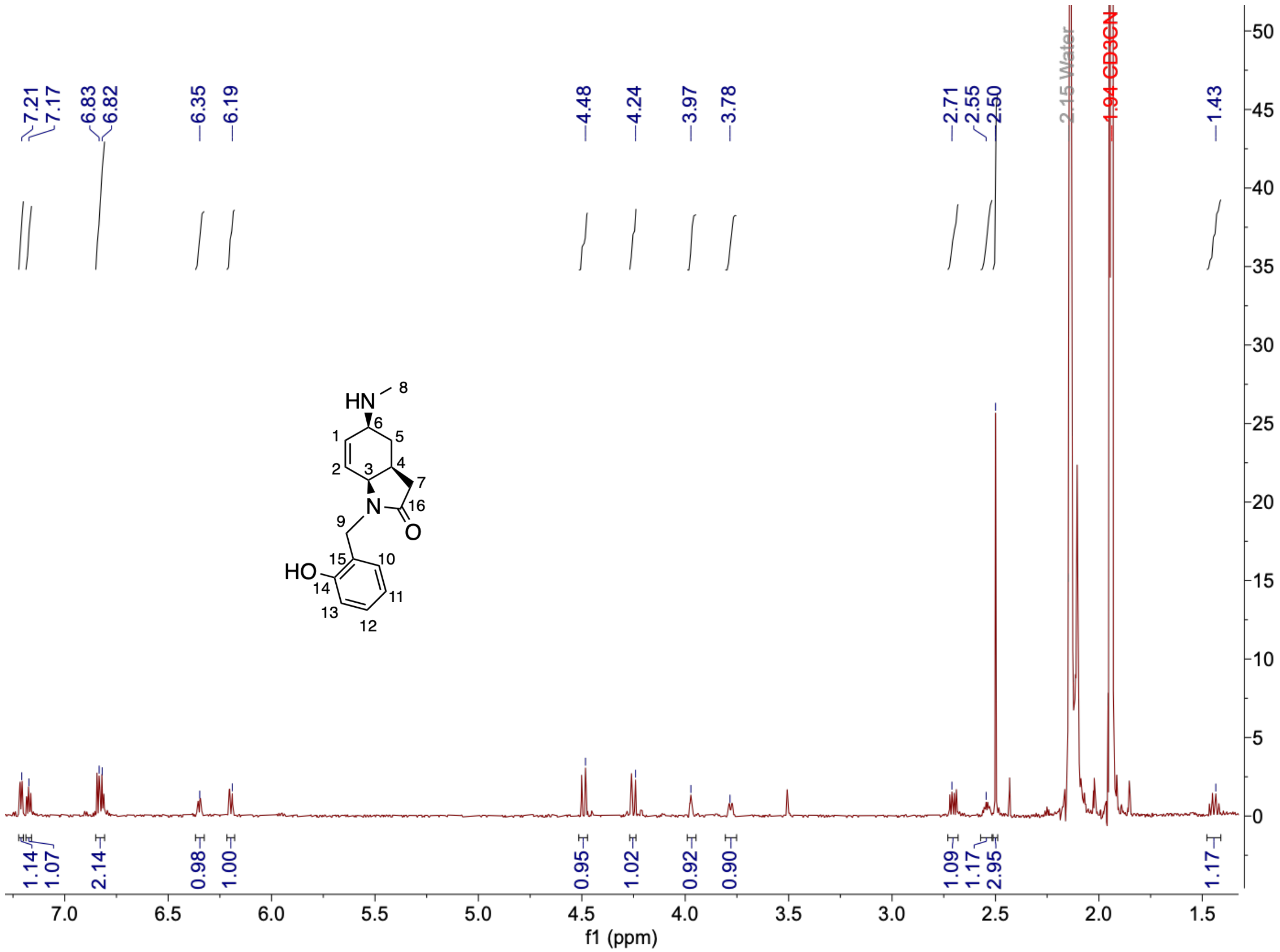

Figure S110: ^{1}H-NMR (CD_3CN) of Compound 65.

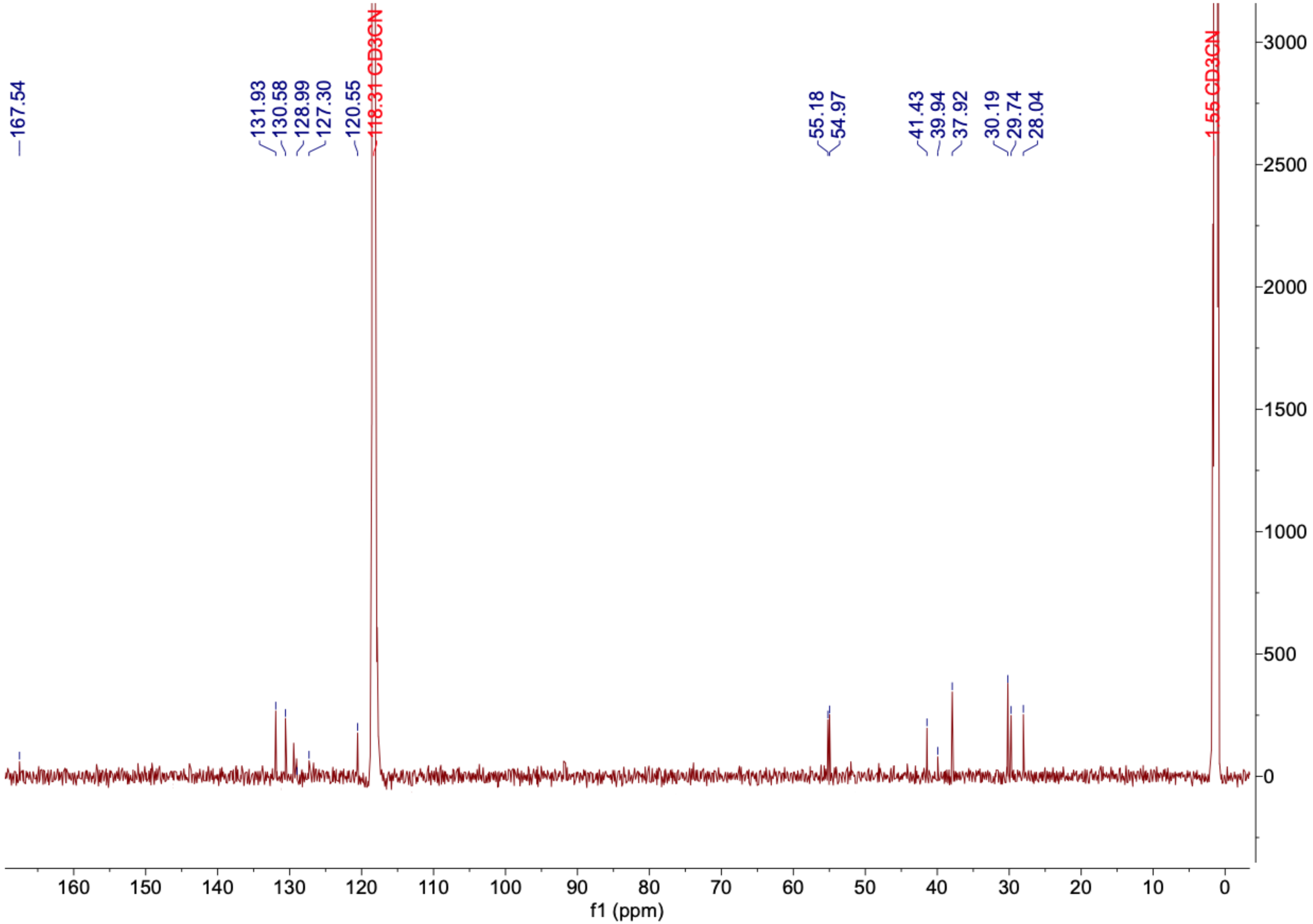

Figure S111: ^{13}C-NMR (CD_3CN) of Compound 65.

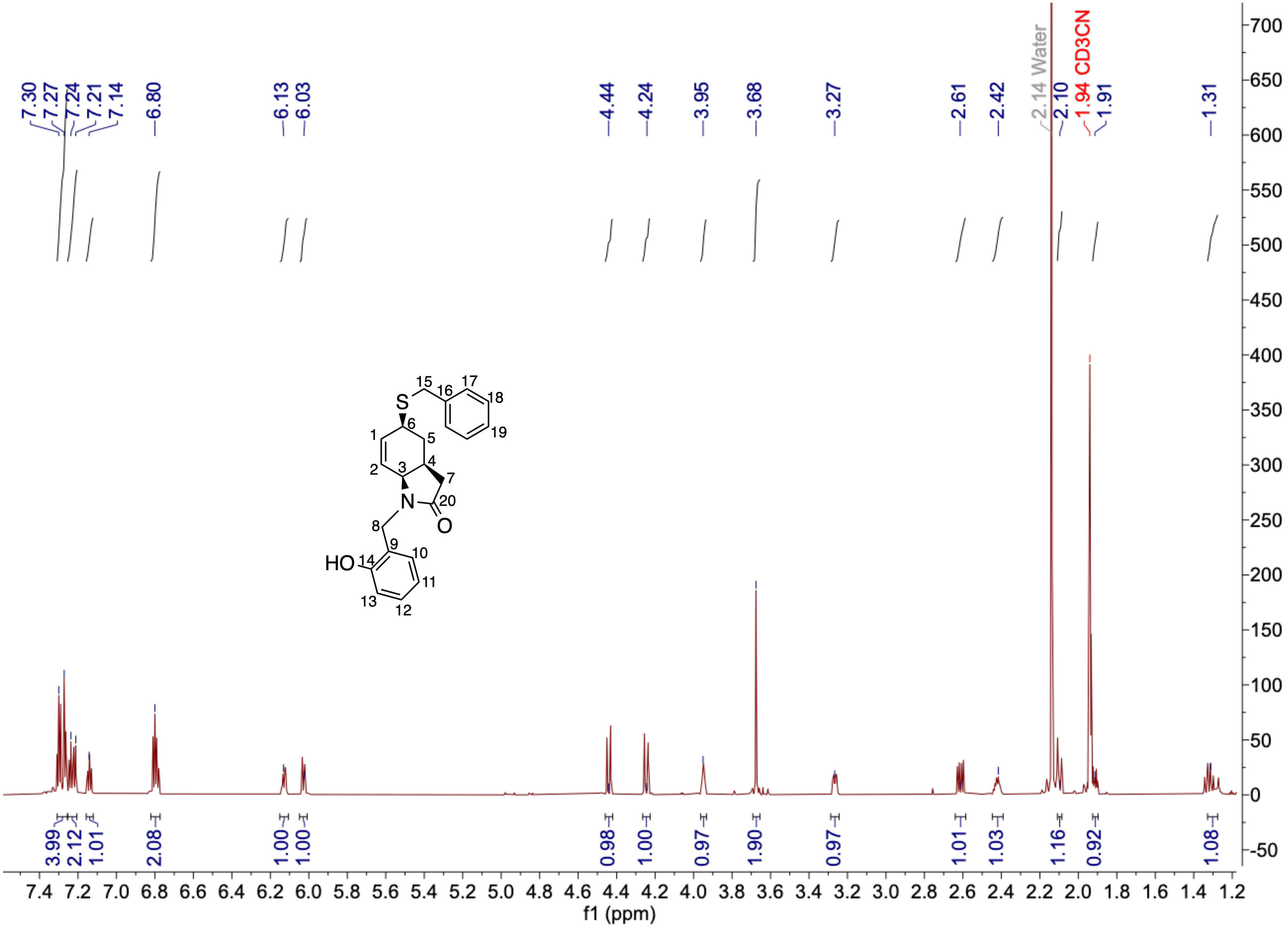

Figure S112: ^{1}H-NMR (CD_3CN) of Compound 66.

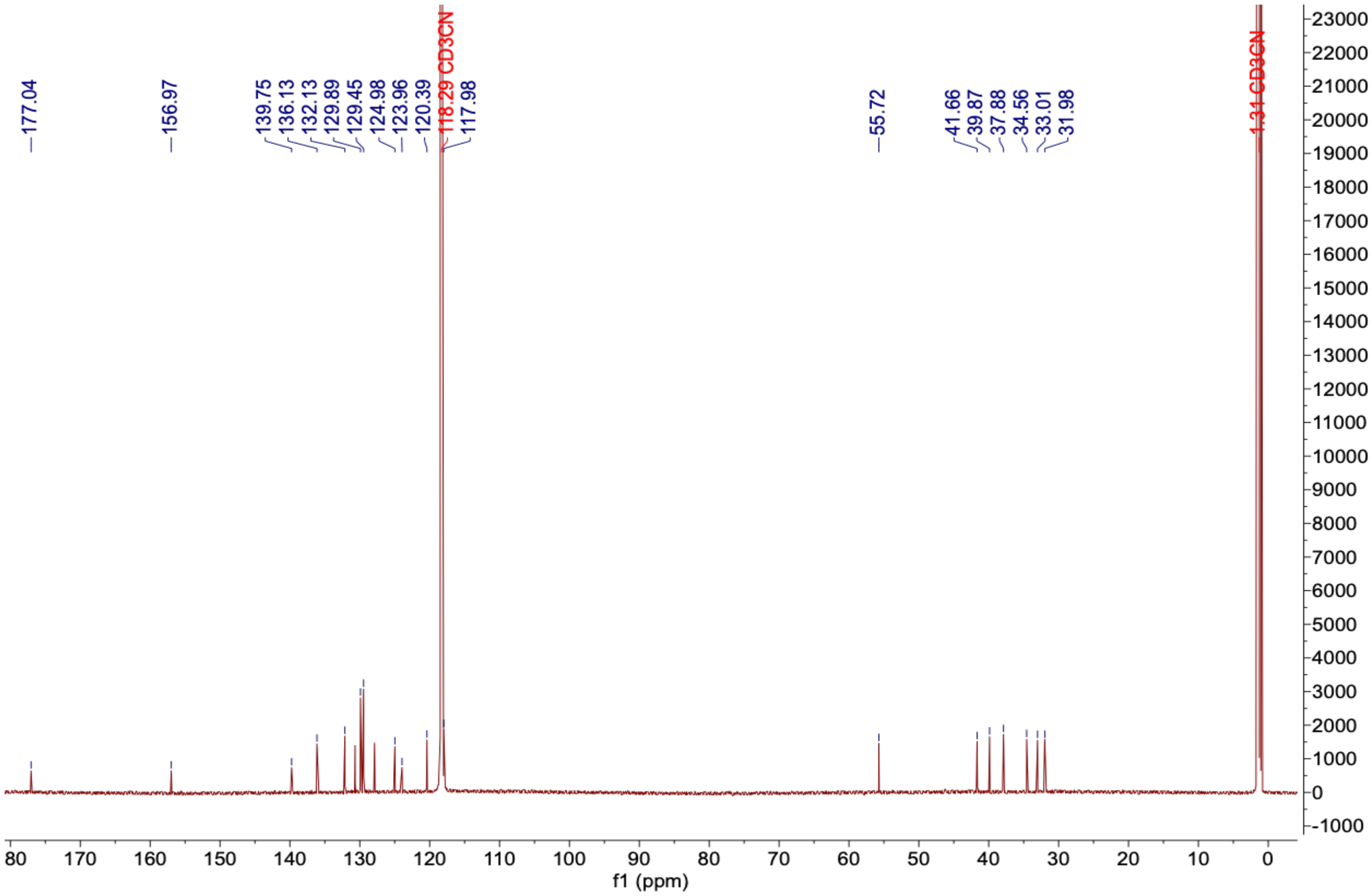

Figure S113: ^{13}C-NMR (CD_3CN) of Compound 66.

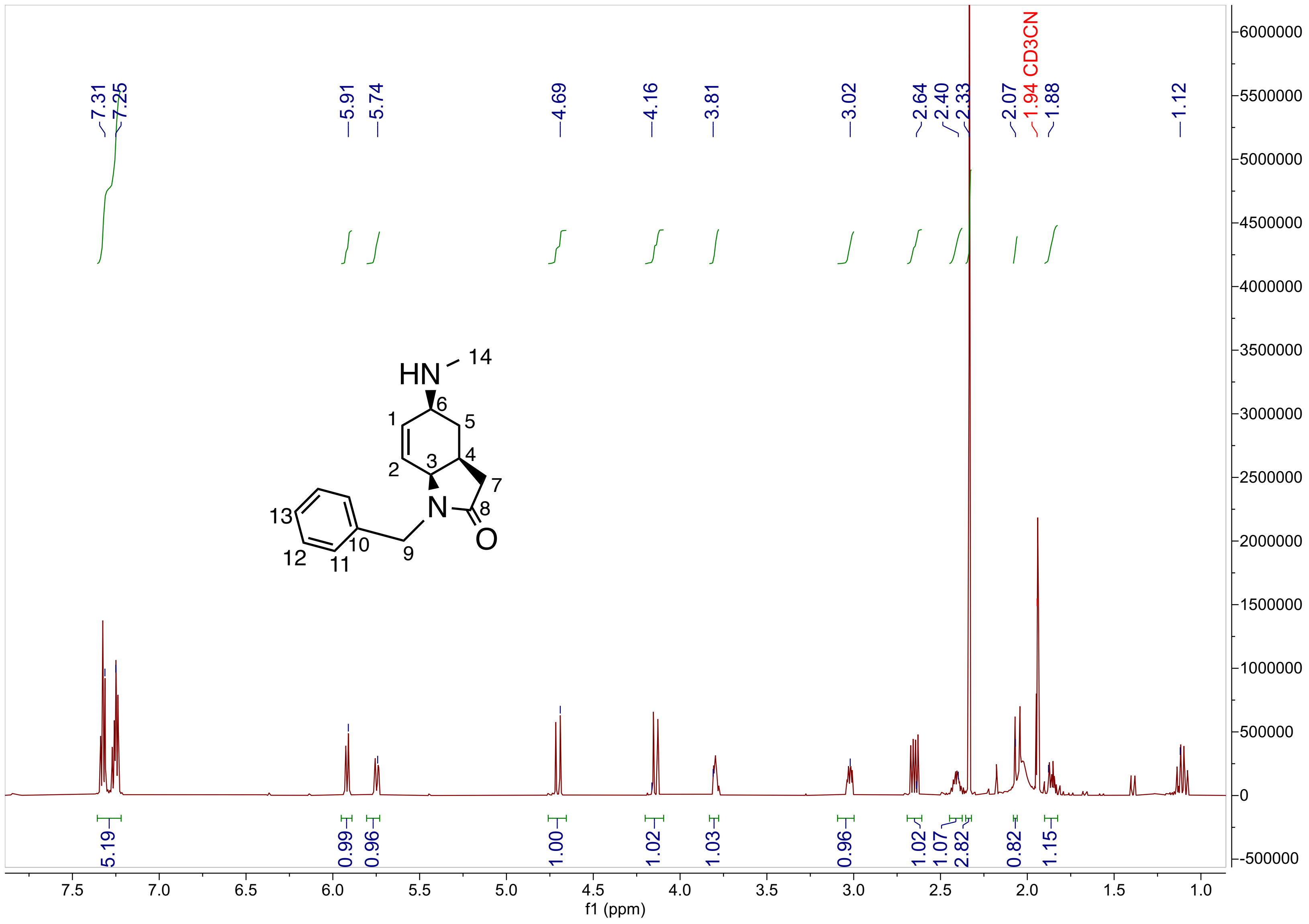

Figure S114: ^{1}H-NMR (CD_3CN) of Compound 69.

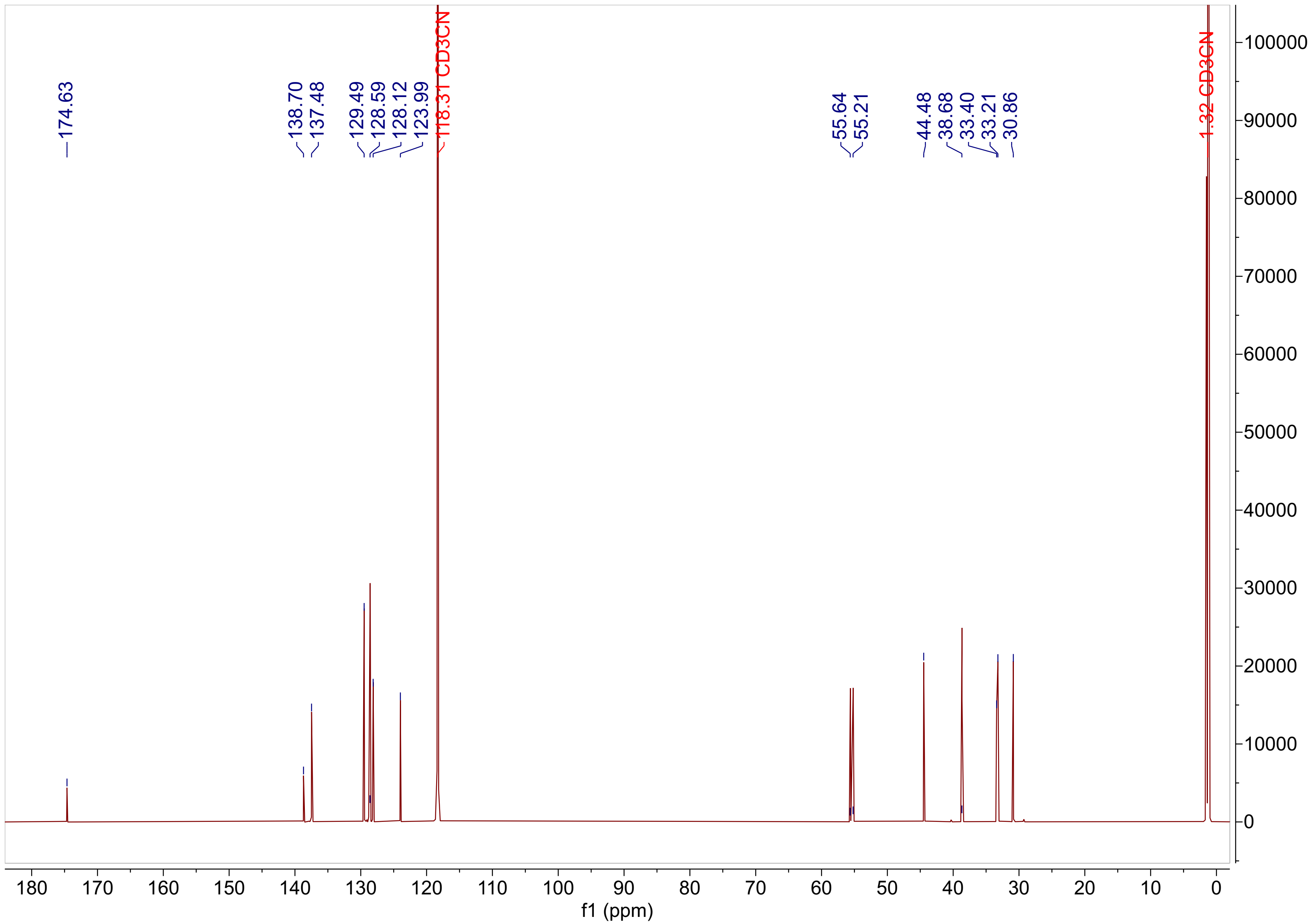

Figure S115: ^{13}C-NMR (CD_3CN) of Compound 69.

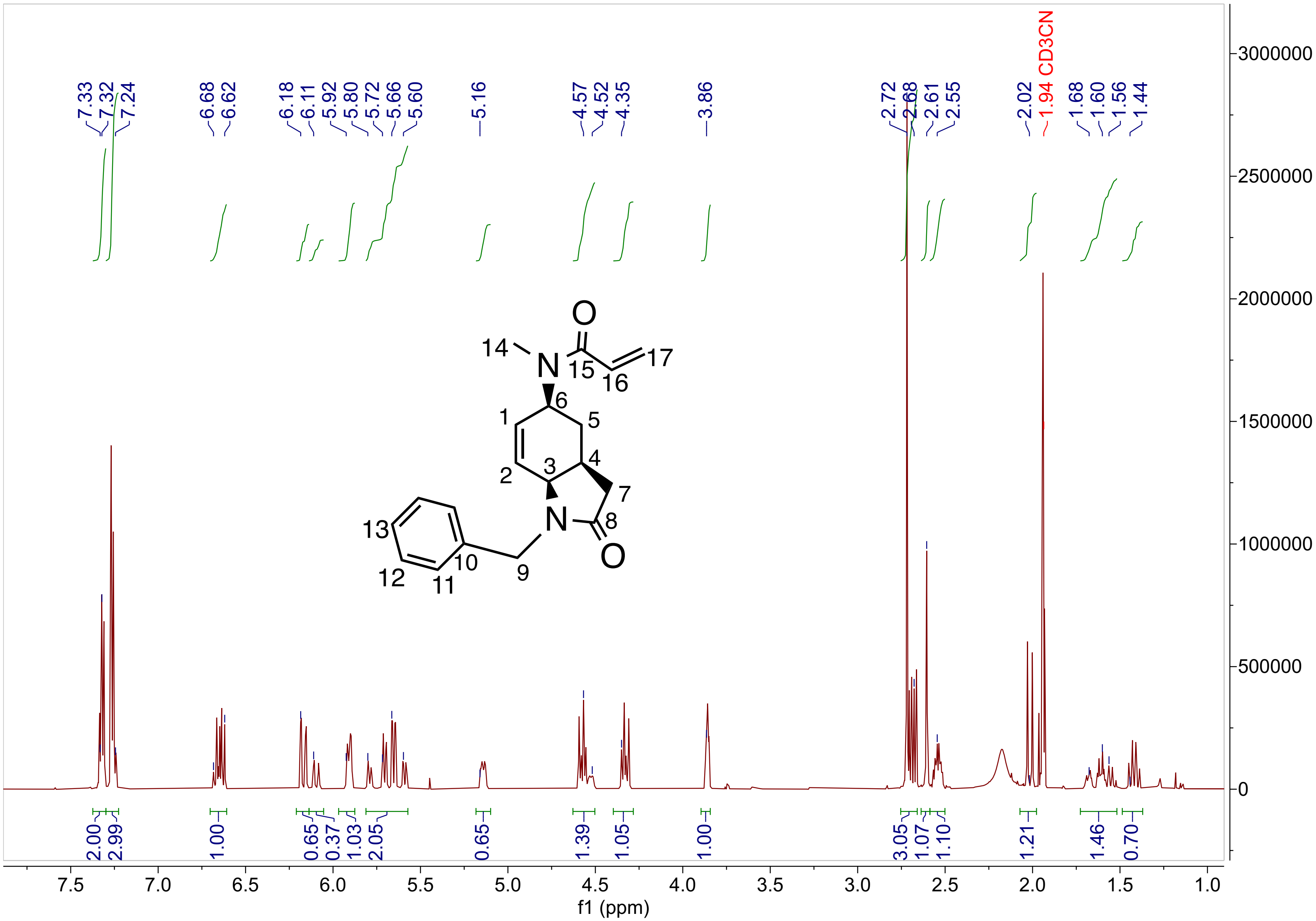

Figure S116: ^{1}H-NMR (CD_3CN) of Compound 70.

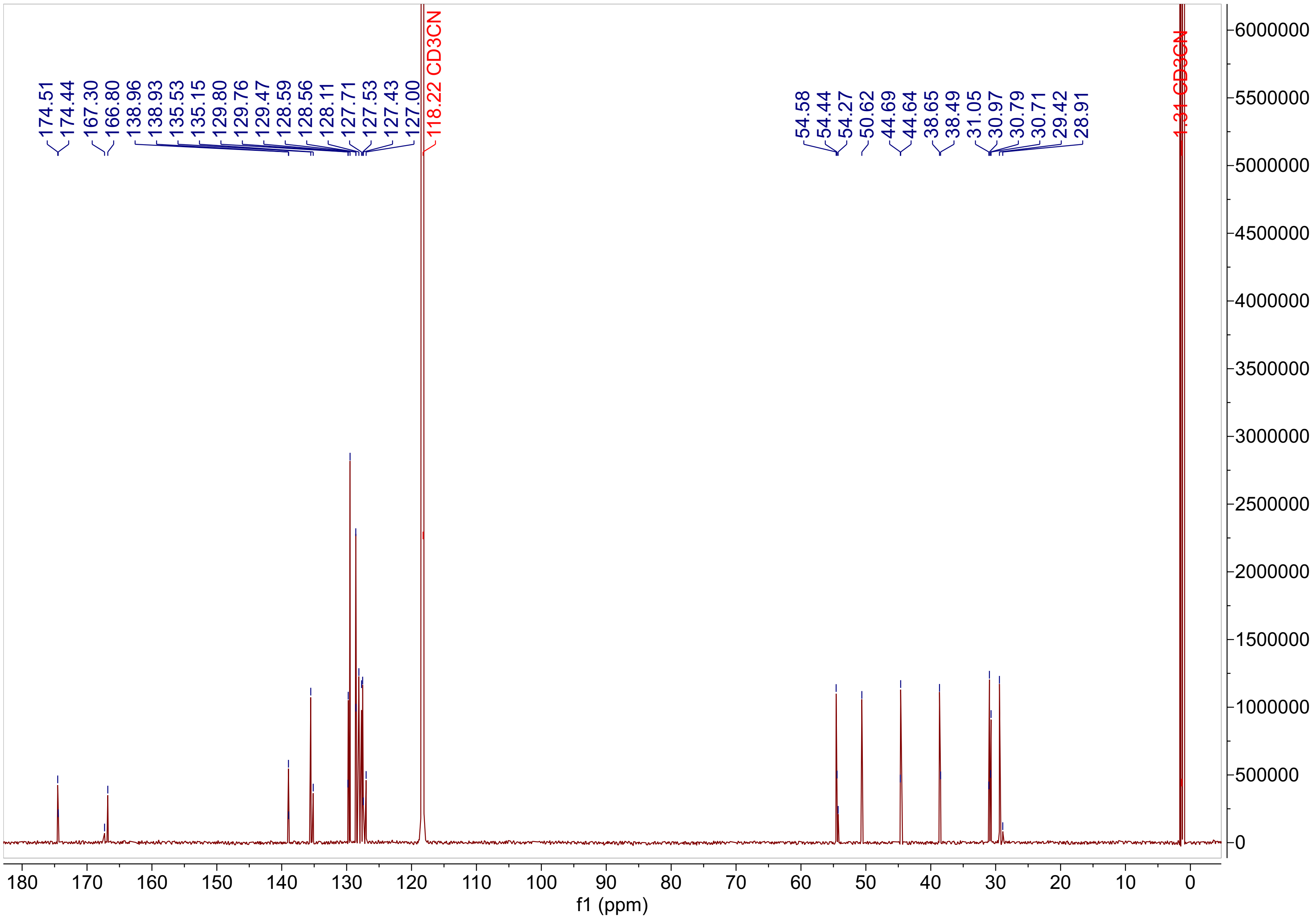

Figure S117: ^{13}C-NMR (CD_3CN) of Compound 70.

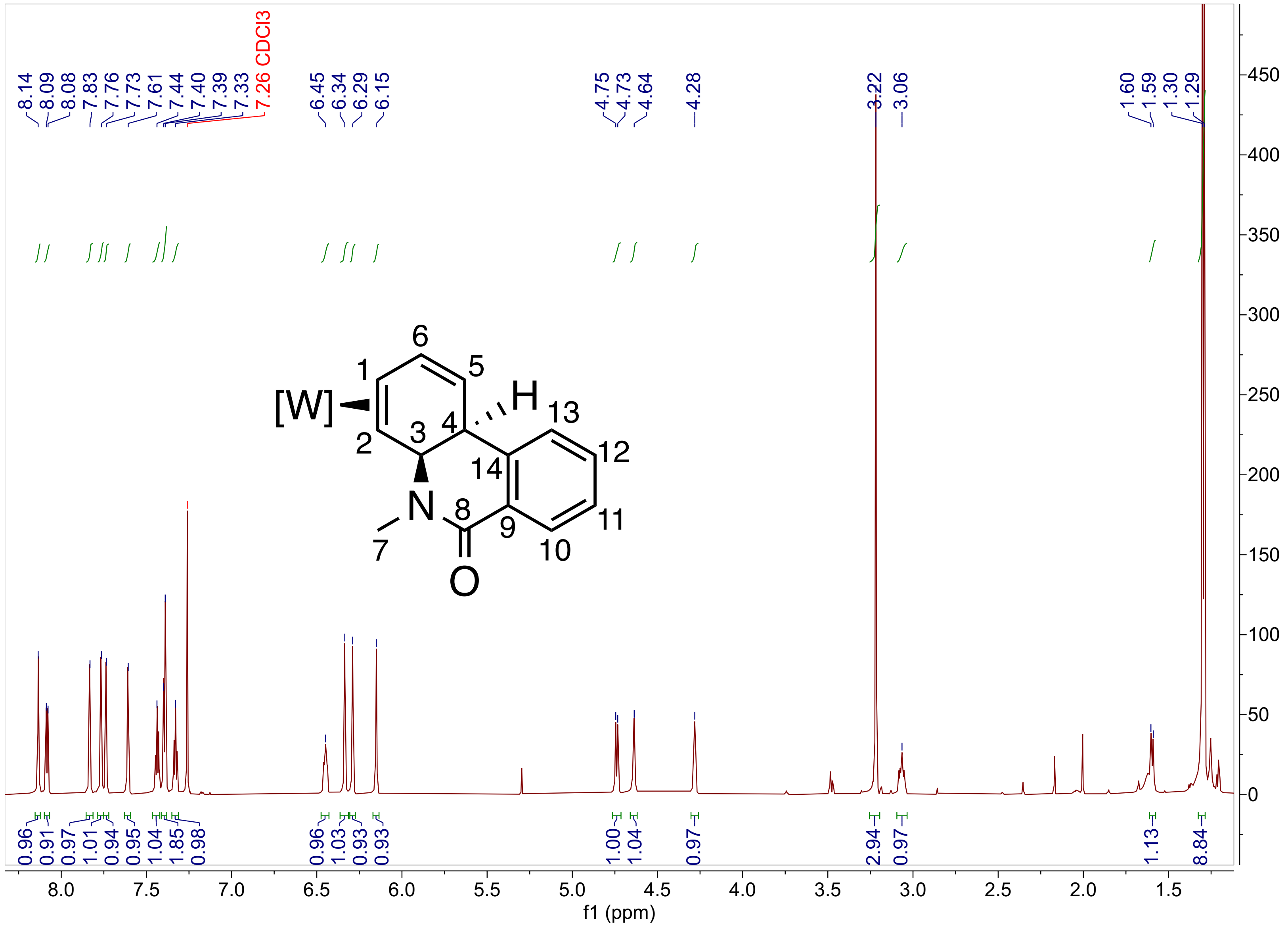

Figure S118: ^{1}H-NMR ($CDCl_3$) of Compound 72.

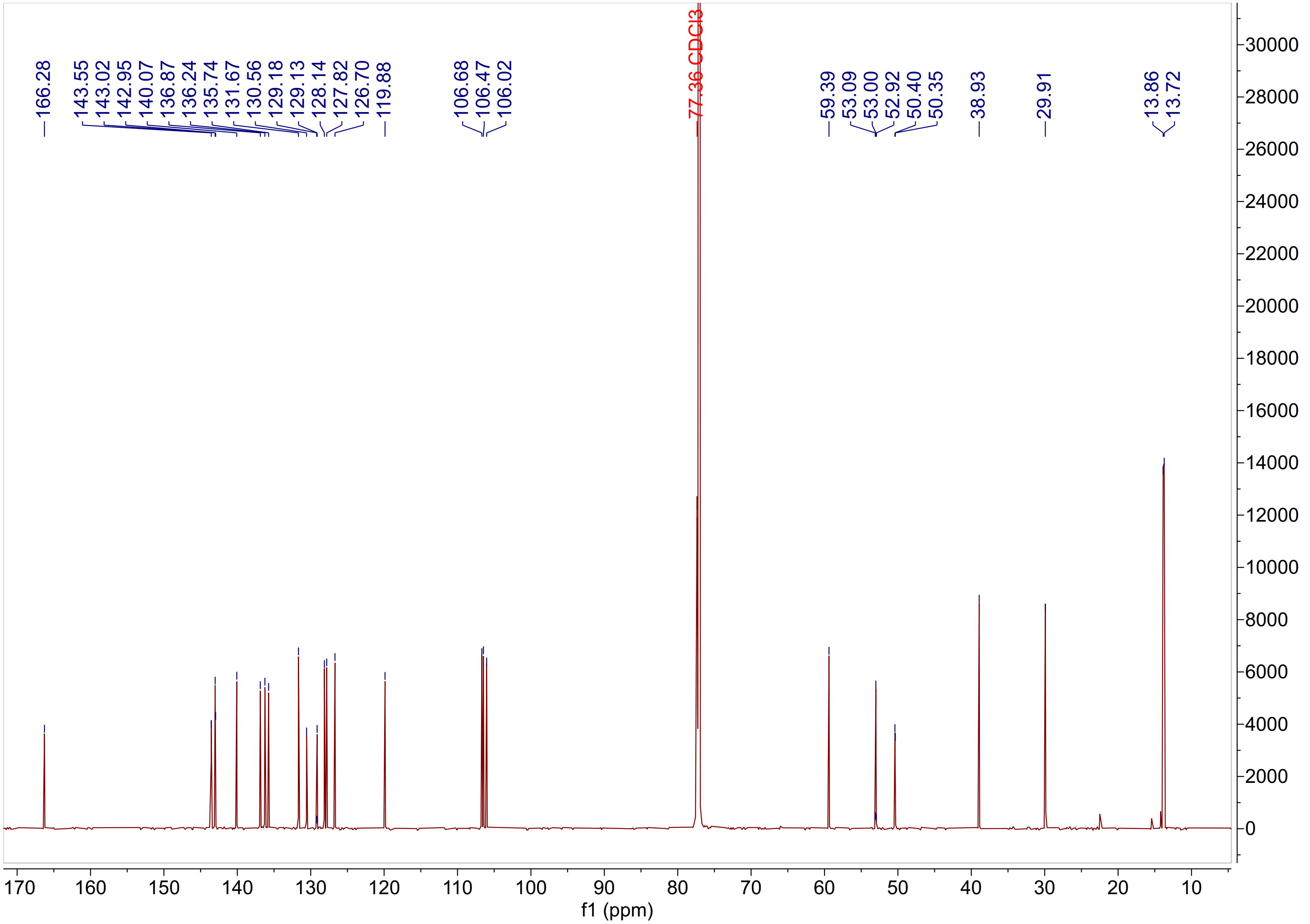

Figure S119: ^{13}C-NMR ($CDCl_3$) of Compound 72.

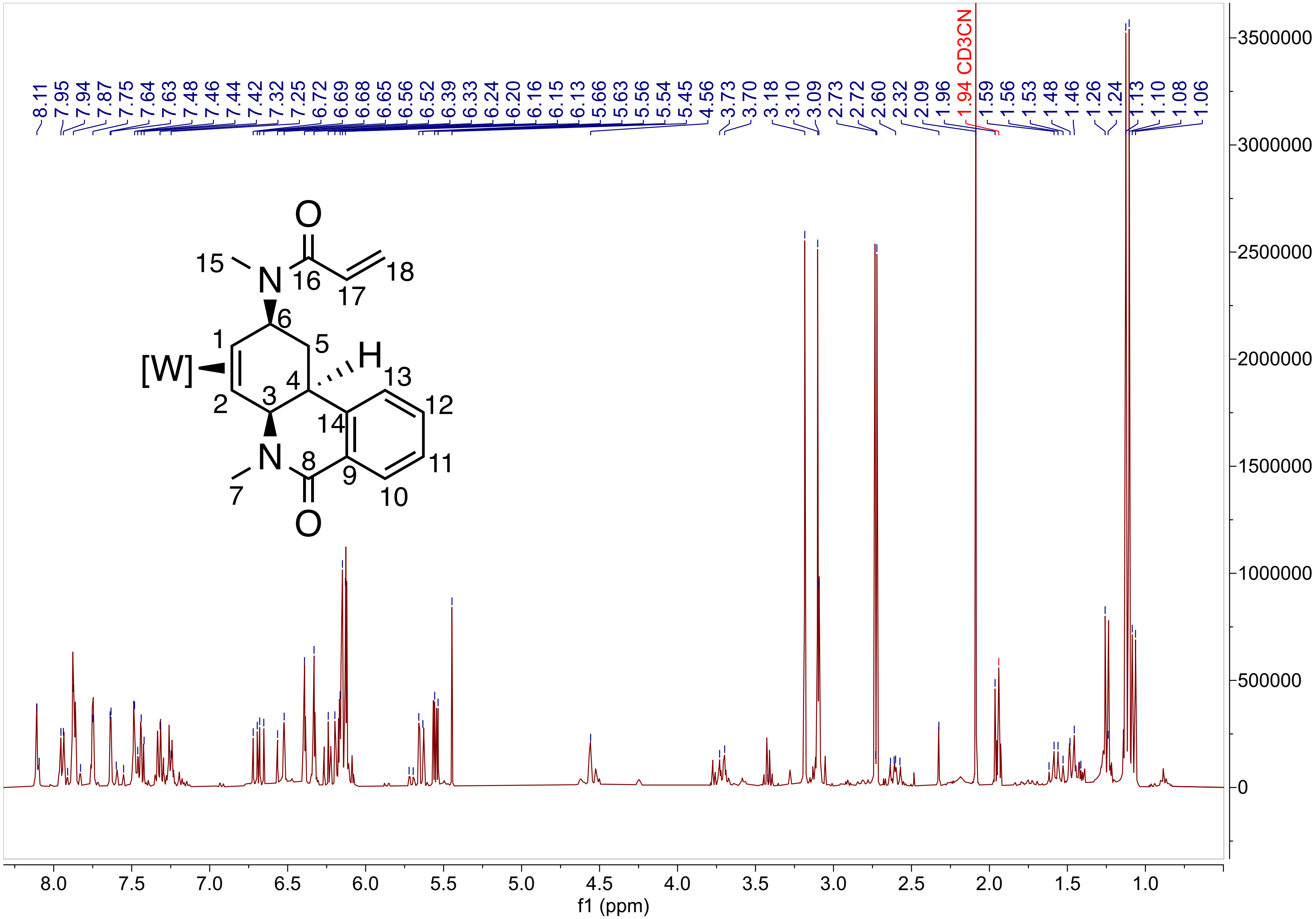

Figure S120: ^{1}H-NMR (CD_3CN) of Compound 74. NMR integrations omitted. Sample was carried forward to compound 75 without further characterization.

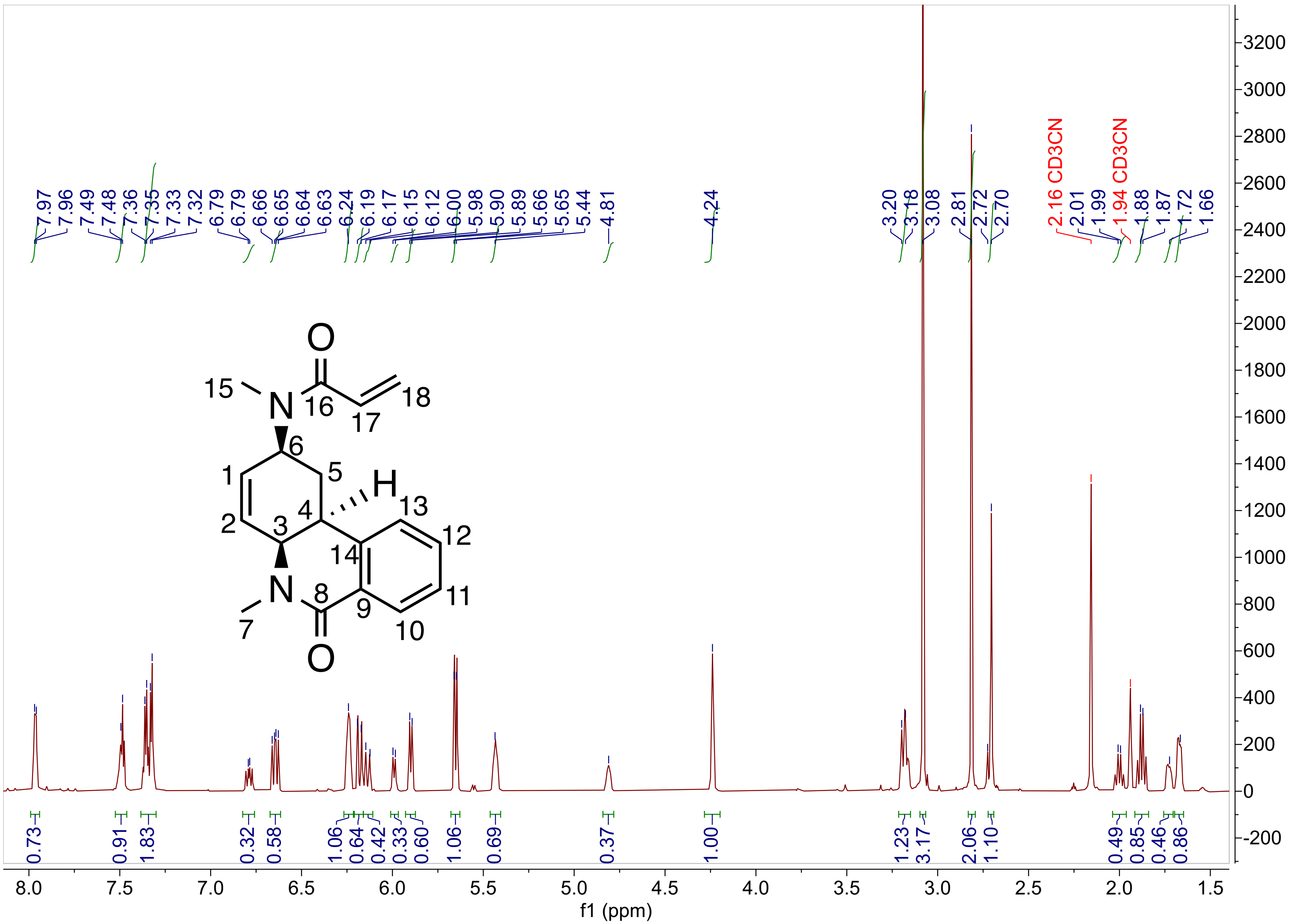

Figure S121: ^{1}H-NMR (CD_3CN) of Compound 75.

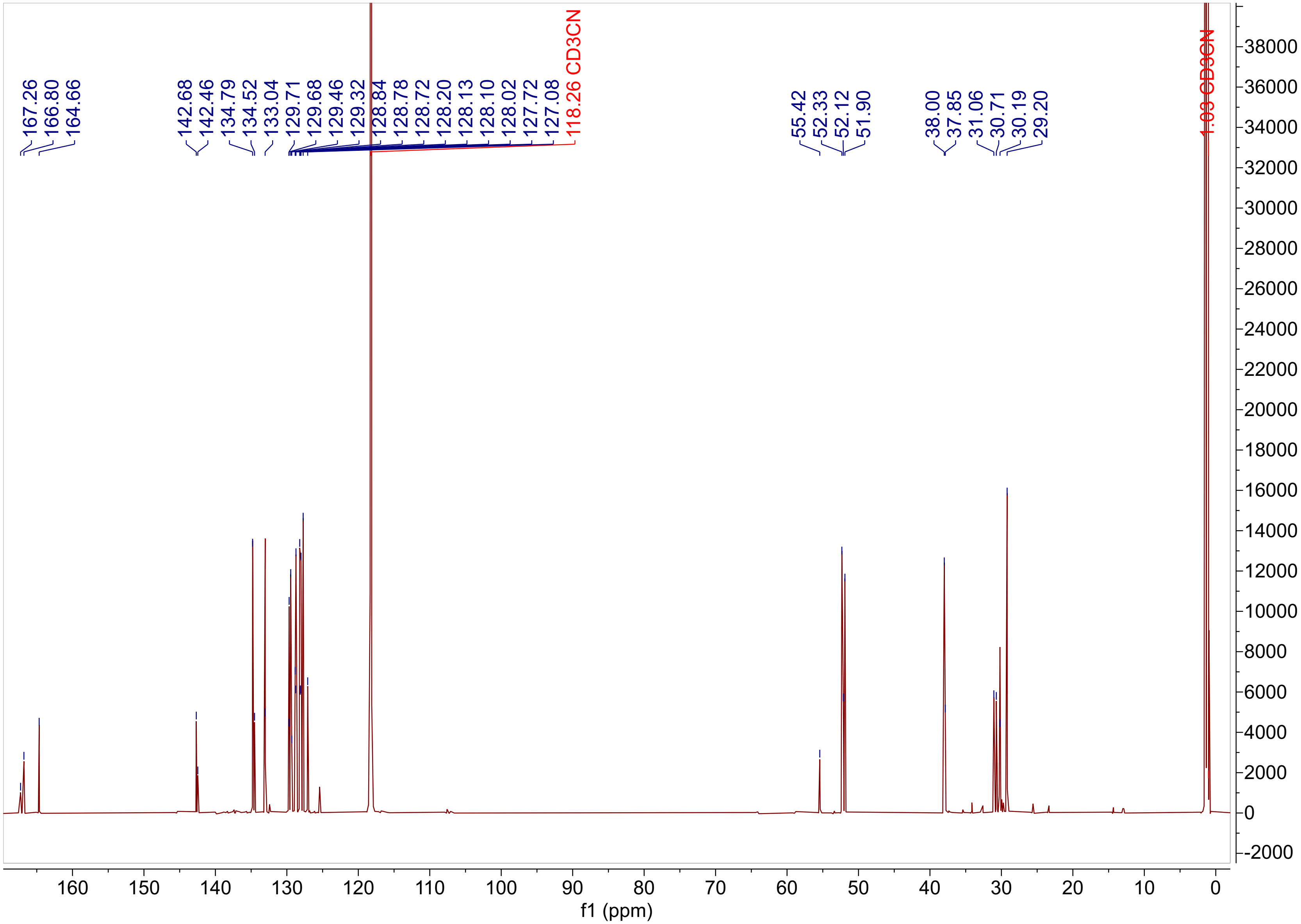

Figure S122: ^{13}C-NMR (CD_3CN) of Compound 75.

Supplementary Methods:

NMR spectra were obtained on 400, 600, or 800 MHz spectrometers. Chemical shifts are referenced to tetramethylsilane (TMS) utilizing residual ^{1}H signals of the deuterated solvents as internal standards. S46 Chemical Shifts are reported in ppm and coupling constants (J) are reported in hertz (Hz). Infrared spectra (IR) were recorded as a solid on a spectrometer with an ATR crystal accessory, and peaks are reported in cm^{-1}. Electrochemical experiments were performed under a nitrogen atmosphere. Most cyclic voltammetric data were recorded at ambient temperature at 100 mV/ s, unless otherwise noted, with a standard three-electrode cell from +1.8 to −1.8 V with a platinum working electrode, acetonitrile or N,N-dimethylacetamide (DMA) solvent, and tetrabutylammonium hexafluorophosphate (TBAH) electrolyte (~1.0 M). All potentials are reported versus the normal hydrogen electrode (NHE) using cobaltocenium hexafluorophosphate (E1/2 = −0.78 V, −1.75 V) or ferrocene (E1/2 = 0.55 V) as an internal standard. The peak separation of all reversible couples was less than 100 mV. All synthetic reactions were performed in a glovebox under a dry nitrogen atmosphere unless otherwise noted. All solvents were purged with nitrogen prior to use. Deuterated solvents were used as received from Cambridge Isotopes and were purged with nitrogen under an inert atmosphere. When possible, pyrazole protons of the tris(pyrazolyl)borate (Tp) ligand were uniquely assigned (e.g., "Tp3B") using two-dimensional NMR data. If unambiguous assignments were not possible, Tp protons were labeled as "Tp3/5 or Tp4". All J values for Tp protons are 2(±0.4) Hz. BH peaks (around 4−5 ppm) in the ^{1}H NMR spectra are not assigned due to their quadrupole broadening; However, confirmation of the BH group is provided by IR data (ca 2500 cm^{-1}). Compounds **1**, **2, 3a, 3b, 3c, 10, 11** and **72** have been previously reported[1,2]

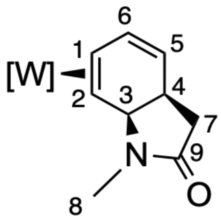

Compound 9:

Compound **3a** (150 mg, 0.204 mmol) was placed in a test tube, with ACN (2 mL), and chilled to -30 °C. After 10 min, a 1 M HOTf/ACN (0.409 mL, 0.409 mmol) solution was added to the test tube and the solution was allowed to stir at -30 °C for 10 min. In a separate test tube, methylamine (1.02 mL, 2.04 mmol) was cooled at -30 °C for 10 min. After the time elapsed, the former solution was added to the latter, dropwise. The reaction stirred at cold temperatures for 24 h and subsequently at room temperature for 19 h. It was then washed three times (H_2O:Na_2CO_3/DCM; 30 mL/30mL) and dried over anhydrous $MgSO_4$. The clear solution was evaporated in vacuo. The resulting film was dissolved in minimal DCM and pipetted in 30 mL of stirring hexane. A tan/white solid precipitated out and was collected on a 15 mL fine-porosity fritted disk, washed with hexane (2 × 10 mL) and desiccated overnight to yield compound **9** (100 mg, 0.153 mmol, 75.1%).

^{1}H-NMR (800 MHz, CD_3CN, δ, 25 C): 8.06 (2H, d, TpB3/C5), 7.86 (2H, d, TpB5/A3), 7.77 (1H, d, TpA5), 7.50 (1H, d, TpC3), 6.43 (1H, m, H6) 6.37 (1H, t, TA4), 6.30 (1H, t, TpB4) 6.27 (1H, t, TpC4), 4.55 (1H, d, H3), 4.54 (1H, d, H5), 3.21 (1H, m, H4), 2.81 (1H, m, H1), 2.61 (3H, s, H8), 2.59 (1H, ddd J= 16.18 Hz, H7), 1.89 (1H, ddd, H7), 1.33 (1H, d, H2), 1.19 (d, J = 8.5 Hz, 9H, PMe_3). **^{13}C-NMR (201 MHz, CD_3CN, δ, 25 ºC):** 175.4 (1C, C9), 145.5 (1C, TpB3), 144.6 (1C, TpC5), 137.9 (2C, TpCA/B5), 137.4 (1C, TpA5), 132.0 (1C, C6), 107.6 (1C, TpA4), 107.3 (1C, Tp4C) 106.8 (1C, Tp4B), 106.89 (1C, Tp4A), 64.3 (1C, C3), 58.9 (1C, C2), 49.2 (1C, C1), 48.3 (1C, C5), 40.3 (1C, C7) 32.9 (1C, C4), 27.8 (1C, C8), 13.6 (3C, d J= 29.30 Hz, PMe3). **CV (DMA):** $E_{p,a}$= 0.84 V (NHE).

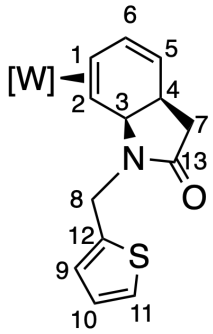

Compound 12:

Compound **3a** (100mg, 0.136 mmol) was placed in a test tube with ACN (2 mL), and chilled to -30 °C. After 10 min, a 1 M HOTf/ACN (0.341 mL, 0.341 mmol) solution was added to the test tube and the solution was allowed to stir at -30 °C for 30 min. In a separate test tube, 2-Thiophenemethylamine (0.140 mL, 1.360 mmol) with ACN (2 mL) was cooled at -30 °C for 20 min. After the time elapsed, the former solution was added to the latter, dropwise. The reaction stirred at -30 °C for 48 h and room temperature for 24 h. The reaction was washed three times (H_2O:Na_2CO_3/DCM; 30 mL/30mL) and dried over anhydrous Na_2SO_4. The organic layer was evaporated in vacuo. The resulting yellow film was dissolved in minimal DCM and pipetted in 25 mL of stirring hexane. A white solid precipitated out and was collected on a 15 mL fine-porosity fritted disk, washed with hexane (2 × 10 mL) and desiccated overnight to yield compound **12** (51 mg, 0.069 mmol, 52%).

^{1}H NMR (800 MHz, $(CD_3)_2CO$, δ, 25 C): 8.13 (s, 1H, TpB3), 7.97 (d, *J* = 2.3 Hz, 1H, TpC5), 7.94 (m, 1H, TpB5), 7.88 (s, 1H, TpA3), 7.79 (m, 1H, TpA5), 7.60 (d, *J* = 2.1 Hz, 1H, TpC3), 7.16 (dt, *J* = 5.1, 1.1 Hz, 1H, H11), 6.75 (ddd, *J* = 5.1, 3.4, 1.0 Hz, 1H, H10), 6.52 (dt, *J* = 3.4, 1.0 Hz, 1H, H9), 6.41 (d, *J* = 7.8 Hz, 1H, H6), 6.40 (tt, *J* = 2.2, 1.0 Hz, 1H, TpB4), 6.36 (td, *J* = 2.2, 0.9 Hz, 1H, TpC4), 6.15 (dt, *J* = 2.3, 1.1 Hz, 1H, TpA4), 4.81 (d, *J* = 15.3 Hz, 1H, H8A), 4.68 (d, *J* = 6.3 Hz, 1H, H3), 4.53 (d, *J* = 9.9 Hz, 1H, H5), 4.38 (d, *J* = 15.4 Hz, 1H, H8B), 3.31 (t, *J* = 8.2 Hz, 1H, H4), 2.78 (m, 1H, H1), 2.57 (dd, *J* = 16.0, 8.2 Hz, 1H, H7A), 1.94 (d, *J* = 16.1 Hz, 1H, H7B), 1.52 (d, *J* = 9.6 Hz, 1H, H2), 1.25 (d, *J* = 8.6 Hz, 9H, PMe_3). **^{13}C NMR (201 MHz, $(CD_3)_2CO$, δ, 25 C):** 174.8 (1C, C13), 144.5 (1C, TpB3), 143.5 (1C, TpB5), 142.0 (1C, TpC3), 137.8 (1C, TpC5), 137.1 (1C, TpA5), 136.7 (1C, TpA3), 131.9 (d, *J* = 3.7 Hz, 1C, C6), 127.2 (1C, C10), 126.3 (1C, C9), 125.1 (1C, C11), 120.3 (1C, C5), 107.2 (1C, TpB4), 107.0 (1C, TpC4), 106.4 (1C, TpA4), 62.2 (1C, C3), 48.1 (1C, C2), 40.2 (1C, C7), 38.4 (1C, C8), 32.8 (1C, C4), 13.7 (d, *J* = 28.3 Hz, 1C, PMe_3)

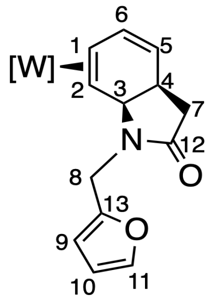

Compound 13:

Compound **3a** (100mg, 0.136 mmol) was placed in a test tube with ACN (2 mL), and chilled to -30 °C. After 10 min, a 1 M HOTf/ACN (0.341 mL, 0.341 mmol) solution was added to the test tube and the solution was allowed to stir at -30 °C for 30 min. In a separate test tube, Furfurylamine (0.120 mL, 1.364 mmol) with ACN (2 mL) was cooled at -30 °C for 20 min. After the time elapsed, the former solution was added to the latter, dropwise. The reaction stirred at -30 °C for 48 h and room temperature for 24 h. The reaction was washed three times (H_2O:Na_2CO_3/DCM; 30 mL/30mL) and dried over anhydrous Na_2SO_4. The organic layer was evaporated in vacuo. The resulting yellow film was dissolved in minimal DCM and pipetted in 25 mL of stirring hexane. A

white solid precipitated out and was collected on a 15 mL fine-porosity fitted disk, washed with hexane (2 × 10 mL) and desiccated overnight to yield compound **13** (52.0 mg, 0.072 mmol, 53.0%).

^{1}H NMR (800 MHz, CD_2Cl_2, δ, 25 C): 8.05 (d, *J* = 2.0 Hz, 1H, TpB3), 7.86 (d, *J* = 2.0 Hz, 1H, TpA5), 7.78 (d, *J* = 2.3 Hz, 1H, TpC5), 7.76 (d, *J* = 2.4 Hz, 1H, TpB5), 7.66 (d, *J* = 2.4 Hz, 1H, TpA3), 7.35 (d, *J* = 2.2 Hz, 1H, TpC3), 7.15 (m, 1H, H11), 6.44 (ddd, *J* = 9.9, 5.1, 2.5 Hz, 1H, H6), 6.34 (m, 1H, TpB4), 6.27 (t, *J* = 2.1 Hz, 1H, TpC4), 6.17 (t, *J* = 2.3 Hz, 1H, TpA4), 6.15 (t, *J* = 2.6 Hz, 1H, H10), 5.83 (d, *J* = 3.2 Hz, 1H, H9), 4.64 (m, 3H, H3/H5/H8A), 4.21 (d, *J* = 15.7 Hz, 1H, H8B), 3.26 (t, *J* = 7.6 Hz, 1H, H4), 2.67 (m, 2H, H1/H7A), 2.05 (d, *J* = 16.3 Hz, 1H, H7B), 1.20 (d, *J* = 8.4 Hz, 9H, PMe_3). **^{13}C NMR (201 MHz, CD_2Cl_2, δ, 25 C):** 175.4 (1C, C13), 152.3 (1C, C12), 143.8 (1C, TpB3), 143.0 (1C, TpA5), 141.7 (1C, C11), 140.9 (1C, TpC3), 137.1 (1C, TpC5, 136.4 (1C, TpA3), 136.1 (1C, TpB5), 131.2 (d, *J* = 3.8 Hz, 1C, C6), 120.1 (1C, C5), 110.4 (1C, C10), 107.3 (1C, C9), 106.8 (1C, TpB4), 106.4 (1C, TpC4), 105.9 (1C, TpA4), 62.6 (1C, C3), 49.2 (d, *J* = 9.3 Hz, 1C, C1), 47.6 (d, *J* = 15.2 Hz, 1C, C2), 40.1 (1C, C7), 36.8 (1C, C8), 32.3 (1C, C4), 13.8 (d, *J* = 28.2 Hz, 1C, PMe_3).

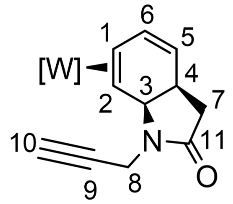

Compound 14:
Compound **3a** (100 mg, 0.136 mmol) was placed in a test tube, with ACN (2 mL), and chilled to -30 °C. After 10 min, a 1 M HOTf/ACN (0.272 mL, 0.272 mmol) solution was added to the test tube and the solution was allowed to stir at -30 °C for 10 min. In a separate test tube, propargylamine (0.088 mL, 1.36 mmol) with ACN (2 mL) was cooled at -30 °C for 10 min. After the time elapsed, the former solution was added to the latter, dropwise. After the time elapsed, the former solution was added to the latter, dropwise. The reaction stirred at -30 °C for 12 h then stirred at room temperature for 24 h. The reaction was washed three times (H_2O:Na_2CO_3/DCM; 30 mL/30mL) and dried over anhydrous Na_2SO_4. The clear solution was evaporated in vacuo. The resulting film was dissolved in minimal DCM and pipetted in 15 mL of stirring hexane. A white solid precipitated out and was collected on a 15 mL fine-porosity fritted disk, washed with hexane (2 × 10 mL) and desiccated overnight to yield compound **14** (76 mg, 0.11 mmol, 82%).

^{1}H-NMR (800 MHz $(CD_3)_2CO$), δ, 25°C): 8.22 (1H, d, TpA3), 8.17 (1H, d, TpB3), 7.96 (2H, t, TpB5/C5), 7.81 (1H, d, TpA5), 7.66 (1H, d, TpC3), 6.43 (2H, m, H6/TpB4), 6.34 (1H, t, TC4), 6.28 (1H, t, TpA4), 4.87 (1H, d, H3), 4.51 (1H, dd, H5), 4.40 (1H, m, H8), 3.72 (1H, dd, H8), 3.38 (1H, t, H4), 2.85 (1H, m, H1), 2.62 (1H, t, H7), 2.33 (1H, t, H10), 1.93 (1H, d, H7), 1.57 (1H, d, H2), 1.26 (9H, d, PMe_3).**^{13}C-NMR (201 MHz $(CD_3)_2CO$), δ, 25°C):** 174.7 (1C, C11). 144.5 (1C, TpA3) 143.9 (1C, TpB3), 142.0 (1C, TpC5), 137.8 (1C, TpB5), 137.0 (1C, TpA5), 136.8 (1C, TpC3), 132.1 (1C, C6), 120.1 (1C, C5), 107.3 (1C, TpB4), 107.0 (1C, TpC4), 106.3 (1C, TpA4), 80.2 (1C,

C9), 72.4 (1C, C10), 62.7 (1C, C3), 49.7 (1C, d, C1), 46.8 (1C, C2), 40.3 (1C, s, C7), 32.7 (1C, s, C4), 29.1 (1C, C8), 13.5 (3C, d, PMe_3).

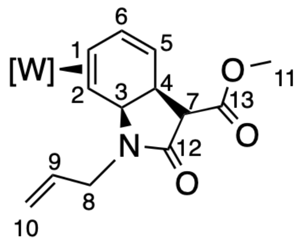

Compound 15:

Compound **3c** (177 mg, 0.223 mmol) was placed in a test tube, with ACN (2 mL), and chilled to -30 °C. After 10 min, a 1 M HOTf/ACN (0.447 mL, 0.447 mmol) solution was added to the test tube and the solution was allowed to stir at -30 °C for 20 min. In a separate test tube, allylamine (0.167 mL, 2.023 mmol) with ACN (2 mL) was cooled at -30 °C for 20 min. After the time elapsed, the former solution was added to the latter, dropwise. The reaction stirred at -30 °C for 12 h then stirred at room temperature for 24 h. The reaction was washed three times (H_2O:Na_2CO_3/DCM; 30 mL/30mL) and dried over anhydrous Na_2SO_4. The clear solution was evaporated in vacuo. The resulting film was dissolved in minimal DCM and pipetted in 15 mL of stirring hexane. A white solid precipitated out and was collected on a 15 mL fine-porosity fritted disk, washed with hexane (2 × 10 mL) and desiccated overnight to yield compound **15** (107 mg, 73%).

^{1}H-NMR (800 MHz, CD_3CN, δ, 25°C): 8.05 (1H, d, Tp3B), 7.93 (1H, d, Tp3A), 7.86 (2H, d, Tp5C/Tp5B), 7.77 (1H, d, Tp5A), 7.47 (1H, d, Tp3C), 6.48 (1H, m, H6), 6.38 (1H, t, Tp4B), 6.30 (1H, t, Tp4C), 6.29 (1H, t, Tp4A), 5.57 (1H, m, H9), 5.01 (1H, d, H3), 4.96 (1H, q, H10), 4.89 (1H, q, H10), 4.53 (1H, dd, H5), 3.97 (1H, dd, H8), 3.73 (3H, s, H11), 3.72 (1H, dd, H8), 3.69 (1H, d, H4), 3.02 (1H, s, H7), 2.81 (1H, m, H1), 1.24 (1H, d, H2), 1.19 (9H, d, PMe3) . **^{13}C-NMR (201 MHz, CD_3CN, δ, 25°C):** 172.1 (1C, C13). 170.5 (1C, C12), 144.6 (1C, TpB3) 143.2 (1C, TpA3), 142.1 (1C, TpC3), 138.1 (1C, TpC5), 137.4 (2C, TpA5/TpB5), 134.4 (1C, C9), 133.1 (1C, d, C6), 120.4 (1C, C5), 116.1 (1C, C10), 107.6 (1C, TpB4), 107.3 (1C, TpC4), 106.9 (1C, TpA4), 61.8 (1C, C3), 57.9 (1C, C7), 52.9 (1C, C11), 49.0 (1C, C1), 47.6 (1C, C2), 42.5 (1C, C8), 38.2 (1C, C4), 13.7 (3C, PMe_3).

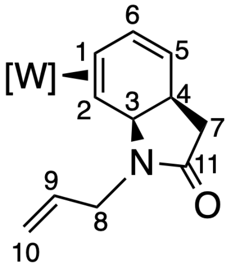

Compound 16:

Compound **3a** (150 mg, 0.2045 mmo) was placed in a test tube, with ACN (2 mL), and chilled to -30 °C. After 10 min, a 1 M HOTf/ACN (0.409 mL, 0.409 mmol solution was added to the test

tube and the solution was allowed to stir at -30 °C for 20 min. In a separate test tube, allylamine (0.153mL, 2.045 mmol) with ACN (2 mL) was cooled at -30 °C for 20 min. After the time elapsed, the former solution was added to the latter, dropwise. The reaction stirred at -30 °C 12 h then stirred at room temperature for 24 h. The reaction was washed three times (H_2O:Na_2CO_3/DCM; 30 mL/30mL) and dried over anhydrous Na_2SO_4. The clear solution was evaporated in vacuo. The resulting film was dissolved in minimal DCM and pipetted in 15 mL of stirring hexane. A white solid precipitated out and was collected on a 15 mL fine-porosity fritted disk, washed with hexane (2 × 10 mL) and desiccated overnight to yield compound **16** (59.6%).

^{1}H-NMR (800 MHz, CD_3CN, δ, 25°C): 8.06 (1H, d, Tp3B), 8.04 (1H, d, Tp3A), 7.86 (2H, d, Tp5C, Tp5B), 7.76 (1H, d, Tp5A), 7.48 (1H, d, Tp3C), 6.45 (1H, m, H6), 6.38 (1H, t, Tp4B), 6.30 (1H, t, Tp4C), 6.25 (1H, t, Tp4A), 5.60 (1H, m, H9), 4.88 (2H, td, H10), 4.74 (1H, d, H3), 4.54 (1H, dd, H5), 3.92 (1H, dt, H8), 3.69 (1H, dd, H8), 3.24 (1H, t, H4), 2.77 (1H, ddd, H1), 2.66 (1H, dd, H7), 1.92 (1H, m, H7), 1.28 (1H, d, H2), 1.18 (9H, d, PMe_3). **^{13}C-NMR (201 MHz, CD_3CN, δ, 25°C):** 175.4 (1C, s, C11). 144.6 (1C, d, Tp3B) 143.8 (1C, s, Tp3A), 142.1 (1C, s, Tp3C), 138.1/137.4 (1C, s, Tp5B/Tp5C), 137.2 (1C, s, Tp5A), 135.3 (1C, s, C9), 132.3 (1C, d, C6), 120.4 (1C, s, C5), 115.9 (1C, s, C10), 107.6 (1C, s, Tp4B), 107.2 (1C, s, Tp4C), 106.7 (1C, s, Tp4A), 62.7 (1C, s, C3), 49.7 (1C, d, C1), 48.2 (1C, s, C2), 42.4 (1C, s, C8), 40.2 (1C, s, C7), 33.2 (1C, s, C4), 13.7 (3C, d, PMe_3).

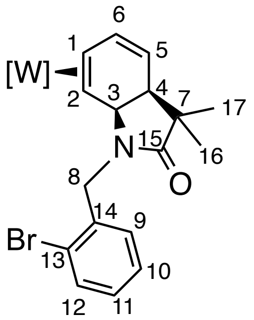

Compound 17:

Compound **3b** (50mg, 0.068 mmol) was placed in a test tube with ACN (2 mL), and chilled to -30 °C. After 10 min, a 1 M HOTf/ACN (0.136 mL, 0.136 mmol) solution was added to the test tube and the solution was allowed to stir at -30 °C for 30 min. In a separate test tube, 2-bromobenzylamine (0.088 mL, 0.682 mmol) with ACN (2 mL) was cooled at -30 °C for 20 min. After the time elapsed, the former solution was added to the latter, dropwise. The reaction stirred at -30 °C for 48 h and room temperature for 24 h. The reaction was washed three times (H_2O:Na_2CO_3/DCM; 30 mL/30mL) and dried over anhydrous Na_2SO_4. The organic layer was evaporated in vacuo. The resulting yellow film was dissolved in minimal DCM and pipetted in 25 mL of stirring hexane. A white solid precipitated out and was collected on a 15 mL fine-porosity fitted disk, washed with hexane (2 × 10 mL) and desiccated overnight to yield compound **17** (29 mg, 0.036 mmol, 53%).

^{1}H NMR (800 MHz, CD_3CN, δ, 25°C): 8.00 (d, J = 2.0 Hz, 1H, TpB3), 7.82 (d, J = 2.4 Hz, 1H, TpB5), 7.81 (d, J = 2.3 Hz, 1H, TpC5), 7.71 (d, J = 2.0 Hz, 1H, TpA3), 7.67 (d, J = 2.4 Hz, 1H,

TpA5), 7.27 (dd, J = 8.0, 1.1 Hz, 1H, H12), 7.23 (m, 3H, TpC3/H10/H9), 7.04 (ddd, J = 8.1, 6.7, 2.4 Hz, 1H, H11), 6.42 (ddd, J = 10.0, 4.9, 2.3 Hz, 1H, H6), 6.34 (t, J = 2.2 Hz, 1H, TpB4), 6.24 (t, J = 2.2 Hz, 1H, TpC4), 6.05 (t, J = 2.2 Hz, 1H, TpA4), 4.80 (dd, J = 9.9, 2.2 Hz, 1H, H5), 4.68 (d, J = 6.0 Hz, 1H, H3), 4.48 (d, J = 15.9 Hz, 1H, H8A), 4.42 (d, J = 15.9 Hz, 1H, H8B), 2.85 (m, 1H, H4), 2.58 (dddd, J = 12.5, 9.5, 4.8, 1.2 Hz, 1H, H1), 1.23 (d, J = 9.2 Hz, 1H, H2), 1.16 (d, J = 8.6 Hz, 9H, PMe_3). **^{13}C NMR (201 MHz, CD_3CN, δ, 25°C):** 180.63 (1C, C15), 133.36 (1C, C12), 132.25 (d, J = 3.6 Hz, 1C, C6), 130.26 (1C, C11), 129.38 (1C, C10), 128.35 (1C, C9), 115.45 (1C, C5), 107.50 (1C, TpB4), 107.09 (1C, TpC4), 106.84 (1C, TpA4), 59.56 (1C, C3), 49.84 (d, J = 9.9 Hz, 1C, C1), 47.51 (1C, C2), 44.18 (1C, C8), 43.88 (1C, C4), 13.66 (d, J = 28.8 Hz, 1C, PMe_3).

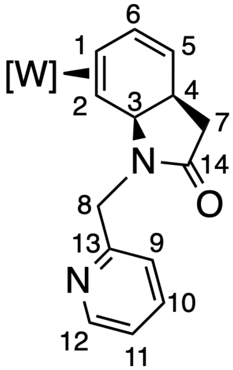

Compound 18:

Compound **3a** (100mg, 0.136 mmol) was placed in a test tube with ACN (2 mL), and chilled to -30 °C. After 10 min, a 1 M HOTf/ACN (0.273 mL, 0.273 mmol) solution was added to the test tube and the solution was allowed to stir at -30 °C for 30 min. In a separate test tube, 2-picolylamine (0.141 mL, 1.370 mmol) with ACN (2 mL) was cooled at -30 °C for 20 min. After the time elapsed, the former solution was added to the latter, dropwise. The reaction stirred at -30 °C for 48 h and room temperature for 24 h. The reaction was washed three times (H_2O:Na_2CO_3/DCM; 30 mL/30mL) and dried over anhydrous Na_2SO_4. The organic layer was evaporated in vacuo. The resulting yellow film was dissolved in minimal DCM and pipetted in 25 mL of stirring hexane. A white solid precipitated out and was collected on a 15 mL fine-porosity fitted disk, washed with hexane (2 × 10 mL) and desiccated overnight to yield compound **18** (72 mg, 0.099 mmol, 73%).

^{1}H NMR (800 MHz $(CD_3)_2CO$), δ, 25°C) 8.27 (ddd, J = 4.8, 1.8, 1.0 Hz, 1H, H12), 8.12 (d, J = 2.0 Hz, 1H, TpB3), 7.97 (d, J = 2.0 Hz, 1H, TpA3), 7.92 (d, J = 2.4 Hz, 1H, TpB5), 7.90 (d, J = 2.3 Hz, 1H, TpC5), 7.73 (d, J = 2.4 Hz, 1H, TpA5), 7.65 (td, J = 7.7, 1.8 Hz, 1H, H10), 7.30 (dt, J = 7.8, 1.0 Hz, 1H, H9), 7.26 (d, J = 2.1 Hz, 1H, TpC3), 7.13 (ddd, J = 7.5, 4.8, 1.1 Hz, 1H, H11), 6.41 (ddd, J = 9.7, 5.1, 2.6 Hz, 1H, H6), 6.38 (t, J = 2.2 Hz, 1H, TpB4), 6.28 (t, J = 2.2 Hz, 1H, TpC4), 6.15 (t, J = 2.2 Hz, 1H, TpA4), 4.89 (d, J = 6.3 Hz, 1H, H3), 4.63 (d, J = 16.1 Hz, 1H, H8A), 4.60 (dd, J = 9.7, 2.0 Hz, 1H, H5), 4.40 (d, J = 16.1 Hz, 1H, H8B), 3.37 (t, J = 7.5 Hz, 1H, H4), 2.73 (dd, J = 15.9, 8.2 Hz, 1H, H7A), 2.40 (dddd, J = 12.1, 9.5, 5.1, 1.1 Hz, 1H, H1), 2.02 (d, J = 16.0 Hz, 1H, H7B), 1.29 (m, 1H, H2), 1.19 (d, J = 8.6 Hz, 9H, PMe_3). **^{13}C NMR (201 MHz $(CD_3)_2CO$), δ, 25°C)** 175.4 (1C, C14), 160.1 (1C, C13), 149.4 (1C, C12), 144.5 (1C, TpB3), 143.3 (1C, TpA3), 141.7 (1C, TpC3), 137.8 (1C, TpC5), 137.0 (2C, TpA5/TpB5), 136.7 (d, J = 5.4 Hz, 1C, C10),

132.2 (d, J = 3.6 Hz, 1C, C6), 122.6 (1C, C9), 122.2 (1C, C11), 120.5 (1C, C5), 107.3 (1C, TpB4), 106.8 (1C, TpC4), 106.4 (1C, TpA4), 63.8 (1C, C3), 50.0 (d, J = 10.0 Hz, 1C, C1), 48.6 (1C, C2), 46.5 (1C, C8), 40.3 (1C, C7), 33.2 (1C, C4), 13.6 (d, J = 28.2 Hz, 1C, PMe_3).

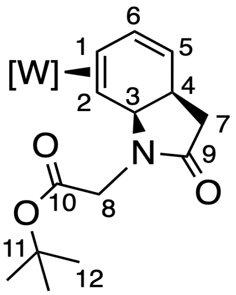

Compound 19:

Compound **3a** (50 mg, 0.068 mmol) was placed in a test tube, with ACN (2 mL), and chilled to -30 °C. After 10 min, a 1 M HOTf/ACN (0.136 mL, 0.136 mmol) solution was added to the test tube and the solution was allowed to stir at -30 °C for 10 min. In a separate test tube, amino-Acetic acid tert-butyl ester (0.089 mg, 0.682 mmol) with ACN (2 mL) was cooled at -30 °C for 10 min. After the time elapsed, the former solution was added to the latter, dropwise. The reaction stirred at -30 °C for 12 h then stirred at room temperature for 24 h. The reaction was washed three times (H_2O:Na_2CO_3/DCM; 30 mL/30mL) and dried over anhydrous Na_2SO_4. The green solution was evaporated in vacuo. The resulting film was dissolved in minimal DCM and pipetted in 15 mL of stirring hexane. A pale green solid precipitated out and was collected on a 15 mL fine-porosity fritted disk, washed hexane (2 × 10 mL) desiccated overnight to yield compound **19** (39.2 mg, 0.052 mmol, 76.4-81.0%).

^{1}H-NMR (800 MHz $(CD_3)_2CO$, δ, 25°C): 8.14 (1H, d, TpB3), 8.06 (1H, d, TpA3), 7.96 (2H, t, TpB5/C5), 7.79 (1H, d, TpA5), 7.64 (1H, d, TpC3), 6.41 (2H, m, H6/TpB4), 6.36 (1H, t, TC4), 6.26 (1H, t, TpA4), 4.87 (1H, d, H3), 4.50 (1H, dd, H5), 4.35 (1H, d, H8), 4.25 (1H, d, H8), 3.37 (1H, t, H4), 2.83 (1H, m, H1), 2.64 (1H, m, H7), 1.94 (1H, d, H7), 1.26 (1H, d, H2), 1.25 (9H, d, PMe_3) 1.12 (9H, s, H12).**^{13}C-NMR (201 MHz $(CD_3)_2CO$, δ, 25°C):** 175.1 (1C, C9), 169.0 (1C, C10), 144.6 (1C, TpB3) 143.6 (1C, TpA3), 141.9 (1C, TpB5), 137.8 (1C, TpC5), 137.1 (1C, TpA5), 136.7 (1C, TpC3), 132.3 (1C, C6), 120.4 (1C, C5), 107.3 (1C, TpB4), 107.0 (1C, TpC4), 106.6 (1C, TpA4), 81.1 (1C, C11), 62.7 (1C, C3), 49.0 (1C, d, C1), 48.6 (1C, C2), 42.7 (1C, C8), 40.2 (1C, s, C7), 33.5 (1C, s, C4), 28.3 (1C, C12), 13.8 (3C, d, PMe_3).

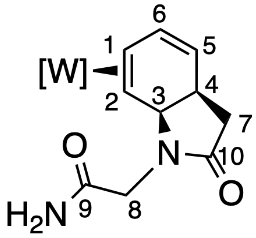

Compound 20:

Compound **3a** (50mg, 0.068 mmol) was placed in a test tube with ACN (2 mL), and chilled to -30 °C. After 10 min, a 1 M HOTf/ACN (0.136 mL, 0.136 mmol) solution was added to the test tube and the solution was allowed to stir at -30 °C for 30 min. In a separate test tube, 2-Aminoacetamide (52 mg, 0.690 mmol) with ACN (2 mL) was cooled at -30 °C for 20 min. After the time elapsed, the former solution was added to the latter, dropwise. The reaction stirred at -30 °C for 48 h and room temperature for 24 h. The reaction was washed three times (H_2O:Na_2CO_3/DCM; 30 mL/30mL) and dried over anhydrous Na_2SO_4. The organic layer was evaporated in vacuo. The resulting yellow film was dissolved in minimal DCM and pipetted in 25 mL of stirring hexane. A white solid precipitated out and was collected on a 15 mL fine-porosity fitted disk, washed with hexane (2 × 10 mL) and desiccated overnight to yield compound **20** (18 mg, 0.026 mmol, 67%).

^{1}H NMR (800 MHz, $CD_3)_2CO$, δ, 25°C) 8.15 (s, 1H, TpB3), 8.13 (s, 1H, TpA3), 7.95 (dd, *J* = 5.6, 2.8 Hz, 2H, TpB5/C4), 7.80 (d, *J* = 2.8 Hz, 1H, TpA5), 7.59 (s, 1H, TpC3), 6.53 (d, *J* = 18.5 Hz, 1H, H6), 6.42 (d, *J* = 2.2 Hz, 1H, TpB4), 6.34 (d, *J* = 2.6 Hz, 1H, TpC4), 6.26 (d, *J* = 2.7 Hz, 1H, TpA4), 4.88 (s, 1H, H3), 4.61 (d, *J* = 9.9 Hz, 1H, H5), 3.88 (dd, *J* = 16.9, 2.6 Hz, 1H, H8A), 3.61 (m, 1H, H8B), 3.38 (s, 1H, H4), 2.78 (m, 1H, H1), 2.71 (m, 1H, H7A), 1.97 (dd, *J* = 16.2, 2.5 Hz, 1H, H7B), 1.36 (m, 1H, H2), 1.26 (d, *J* = 6.7 Hz, 9H, PMe_3). **^{13}C NMR (201 MHz, $CD_3)_2CO$, δ, 25°C)** 176.0 (1C, C10), 144.5 (1C, TpB3), 143.6 (1C, TpA3), 141.9 (TpC3), 137.03 & 136.25 (2C, TpC3/TpC5), 136.02 (1C, TpA5), 131.46 (d, *J* = 3.6 Hz, 1C, H6), 119.84 (1C, H5), 106.46 (1C, TpC4), 106.20 (1C, TpB4), 105.68 (1C, TpA4), 63.32 (1C, C3), 49.00 (d, *J* = 10.1 Hz, 1C, C1), 47.22 (1C, C2), 43.75 (d, *J* = 5.6 Hz, 1C, C8), 39.01 (1C, C7), 32.69 (1C, C4), 12.71 (d, *J* = 28.3 Hz, 1C, PMe_3).

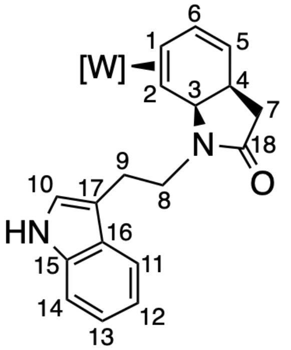

Compound 21:

Compound **3a** (100mg, 0.136 mmol) was placed in a test tube with ACN (2 mL), and chilled to -30 °C. After 10 min, a 1 M HOTf/ACN (0.272 mL, 0.272 mmol) solution was added to the test tube and the solution was allowed to stir at -30 °C for 30 min. In a separate test tube, Tryptamine (220 mg, 1.38 mmol) was cooled at -30 °C for 20 min. After the time elapsed, the former solution was added to the latter, dropwise. The reaction stirred at -30 °C for 48 h and room temperature for 24 h. The reaction was washed three times (H_2O:NaOH/DCM; 30 mL/30mL) and dried with anhydrous Na_2SO_4. The organic layer was evaporated in vacuo. The resulting yellow film was dissolved in minimal DCM and pipetted in 25 mL of stirring hexane. A white solid precipitated out

and was collected on a 15 mL fine-porosity fitted disk, washed with hexane (2 × 10 mL) and desiccated overnight to yield compound **21** (210 mg, 0.268 mmol, 76%).

^{1}H NMR (800 MHz, $(CD_3)_2CO$, δ, 25°C) 10.0 (s, 1H, NH), 8.19 (d, *J* = 2.1 Hz, 1H, TpA3), 8.17 (d, *J* = 2.0 Hz, 1H, TpB3), 8.02 (d, *J* = 2.3 Hz, 1H, TpC5), 7.97 (d, *J* = 2.2 Hz, 1H, TpB5), 7.84 (d, *J* = 2.4 Hz, 1H, TpA5), 7.58 (d, *J* = 2.1 Hz, 1H, TpC3), 7.40 (dt, *J* = 8.1, 0.9 Hz, 1H, H10), 6.43 (ddd, *J* = 9.6, 5.0, 2.5 Hz, 1H, H6), 6.41 (t, *J* = 2.2 Hz, 1H, TpB4), 6.37 (t, *J* = 2.2 Hz, 1H, TpC4), 6.31 (t, *J* = 2.2 Hz, 1H, TpA4), 4.87 (d, *J* = 6.2 Hz, 1H, H3), 4.57 (dd, *J* = 9.7, 2.0 Hz, 1H, H5), 3.57 (m, 1H, H8A), 3.37 (m, 2H, H4/H8B), 2.81 (m, 3H, H1/H9A/H9B), 2.62 (dd, *J* = 15.7, 8.2 Hz, 1H, H7A), 1.93 (d, *J* = 15.6 Hz, 1H, H7B), 1.59 (d, *J* = 9.6 Hz, 1H, H2), 1.27 (d, *J* = 8.5 Hz, 9H, PMe_3). **^{13}C NMR (201 MHz, $(CD_3)_2CO$, δ, 25°C)** 174.7 (1C, C18), 144.6 (1C, TpA3), 143.4 (1C, TpB3), 142.0 (1C, TpC3), 137.8 (1C, TpC5), 137.1 (1C, TpA5), 137.0 (1C, TpB5), 131.9 (d, *J* = 3.5 Hz, 1C, C6), 120.5 (1C, C5), 119.6 (1C, C10), 107.3 (1C, TpB4), 107.2 (1C, TpC4), 106.6 (1C, TpA4), 62.6 (1C, C3), 49.4 (d, *J* = 10.0 Hz, 1C, C1), 48.2 (1C, C2), 41.4 (1C, C8), 40.4 (1C, C7), 33.0 (1C, C4), 24.6 (1C, C9), 13.7 (d, *J* = 28.2 Hz, 1C, PMe_3).

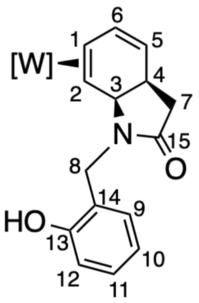

Compound 22:

Compound **3a** (150 mg, 0.205 mmol) was placed in a test tube with ACN (2 mL), and chilled to -30 °C. After 10 min, a 1 M HOTf/ACN (0.409 mL, 0.409 mmol) solution was added to the test tube, and the solution was allowed to stir at -30 °C for 30 min. In a separate test tube, 2-(aminomethyl)phenol (150 mg, 2.05 mmol) was cooled at -30 °C for 20 min. After the time elapsed, the former solution was added to the latter, dropwise. The reaction stirred at -30 °C for 48 h and room temperature for 24 h. The reaction was washed three times (H_2O:NaOH/DCM; 30 mL/30mL) and dried with anhydrous Na_2SO_4. The organic layer was evaporated in vacuo. The resulting green film was dissolved in minimal DCM and pipetted in 25 mL of stirring hexane. A pale green solid precipitated out and was collected on a 15 mL fine-porosity fritted disk, washed with hexane (2 × 10 mL) and desiccated overnight to yield compound **22** (101 mg, 0.136 mmol, 66%).

^{1}H NMR (800 MHz, CD_3CN, δ, 25°C): 8.09 (d, *J* = 2.1 Hz, 1H, TpA3), 7.90 (d, *J* = 2.0 Hz, 1H, TpB3), 7.81 (d, *J* = 2.3 Hz, 1H, TpC5), 7.74 (d, *J* = 2.2 Hz, 1H, TpB5), 7.67 (d, *J* = 2.4 Hz, 1H, TpA5), 7.42 (d, *J* = 2.1 Hz, 1H, TpC3), 7.11 (1H, td, H12), 6.91 (1H, dt, H9), 6.48 (1H, tt, H10), 6.45 (1H, dd, H11), 6.36 (1H, m, H6), 6.34 (t, *J* = 2.2 Hz, 1H, TpB4), 6.32 (t, *J* = 2.2 Hz, 1H, TpC4), 6.19 (t, *J* = 2.2 Hz, 1H, TpA4) 4.70 (1H, d, H3), 4.64 (1H, dd, H5), 4.47 (1H, d, H8A), 4.28 (1H, d, H8B), 3.39 (1H, t, H4), 2.69 (1H, dd, H7A), 2.66 (1H, m, H1), 2.17 (1H, m, H7B), 1.67 (1H, d, H2), 1.22 (9H, d, PMe3). **^{13}C NMR (201 MHz, CD_3CN, δ, 25°C):** 177.8 (1C, C15), 156.5 (1C,

C13), 131 (1C, C11), 130.6 (1C, C6), 129.5 (1C, C12), 123.5 (1C, C14), 119.7 (1C, C5), 119.0 (1C, C10), 118.2 (1C, C9), 63.4 (1C, C3), 49.1 (1C, C1), 47.0 (1C, C2), 40.3 (1C, C8), 39.5 (1C, C7), 32.5 (1C, C4), 13.9 (3C, PMe3).

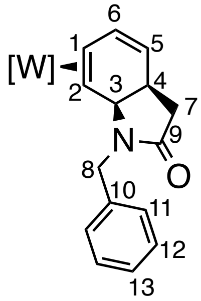

Compound 23:
Compound **3a** (150 mg, 0.204 mmol) was placed in a test tube, with CH_3CN, and chilled to -30 °C. After 10 min, a 1 M HOTf/CH_3CH_2CN (0.409 mL, 0.409 mmol) solution was added to the test tube and the solution was allowed to stir at -30 °C for 10 min. In a separate test tube, benzylamine (0.446 mL, 4.08 mmol) was cooled at -30 °C for 10 min. After the time elapsed, the former solution was added to the latter, dropwise. The reaction was washed three times (H_2O:Na_2CO_3/DCM; 30 mL/30mL) and dried with Mg_2SO_4. The clear solution was evaporated in vacuo. The resulting film was dissolved in minimal DCM and pipetted in 15 mL of stirring hexane. A tan/white solid precipitated out and was collected on a 15 mL fine-porosity fritted disk, washed hexane (2 × 10 mL) desiccated overnight to yield **23** (94.1 mg, 0.130 mmol, 63.0%).

^{1}H NMR (800 MHz, CD_3CN, δ, 25°C): 8.03 (1H, d, TpB3), 7.83 (2H, d, TpB5/C5), 7.81 (1H, d, TpA3), 7.28 (1H, d, TpC3), 7.15 (3H, m, H12/13/14), 6.99 (2H, m, H11/15), 6.42 (1H, m, H6) 6.35 (1H, t, TB4), 6.27 (1H, t, TpC4) 6.08 (1H, t, TpA4), 4.67 (1H, d, H3), 4.56 (1H, d, H5), 4.49 (1H, d, H8), 4.31 (1H, d, H8), 3.23 (1H, m, H4), 2.70 (1H, ddd J= 16.18 Hz, H7), 2.55 (1H, m, H1), 1.99 (1H, ddd, H7), 1.25 (1H, d, H2). **^{13}C NMR (201 MHz, CD_3CN, δ, 25°C)**: 175.9 (1C, C9), 144.5 (1C, TpB3), 143.4 (1C, TpA5), 142.0 (1C, TpC3), 137.9 (1C, TpA3), 137.5 (1C, TpB5), 137.0 (1C, TpA5), 132.3 (1C, C6), 129.0 (2C, C12/14), 128.3 (2C, C10/11/15), 127.4 (1C, C13), 120.3 (1C, C5), 107.5 (1C, TpB4), 107.1 (1C, Tp4C) 106.7 (1C, Tp4A), 62.9 (1C, C3), 49.7 (1C, C1), 48.5 (1C, C2), 43.9 (1C, C8), 40.2 (1C, C7) 33.1 (1C, C4), 13.6 (3C, d J= 29.30 Hz, PMe3).

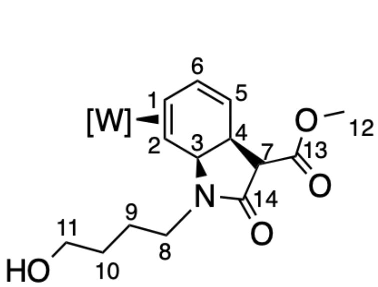

Compound 24:
Compound **3c** (117 mg, 0.148 mmol) was placed in a test tube with ACN (2 mL), and chilled to -30 °C. After 10 min, a 1 M HOTf/ACN (0.296 mL, 0.296 mmol) solution was added to the test tube

and the solution was allowed to stir at -30 °C for 30 min. In a separate test tube, 4-aminobutanol (0.136 mL, 1.480 mmol) with ACN (2 mL) was cooled at -30 °C for 20 min. After the time elapsed, the former solution was added to the latter, dropwise. The reaction stirred at -30 °C for 24 h and room temperature for 24 h. The reaction was washed three times (H_2O:NaOH/DCM; 30 mL/30mL) and dried over anhydrous Na_2SO_4. The organic layer was evaporated in vacuo. The resulting yellow film was dissolved in minimal DCM and pipetted in 25 mL of stirring hexane. A white solid precipitated out and was collected on a 15 mL fine-porosity fitted disk, washed with hexane (2 × 10 mL) and desiccated overnight to yield compound **24** (102 mg, 0.133 mmol, 89%).

^{1}H NMR (800 MHz, CD_3CN, δ, 25°C) 8.06 (d, *J* = 2.1 Hz, 1H, TpB3), 7.98 (d, *J* = 2.2 Hz, 1H, TpA5), 7.87 (m, 2H, TpB5/TpC5), 7.80 (d, *J* = 2.4 Hz, 1H, TpA3), 7.50 (d, *J* = 2.2 Hz, 1H, TpC3), 6.49 (ddd, *J* = 9.3, 4.9, 2.4 Hz, 1H, H6), 6.38 (t, *J* = 2.2 Hz, 1H, TpB4), 6.32 (dt, *J* = 3.1, 2.2 Hz, 2H, TpA4/TpC4), 4.97 (d, *J* = 6.2 Hz, 1H, H3), 4.52 (dd, *J* = 9.6, 2.2 Hz, 1H, H5), 3.72 (s, 3H, H12), 3.36 (m, 4H, H4/H8A/H11A/H11B), 3.12 (dd, *J* = 12.0, 7.8 Hz, 1H, H8B), 2.99 (s, 1H, H7), 2.83 (m, 1H, H1), 1.31 (m, 5H, H2/H9A/H9B/H10A/H10B), 1.19 (d, *J* = 8.7 Hz, 9H, PMe3). **^{13}C NMR (201 MHz, CD_3CN, δ, 25°C)** 172.1 (1C, C14), 170.5 (1C, C13), 144.7 (1C, TpB3), 143.1 (1C, TpA3), 142.2 (1C, TpC3), 138.1 (1C, TpC5), 137.5 (2C, TpA5/TpB5), 133.0 (1C, C6), 118.3 (1C, C5), 107.6 (1C, TpB4), 107.3 (1C, TpC4), 107.0 (1C, TpA4), 62.2 (1C, C11), 61.5 (1C, C3), 58.1 (1C, C7), 52.9 (1C, C12), 49.1 (1C, C1), 47.6 (1C, C2), 40.0 (1C, C8), 37.9 (1C, C4), 30.3 (1C, C9), 25.1 (1C, C10), 13.8 (3C, PMe3).

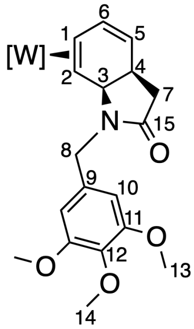

Compound 25:

Compound **3a** (105 mg, 0.143 mmol) was placed in a test tube with ACN (2 mL), and chilled to -30 °C. After 10 min, a 1 M HOTf/ACN (0.286 mL, 0286 mmol) solution was added to the test tube and the solution was allowed to stir at -30 °C for 30 min. In a separate test tube, 3,4,5-trimethoxybenzylamine (282 mg, 1.430 mmol) with ACN (2 mL) was cooled at -30 °C for 20 min. After the time elapsed, the former solution was added to the latter, dropwise. The reaction stirred at -30 °C for 48 h and room temperature for 24 h. The reaction was washed three times (H_2O:Na_2CO_3/DCM; 30 mL/30mL) and dried over anhydrous Na_2SO_4. The organic layer was evaporated in vacuo. The resulting yellow film was dissolved in minimal DCM and pipetted in 25 mL of stirring hexane. A white solid precipitated out and was collected on a 15 mL fine-porosity fitted disk, washed with hexane (2 × 10 mL) and desiccated overnight to yield compound **25** (81 mg, 0.099 mmol, 69%).

^{1}H NMR (800 MHz, CD_3CN, δ, 25°C) 8.02 (d, *J* = 2.1 Hz, 1H, TpB3), 7.83 (d, *J* = 2.5 Hz, 1H, TpB5), 7.82 (d, *J* = 2.4 Hz, 1H, TpC5), 7.77 (d, *J* = 2.1 Hz, 1H, TpA3), 7.68 (d, *J* = 2.4 Hz, 1H, TpA5), 7.29 (d, *J* = 2.4 Hz, 1H, TpC3), 6.43 (ddd, *J* = 9.9, 5.2, 2.6 Hz, 1H, H6), 6.35 (t, *J* = 2.2 Hz, 1H, TpB4), 6.27 (t, *J* = 2.2 Hz, 1H, TpC4), 6.22 (s, 2H, H10), 6.05 (t, *J* = 2.2 Hz, 1H, TpA4), 4.57 (m, 2H, H3/H5), 4.47 (d, *J* = 15.1 Hz, 1H, H8A), 4.20 (d, *J* = 15.1 Hz, 1H, H8B), 3.63 (s, 3H, H13), 3.52 (s, 6H, H14), 3.19 (t, *J* = 7.3 Hz, 1H, H4), 2.71 (dd, *J* = 16.0, 8.1 Hz, 1H, H7A), 2.59 (ddd, *J* = 13.9, 9.7, 5.1 Hz, 1H, H1), 1.97 (d, *J* = 16.1 Hz, 1H, H7B), 1.27 (d, *J* = 9.7 Hz, 1H, H2), 1.15 (d, *J* = 8.7 Hz, 9H, PMe3). **^{13}C NMR (201 MHz, CD_3CN, δ, 25°C)** 175.7 (1C, C15), 154.01 (2C, C11), 144.6 (1C, TpB3), 143.4 (1C, TpA3), 141.8 (1C, TpC3), 137.9 (1C, C12), 137.4 (1C, TpB5), 137.3 (1C, TpC5), 137.0 (1C, TpA5), 135.5 (1C, C9), 132.3 (1C, C6), 120.4 (1C, C5), 107.5 (1C, TpB4), 107.1 (1C, TpC4), 106.6 (1C, TpA4), 105.3 (2C, C10), 62.9 (1C, C3), 60.8 (1C, C13), 56.3 (2C, C14), 49.6 (1C, C1), 48.4 (1C, C2), 44.3 (1C, C8), 40.3 (1C, C7), 33.1 (1C, C4), 13.7 (3C, PMe3).

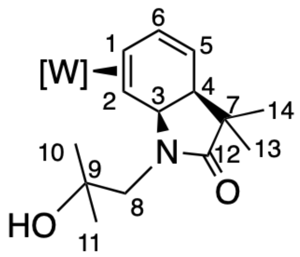

Compound 26:

Compound **3b** (100mg, 0.131 mmol) was placed in a test tube with ACN (2 mL), and chilled to -30 °C. After 10 min, a 1 M HOTf/ACN (0.263 mL, 0.263 mmol) solution was added to the test tube and the solution was allowed to stir at -30 °C for 30 min. In a separate test tube, 4-amino-2-methylbutan-2-ol (0.142 mL, 1.31 mmol) with ACN (2 mL) was cooled at -30 °C for 30 min. After the time elapsed, the former solution was added to the latter, dropwise. The reaction stirred at -30 °C for 24 h and at room temperature for 24 h. The reaction was washed three times (H_2O:Na_2CO_3/DCM; 30 mL/30mL) and dried over anhydrous Na_2SO_4. The organic layer was evaporated in vacuo. The resulting yellow film was dissolved in minimal DCM and pipetted in 25 mL of stirring hexane. A white solid precipitated out and was collected on a 15 mL fine-porosity fitted disk, washed with hexane (2 × 10 mL) and desiccated overnight to yield compound **26** (78 mg, 0.106 mmol, 80.4%).

^{1}H NMR (800 MHz, CD_3CN, δ, 25°C) 8.06 (1H, d, TpB3), 7.98 (1H, TpA5), 7.86 (1H, d, TpA3), 7.86 (d, 1H, TpC5), 7.76 (1H, d, TpB5), 7.48 (1H, d, TpC3), 6.43 (1H, m, H6), 6.37 (t, 1H, TpB4), 6.31 (t, 1H, TpC4), 6.26 (t, 1H, TpA4), 4.83 (1H, d, H3), 4.74 (1H, dd, H5), 3.16 (2H, s, H8), 2.90 (1H, m, H4), 2.82 (1H, m, H1), 1.40 (1H, d, H2), 1.27 (3H, s, H10), 1.18 (9H, d, PMe_3), 1.12 (3H, s, H11), 1.02 (3H, s, H13), 0.94 (3H, s, H14). **^{13}C NMR (201 MHz, CD_3CN, δ, 25°C)** 183.2 (1C, C12), 144.6 (1C, TpB3), 143.6 (1C, TpA5), 142.1 (1C, TpA3), 138.0 (1C, TpC5), 137.4 (1C, TpB5), 137.3 (1C, TpC3), 132.3 (1C, C6), 115.2 (1C, C5), 107.6 (1C, TpB4), 107.3 (1C, TpC4) , 106.9 (1C, TpA4), 72.6 (1C, C9), 61.9 (1C, C3), 52.7 (1C, C8), 50.2 (1C, C1), 47.3 (1C, C2), 44.8 (1C, C7), 44.7 (1C, C4), 28.7 (1C, C14), 28.3 (1C, C13), 25.3 (1C, C10), 20.8 (1C, C11), 13.6 (d, *J* = 28.5 Hz, 1C, PMe_3).

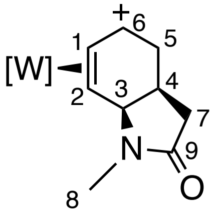

Compound 27:
Compound **9** (50 mg, 0.077 mmol) was placed in a test tube, with ACN (2 mL), and chilled to -30 °C. After 10 min, a 1 M HOTf/ACN (0.15 mL, 0.415 mmol) solution was added to the test tube and the solution was allowed to stir at -30 °C for 15 min. The reaction mixture was pipetted in 30 mL of stirring diethyl ether. A tan solid precipitated out and was collected on a 15 mL fine-porosity fritted disk, washed with hexane (2 × 10 mL) and desiccated overnight to yield compound **27** (45 mg, 0.069 mmol, 90.0%).

^{1}H-NMR (800 MHz, $(CD_3)_2CO$, δ, 25°C) 8.57 (1H, d, TpB3), 8.39 (1H, d, TpB5), 8.27 (1H, d, TpC3), 8.21 (1H, d, TpA3), 8.20 (1H, d, TpC5), 8.03 (1H, d, TpA5), 6.65 (1H, t, TpC5), 6.63 (1H, t, TpA4) 6.49 (1H, t, TpC4), 5.73 (1H, t, H1), 5.48 (1H, d, H2), 5.33 (1H, m, H6), 5.05 (1H, d, H3), 3.33 (1H, m, H5), 3.07 (3H, s, H8), 3.02 (1H, d, H5), 2.63 (1H, t, H4), 2.36 (1H, m, H7), 2.23 (1H, d, H7), 1.36 (3C, d J= 29.30 Hz, PMe3). **^{13}C-NMR (201 MHz, $(CD_3)_2CO$, δ, 25 ºC):** 172.7 (1C, C9), 147.8 (1C, TpC5), 147.7 (1C, TpA3),145.8 (1C, TpB3), 144.1 (1C, TpC3), 139.8 (1C, TpC5), 139.6 (1C, TpA5), 109.5 (1C, TpB4), 109.2 (1C, TpB4), 108.4 (1C, TpA4) 108.5 (1C, C2), 105.2 (1C, C3), 78.5 (1C, C1), 61.0 (1C, C4), 36.5 (1C, C7), 27.9 (1C, C5), 27.6 (1C, C8), 27.5 (1C, C5), 13.0 (3C, d J= 29.30 Hz, PMe3).

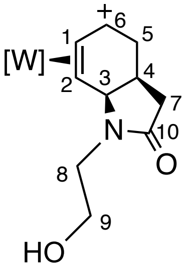

Compound 28:
Compound **10** (140 mg, 0.205 mmol) was placed in a test tube, with ACN (2 mL), and chilled to -30 °C. After 10 min, a 1 M HOTf/ACN (0.15 mL, 0.415 mmol) solution was added to the test tube and the solution was allowed to stir at -30 °C for 15 min. The reaction mixture was pipetted in 30 mL of stirring diethyl ether. A tan solid precipitated out and was collected on a 15 mL fine-porosity fritted disk, washed with hexane (2 × 10 mL) and desiccated overnight to yield compound **28** (120 mg, 0.176 mmol, 85.6%). Complex is unstable in solution. Partial characterization.

^{1}H-NMR (800 MHz, $((CD_2)_4O)$, δ, 25°C) 8.58 (1H, d, TpB3), 8.48 (1H, d, TpB5), 8.40 (1H, d, TpC3), 8.07 (2H, d, TpA3/TpA5), 7.89 (1H, d, TpC5), 6.58 (1H, t, TpB4), 6.55 (1H, t, TpA4), 6.41 (1H, t, TpC4), 5.64 (1H, t, H1), 5.51 (1H, d, H2), 5.44 (1H, m, H6), 5.40 (1H, d, H3), 3.80 (3H, m,

H5A, H9A/H9B), 3.46 (1H, ,m, H5B), 2.93 (1H, dd, H4), 2.68 (1H, s, H8A), 2.58 (2H, d, H8B/H7A), 1.94 (1H, m, H7B), 1.26 (9H, d, PMe_3).

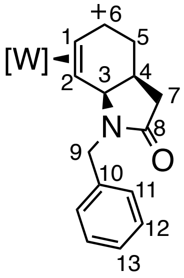

Compound 29:

Compound **23** (100 mg, 0.137 mmol) taken in DCM (2 mL) and cooled to -30°C. 1M HOTf in ACN (151 mL, 0.151 mmol) was then added and the reaction stirred for 30 min. Precipitation was then induced by adding the crude reaction dropwise into stirring Ether (60 mL) and the product collected as a white powder on a fritted disc to yield compound **29** (95 mg, 0.108 mmol, 79%)

^{1}H NMR (600 MHz, CD_3CN) δ 8.29 (1H, d, *J* = 2.3 Hz, Tp3B), 7.99 (1H, dt, *J* = 2.4, 0.6 Hz, Tp5C), 7.94 (1H, dt, *J* = 2.5, 0.8 Hz, Tp5B), 7.80 (2H, td, *J* = 1.9, 1.0 Hz, Tp5A/Tp3C), 7.56 (1H, d, *J* = 2.2 Hz, Tp3A), 7.38 (5H, m, H11/H12/H13), 6.51 (1H, t, *J* = 2.3 Hz, Tp4C), 6.49 (1H, t, *J* = 2.4 Hz, Tp4B), 6.31 (1H, t, *J* = 2.4 Hz, Tp4A), 5.37 (1H, t, *J* = 7.6 Hz, H3), 5.04 (1H, dd, *J* = 14.3, 7.0 Hz, H1), 5.00 (2H, t, *J* = 8.2 Hz, H2/H4), 4.93 (1H, d, *J* = 15.8 Hz, H9), 4.62 (1H, d, *J* = 15.8 Hz, H9), 3.24 (1H, dd, *J* = 17.4, 6.6 Hz, H6), 2.85 (1H, ddd, *J* = 17.4, 7.6, 1.2 Hz, H6), 2.80 (1H, dd, *J* = 16.7, 8.1 Hz, H7), 2.71 (1H, p, *J* = 8.4 Hz, H5), 2.63 (1H, dd, *J* = 16.6, 9.5 Hz, H7), 1.13 (9H, d, *J* = 9.9 Hz, PMe_3). **^{13}C NMR (201 MHz, CD_3CN) δ** 176.6 (1C, C8), 147.2 (1C, Tp3A), 145.7 (1C, Tp3B), 143.8 (1C, Tp3C), 139.9 (1C,Tp5C), 139.8 (1C, Tp5B), 139.6 (1C, Tp5A), 136.6 (1C, C10), 130.1 (1C, C12), 129.2 (1C, C13), 128.9 (1C, C11), 122.0 (1C, d, *J* = 320.6 Hz, -OTf), 109.6 (1C, Tp4B), 109.2 (1C, Tp4C), 108.5 (1C, Tp4A), 107.5 (1C, d, *J* = 3.2 Hz, C3), 102.3 (1C, C2), 80.8 (1C, C1), 61.5 (1C, C4), 46.7 (1C, C9), 36.7 (1C, C7), 28.4 (1C, C5), 28.1 (1C, d, *J* = 3.2 Hz, C6), 13.0 (3C, d, *J* = 33.4 Hz, PMe_3). **HRMS (APCI^{+}):** [M+] calcd. for $C_{27}H_{35}BN_8O_2PW$, 729.2223; found, 729.2223.

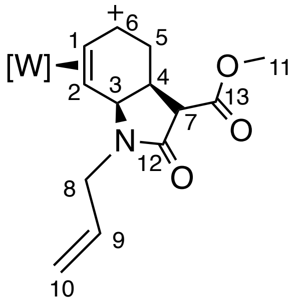

Compound 30:
Compound **15** (115 mg, 0.156 mmol) was placed in a test tube, with ACN (2 mL), and chilled to -30 °C. After 10 min, a 1 M HOTf/ACN (0.15 mL, 0.415 mmol) solution was added to the test tube and the solution was allowed to stir at -30 °C for 15 min. The reaction mixture was pipetted in 30 mL of stirring diethyl ether. A tan solid precipitated out and was collected on a 15 mL fine-porosity fritted disk, washed with hexane (2 × 10 mL) and desiccated overnight to yield compound **30** (100 mg, 0.136 mmol, 86.8%). Complex is unstable in solution. Partial characterization.

^{1}H NMR (800 MHz, CD_3CN, δ, 25°C) 8.37 (1H, d, TpB3), 8.04 (1H, d, TpB5), 8.00 (1H, d, TpC3), 7.96 (1H, d, TpA3), 7.84 (1H, d, TpA5), 7.83 (1H, d, TpC5), 6.53 (1H, t, TpB4), 6.52 (1H, t, TpA4) 6.40 (1H, t, TpC4), 5.93 (1H, m, H9), 5.42 (3H, m, H1/H10A/H10B), 5.27 (1H, d, H2), 5.05 (1H, d, H3), 4.79 (1H, m, H6), 4.30 (1H, q, H8A), 3.97 (1H, q, H8B), 3.79 (1H, m, H5A), 3.76 (3H, s, H11), 3.45 (1H, d, H5B), 3.19 (1H, m, H4), 1.16 (9H, d, PMe3) .

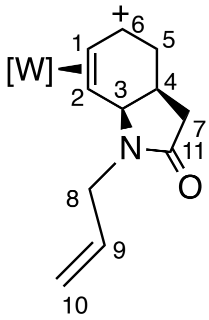

Compound 31:
Compound **16** (150 mg, 0.221 mmol) was placed in a test tube, with ACN (2 mL), and chilled to -30 °C. After 10 min, a 1 M HOTf/ACN (0.15 mL, 0.415 mmol) solution was added to the test tube and the solution was allowed to stir at -30 °C for 15 min. The reaction mixture was pipetted in 30 mL of stirring diethyl ether. A tan solid precipitated out and was collected on a 15 mL fine-porosity fritted disk, washed with hexane (2 × 10 mL) and desiccated overnight to yield compound **31** (120 mg, 0.177 mmol, 79.9%). Complex is unstable in solution. Partial characterization.

^{1}H NMR (800 MHz, CD_3CN, δ, 25°C) 8.35 (1H, d, TpB3), 8.01 (2H, d, TpB5/TpC3), 7.97 (1H, d, TpA3), 7.85 (2H, d, TpA5), 7.83 (1H, d, TpC5), 6.53 (2H, t, TpB4/TpA4), 6.40 (1H, t, TpC4),5.93 (1H, m, H9), 5.48 (1H, s, H1), 5.40 (1H, d, H10A), 5.30 (1H, d, H10B), 5.15 (1H, d, H2), 5.09 (2H, m, H3/H6), 4.37 (1H, q, H8A), 4.01 (1H, q, H8B), 3.23 (1H, dd, H5A), 2.89 (1H, dd, H5B), 2.72 (2H, m, H4/H7A), 2.54 (1H, m, H7B) 1.16 (9H, d, PMe3) .

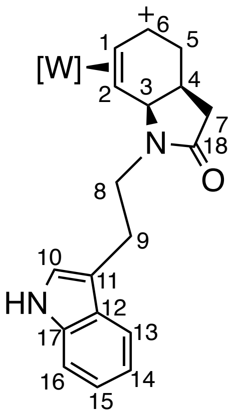

Compound 32:
Compound **21** (95 mg, 0.121 mmol) was placed in a test tube, with ACN (2 mL), and chilled to -30 °C. After 10 min, a 1 M HOTf/ACN (0.15 mL, 0.415 mmol) solution was added to the test tube and the solution was allowed to stir at -30 °C for 15 min. The reaction mixture was pipetted in 30 mL of stirring diethyl ether. A tan solid precipitated out and was collected on a 15 mL fine-porosity fritted disk, washed with hexane (2 × 10 mL) and desiccated overnight to yield compound **32** (75 mg, 0.101 mmol, 78.8%). Complex is unstable in solution. Partial characterization.

^{1}H NMR (800 MHz, CD_3CN, δ, 25°C) 8.23 (1H, d, TpB3), 7.99 (1H, d, TpB5), 7.94 (1H, d, TpC3), 7.83 (1H, d, TpA3), 7.62 (1H, d, TpA5), 7.58 (2H, d, TpC5/H10), 7.44 (1H, d, H16), 7.31 (1H, d, H13), 7.10 (2H, m, H15/H14), 6.40 (2H, t, TpB4/TpA4), 6.29 (1H, t, TpC4), 5.17 (1H, t, H1), 4.93 (1H, d, H2), 4.67 (1H, m, H6), 4.46 (1H, d, H3), 3.55 (1H, m, H8A), 3.27 (1H, m, H8B), 3.21 (3H, m, H5A/H5B/H9A), 2.78 (1H, m, H9B), 2.64 (1H, q, H7A), 2.48 (1H, t, H4), 2.40 (1H, q, H7B), 1.10 (9H, d, PMe_3).

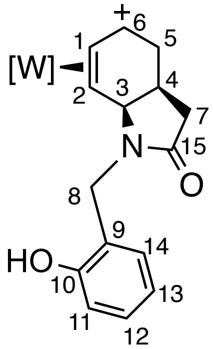

Compound 33:
Compound **22** (130 mg, 0.175 mmol) was placed in a test tube, with ACN (2 mL), and chilled to -30 °C. After 10 min, a 1 M HOTf/ACN (0.15 mL, 0.415 mmol) solution was added to the test tube and the solution was allowed to stir at -30 °C for 15 min. The reaction mixture was pipetted in 30 mL of stirring diethyl ether. A tan solid precipitated out and was collected on a 15 mL fine-porosity fritted disk, washed with hexane (2 × 10 mL) and desiccated overnight to yield compound **33** (83 mg, 0.112 mmol, 63.7%). Complex is unstable in solution. Partial characterization.

¹H NMR (800 MHz, CD_3CN, δ, 25°C) 8.35 (1H, d, TpB3), 8.12 (1H, d, TpB5), 8.00 (1H, d, TpC3), 7.95 (1H, d, TpA3), 7.83 (1H, d, TpA5), 7.81 (1H, d, TpC5), 7.30 (1H, d, H14), 7.19 (2H, m, H12/H11), 6.84 (1H, m, H13) 6.52 (2H, t, TpB4/TpA4), 6.41 (1H, t, TpC4), 5.34 (2H, m, H2/H1), 4.98 (1H, m, H6), 4.75 (1H, d, H3), 4.69 (1H, dd, H8A), 4.53 (1H, dd, H8B), 3.13 (2H, m, H5A/H4), 2.80 (1H, m, H5B), 2.53 (2H, m, H7A/H7B), 1.14 (9H, d, PMe_3).

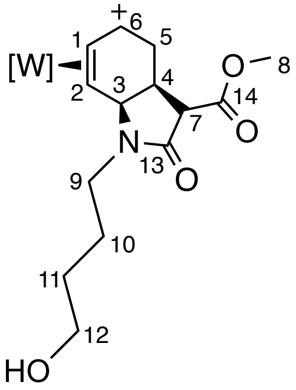

Compound 34:
Compound **24** (105 mg, 0.137 mmol) was placed in a test tube, with ACN (2 mL), and chilled to -30 °C. After 10 min, a 1 M HOTf/ACN (0.15 mL, 0.415 mmol) solution was added to the test tube and the solution was allowed to stir at -30 °C for 15 min. The reaction mixture was pipetted in 30 mL of stirring diethyl ether. A tan solid precipitated out and was collected on a 15 mL fine-porosity fritted disk, washed with hexane (2 × 10 mL) and desiccated overnight to yield compound **34** (85 mg, 0.110 mmol, 80.7%). Complex is unstable in solution. Partial characterization.

¹H NMR (800 MHz, CD_3CN, δ, 25°C) 8.38 (1H, d, TpB3), 8.08 (1H, d, TpB5), 8.00 (1H, d, TpC3), 7.97 (1H, d, TpA3), 7.84 (1H, d, TpA5), 7.83 (1H, d, TpC5), 6.53 (2H, t, TpB4/TpA4), 6.41 (1H, t, TpC4), 5.43 (1H, t, H1), 5.35 (1H, d, H2), 5.07 (1H, m, H6), 4.82 (1H, d, H3), 3.75 (3H, s, H8), 3.71 (1H, m, H12A), 3.55 (2H, m, H12B/H9A), 3.43 (2H, m, H9B/H7), 3.20 (2H, m, H5A/H5B), 2.87 (1H, m, H4), 1.80 (2H, m, H11A/H11B), 1.55 (2H, m, H10A/H10B), 1.14 (9H, d, PMe_3)

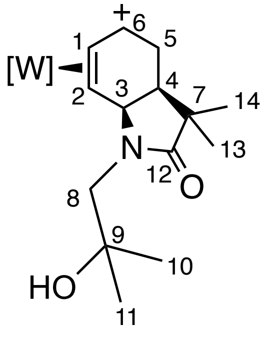

Compound 35:
Compound **26** (50 mg, 0.068 mmol) was placed in a test tube, with ACN (2 mL), and chilled to -30 °C. After 10 min, a 1 M HOTf/ACN (0.15 mL, 0.415 mmol) solution was added to the test tube and the solution was allowed to stir at -30 °C for 15 min. The reaction mixture was pipetted in 30

mL of stirring diethyl ether. A tan solid precipitated out and was collected on a 15 mL fine-porosity fritted disk, washed with hexane (2 × 10 mL) and desiccated overnight to yield compound **35** (40 mg, 0.054 mmol, 79.8%). Complex is unstable in solution. Partial characterization.

^{1}H NMR (800 MHz, CD_3CN, δ, 25°C) 8.59 (1H, d, TpB3), 8.36 (1H, d, TpB5), 8.00 (1H, d, TpC3), 7.95 (1H, d, TpA3), 7.84 (2H, d, TpA5/TpC5), 6.51 (2H, t, TpB4/TpA4), 6.38 (1H, t, TpC4), 5.43 (1H, t, H1), 5.35 (1H, d, H2), 5.07 (1H, m, H6), 4.82 (1H, d, H3), 3.68 (1H, dd, H8A), 3.37 (1H, m, H5A), 3.24 (1H, dd, H8B), 3.13 (1H, m, H5B), 2.88 (1H, m, H4), 1.18 (21H, m, H14/H13/H12/H11/PMe_3).

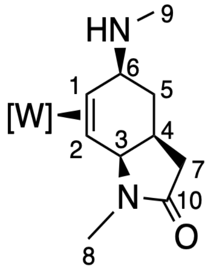

Compound 36:

Compound **27** (100 mg, 0.153 mmol) was placed in a test tube, with THF (2 mL), and chilled to -60 °C. In a separate test tube, methylamine in THF (0.766 mL, 2 M) was cooled at -60 °C for 10 min. After the time elapsed, the former solution was added to the latter, dropwise. The reaction stirred cold for 10 min and then quenched with a solution of sodium tert-butoxide (0.265 mL, 0.459 mmol, 20%). The solution was evaporated in vacuo. The resulting yellow film was dissolved in minimal THF and pipetted in 15 mL of cold stirring pentane. A tan solid precipitated out and was collected on a 15 mL fine-porosity fritted disk, washed with pentane (2 × 10 mL) and desiccated overnight to yield compound **36** (78 mg, 0.110 mmol, 75%).

^{1}H-NMR (800 MHz, $(CD_3)_2CO$, δ, 25 ºC): 8.27 (TpA3), 8.14 (1H, d, TpB3), 7.94 (1H, d, TpB5), 7.93 (1H, d, TpC5), 7.84 (1H, d, TpA5), 7.59 (1H, d, TpC3), 6.41 (1H, t, TpB4), 6.34 (1H, t, Tpc4), 6.29 (1H, t, TpA4), 4.80 (1H, d, H3), 3.52 (1H, m, H6), 2.57 (1H, m, H4), 2.49 (1H, dd, H7), 2.46 (1H, s, H8), 2.48 (1H, m, H1), 2.45 (1H, s, H9), 2.28 (1H, d, H7), 1.96 (1H, m, H5), 1.39 (1H, d, H2), 1.27 (1H, m, H5), 1.27 (9H, s, PMe3), 1.16 (2H, m, H5/H2). **^{13}C-NMR (201 MHz, $(CD_3)_2CO$, δ, 25 ºC):** 174.4 (1C, C10), 144.4 (1C, TpA3), 142.9 (1C, TpB3), 141.8 (1C, TpB5), 137.7 (1C, TpA5), 137.3 (1C, TpC5), 136.9 (1C, TpC3), 107.1 (1C, TpB4), 106.7 (1C, TpC4) 106.1 (1C, TpA4), 62.9 (1C, C3), 58.9 (1C, C6), 56.9 (1C, C1), 54.3 (1C, C4), 47.7 (1C, C2), 39.3 (1C, C7), 33.0 (1C, C9), 31.6 (1C, C5), 26.1 (1C, C8), 13.8 (3C, d J= 29.3 Hz, PMe3).

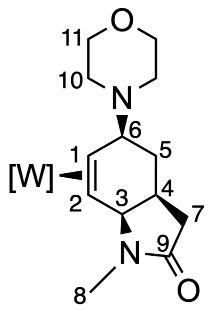

Compound 37:
Compound **27** (100 mg, 0.153 mmol) was placed in a test tube, with THF (2 mL), and chilled to -60 °C. In a separate test tube, morpholine (0.132 mL, 1.53 mmol) with THF (2 mL) was cooled at -60 °C for 10 min. After the time elapsed, the former solution was added to the latter, dropwise. The reaction stirred cold for 10 min and then quenched with a solution of sodium tert-butoxide (0.265 mL, 0.459 mmol, 20%). The solution was evaporated in vacuo. The resulting yellow film was dissolved in minimal THF and pipetted in 15 mL of cold stirring pentane. A tan solid precipitated out and was collected on a 15 mL fine-porosity fritted disk, washed pentane (2 × 10 mL) desiccated overnight to yield compound **37** (73 mg, 0.099, 64%).

^{1}H-NMR (800 MHz, CD_3CN C δ, 25 ºC): 8.18 (TpA3), 8.04 (1H, d, TpB3), 7.86 (1H, d, TpB5), 7.85 (1H, d, TpC5), 7.79 (1H, d, TpA5), 7.46 (1H, d, TpC3), 6.38 (1H, t, TpB4), 6.29 (1H, t, Tpc4), 6.24 (1H, t, TpA4), 4.71 (1H, d, H3), 3.73 (1H, m, H6), 3.68 (2H, m, H10), 2.98 (1H, m, H9), 2.64 (1H, m, H1), 2.58 (1H, dd, H7), 2.49 (1H, m, H9), 2.46 (1H, s, H8), 2.39 (1H, m, H4), 1.90 (1H, m, H7), 1.44 (1H, m, H5), 1.24 (9H, s, PMe3), 1.16 (2H, m, H5/H2). **^{13}C-NMR (201 MHz, CD_3CN C δ, 25 ºC)**: 175.8 (1C, C11), 144.6 (1C, TpA3), 143.2 (1C, TpB3), 142.4 (1C, TpB5), 138.1 (1C, TpC5), 137.9 (1C, TpA5), 137.4 (1C, TpC3), 106.6 (1C, TpB4), 106.0 (1C, TpC4) 105.5 (1C, TpA4), 66.9 (2C, C10), 63.8 (1C, C6), 62.8 (1C, C3), 53.4 (1C, C1), 48.6 (1C, C9), 47.8 (1C, C2), 39.4 (1C, C7), 30.5 (1C, C4), 26.5 (1C, C8), 25.5 (1C, C5), 13.4 (3C, d J= 30.23 Hz, PMe3).

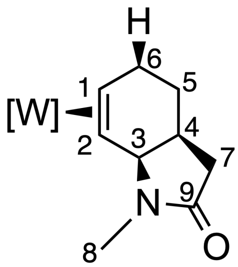

Compound 38:
Compound **27** (60 mg, 0.092 mmol) was placed in a test tube, with ACN (2 mL), and chilled to -30 °C. In a separate test tube, tetrabutylammonium borohydride (0.71 mg, 0.276 mmol) with ACN (2 mL) was cooled at -30 °C for 10 min. After the time elapsed, the former solution was added to the latter, dropwise and stirred for 15 min. The reaction was washed three times (H_2O:Na_2CO_3/DCM; 30 mL/30mL) and dried over anhydrous Mg_2SO_4. The clear solution was evaporated in vacuo. The resulting film was dissolved in minimal DCM and pipetted in 15 mL of stirring hexane. A white solid precipitated out and was collected on a 15 mL fine-porosity fritted

disk, washed with hexane (2 × 10 mL) and desiccated overnight to yield compound **38** (46.3 mg, 0.071 mmol, 77.2%).

^{1}H-NMR (800 MHz, CD_3CN C δ, 25 ºC): 8.29 (1H, d, TpA3), 8.06 (1H, d, TpB3), 7.86 (1H, d, TpB5), 7.84 (1H, d, TpA5), 7.81 (1H, d, TpC5), 7.45 (1H, d, TpC3), 6.38 (1H, t, TpB4), 6.29 (1H, t, TpA4), 6.27 (1H, t, TpC4), 4.82 (1H, d, H3), 3.08 (1H, m, H6), 2.72 (1H, m, H1), 2.50 (1H, m, H6), 2.48 (3H, s, H8), 2.40 (1H, m, H4) 2.38 (1H, dd, J= 15.47 Hz, H7), 2.09 (1H, H7), 1.66 (1H, ddd J= 12.68 Hz, H5), 1.35 (1H, ddd, H5), 1.15 (1H, d, H2). **^{13}C-NMR (201 MHz, CD_3CN C δ, 25 ºC):** 175.4 (1C, C9), 144.3 (1C, TpA5), 142.9 (1C, TpA3), 142.1 (1C, TpB3) 137.9 (1C, TpC3), 137.7 (1C, TpC5), 137.3 (1C, TpB5), 107.6 (1C, TpA4), 107.1 (1C, Tp4C) 106.5 (1C, Tp4B), 63.8 (1C, C3), 52.4 (1C, C1), 49.5 (1C, C2), 38.3 (1C, C7), 32.8 (1C, C4), 28.0 (1C, C8), 27.6 (1C, J= 4.48, C6), 27.5 (1C, J= 8.89 J, C5), 13.7 (3C, d J= 29.11 Hz, PMe3).

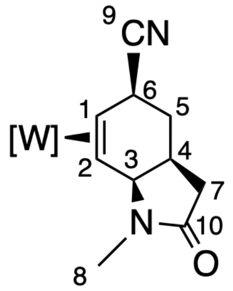

Compound 39:

Compound **27** (60 mg, 0.092 mmol) was placed in a test tube, with ACN, and chilled to -30 °C. In a separate test tube, NaCN (13.5 mg, 0.276 mmol) with MeOH (2 mL) was cooled at -30 °C for 10 min. After the time elapsed, the former solution was added to the latter, dropwise and stirred for 20 min. The reaction was washed three times (H_2O:Na_2CO_3/DCM; 30 mL/30mL) and dried over anhydrous Mg_2SO_4. The clear solution was evaporated in vacuo. The resulting film was dissolved in minimal DCM and pipetted in 15 mL of stirring hexane. A white solid precipitated out and was collected on a 15 mL fine-porosity fritted disk, washed with hexane (2 × 10 mL) and desiccated overnight to yield compound **39** (47.4 mg, 0.070 mmol, 75.6%).

^{1}H-NMR (800 MHz, CD_3CN C δ, 25 ºC): 8.13 (1H, d, TpA3), 8.06 (1H, d, TpB3), 7.87 (1H, d, TpB5), 7.87 (1H, d, TpC5), 7.81 (1H, d, TpA5), 7.47 (1H, d, TpC3), 6.39 (1H, t, TpB4), 6.30 (1H, t, TpC4), 6.27 (1H, t, TpA4), 4.73 (1H, d, H3), 3.68 (1H, q, H6), 2.72 (1H, m, H1), 2.57 (1H, d, H7), 2.52 (1H, m, H4), 2.48 (3H, s, H8), 2.03 (1H, m, H7), 2.03 (1H, ddd J= 13.59 Hz, H5), 1.58 (1H, ddd, H5), 1.15 (1H, d, H2). **^{13}C-NMR (201 MHz, CD_3CN C δ, 25 ºC):** 175.2 (1C, C10), 144.8 (1C, TpA5), 143.5 (1C, TpA3), 142.3 (1C, TpB3) 138.3 (1C, TpC3), 137.9 (1C, TpC5), 137.9 (1C, TpB5), 137.7 (1C, C9), 107.8 (1C, TpA4), 107.3 (1C, Tp4C) 106.8 (1C, Tp4B), 62.6 (1C, C3), 49.6 (1C, C1), 47.4 (1C, C2), 39.3 (1C, C7), 31.30 (1C, C4), 30.91 (1C, C6), 30.18 (1C, C7), 29.63 (1C, J= 3.72, C5), 27.5 (1C, C8), 13.20 (3C, d J= 29.08 Hz, PMe3).

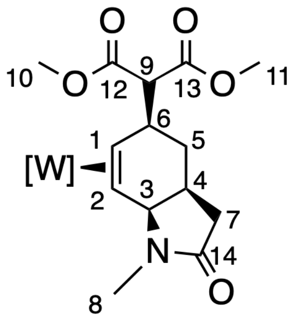

Compound 40:

Compound **27** (110 mg, 0.168 mmol) was placed in a test tube, with ACN (2 mL), and chilled to -30 °C. Lithium dimethyl malonate (70 mg, 0.504 mmol) with ACN (2 mL) was cooled in a separate test tube, at -30 °C for 10 min. After the time elapsed, the former solution was added to the latter, dropwise. The reaction stirred at -30 °C for 10 min. The reaction was washed three times (H_2O:Na_2CO_3/DCM; 30 mL/30mL) and dried over anhydrous Na_2SO_4. The clear solution was evaporated in vacuo. The resulting yellow film was dissolved in minimal DCM and pipetted in 50 mL of stirring pentane. A white solid precipitated out and was collected on a 15 mL fine-porosity fritted disk, washed with pentane (2 × 10 mL) and desiccated overnight to yield compound **40** (97 mg, 0.12 mmol, 74%).

^{1}H-NMR (800 MHz, CD_3CN, δ, 25 ºC): 8.12 (1H, d, TpB3), 7.98 (1H, d, TpA3), 7.87 (1H, d, TpB5), 7.85 (1H, d, TpC5), 7.76 (1H, d, TpA5), 7.28 (1H, d, TpC3), 6.37 (1H, t, TpB4), 6.31 (1H, t, TpC4), 6.25 (1H, t, TpA4), 4.48 (1H, dd, H3), 3.75 (1H, s, H10), 3.69 (4H, m, H11/9), 3.22 (1H, m, H6), 2.64 (1H, m, H7), 2.61 (3H, s, H8), 2.62 (1H, m, H4), 2.24 (1H, m, H5), 2.15 (1H, m, H1) 1.86 (1H, d, H7), 1.33 (1H, dt, H2), 1.19 (9H, PMe3), 1.07 (1H, d, H5). **^{13}C-NMR (201 MHz, CD_3CN, δ, 25 ºC):** 175.6 (1C, C14), 171.0 (1C, C12), 170.7 (1C, C13), 144.7 (1C, TpB3), 144.2 (1C, TpA3), 142.0 (1C, TpC3) 138.4 (1C, TpB5), 137.5 (2C, TpA5/C5), 107.5 (1C, TpB4), 107.4 (1C, TpC4) 107.0 (1C, TpA4), 63.7 (1C, C3), 63.2 (1C, C9), 53.8 (1C, C1), 52.9 (2C, C10/11), 47.5 (1C, C2), 43.5 (1C, C7), 39.4 (1C, C6), 31.4 (1C, C5), 27.3 (1C, C8), 27.2 (1C, C4), 13.5 (3C, d J= 29.11 Hz, PMe3).

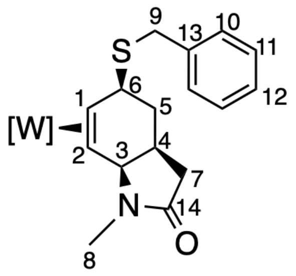

Compound 41:

Compound **27** (25.0 mg; 0.038 mmol) was placed in a test tube, with ACN (2 mL), and chilled to -30 °C. NaOtBu (15.0 mg; 0.156 mmol) and Phenylmethanethiol (0.027 mL, 0.230 mmol) with ACN (2 mL) was cooled in a separate test tube, at -30 °C for 10 min. After the time elapsed, the former solution was added to the latter, dropwise. The reaction stirred at -30 °C for 1 h. The reaction was washed three times (H_2O:Na_2CO_3/DCM; 30 mL/30mL) and dried over anhydrous

Na_2SO_4. The clear solution was evaporated in vacuo. The resulting yellow film was dissolved in minimal DCM and pipetted in 50 mL of stirring pentane. A white solid precipitated out and was collected on a 15 mL fine-porosity fritted disk, washed with pentane (2 × 10 mL) and desiccated overnight to yield compound **41** (97 mg, 0.12 mmol, 74%).

^{1}H NMR (800 MHz, $(CD_3)_2CO$, δ, 25 °C): 8.19 (d, J = 2.1 Hz, 1H), 8.07 (d, J = 2.0 Hz, 1H), 7.94 (d, J = 2.3 Hz, 1H), 7.92 (d, J = 2.4 Hz, 1H), 7.84 (d, J = 2.4 Hz, 1H), 7.44 (d, J = 2.2 Hz, 1H), 7.42 (d, J = 6.8 Hz, 1H), NaN (m, 1H), 7.34 (t, J = 7.7 Hz, 1H), 7.23 (tt, J = 7.3, 1.4 Hz, 1H), 6.38 (t, J = 2.2 Hz, 1H), 6.32 (t, J = 2.2 Hz, 1H), 6.29 (t, J = 2.2 Hz, 1H), 4.73 (dt, J = 6.9, 1.5 Hz, 1H), 3.88 (d, J = 2.1 Hz, 2H), 3.78 (m, 1H), 2.69 (dd, J = 16.1, 4.9 Hz, 1H), 2.64 (dtt, J = 8.6, 6.7, 5.2 Hz, 1H), 2.52 (s, 2H), 2.43 (m, 2H), 2.30 (m, 1H), 1.78 (dt, J = 14.5, 5.0 Hz, 1H), 1.22 (dt, J = 11.1, 1.6 Hz, 1H), 0.98 (d, J = 8.4 Hz, 8H). **^{13}C NMR (201 MHz, $(CD_3)_2CO$, δ, 25 °C):** 174.4, 144.2, 143.5, 142.0, 140.6, 137.9, 137.4, 137.1, 130.1, 129.3, 127.5, 107.3, 107.0, 106.4, 62.4, 54.1 (d, J = 10.9 Hz), 49.1, 43.9 (d, J = 3.7 Hz), 40.1, 36.6, 30.5, 30.3, 27.3, 13.9 (d, J = 27.6 Hz).

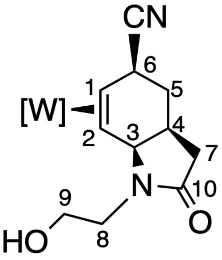

Compound 42:

Compound **28** (120 mg, 0.176 mmol) was placed in a test tube, with ACN (2 mL), and chilled to -30 °C. NaCN (26 mg, 0.527 mmol) with MeOH (2 mL) was cooled in a separate test tube, at -30 °C for 10 min. After the time elapsed, the former solution was added to the latter, dropwise. The reaction stirred at -30 °C for 10 min. The reaction was washed three times (H_2O:Na_2CO_3/DCM; 30 mL/30mL), and dried over anhydrous Na_2SO_4. The clear solution was evaporated in vacuo. The resulting yellow film was dissolved in minimal DCM and pipetted in 50 mL of stirring pentane. A white solid precipitated out and was collected on a 15 mL fine-porosity fritted disk, washed with pentane (2 × 10 mL) and desiccated overnight to yield compound **42** (102.0 mg, 0.144 mmol, 81.9%).

^{1}H-NMR (800 MHz, CD_3CN, δ, 25 °C): 8.07 (1H, d, TpB3), 8.05 (1H, d, TpA3), 7.88 (2H, d, TpC5/B5), 7.81 (1H, d, TpA5), 7.47 (1H, d, TpC3), 6.38 (1H, t, TpB4), 6.31 (1H, t, TpC4), 6.28 (1H, t, TpA4), 4.78 (1H, d, H3), 3.68 (1H, m, H9), 3.28 (1H, m, H8), 3.24 (1H, m, H9), 3.16 (1H, m, H6), 2.99 (1H, m, H8), 2.73 (1H, m, H4), 2.59 (1H, dd, H7), 2.53 (2H, m, H5/H1), 1.99 (1H, m, H7), 1.59 (1H, m, H5), 1.18 (1H, dd, H2), 1.10 (9H, d, PMe3), 1.08 (1H, d, H5). **^{13}C-NMR (201 MHz, CD_3CN, δ, 25 °C):** 176.4 (1C, C15), 144.6 (1C, TpB3), 143.68 (1C, TpA3), 142.3 (1C, TpC3) 138.0 (1C, TpC5), 137.7 (1C, TpB5), 129.9 (1C, TpC5), 129.2 (1C, CN), 106.9 (3C, TpA4/B4/C4), 66.3 (1C, C3), 61.7 (1C, C9), 49.0 (1C, C1), 47.7 (1C, C2), 43.7 (1C, C8), 31.3 (1C, C7), 31.1 (1C, C6), 29.5 (1C, C5), 15.6 (1C, C4), 13.8 (3C, d J= 27.19 Hz, PMe3).

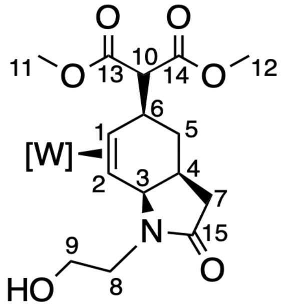

Compound 43:

Compound **28** (120 mg, 0.176 mmol) was placed in a test tube, with ACN (2 mL), and chilled to -30 °C. Lithium dimethyl malonate (73 mg, 0.527 mmol) with ACN (2 mL) was cooled in a separate test tube, at -30 °C for 10 min. After the time elapsed, the former solution was added to the latter, dropwise. The reaction stirred at -30 °C for 10 min. The reaction was washed three times (H_2O:Na_2CO_3/DCM; 30 mL/30mL) and dried over anhydrous Na_2SO_4. The clear solution was evaporated in vacuo. The resulting yellow film was dissolved in minimal DCM and pipetted in 50 mL of stirring pentane. A white solid precipitated out and was collected on a 15 mL fine-porosity fritted disk, washed with pentane (2 × 10 mL) and desiccated overnight to yield compound **43** (120.0 mg, 0.176 mmol, 84.0%).

^{1}H-NMR (800 MHz, CD_3CN, δ, 25 ºC): 8.12 (1H, d, TpB3), 7.99 (1H, d, TpA3), 7.87 (1H, d, TpC5), 7.83 (1H, d, TpB5), 7.77 (1H, d, TpA5), 7.29 (1H, d, TpC3), 6.37 (1H, t, TpB4), 6.31 (1H, t, TpC4), 6.26 (1H, t, TpA4), 4.70 (1H, d, H3), 3.71 (4H, m, H9/H10/12), 3.70 (3H, s, H11), 3.45 (1H, m, H8), 3.38 (1H, m, H9), 3.22 (1H, m, H6), 3.12 (1H, m, H8), 2.74 (1H, dd, H7), 2.66 (1H, m, H4), 2.19 (2H, m, H5/H1), 2.12 (1H, m, H7), 1.34 (1H, dd, H2), 1.18 (9H, d, PMe3), 1.08 (1H, d, H5).
^{13}C-NMR (201 MHz, CD_3CN, δ, 25 ºC): 176.8 (1C, C15), 170.9 (1C, C13), 170.7 (1C, C14), 144.6 (1C, TpB3), 144.0 (1C, TpA3), 142.3 (1C, TpC3) 138.3 (1C, TpC5), 137.4 (2C, TpB5/C5), 107.5 (1C, TpB4), 107.4 (1C, TpC4) 106.9 (1C, TpA4), 63.1 (1C, C10), 62.3 (1C, C3), 60.5 (1C, C9), 53.9 (1C, C1), 52.8 (2C, C11/12), 47.5 (1C, C2), 43.5 (1C, C8), 43.2 (1C, C7), 39.3 (1C, C6), 31.2 (1C, C5), 27.3 (1C, C4), 13.5 (3C, d J= 27.19 Hz, PMe3).

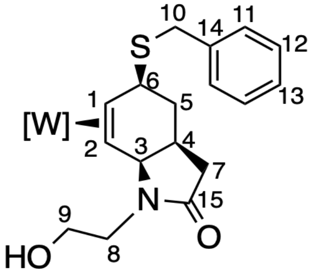

Compound 44:

Compound **28** (40 mg, 0.059 mmol) was placed in a test tube, with ACN (2 mL), and chilled to -30 °C. Phenylmethanethiol (0.021 mL, 0.180 mmol) was cooled in a separate test tube, at -30 °C for 10 min with potassium tert-butoxide (0.071 mL, 0.120 mmol). After the time elapsed, the former solution was added to the latter, dropwise. The reaction stirred at -30 °C for 10 min. The reaction was washed three times (H_2O/DCM; 30 mL/30mL) and dried over anhydrous Na_2SO_4. The clear

solution was evaporated in vacuo. The resulting yellow film was dissolved in minimal DCM and pipetted in 50 mL of stirring pentane. A white solid precipitated out and was collected on a 15 mL fine-porosity fritted disk, washed pentane (2 × 10 mL) desiccated overnight to yield compound **44** (32.0 mg, 0.059, 68.0%).

^{1}H-NMR (800 MHz, CD_3CN, δ, 25 ºC): 8.06 (1H, d, TpA3), 8.00 (1H, d, TpB3), 7.78 (2H, t, TpC5/A5), 7.78 (1H, d, TpB5), 7.40-7.22 (5H, m, Ph), 7.32 (1H, d, TpC3), 6.34 (1H, t, TpB4), 6.28 (1H, t, TpC4), 6.25 (1H, t, TpA4), 4.75 (1H, dd, H3), 3.87 (1H, s, H11), 3.71 (1H, t, H6), 3.39 (2H, m, H8), 3.20 (1H, m, H9), 3.01 (1H, m, H9), 2.64 (1H, m, H4), 2.59 (2H, m, H7), 2.41 (1H, dt, H1), 2.28 (1H, m, H5) 1.73 (1H, dt, H5), 1.14 (1H, dt, H2), 0.91 (9H, PMe3). **^{13}C-NMR (201 MHz, CD_3CN, δ, 25 ºC):** 176.9 (1C, C10), 144.3 (1C, TpA3), 143.8 (1C, TpB3), 142.2 (1C, TpC5) 138.1 (1C, TpA5), 137.3 (2C, TpB5/C3), 129-126 (6C, Ph), 106.7 (1C, TpB4), 106.2 (1C, TpC4) 105.9 (1C, TpA4), 61.1 (1C, C3), 60.5 (1C, C8), 53.3 (1C, C1), 48.2 (1C, C2), 43.2 (1C, C9), 42.8 (1C, C6), 40.0 (1C, C7), 35.8 (1C, C11), 30.1 (1C, C5), 28.6 (1C, C4), 12.8 (3C, d J= 28.07 Hz, PMe3).

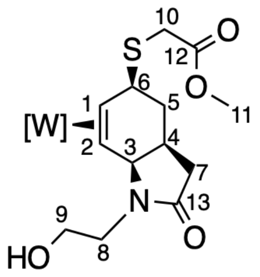

Compound 45:

Compound **28** (85 mg, 0.124 mmol) was placed in a test tube, with ACN (2 mL), and chilled to -30 °C. Methyl 2-mercaptoacetate (0.034 mL, 0.373 mmol) was cooled in a separate test tube, at -30 °C for 10 min with potassium tert-butoxide (0.143 mL, 0.249 mmol). After the time elapsed, the former solution was added to the latter, dropwise. The reaction stirred at -30 °C for 10 min. The reaction was washed three times (H_2O/DCM; 30 mL/30mL) and dried over anhydrous Na_2SO_4. The clear solution was evaporated in vacuo. The resulting white film was dissolved in minimal DCM and pipetted in 50 mL of stirring pentane. A white solid precipitated out and was collected on a 15 mL fine-porosity fritted disk, washed with pentane (2 × 10 mL) and desiccated overnight to yield compound **45** (72.0 mg, 0.124, 73.0%).

^{1}H-NMR (800 MHz, CD_3CN, δ, 25 ºC): 8.06 (1H, d, TpB3), 8.04 (1H, d, TpA3), 7.86 (2H, t, TpC5/B5), 7.79 (1H, d, TpA5), 7.48 (1H, d, TpC3), 6.37 (1H, t, TpB4), 6.29 (1H, t, TpC4), 6.27 (1H, t, TpA4), 4.76 (1H, m, H3), 4.10 (1H, m, H6), 3.70 (3H, s, H11), 3.46 (1H, d, H10), 3.37 (3H, m, H10/9), 3.27 (1H, m, H8), 2.99 (1H, m, H8), 2.64 (1H, m, H4), 2.60 (1H, m, H7), 2.56 (1H, m, H1), 2.39 (1H, d, H7), 2.29 (1H, m, H5), 1.63 (1H, d, H5), 1.18 (1H, d, H2), 1.17 (9H, d, PMe3). **^{13}C-NMR (201 MHz, CD_3CN, δ, 25 ºC):** 176.7 (1C, C13), 172.5 (1C, C12), 144.4 (1C, TpB3), 143.8 (1C, TpA3), 142.3 (1C, TpC3), 138.2 (1C, TpB5) 137.8 (1C, TpC5), 137.4 (1C, TpA5), 107.6 (1C, TpB4), 107.3 (1C, TpC4) 106.9 (1C, TpA4), 62.0 (1C, C3), 61.2 (1C, C9), 53.8 (1C, C1), 52.9

(1C, C11), 48.9 (1C, C2), 46.4 (1C, C6), 44.1 (1C, C8), 41.1 (1C, C7), 34.3 (1C, C10), 31.5 (1C, C5), 29.4 (1C, C4), 13.8 (3C, d J= 29.45 Hz, PMe3).

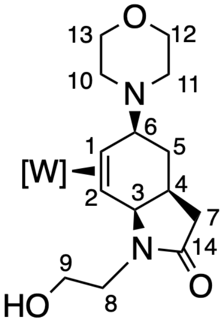

Compound 46:
Compound **28** (100 mg, 0.146 mmol) was placed in a test tube, with THF (2 mL), and chilled to -60 °C. In a separate test tube, morpholine (0.126 mL, 1.46 mmol) with THF (2 mL) was cooled at -60 °C for 10 min. After the time elapsed, the former solution was added to the latter, dropwise. The reaction stirred cold for 10 min and then quenched with a solution of sodium tert-butoxide (0.253 mL, 0.439 mmol, 20%). The solution was evaporated in vacuo. The resulting yellow film was dissolved in minimal THF and pipetted in 15 mL of cold stirring pentane. A tan solid precipitated out and was collected on a 15 mL fine-porosity fritted disk, washed with pentane (2 × 10 mL) and desiccated overnight to yield compound **46** (97 mg, 0.13 mmol, 86%).

^{1}H-NMR (800 MHz, CD_3CN, δ, 25 C): 8.09 (TpA3), 8.03 (1H, d, TpB3), 7.85 (1H, d, TpB5), 7.85 (1H, d, TpC5), 7.79 (1H, d, TpA5), 7.46 (1H, d, TpC3), 6.36 (1H, t, TpB4), 6.30 (1H, t, Tpc4), 6.25 (1H, t, TpA4), 4.75 (1H, d, H3), 3.76 (1H, m, H6), 3.69 (2H, m, H12), 3.56 (1H, m, H9), 3.26 (1H, m, H9), 3.21 (2H, m, H8), 3.08 (1H, m, H10), 2.98 (1H, m, H1), 2.61 (1H, dd, H7), 2.51 (1H, m, H4), 2.46 (2H, m, H11), 1.92 (1H, m, H7), 1.41 (1H, m, H2), 1.24 (2H, m, H5), 1.24 (9H, s, PMe3), 1.09 (2H, m, H5/H2). **^{13}C-NMR (201 MHz, CD_3CN, δ, 25 C)**: 177.2 (1C, C14), 144.6 (1C, TpB3), 143.4 (1C, TpA3), 142.4 (1C, TpB5), 137.9 (2C, TpC5/A5), 137.4 (1C, TpA5), 107.6 (1C, TpC3), 107.1 (1C, TpB4), 106.7 (1C, TpC4), 68.1 (2C, C12/C13), 64.4 (1C, C6), 62.7 (1C, C3), 61.2 (1C, C9), 53.7 (1C, C1), 49.0 (1C, C2), 48.9 (1C, C10/C11), 43.8 (1C, C8), 40.4 (1C, C7), 32.1 (1C, C4), 31.4 (1C, C5), 14.4 (3C, d J= 30.23 Hz, PMe3).

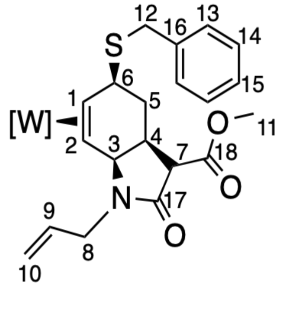

Compound 47:

Compound **30** (100 mg, 0.136 mmol) was placed in a test tube, with ACN (2 mL), and chilled to -30 °C. Phenylmethanethiol (0.048 mL, 0.407 mmol) was cooled in a separate test tube, at -30 °C for 10 min in potassium tert-butoxide (0.164 mL, 0.271 mmol). After the time elapsed, the former solution was added to the latter, dropwise. The reaction stirred at -30 °C for 10 min. The reaction was washed three times (H_2O/DCM; 30 mL/30mL) and dried over anhydrous Na_2SO_4. The clear solution was evaporated in vacuo. The resulting yellow film was dissolved in minimal DCM and pipetted in 50 mL of stirring pentane. A white solid precipitated out and was collected on a 15 mL fine-porosity fritted disk, washed pentane (2 × 10 mL) desiccated overnight to yield compound **47** (92 mg, 0.11 mmol, 79%).

^{1}H NMR (800 MHz, CD_3CN, δ, 25 ºC): 7.97 (t, *J* = 2.7 Hz, 2H, TpA3/TpB3), 7.83 (t, *J* = 2.2 Hz, 2H, TpB5/TpC5), 7.79 (d, *J* = 2.4 Hz, 1H, TpA5), 7.38 (dt, *J* = 8.0, 1.7 Hz, 2H, H13), 7.35 (dd, *J* = 8.6, 6.9 Hz, 2H, H14), 7.32 (d, *J* = 2.3 Hz, 1H, TpC3), 7.24 (tt, *J* = 7.2, 1.4 Hz, 1H, H15), 6.34 (t, *J* = 2.2 Hz, 1H, TpB4), 6.27 (t, *J* = 2.2 Hz, 1H, TpA4), 6.24 (t, *J* = 2.2 Hz, 1H, TpC4), 5.57 (ddt, *J* = 17.3, 9.8, 4.8 Hz, 1H, H9), 4.93 (d, *J* = 7.7 Hz, 1H, H3), 4.88 (dq, *J* = 10.3, 1.7 Hz, 1H, H10A), 4.81 (dq, *J* = 17.3, 1.8 Hz, 1H, H10B), 4.40 (d, *J* = 7.2 Hz, 1H, H7), 3.91 (ddt, *J* = 17.2, 4.9, 2.3 Hz, 1H, H8A), 3.86 (s, 2H, H12), 3.77 (d, *J* = 1.2 Hz, 1H, H11), 3.76 (m, 1H, H6), 3.26 (dd, *J* = 16.6, 5.4 Hz, 1H, H8B), 2.91 (qd, *J* = 7.2, 3.3 Hz, 1H, H4), 2.34 (m, 2H, H1/H5A), 1.06 (dt, *J* = 11.3, 1.7 Hz, 1H, H2), 0.88 (dd, *J* = 8.3, 1.3 Hz, 9H, PMe3). **^{13}C NMR (201 MHz, CD_3CN, δ, 25 ºC):** 172.7 (1C, C17), 170.4 (1C, C18), 144.2 (1C, TpB3), 143.3 (1C, TpA3), 142.1 (1C, TpC3), 138.1 (1C, TpC5), 137.8 (1C, TpA5), 137.4 (1C, TpB5), 134.2 (1C, C9), 130.1 (1C, C13), 129.5 (1C, C14), 127.8 (1C, C15), 115.8 (1C, C10), 107.6 (1C, TpB4), 107.2 (1C, TpA4), 106.8 (1C, TpC4), 59.5 (1C, C3), 56.0 (1C, C7), 53.8 (1C, C1), 52.9 (1C, C11), 49.3 (1C, C2), 43.3 (1C, C6), 43.1 (1C, C8), 36.8 (1C, C12), 35.0 (1C, C4), 26.6 (1C, C5), 13.7 (3C, PMe3).

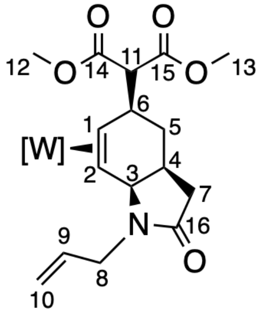

Compound 48:

Compound **31** (125 mg, 0.184 mmol) was placed in a test tube, with ACN (2 mL), and chilled to -30 °C. Lithium dimethyl malonate (76.2 mg, 0.552 mmol) with ACN (2 mL) was cooled in a separate test tube, at -30 °C for 10 min. After the time elapsed, the former solution was added to the latter, dropwise. The reaction stirred at -30 °C for 10 min. The reaction was washed three times (H_2O:Na_2CO_3/DCM; 30 mL/30mL) and dried over anhydrous Na_2SO_4. The clear solution was evaporated in vacuo. The resulting film was dissolved in minimal DCM and pipetted in 50 mL of stirring pentane. A white solid precipitated out and was collected on a 15 mL fine-porosity fritted disk, washed with pentane (2 × 10 mL) and desiccated overnight to yield compound **48** (110 mg, 0.130 mmol, 71%).

^{1}H NMR (800 MHz, CD_3CN, δ, 25 ºC): 8.14 (d, *J* = 2.0 Hz, 1H, TpB3), 7.98 (d, *J* = 2.1 Hz, 1H, TpA3), 7.88 (d, *J* = 2.3 Hz, 2H, TpB5/TpC5), 7.77 (d, *J* = 2.4 Hz, 1H, TpA5), 7.32 (d, *J* = 2.2 Hz, 1H, TpC3), 6.40 (t, *J* = 2.2 Hz, 1H, TpB4), 6.34 (t, *J* = 2.2 Hz, 1H, TpC4), 6.25 (t, *J* = 2.2 Hz, 1H, TpA4), 5.59 (dddd, *J* = 17.0, 10.7, 6.5, 4.6 Hz, 1H, H9), 4.84 (dq, *J* = 10.3, 1.5 Hz, 1H, H10A), 4.80 (dq, *J* = 17.3, 1.7 Hz, 1H, H10B), 4.73 (dt, *J* = 7.2, 1.9 Hz, 1H, H3), 4.21 (m, 1H, H8A), 3.78 (s, 3H, H12), 3.73 (s, 3H, H13), 3.61 (m, 1H, H8B), 3.26 (m, 1H, H6), 2.74 (dd, *J* = 16.9, 9.8 Hz, 1H, H7A), 2.67 (m, 1H, H4), 2.23 (m, 2H, H5A/H1), 1.70 (m, 1H, H7B), 1.33 (ddt, *J* = 10.3, 8.8, 1.6 Hz, 1H, H2), 1.23 (d, *J* = 8.5 Hz, 9H, PMe3), 1.11 (m, 1H, H5B). **^{13}C NMR (201 MHz, CD_3CN, δ, 25 ºC):** 175.5 (1C, C16), 171.0 (1C, C12), 170.7 (1C, C13), 144.6 (1C, TpB3), 144.2 (1C, TpA3), 142.1 (1C, TpC3), 138.3 (1C, TpA5), 137.4 (1C, TpB5), 137.3 (1C, TpC5), 134.4 (1C, C9), 116.6 (1C, C10), 107.5 (1C, TpB4), 107.3 (1C, TpC4), 106.7 (1C, TpA4), 61.2 (1C, C3), 53.7 (1C, C1), 53.0 (1C, C12), 52.9 (1C, C13), 47.2 (1C, C2), 43.2 (1C, C7), 42.6 (1C, C8), 39.3 (1C, C6), 31.2 (1C, C5), 27.3 (1C, C4), 13.8 (3C, PMe3).

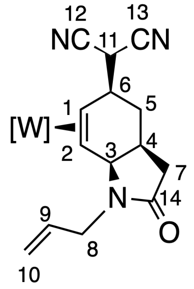

Compound 49:
Compound **31** (120 mg, 0.177 mmol) was placed in a test tube, with ACN (2 mL), and chilled to -30 °C. Lithium Malononitrile (55.2 mg, 0.530 mmol) with ACN (2 mL) was cooled in a separate test tube, at -30 °C for 10 min. After the time elapsed, the former solution was added to the latter, dropwise. The reaction stirred at -30 °C for 10 min. The reaction was washed three times (H_2O:Na_2CO_3/DCM; 30 mL/30mL) and dried over anhydrous Na_2SO_4. The clear solution was evaporated in vacuo. The resulting film was dissolved in minimal DCM and pipetted in 50 mL of stirring pentane. A white solid precipitated out and was collected on a 15 mL fine-porosity fritted disk, washed with pentane (2 × 10 mL) and desiccated overnight to yield compound **49** (93 mg, 0.130 mmol, 71%).

^{1}H NMR (800 MHz, CD_3CN, δ, 25 ºC): 8.12 (s, 1H, TpB3), 7.92 (s, 1H, TpA3), 7.89 (s, 1H, TpC5), 7.88 (s, 1H, TpB5), 7.77 (s, 1H, TpA5), 7.43 (s, 1H, TpC3), 6.39 (t, *J* = 1.9 Hz, 1H, TpB4), 6.34 (t, *J* = 2.2 Hz, 1H, TpC4), 6.25 (d, *J* = 2.1 Hz, 1H, TpA4), 5.58 (m, 1H, H9), 4.87 (m, 1H, H10A), 4.78 (m, 1H, H10B), 4.62 (d, *J* = 6.5 Hz, 1H, H3), 4.23 (dd, *J* = 10.0, 2.0 Hz, 1H, H11), 4.03 (dd, *J* = 15.5, 4.9 Hz, 1H, H8A), 3.60 (dd, *J* = 15.8, 6.7 Hz, 1H, H8B), 3.23 (s, 1H, H6), 2.76 (m, 1H, H7A), 2.71 (d, *J* = 8.4 Hz, 1H, H4), 2.50 (t, *J* = 11.2 Hz, 1H, H1), 2.43 (dt, *J* = 16.6, 8.2 Hz, 1H, H5A), 2.04 (d, *J* = 16.8 Hz, 1H, H7B), 1.48 (d, *J* = 15.7 Hz, 1H, H5B), 1.27 (d, *J* = 10.8 Hz, 1H, H2), 1.18 (dt, *J* = 8.4, 1.6 Hz, 9H, PMe3). **^{13}C NMR (201 MHz, CD_3CN, δ, 25 ºC):** 175.7 (1C, C14), 144.5 (1C, TpB3), 144.4 (1C, TpC5), 142.0 (1C, TpC3), 138.4 (1C, TpA3), 137.6 (1C, TpB5), 137.5 (1C, TpA5), 134.1 (1C, C9), 117.4 (1C, C10), 115.7 (1C, C12), 115.1 (1C, C13), 107.7 (1C, TpB4),

107.5 (1C, TpC4), 106.9 (1C, TpA4), 61.5 (1C, C3), 51.5 (1C, C1), 47.0 (1C, C2), 43.0 (1C, C8), 42.9 (1C, C7), 41.2 (1C, C6), 35.9 (1C, C11), 30.5 (1C, C5), 27.4 (1C, C4), 13.3 (3C, PMe3).

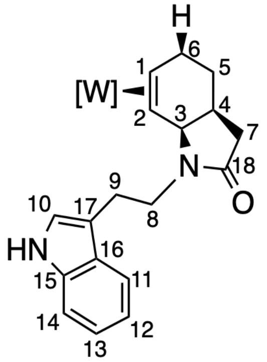

Compound 50:

Compound **32** (150 mg, 0.192 mmol) was placed in a test tube, with ACN (2 mL), and chilled to -30 °C. In a separate test tube, sodium borohydride (12 mg, 0.192 mmol) with ACN (2 mL) was cooled at -30 °C for 10 min. After the time elapsed, the former solution was added to the latter, dropwise and stirred for 15 min. The reaction was washed three times (H_2O:Na_2CO_3/DCM; 30 mL/30mL), and dried over anhydrous Mg_2SO_4. The clear solution was evaporated in vacuo. The resulting film was dissolved in minimal DCM and pipetted in 15 mL of stirring hexane. A white solid precipitated out and was collected on a 15 mL fine-porosity fritted disk, washed with hexane (2 × 10 mL) and desiccated overnight to yield compound **50** (120 mg, 0.150 mmol, 80%).

^{1}H NMR (800 MHz, CD_3CN, δ, 25 ºC): 8.21 (d, *J* = 2.1 Hz, 1H, TpA3), 8.05 (d, *J* = 2.0 Hz, 1H, TpB3), 7.92 (d, *J* = 2.3 Hz, 1H, TpC5), 7.86 (dt, *J* = 2.4, 0.7 Hz, 1H, TpB5), 7.75 (dd, *J* = 1.9, 1.2 Hz, 1H, TpA5), 7.45 (d, *J* = 2.2 Hz, 1H, TpC3), 7.28 (dt, *J* = 8.1, 0.9 Hz, 1H, H14), 7.00 (ddd, *J* = 8.2, 7.0, 1.2 Hz, 1H, H13), 6.85 (m, 1H, H10), 6.71 (ddd, *J* = 7.9, 6.9, 1.0 Hz, 1H, H12), 6.57 (m, 1H, H11), 6.38 (t, *J* = 2.2 Hz, 1H, TpB4), 6.26 (t, *J* = 2.2 Hz, 1H, TpC4), 6.17 (t, *J* = 2.2 Hz, 1H, TpA4), 4.91 (dd, *J* = 5.6, 2.1 Hz, 1H, H3), 3.37 (ddd, *J* = 13.5, 10.9, 6.2 Hz, 1H, H9A), 3.01 (m, 1H, H9B), 2.96 (m, 1H, H6A), 2.79 (m, 1H, H1), 2.58 (m, 3H, H8A/H8B/H6), 2.52 (dd, *J* = 16.0, 7.7 Hz, 1H, H7A), 2.44 (ddt, *J* = 12.3, 10.1, 4.7 Hz, 1H, H4), 2.03 (m, 1H, H7B), 1.55 (m, 1H, H5A), 1.32 (m, 3H, H5B/H2), 1.15 (d, *J* = 8.4 Hz, 9H, PMe3). **^{13}C NMR (201 MHz, CD_3CN, δ, 25 ºC):** 175.5 (1C, C18), 144.4 (1C, TpB3), 143.3 (1C, TpA3), 142.0 (1C, TpC3), 138.0 (1C, TpC5), 137.9 (1C, TpA5), 137.3 (1C, TpB5), 123.3 (1C, C10), 122.7 (1C, C13), 119.7 (1C, C12), 119.0 (1C, C11), 112.1 (1C, C14), 107.5 (1C, TpB4), 107.3 (1C, TpC4), 106.8 (1C, TpA4), 62.3 (1C, C3), 51.2 (1C, C1), 49.7 (1C, C2), 42.00 (1C, C9), 39.4 (1C, C7), 33.0 (1C, C4), 28.1 (1C, C6), 28.1 (1C, C5), 24.6 (1C, C8), 13.7 (3C, PMe3).

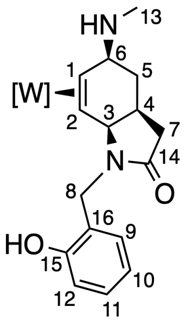

Compound 51:

Compound **33** (112 mg, 0.150 mmol) was placed in a test tube with THF (2 mL) and chilled to -60 °C. In a separate test tube, Methylamine (0.751 mL, 1.50 mmol) with THF (2 mL) was cooled at -60 °C for 10 min. After the time elapsed, the former solution was added to the latter, dropwise. The reaction stirred cold for 10 min and then quenched with a solution of sodium tert-butoxide (0.260 mL, 0.451 mmol, 20%). The solution was evaporated in vacuo. The resulting yellow film was dissolved in minimal THF and pipetted in 15 mL of cold stirring pentane. A white solid precipitated out and was collected on a 15 mL fine-porosity fritted disk, washed with pentane (2 × 10 mL) and desiccated overnight to yield compound **51** (85 mg, 0.110, 73%).

^{1}H NMR (800 MHz, C_4D_8O, δ, 25 ºC): 8.04 (d, *J* = 2.0 Hz, 1H, TpA3), 7.95 (d, *J* = 2.0 Hz, 1H, TpB3), 7.89 (d, *J* = 2.2 Hz, 1H, TpB5), 7.80 (d, *J* = 2.4 Hz, 1H, TpC5), 7.76 (d, *J* = 2.4 Hz, 1H, TpA5), 7.40 (d, *J* = 2.1 Hz, 1H, TpC3), 6.85 (td, *J* = 7.6, 1.7 Hz, 1H, H10), 6.59 (dd, *J* = 8.1, 1.3 Hz, 1H, H12), 6.28 (dt, *J* = 9.0, 2.2 Hz, 2H, TpB4/TpC4), 6.16 (td, *J* = 7.4, 1.4 Hz, 1H, H11), 6.13 (t, *J* = 2.1 Hz, 1H, TpA4), 5.86 (dd, *J* = 7.5, 1.8 Hz, 1H, H9), 4.77 (d, *J* = 5.0 Hz, 1H, H6), 4.14 (d, *J* = 14.8 Hz, 1H, H8A), 3.90 (d, *J* = 14.7 Hz, 1H, H8B), 3.48 (m, 1H, H3), 3.22 (d, *J* = 1.4 Hz, 3H, MeNH2), 2.51 (m, 3H, H7A/H5/H4), 2.32 (ddd, *J* = 13.4, 11.0, 2.8 Hz, 1H, H2), 2.27 (dd, *J* = 20.7, 6.2 Hz, 1H, H7B), 1.28 (d, *J* = 11.1 Hz, 1H, H1), 1.22 (d, *J* = 8.6 Hz, 9H, PMe3). **^{13}C NMR (201 MHz, C_4D_8O, δ, 25 ºC):** 176.3 (1C, C14), 144.4 (1C, TpA3), 141.8 (1C, TpB3), 137.3 (1C, TpB5), 136.9 (1C, TpC5), 136.7 (1C, TpA5), 129.0 (1C, C12), 128.5 (1C, C11), 122.8 (1C, C16), 121.2 (1C, C15), 119.5 (1C, C9) 106.8 (1C, TpB4), 106.7 (2C, TpB4/TpC4), 64.1 (1C, C6), 59.5 (1C, C3), 56.3 (1C, C2), 49.9 (2C, C1/MeNH2), 41.3 (2C, C7/C8), 31.8 (1C, C4), 29.9 (1C, C5), 14.1 (3C, PMe3).

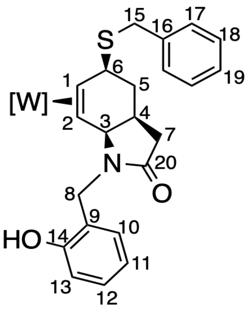

Compound 52:

Compound **33** (132 mg, 0.177 mmol) was placed in a test tube, with ACN (2 mL), and chilled to -30 °C. Phenylmethanethiolate (109 mg, 0.886 mmol) with ACN (2 mL) was cooled in a separate test tube, at -30 °C for 10 min. After the time elapsed, the former solution was added to the latter,

dropwise. The reaction stirred at -30 °C for 10 min. The reaction was washed three times (H_2O/DCM; 30 mL/30mL) and dried over anhydrous Na_2SO_4. The clear solution was evaporated in vacuo. The resulting yellow film was dissolved in minimal DCM and pipetted in 50 mL of stirring pentane. A white solid precipitated out and was collected on a 15 mL fine-porosity fritted disk, washed with pentane (2 × 10 mL) and desiccated overnight to yield compound **52** (105 mg, 0.121 mmol, 68%).

^{1}H NMR (800 MHz, CD_3CN, δ, 25 ºC): 7.98 (dd, *J* = 4.6, 2.1 Hz, 2H, TpA3/TpB3), 7.89 (d, *J* = 2.3 Hz, 1H, TpA5), 7.85 (dd, *J* = 7.1, 2.4 Hz, 2H, TpB5/TpC5), 7.34 (m, 4H, H17/H18), 7.23 (m, 2H, TpC3/H19), 6.97 (m, 1H, H12), 6.68 (dd, *J* = 8.1, 1.2 Hz, 1H, H13), 6.34 (t, *J* = 2.2 Hz, 1H, TpA4), 6.25 (td, *J* = 2.3, 1.2 Hz, 3H, TpB4/TpC4/H11), 5.83 (m, 1H, H10), 4.81 (d, *J* = 7.3 Hz, 1H, H3), 4.25 (d, *J* = 15.1 Hz, 1H, H8A), 4.04 (d, *J* = 15.0 Hz, 1H, H8B), 3.81 (s, 2H, H15), 3.69 (s, 1H, H6), 2.80 (m, 1H, H7A), 2.67 (m, 1H, H4), 2.63 (dd, *J* = 16.7, 9.0 Hz, 1H, H7B), 2.32 (t, *J* = 11.9 Hz, 1H, H1), 2.25 (m, 1H, H5A), 1.73 (dd, *J* = 14.8, 4.2 Hz, 1H, H5B), 1.28 (m, 1H, H2), 0.92 (d, *J* = 8.4 Hz, 9H, PMe3). **^{13}C NMR (201 MHz, CD_3CN, δ, 25 ºC):** 178.2 (1C, C20), 144.4 (1C, TpA3), 143.9 (1C, TpB3), 142.0 (1C, TpC5), 140.4 (1C, C16), 138.0 (1C, TpA5), 137.8 (1C, TpC3), 137.5 (1C, TpB5), 131.7 (1C, C10), 130.1 (1C, C12), 130.1 (1C, C18), 129.5 (1C, C17), 127.7 (1C, C19), 119.9 (1C, C13), 107.5 (1C, TpB4), 107.3 (1C, TpC4), 107.1 (1C, TpA4), 63.4 (1C, C3), 53.5 (1C, C1), 49.0 (1C, C2), 43.6 (1C, C6), 42.3 (1C, C8), 40.1 (1C, C7), 36.6 (1C, C15), 29.9 (1C, C5), 29.6 (1C, C4), 13.9 (3C, PMe3).

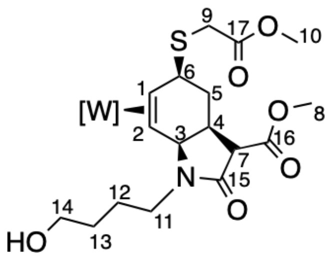

Compound 53:

Compound **34** (85 mg, 0.110 mmol) was placed in a test tube, with ACN (2 mL), and chilled to -30°C. 2-methoxy-2-oxoethyl-1-potassium thiolate (35 mg, 0.33 mmol) with ACN (2 mL) was cooled in a separate test tube, at -30 °C for 10 mins. After the time elapsed, the former solution was added to the latter, dropwise. The reaction stirred at -30 °C for 10 min. The reaction was washed three times (H_2O/DCM; 30 mL/30mL) and dried over anhydrous Na_2SO_4. The clear solution was evaporated in vacuo. The resulting yellow film was dissolved in minimal DCM and pipetted in 50 mL of stirring pentane. A white solid precipitated out and was collected on a 15 mL fine-porosity fritted disk, washed with pentane (2 × 10 mL) and desiccated overnight to yield compound **53** (61 mg, 0.070 mmol, 63 %).

^{1}H NMR (400 MHz, CD_3CN, δ, 25 ºC): 8.05 (t, *J* = 2.7 Hz, 2H, TpA3/TpB3), 7.85 (dd, *J* = 4.8, 2.3 Hz, 2H, TpB5/TpC5), 7.82 (d, *J* = 2.5 Hz, 1H, TpA5), 7.45 (d, *J* = 2.3 Hz, 1H, TpC3), 6.37 (t, *J* = 2.2 Hz, 1H, TpB4), 6.32 (t, *J* = 2.3 Hz, 1H, TpA4), 6.28 (t, *J* = 2.3 Hz, 1H, TpC4), 4.91 (d, *J* = 7.3 Hz, 1H, H3), 4.18 (s, 1H, H6), 4.02 (d, *J* = 6.0 Hz, 1H, H7), 3.72 (s, 3H, H10), 3.70 (s, 3H, H8),

3.45 (d, J = 14.8 Hz, 1H, H9A), 3.36 (d, J = 14.8 Hz, 1H, H9B), 3.30 (s, 3H, H11A/H14A/H14B), 2.84 (m, 1H, H4), 2.62 (d, J = 14.1 Hz, 1H, H11), 2.50 (t, J = 11.6 Hz, 1H, H1), 2.42 (m, 1H, H5A), 1.79 (d, J = 15.4 Hz, 1H, H5B), 1.26 (t, J = 8.3 Hz, 4H, H12A/H12B/H13A/H13B), 1.17 (d, J = 8.5 Hz, 9H, PMe3), 1.05 (d, J = 11.0 Hz, 1H, H2). **^{13}C NMR (201 MHz, CD_3CN, δ, 25 ºC):** 172.4 (1C, C15), 172.1 (1C, C16), 170.2 (1C, C17), 144.2 (1C, TpA3), 143.2 (1C, TpB3), 142.0 (1C, TpC3), 138.0 (1C, TpA5), 137.9 (1C, TpB5), 137.4 (1C, TpC5), 107.5 (1C, TpB4), 107.2 (1C, TpA4), 107.0 (1C, TpC4), 62.1 (1C, C14), 59.2 (1C, C3), 56.5 (1C, C7), 53.0 (1C, C1), 52.8 (1C, C9), 52.7 (1C, C10), 48.9 (1C, C2), 45.7 (1C, C6), 40.5 (1C, C11), 34.6 (1C, C5), 34.2 (1C, C10), 30.5 (1C, C12), 27.8 (1C, C5), 24.9 (1C, C13), 13.6 (3C, PMe3).

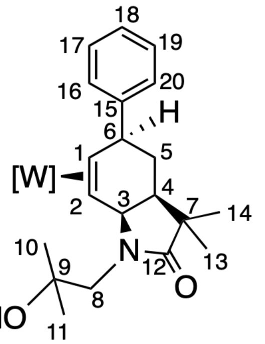

Compound 54:

Compound **35** (40 mg, 0.056 mmol) was placed in a test tube, with ACN (2 mL), and chilled to -30 °C. Phenyllithium (0.150 mL, 0.280 mmol, 2 M) was cooled in a separate test tube, at -30 °C for 30 min with CuI (32 mg, 0.170 mmol). After the time elapsed, the former solution was added to the latter, dropwise. The reaction stirred at -30 °C for 30 min. The reaction was washed three times (H_2O/DCM; 30 mL/30mL) and dried over anhydrous Na_2SO_4. The clear solution was evaporated in vacuo. The resulting clear film was dissolved in minimal DCM and pipetted in 100 mL of stirring pentane. A white solid precipitated out and was collected on a 15 mL fine-porosity fritted disk, washed with pentane (2 × 10 mL) and desiccated overnight to yield compound **54** (32.0 mg, 0.041, 72.0%).

^{1}H NMR (800 MHz, CD_2Cl_2, δ, 25 ºC): 8.03 (1H, d, TpB3), 7.99 (1H, TpA3), 7.77 (1H, d, TpB5), 7.75 (d, 1H, TpA5), 7.73 (1H, d, TpC5), 7.47 (2H, m, H16/H20), 7.47 (1H, d, TpC3), 7.38 (2H, m, H17/H19), 7.24 (1H, H18), 6.33 (t, 1H, TpB4), 6.27 (t, 1H, TpC4), 6.23 (t, 1H, TpA4), 4.95 (1H, d, H3), 3.90 (1H, m, H6), 3.23 (1H, d, H8), 2.93 (1H, dt, H1), 2.87 (1H, d, H8), 2.36 (1H, m, H4), 1.63 (1H, m, H5), 1.28 (5H, m, H2/H5/H10), 1.17 (3H, s, H11), 1.02 (3H, s, H13), 0.89 (3H, s, H14), 0.82 (9H, d, PMe_3). **^{13}C NMR (201 MHz, CD_2Cl_2, δ, 25 ºC):** 183.7 (1C, C12), 152.3 (1C, C15), 143.6 (1C, TpA3), 142.5 (1C, TpB3), 140.5 (1C, TpB5), 137.2 (1C, TpA5), 137.1 (1C, TpC5), 136.6 (1C, TpC3), 129.1 (2C, C16/C20), 128.4 (2C, C17/C19), 126.1 (1C, C18), 106.9 (1C, TpB4), 106.4 (1C, TpC4) , 106.1 (1C, TpA4), 71.7 (1C, C9), 60.7 (1C, C3), 55.4 (1C, C1), 53.7 (1C, C8), 50.3 (1C, C2), 45.5 (1C, C6), 44.5 (1C, C4), 44.4 (1C, C7), 36.8 (1C, C5), 28.5 (1C, C14), 28.3 (1C, C13), 23.8 (1C, C11), 19.7 (1C, C10), 14.3 (d, J = 29.7 Hz, 1C, PMe_3).

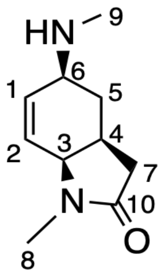

Compound 55:

Compound **36** (70.0 mg, 0.100 mmol) was placed in a test tube with no solvent at -40 °C for 10 min. In a separate test tube, $NOPF_6$ (25 mg, 0.14 mmol) with ACN (2 mL) was cooled to -40°C for 10 min. The latter solution was added to the former dropwise, then allowed to react for 5 minutes. The reaction was washed three times (H_2O:$NaHCO_3$/DCM; 30mL/30mL) and dried over anhydrous Na_2SO_4. The organic layer was evaporated in vacuo. The resulting yellow film was dissolved in minimal DCM and pipetted in stirring cold pentane (50 mL). A brown solid precipitated out and was collected on a 15 mL fine-porosity fitted disk and washed two times with cold pentane (2 x 30 ml). The resulting filtrate was evaporated to dryness to yield compound **55** as a colourless oil (13 mg, 0.072 mmol, 70%).

^{1}H NMR (800 MHz, CD_3CN, δ, 25 ºC): 6.21 (1H, m, H2), 6.07 (1H, m, H1), 5.30 (1H, m, H6), 3.92 (1H, bs, H3), 2.86 (3H, s, H10), 2.76 (3H, s, H8), 2.68 (1H, m, H4), 2.64 (1H, m, H7), 1.88 (2H, m, H5/H7), 1.56 (1H, q, H5). **^{13}C NMR (201 MHz, CD_3CN, δ, 25 ºC):** 174.2 (1C, C9), 133.4 (1C, C2), 128.8 (1C, C1), 60.4 (1C, C6), 56.1 (1C, C3), 38.8 (1C, C7), 32.0 (1C, C5), 30.7 (1C, C4), 29.5 (1C, C10), 27.7 (1C, C8). **HRMS (APCI$^+$, deconvoluted):** [M] calcd. for $C_{10}H_{16}N_2O$ 180.1263; found, 180.1262.

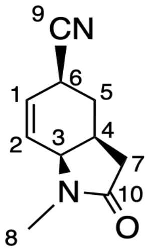

Compound 56:

Compound **39** (80 mg, 0.12 mmol) was placed in a test tube with acetone (2 mL). $NOPF_6$ was added (29 mg. 0.17 mmol) and the reaction stirred at room temperature for 10 min. The reaction was washed three times (H_2O:$NaHCO_3$/DCM; 30mL/30mL) and dried over anhydrous Na_2SO_4. The organic layer was evaporated in vacuo. The resulting yellow film was dissolved in minimal DCM and pipetted in 25 mL of stirring hexane. A brown solid precipitated out and was collected on a 15 mL fine-porosity fitted disk and washed two times with diethyl ether (5 ml). The resulting filtrate was evaporated to dryness to yield compound **56** as a colourless oil (13 mg, 0.074 mmol, 62%).

^{1}H-NMR (800 MHz, CD_3CN, δ, 25°C): 6.12 (1H, m, H2), 6.00 (1H, d, H1), 3.84 (1H, m, H3), 3.42 (1H, m, H6), 2.73 (1H, m, H8), 2.53 (1H, m, H7), 2.45 (1H, m, H4), 2.05 (2H, m, H5/H7), 1.57 (1H, m, H5). **^{13}C-NMR (201 MHz, CD_3CN, δ, 25°C):** 173.6 (1C, C10), 127.6 (1C, C2),122.0 (1C, C1), 122.0 (1C, C9), 55.6 (1C, C3), 37.5 (1C, C7), 30.3 (1C, C4), 29.5 (1C, C5), 27.4 (1C, C8), 26.7 (1C, C6). **HRMS ($APCI^+$, deconvoluted):** [M] calcd. for $C_{10}H_{12}N_2O$ 176.0950; Found $C_{10}H_{12}N_2NaO$ 199.0847.

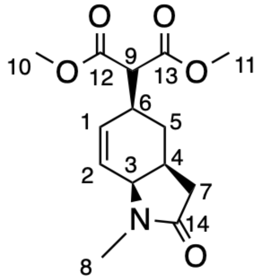

Compound 57:

Compound **40** (80 mg, 0.11 mmol) was placed in a test tube with acetone (2 mL). $NOPF_6$ was added (25 mg. 0.14 mmol) and the reaction stirred at room temperature for 10 min. The reaction was washed three times (H_2O:$NaHCO_3$/DCM; 30mL/30mL) and dried over anhydrous Na_2SO_4. The organic layer was evaporated in vacuo. The resulting yellow film was dissolved in minimal DCM and pipetted in 25 mL of stirring hexane. A brown solid precipitated out and was collected on a 15 mL fine-porosity fitted disk and washed two times with diethyl ether (5ml). The resulting filtrate was evaporated to dryness to yield compound **57** as a colourless oil (20 mg, 0.071 mmol, 69%).

^{1}H-NMR (800 MHz, CD_3CN, δ, 25°C): 5.96 (1H, m, H2), 5.90 (1H, m, H1), 3.82 (1H, m, H3), 3.68 (6H, s, H10/11), 3.33 (1H, d, H9) 2.83 (1H, m, H6), 2.69 (3H, s, H8), 2.56 (1H, m, H7), 2.43 (1H, m, H4), 1.89 (1H, d, H7), 1.63 (1H, m, H5), 1.10 (1H, m, H5). **^{13}C-NMR (201 MHz, CD_3CN, δ, 25°C):** 174.0 (1C, C14), 169.6 (1C, C12), 169.5 (1C, C13), 134.7 (1C, C1), 125.6 (1C, C2), 56.5 (1C, C9), 56.3 (1C, C3), 53.1 (2C, C11/10), 38.7 (1C, C7), 36.2 (1C, C6), 31.3 (1C, C4), 30.7 (1C, C5), 27.2 (1C, C8). **HRMS ($APCI^+$, deconvoluted):** [M] calcd. for $C_{14}H_{19}NO_5$ 281.1263; found, 281.1265.

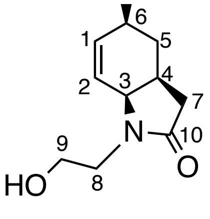

Compound 58:

Compound **42** (70 mg, 0.98 mmol) was placed in a test tube with acetone (2 mL). $NOPF_6$ was added (24.0 mg. 0.138 mmol) and the reaction stirred at room temperature for 10 min. The reaction was washed three times (H_2O:$NaHCO_3$/DCM; 30mL/30mL) and dried over anhydrous

Na_2SO_4. The organic layer was evaporated in vacuo. The resulting yellow film was dissolved in minimal DCM and pipetted in 25 mL of stirring hexane. A brown solid precipitated out and was collected on a 15 mL fine-porosity fitted disk and washed two times with diethyl ether (5 ml). The resulting filtrate was evaporated to dryness to yield compound **58** as a colourless oil (14 mg, 0.068 mmol, 69%).

^{1}H-NMR (800 MHz, $CDCl_3$, δ, 25°C): 6.14 (1H, m, H2), 6.04 (1H, d, H1), 4.10 (1H, m, H3), 3.78 (2H, m, H9), 3.68 (1H, m, H8), 3.32 (1H, m, H6), 3.27 (1H, m, H8), 2.72 (1H, m, H7), 2.49 (1H, m, H4), 2.27 (1H, d, H7), 2.09 (1H, m, H5), 1.79 (1H, m, H5). **^{13}C-NMR (201 MHz, $CDCl_3$, δ, 25°C):** 174.6 (1C, C10), 127.1 (1C, C2), 126.5 (1C, C1), 120.0 (1C, C11), 61.8 (1C, C9), 54.5 (1C, C3), 43.6 (1C, C8), 37.0 (1C, C7), 30.4 (1C, C4), 28.4 (1C, C5), 26.4 (1C, C6). **HRMS ($APCI^+$, deconvoluted):** [M] calcd. for $C_{11}H_{14}N_2O_2$ 206.1055; found 206.1057.

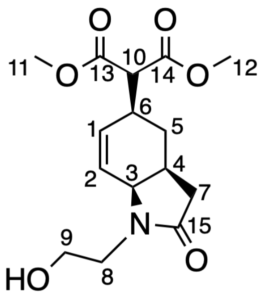

Compound 59:

Compound **43** (100 mg, 0.123 mmol) was placed in a test tube with acetone (2 mL). $NOPF_6$ was added (30.0 mg. 0.172 mmol) and the reaction stirred at room temperature for 10 min. The reaction was washed three times (H_2O:$NaHCO_3$/DCM; 30mL/30mL) and dried over anhydrous Na_2SO_4. The organic layer was evaporated in vacuo. The resulting yellow film was dissolved in minimal DCM and pipetted in 25 mL of stirring hexane. A brown solid precipitated out and was collected on a 15 mL fine-porosity fitted disk and washed two times with diethyl ether (5ml). The resulting filtrate was evaporated to dryness to yield compound 59 as a colourless oil (25 mg, 0.080 mmol, 65%).

^{1}H-NMR (800 MHz, CD_3CN, δ, 25°C): 5.98 (1H, m, H1), 5.90 (1H, m, H2), 4.02 (1H, m, H3), 3.68 (6H, s, H14/15), 3.56 (1H, m, H9), 3.51 (1H, m, H9), 3.45 (1H, m, H8), 3.35 (1H, d, H10), 3.14 (1H, m, H8), 2.84, 1H, m, H6), 2.62 (1H, m, H7), 2.43 (1H, m, H4), 1.89, (1H, d, H7), 1.62 (1H, m, H5), 1.11 (1H, m, H5). **^{13}C-NMR (201 MHz, CD_3CN, δ, 25°C):** 175.0 (1C, C13), 169.6 (1C, C11), 169.5 (1C, C12), 134.8 (1C, C1), 125.5 (1C, C2), 61.1 (1C, C9), 56.5 (1C, C10), 55.5 (1C, C3), 53.0 (2C, C14/15), 43.6 (1C, C8), 38.7 (1C, C7), 36.1 (1C, C6), 31.8 (1C, C4), 30.3 (1C, C5). **HRMS ($APCI^+$, deconvoluted):** [M] calcd. for $C_{15}H_{21}NO_6$ 311.1369; found 311.1371.

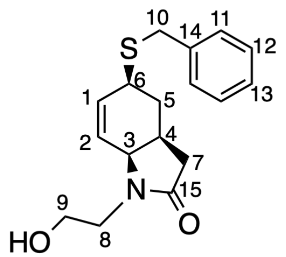

Compound 60:

Compound **44** (80.0 mg, 0.099 mmol) with ACN (2 mL) was placed in a test tube. The solution was stirred at room temperature for 10 min. In a separate test tube, 2,3-Dichloro-5,6-dicyano-1,4-benzoquinone (31.5 mg, 0.139 mmol) with ACN (2 mL) was cooled to -30°C for 10 min. The former solution was added to the later dropwise, then allowed to warm to room temperature over 10 minutes. The reaction was washed three times (H_2O:$NaHCO_3$/DCM; 30mL/30mL) and dried over anhydrous Na_2SO_4. The organic layer was evaporated in vacuo. The resulting yellow film was dissolved in minimal DCM and pipetted in 25 mL of stirring hexane. A brown solid precipitated out and was collected on a 15 mL fine-porosity fitted disk and washed two times with diethyl ether (5 ml). The resulting filtrate was evaporated to dryness to yield compound **60** as a colourless oil (24 mg, 0.079 mmol, 80%).

^{1}H NMR (800 MHz, $CDCl_3$, δ, 25°C): 7.33-7.30 (5H, phenyl ring), 6.05 (1H, m, H1), 5.88 (1H, m, H2), 4.01 (1H, m, H3), 3.77 (3H, s, H11), 3.76 (2H, m, H9), 3.59 (1H, m, H8), 3.31 (1H, m, H8), 3.21 (1H, m, H6), 2.67 (1H, dd, H7), 2.42 (1H, m, H4), 2.24 (1H, d, H7), 1.95 (1H, m, H5), 1.54 (1H, m, H5).**^{13}C NMR (800 MHz, $CDCl_3$, δ, 25°C):** 175.7 (1C, C10), 138.1 (1C, C1), 135.5 (1C, C2), 129.0, 128.7, 127.3, 123.7 (6C, phenyl ring), 61.9 (1C, C9), 55.3 (1C, C3), 44.0 (1C, C8), 38.9 (1C, C6), 37.8 (1C, C7), 34.8 (1C, C11), 32.6 (1C, C5), 31.6 (1C, C4). **HRMS (APCI$^+$, deconvoluted):** [M] calcd. for $C_{17}H_{21}NO_2S$ 303.1293; found, 303.1298.

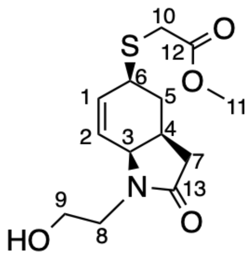

Compound 61:

Compound **45** (100.0 mg, 0.127 mmol) with ACN (2 mL) was placed in a test tube. The solution was stirred at room temperature for 10 min. In a separate test tube, 2,3-Dichloro-5,6-dicyano-1,4-benzoquinone (57.6 mg, 0.254 mmol) with ACN (2 mL) was cooled to -30°C for 10 min. The former solution was added to the later dropwise, then allowed to warm to room temperature over 10 minutes. The reaction was washed three times (H_2O:$NaHCO_3$/DCM; 30mL/30mL) and dried over anhydrous Na_2SO_4. The organic layer was evaporated in vacuo. The resulting yellow film was dissolved in minimal DCM and pipetted in 25 mL of stirring hexane. A brown solid precipitated out and was collected on a 15 mL fine-porosity fitted disk and washed two times with diethyl ether (5

ml). The resulting filtrate was evaporated to dryness to yield compound **61** as a colourless oil (24 mg, 0.077 mmol, 61%).

^{1}H NMR (800 MHz, CD_3CN, δ, 25°C): 6.03 (2H, s, H1/H2), 4.02 (1H, d, H3), 3.66 (3H, s, H11), 3.58 (2H, m, H9), 3.48 (2H, m, H8/H6), 3.30 (2H, s, H10), 3.15 (1H, m, H8), 2.57 (1H, m, H7), 2.45 (1H, m, H4), 2.02 (1H, d, H7), 1.97 (1H, m, H5), 1.32 (1H, q, H5).**^{13}C NMR (800 MHz, CD_3CN, δ, 25°C):** 174. 9 (1C, C13), 172.0 (1C, C12), 134.8 (1C, C2), 126.2 (1C, C1), 61.1 (1C, C9), 55.3 (1C, C3), 52.9 (1C C11), 43.8 (1C, C8), 40.8 (1C, C6), 38.3 (1C, C7), 33.0 (1C, C10), 32.4 (1C, C5), 32.0 (1C, C4). **HRMS (APCI$^+$, deconvoluted):** [M] calcd. for $C_{13}H_{19}NO_4S$ 285.1035; Found, 285.1038.

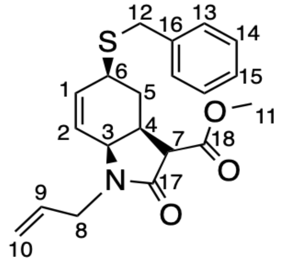

Compound 62:

Compound **47** (92 mg, 0.260 mmol) with ACN (2 mL) was placed in a test tube. The solution was stirred at room temperature for 10 min. In a separate test tube, 2,3-Dichloro-5,6-dicyano-1,4-benzoquinone (120 mg, 0.510 mmol) with ACN (2 mL) was cooled to -30°C for 10 min. The former solution was added to the later dropwise, then allowed to warm to room temperature over 10 minutes. The reaction was washed three times (H_2O:$NaHCO_3$/DCM; 30mL/30mL) and dried over anhydrous Na_2SO_4. The organic layer was evaporated in vacuo. The resulting yellow film was dissolved in minimal DCM and pipetted in 25 mL of stirring hexane. A brown solid precipitated out and was collected on a 15 mL fine-porosity fitted disk and washed two times with diethyl ether (5 ml). The resulting filtrate was evaporated to dryness to yield compound 62 as a colourless oil (49 mg, 0.14 mmol, 53%).

^{1}H NMR (800 MHz, CD_3CN, δ, 25°C): 7.37 – 7.23 (m, 5H, H13/H14/H15), 5.98 (m, 1H, H2), 5.85 (dt, J = 10.2, 3.1 Hz, 1H, H1), 5.74 (ddt, J = 16.7, 11.0, 5.7 Hz, 1H, H9), 5.20 (dt, J = 17.4, 1.6 Hz, 1H, H10A), 5.16 (dt, J = 10.4, 1.5 Hz, 1H, H10B), 4.14 (dt, J = 10.3, 4.8 Hz, 2H, H8A/H3), 3.81 (d, J = 1.5 Hz, 2H, H12), 3.70 (d, J = 1.2 Hz, 1H, H11), 3.61 (dd, J = 16.0, 6.4 Hz, 1H, H8B), 3.33 (s, 1H, H6), 2.74 (dq, J = 10.5, 4.6 Hz, 1H, H4), 2.27 (m, 1H, H7) 2.06 (dt, J = 13.5, 4.7 Hz, 1H, H5A), 1.53 (dt, J = 13.8, 9.2 Hz, 1H, H5B). **^{13}C NMR (201 MHz, CD_3CN, δ, 25°C):** 171.2, (1C, C17), 169.3 (1C, C18), 134.2 (1C, C2), 133.7 (1C, C9), 130.4 (1C, C16), 129.92 - 129.50 (3C, C13/C14/C15), 124.9 (1C, C1), 117.7 (1C, C10), 53.9 (1C, C3), 53.0 (1C, C11), 43.6 (2C, C7/C8), 39.3 (1C, C6), 36.3 (1C, C4), 35.5 (1C, C12), 31.3 (1C, C5). **HRMS (APCI$^+$, deconvoluted):** [M] calcd. for $C_{20}H_{23}NO_3S$ 357.1399; Found, 357.1399.

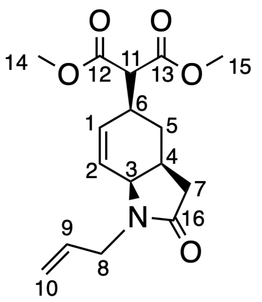

Compound 63:
Compound **48** (118.0 mg, 0.146 mmol) was placed in a test tube with acetone (2 mL). $NOPF_6$ was added (35.7 mg. 0.204 mmol) and the reaction stirred at room temperature for 10 min. The reaction was washed three times (H_2O:$NaHCO_3$/DCM; 30mL/30mL) and dried over anhydrous Na_2SO_4. The organic layer was evaporated in vacuo. The resulting yellow film was dissolved in minimal DCM and pipetted in 25 mL of stirring hexane. A brown solid precipitated out and was collected on a 15 mL fine-porosity fitted disk and washed two times with diethyl ether (5ml). The resulting filtrate was evaporated to dryness to yield compound **63** as a colourless oil (32 mg, 0.10 mmol, 71%).

^{1}H-NMR (800 MHz, CD_3CN, δ, 25°C): 5.89 (2H, m, H1/H2), 5.73 (1H, m, H9), 5.16 (1H, m, H10), 5.13 (1H, m, H10), 4.06 (1H, m, H8), 3.93 (1H, m, H3), 3.69 (6H, s, H15/H14), 3.57 (1H, m, H8), 3.35 (1H, s, H11), 2.83 (1H, m, H6), 2.60 (1H, dd, H7), 2.43 (1H, m, H4), 1.91 (1H, m, H7), 1.65 (1H, m, H5), 1.13 (1H, m, H5). **^{13}C-NMR (201 MHz, CD_3CN, δ, 25°C):** 173.9 (1C, C16), 169.5 (2C, C13/C12), 134.5 (2C, C9/C1), 125.5 (1C, C2), 117.0 (1C, C10), 56.3 (1C, C11), 54.7 (1C, C3), 53.1(2C, C15/C14), 43.2 (1C, C8), 38.6 (1C, C7), 36.2 (1C, C6), 31.5 (1C, C4), 30.5 (1C, C5). **HRMS (APCI$^+$, deconvoluted):** [M] calcd. for $C_{16}H_{21}NO_5$ 307.1420; found $C_{16}H_{22}NO_5$ 308.1495.

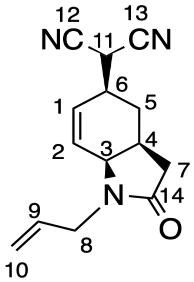

Compound 64:
Compound **49** (93 mg, 0.12 mmol) was placed in a test tube with acetone (2 mL). $NOPF_6$ was added (26 mg. 0.15 mmol) and the reaction stirred at room temperature for 10 min. The reaction was washed three times (H_2O:$NaHCO_3$/DCM; 30mL/30mL) and dried over anhydrous Na_2SO_4. The organic layer was evaporated in vacuo. The resulting yellow film was dissolved in minimal DCM and pipetted in 25 mL of stirring hexane. A brown solid precipitated out and was collected on a 15 mL fine-porosity fitted disk and washed two times with diethyl ether (5ml). The resulting filtrate was evaporated to dryness to yield compound **64** as a colourless oil (22 mg, 0.12 mmol, 73%).

^{1}H NMR (800 MHz, CD_3CN, δ, 25°C): 6.14 (dt, J = 10.1, 3.3 Hz, 1H, H2), 5.94 (dq, J = 10.1, 1.5 Hz, 1H, H1), 5.74 (m, 1H, H9), 5.19 (dt, J = 17.3, 1.5 Hz, 1H, H10A), 5.13 (dt, J = 10.3, 1.4 Hz, 1H, H10B), 4.24 (dd, J = 5.2, 0.9 Hz, 1H, H11), 4.07 (ddt, J = 16.1, 4.5, 2.2 Hz, 1H, H8A), 3.98 (t, J = 5.1 Hz, 1H, H3), 3.64 (m, 1H, H8B), 2.91 (m, 1H, H6), 2.65 (dd, J = 16.8, 8.2 Hz, 1H, H7A), 2.51 (m, 1H, H4), 2.03 (d, J = 16.8 Hz, 1H, H7B), 1.92 (d, J = 1.4 Hz, 1H, H5A), 1.27 (s, 1H, H5B). **^{13}C NMR (201 MHz, CD_3CN, δ, 25°C):** 173.8 (1C, C140, 134.3 (1C, C9), 130.7 (1C, C1), 129.3 (1C, C2), 117.5 (1C, C10), 113.5 (1C, C12), 113.5 (1C, C13), 54.5 (1C, C3), 43.4 (1C, C8), 38.3 (1C, C7), 37.4 (1C, C6), 30.7 (1C, C4), 30.0 (1C, C5), 28.9 (1C, C11). **HRMS (APCI$^+$, deconvoluted):** [M] calcd. for $C_{14}H_{15}N_3O$ 241.1215; Found, 241.1216.

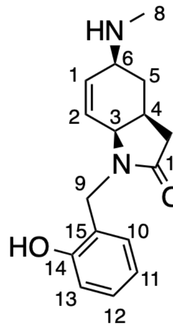

Compound 65:

Compound **51** (81 mg, 0.10 mmol) was placed in a test tube with no solvent at -40 °C for 10 min. In a separate test tube, $NOPF_6$ (26 mg, 0.15 mmol) with ACN (2 mL) was cooled to -40°C for 10 min. The latter solution was added to the former dropwise, then allowed to react for 5 minutes. The reaction was washed three times (H_2O:NH4Cl/DCM; 30mL/30mL) and the DCM layers discarded. The aqueous layer was then washed three times (H_2O:$NaHCO_3$/DCM; 30mL/30mL). The DCM layer was evaporated in vacuo to yield compound **65** as a colourless oil (19 mg, 0.070 mmol, 67%).

^{1}H NMR (800 MHz, CD_3CN, δ, 25°C): 7.21 (dd, J = 7.5, 1.7 Hz, 1H, H10), 7.17 (ddd, J = 8.1, 7.3, 1.7 Hz, 1H, H12), 6.83 (dd, J = 8.1, 1.2 Hz, 1H, H13), 6.82 (dd, J = 7.4, 1.2 Hz, 1H, H11), 6.35 (m, 1H, H2), 6.19 (dd, J = 10.2, 1.7 Hz, 1H, H1), 4.48 (d, J = 15.3 Hz, 1H, H9A), 4.24 (d, J = 15.3 Hz, 1H, H9B), 3.97 (s, 1H, H3), 3.78 (m, 1H, H6), 2.71 (dd, J = 17.3, 8.2 Hz, 1H, H7A), 2.55 (m, 1H, H4), 2.50 (s, 3H, MeNH2), 2.15 (2H, H7B & H5B), 1.43 (m, 1H, H5A). **^{13}C NMR (201 MHz, CD_3CN, δ, 25°C):** 167.54 (1C, C16), 131.93 (1C, C10), 130.58 (1C, C12), 129.65 (1C, C1), 128.99 (1C, C2), 127.30 (2C, C14/C15), 120.55 (1C, C11), 55.18 (1C, C3), 54.97 (1C, C6), 41.43 (1C, C9), 37.92 (1C, C7), 30.19 (1C, C8), 29.74 (1C, C4), 28.04 (1C, C5). **HRMS (APCI$^+$, deconvoluted):** [M] calcd. for $C_{16}H_{20}N_2O_2$ = 273.1598; Found, 273.1599.

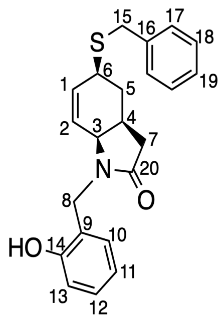

Compound 66:

Compound **52** (100 mg, 0.274 mmol) with ACN (2 mL) was placed in a test tube. The solution was stirred at room temperature for 10 min. In a separate test tube, 2,3-Dichloro-5,6-dicyano-1,4-benzoquinone (124 mg, 0.547 mmol) with ACN (2 mL) was cooled to -30°C for 10 min. The former solution was added to the later dropwise, then allowed to warm to room temperature over 10 minutes. The reaction was washed three times (H_2O:$NaHCO_3$/DCM; 30mL/30mL) and dried over anhydrous Na_2SO_4. The organic layer was evaporated in vacuo. The resulting yellow film was dissolved in minimal DCM and pipetted in 25 mL of stirring hexane. A brown solid precipitated out and was collected on a 15 mL fine-porosity fitted disk and washed two times with diethyl ether (5 ml). The resulting filtrate was evaporated to dryness to yield compound 66 as a colourless oil (63 mg, 0.17 mmol, 63%).

^{1}H NMR (800 MHz, CD_3CN, δ, 25°C): 7.27 (1H, dd, H17), 7.23 (1H, dd, H19/H10), 7.12 (1H, ddd, H12), 6.82 (2H, dd, H13/H11), 6.13 (m, 1H, H2), 6.03 (dd, 1H, H1), 4.44 (d, 1H, H8), 4.24 (d, 1H, H8), 3.95 (s, 1H, H3), 3.68 (s, 2H, H15), 3.27 (dd, 1H, 6), 2.61 (dd, 1H, H7), 2.42 (m, 1H, H4), 2.08 (1H, m, H7), 1.90 (m, 1H, H5) 1.32 (1H, m, H5).**^{13}C NMR (201 MHz, CD_3CN, δ, 25°C):** 176.9 (1C, C20), 156,8 (1C, C14), 139.8 (1C, C16), 136.2 (1C, C1), 132.2 (1C, C10), 130.7 (1C, C12), 129.9 (1C, C17), 129.5 (1C, C18), 129.5 (1C, C18), 127.9 (1C, C19), 125.0 (1C, C2), 124.0 (1C, C9), 120.6 (1C, C11), 118.0 (1C, C13), 55.9 (1C, C3), 41.7 (1C, C8), 40.0 (1C, C6), 38.1 (1C, C7), 34.5 (1C, C15), 32.9 (1C, C5), 31.9 (1C, C4). **HRMS (APCI$^+$, deconvoluted):** [M] calcd. for $C_{22}H_{23}NO_2S$ 365.1450; Found, 365.1453.

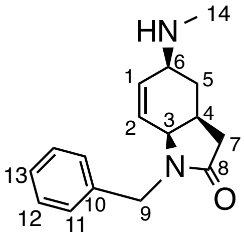

Compound 69:

Compound **29** (200 mg, 0.228 mmol) was taken in THF (3 mL) and cooled to -60°C. 2M $MeNH_2$ in THF (1.14 mL, 2.28 mmol) was added to this and stirred for 15 min. Meanwhile, KOtBu (256 mg, 2.28 mmol) in THF (2 mL) was cooled to -60°C and then added to the initial reaction. The combined reaction was then precipitated into 50 mL pentane; the precipitate collected on a fritted disk and washed 2x2 mL H_2O to yield compound **68** as a white powder**.** Compound **68** was dried under high vacuum, before being redissolved in ACN (3 mL) and cooled to -40°C. $NOPF_6$ (40 mg, 0.228 mmol) in ACN (1 mL), cooled to -40°C, was then added and the reaction was allowed to

warm to room temperature for 10 min. The crude mixture was taken in a sep. funnel with 0.1M HCl and extracted 4x10 mL with DCM. The aqueous layer was then basified with 1M NaOH (pH ~10 by litmus paper) and extracted 4x10 mL DCM. These DCM layers were combined, dried over Na_2SO_4, and evaporated to dryness in a weighed vile to yield compound **69** as a colourless oil. (11mg, 0.043 mmol, 19% from 29).

^{1}H NMR (600 MHz, CD_3CN) δ 7.28 (m, 5H), 5.92 (dq, *J* = 10.1, 1.5 Hz, 1H), 5.75 (ddd, *J* = 10.2, 3.9, 2.2 Hz, 1H), 4.70 (d, *J* = 15.4 Hz, 1H), 4.14 (d, *J* = 15.4 Hz, 1H), 3.79 (tdd, *J* = 6.7, 4.3, 2.3 Hz, 1H), 3.02 (ddt, *J* = 10.5, 4.1, 2.0 Hz, 1H), 2.65 (dd, *J* = 16.7, 8.3 Hz, 1H), 2.41 (m, 1H), 2.33 (s, 3H), 2.06 (m, 2H), 1.86 (dtd, *J* = 12.4, 4.3, 1.3 Hz, 1H), 1.12 (m, 1H). **^{13}C NMR (201 MHz, CD_3CN) δ** 174.6, 138.7, 137.5, 129.5, 128.6, 128.1, 124.0, 55.6, 55.2, 44.5, 38.7, 33.4, 33.2, 30.9. **HRMS (APCI$^+$, deconvoluted):** [M] calcd. for $C_{16}H_{20}N_2O$, 256.1576; found, 256.1578.

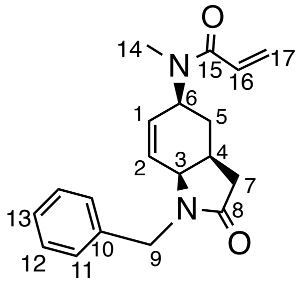

Compound 70:

Compound **69** (11 mg, 0.043 mmol) was taken in DCM (4 mL) in a vial with 5 mL of sat. Na_2CO_3 (aq). Acryloyl chloride (5.8 mg, 0.064 mmol) in DCM (1 mL) was added to this mixture and stirred vigorously for 10 min. The crude reaction was then transferred to a sep. funnel and the aq. layer extracted 3x5 mL DCM. The combined organic layers were dried over Na_2SO_4 and evaporated to dryness. The crude mixture was purified by CombiFlash (4 g silica column) with a hexane to EtOAc to MeOH gradient. The product was observed as a uv absorbance peak, eluting in pure MeOH. This fraction was evaporated to dryness, redissolved in ACN, filtered through 0.45 µm syringe filter, and collected in a weighed vial to yield compound **70** as a colourless oil (7mg, 0.023 mmol, 53%) as a mixture of rotamers which are distinct by NMR at room temperature. Peaks are labeled Ma and mi for major and minor if distinct, otherwise reported as one peak.

^{1}H NMR (600 MHz, CD_3CN) δ 7.33 (2H, m, H12), 7.26 (3H,m, H11/H13), 6.65 (dd, *J* = 16.7, 10.5 Hz, H16), 6.17 (1H, dd, *J* = 16.8, 2.3 Hz, H17Ma), 6.09 (1H dd, *J* = 16.8, 2.3 Hz, H17mi), 5.91 (1H, ddt, *J* = 8.5, 4.3, 2.1 Hz, H2), 5.79 (1H, d, *J* = 10.3, H1mi), 5.70 (1H, dq, *J* = 10.2, 1.6 Hz, H1Ma), 5.65 (1H, dd, *J* = 10.5, 2.3 Hz, H17Ma), 5.59 (1H, dd, *J* = 10.6, 2.3 Hz, H17mi), 5.14 (1H, ddd, *J* = 11.2, 4.9, 2.4 Hz, H6Ma), 4.58 (1H, d, *J* = 15.4 Hz, H9Ma), 4.57 (1H, d, *J* = 15.5 Hz, H9mi), 4.52 (1H, m, H6mi), 4.34 (1H, d, *J* = 15.4 Hz, H9mi), 4.32 (1H, d, *J* = 15.4 Hz, H9Ma), 3.86 (1H, ddd, *J* = 5.8, 3.9, 1.8 Hz, H3), 2.72 (3H, s, H14Ma), 2.68 (1H, dd, *J* = 16.7, 8.1 Hz, H7), 2.61 (3H, s, H14mi), 2.54 (1H, m, H4), 2.02 (1H, d, *J* = 16.7 Hz, H7), 1.68 (1H, dt, *J* = 10.7, 5.0 Hz, H5mi), 1.61 (1H, m, H5Ma), 1.55 (1H, m, H5mi), 1.42 (1H, m, H5Ma). **^{13}C NMR (151 MHz, CD_3CN) δ** 174.5 (1C, C8Ma), 174.4 (1C, C8mi), 167.3 (1C, C15mi), 166.8 (1C, C15Ma), 139.0 (1C, C10Ma), 138.9 (1C, C10mi), 135.5 (1C, C1Ma), 135.1 (1C, C1mi), 129.8 (1C, C16Ma), 129.8 (1C, C16mi), 129.5 (2C, C12), 128.6 (2C, C11), 128.1 (1C, C13), 127.7 (1C, C17Ma), 127.5 (1C,

C2Ma), 127.4 (1C, C2mi), 127.0 (1C, C17mi), 54.6 (1C, C3Ma), 54.4 (1C, C3mi), 54.3 (1C, C6mi), 50.6 (1C, C6Ma), 44.7 (1C, C9mi), 44.6 (1C, C9Ma), 38.7 (1C, C7Ma), 38.5 (1C, C7mi), 31.1 (1C, C5mi), 31.0 (1C, C4Ma), 30.8 (1C, C4mi), 30.7 (1C, C14Ma), 29.4 (1C, C5Ma), 28.9 (1C, C14mi). **HRMS (APCI$^+$, deconvoluted):** [M] calcd. for $C_{19}H_{22}N_2O_2$, 310.1681; found, 310.1670.

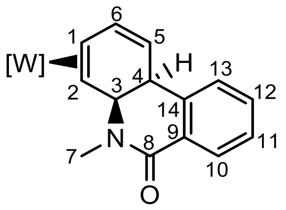

Compound 72:

Compound **71** (150 mg, 0.189 mmol) was taken into a test tube along with CAN (5mL). The solution was chilled to -30°C for 5 minutes. 1 M HOTf (377 µl, 0.377 mmol) was added to the solution. After 15 minutes, a solution of 2 M methylamine in THF (943 µl, 1.89 mmol) was added. This mixture was stirred at -30°C for 2 days, and then 6 h at room temperature. Then, 5 mL of saturated Na2CO3 solution was added followed by 5 mL of dichloromethane. The DCM layer was extracted and dried using Na_2SO_4 and the extraction\drying process was repeated twice more. The extracted organic layers were combined and evaporated until a film was formed. The film was then redissolved in the minimum amount of DCM possible and precipitated into 60 mL of stirring hexanes. The precipitate was then collected on a 15 mL fine-porosity fritted disc and desiccated overnight yielding compound **72** (130 mg, 0.182 mmol, 96%).

^{1}H NMR (800 MHz, $CDCl_3$) δ 8.13 (1H, d, *J* = 2.1 Hz, Tp3B), 8.08 (1H, dd, *J* = 7.8, 1.4 Hz, H10), 7.83 (1H, d, *J* = 2.1 Hz, Tp3A), 7.77 (1H, d, *J* = 2.4 Hz, Tp5C), 7.74 (1H, d, *J* = 2.4 Hz, Tp5B), 7.61 (1H, d, *J* = 2.4 Hz, Tp5A), 7.44 (1H, td, *J* = 7.4, 1.4 Hz, H12), 7.39 (2H, m, Tp3C/H13), 7.33 (1H, td, *J* = 7.5, 1.4 Hz, H11), 6.45 (1H, ddd, *J* = 9.0, 4.9, 2.9 Hz, H6), 6.34 (1H, t, *J* = 2.2 Hz, Tp4B), 6.29 (1H, t, *J* = 2.2 Hz, Tp4C), 6.15 (1H, t, *J* = 2.3 Hz, Tp4A), 4.74 (1H, dt, *J* = 9.4, 1.6 Hz, H5), 4.64 (1H, m, H3), 4.28 (1H, d, *J* = 4.5 Hz, H4), 3.22 (3H, s, H7), 3.06 (1H, ddd, *J* = 14.5, 9.7, 5.3 Hz, H1), 1.60 (1H, dd, *J* = 9.7, 2.9 Hz, H2), 1.30 (9H, d, *J* = 8.5 Hz, PMe_3). **^{13}C NMR (201 MHz, $CDCl_3$) δ** 166.3 (1C, C8), 143.6 (1C, Tp3B), 143.0 (1C, Tp3A), 142.9 (1C,C14), 140.1 (1C, Tp3C), 136.9 (1C,Tp5C), 136.2 (1C,Tp5B), 135.7 (1C,Tp5A), 131.7 (1C, H12), 130.6 (1C, C6), 129.1 (1C, C9), 128.1 (1C, C10), 127.8 (1C, C13), 126.7 (1C, C11), 119.9 (1C, C5), 106.7 (1C, Tp4B), 106.5 (1C, Tp4C), 106.0 (1C, Tp4A), 59.4 (1C, C3), 53.0 (1C, C2), 50.4 (1C, d, *J* = 10.7 Hz, C1), 38.9 (1C, C4), 29.9 (1C, C7), 13.8 (3C, d, *J* = 28.3 Hz, PMe_3).

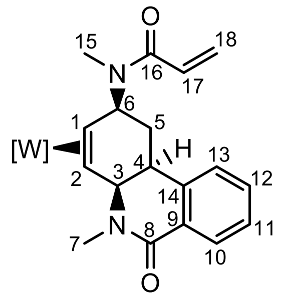

Compound 74:

Compound **72** (94 mg, 0.132 mmol) was taken in ACN (1 mL) and cooled to -30°C. To this 1M HOTf in ACN (290 µL) was added and stirred 30 min. The reaction was then precipitated into chilled (-10°C) Et_2O, collected on a fritted disc, and the resulting white powder was washed 2x3 mL Et_2O to yield compound **73**. This was then redissolved, without further purification, in ACN (1 mL) and cooled -30°C. 2M $MeNH_2$ in THF (194 µL, 0.389 mmol) was added and the reaction stirred for 20 min. Meanwhile, Na_2CO_3 (103 mg, 0.972 mmol) was dissolved in H_2O (1.5 mL) and cooled in an ice bath. The initial reaction was then added directly to the carbonate solution and followed quickly by the addition of acryloyl chloride (44 mg, 0.486 mmol). The mixture was stirred vigorously and allowed to warm to room temperature over 30 min. After this time the reaction was diluted with Na_2CO_3 (sat. aq.) and extracted 3x10 mL DCM. The combined organic layers were dried over Na_2SO_4, evaporated to dryness, redissolved in minimal DCM and precipitated into 50 mL hexanes. The precipitate was then collected as an off-white powder over a fritted disc to yield compound **74** (66mg, 0.083 mmol, 63%). Compound 74 was confirmed by H-NMR and SC-XRD. As compound 74 exists as rotamers at room temperature, it was taken on without any further purification.

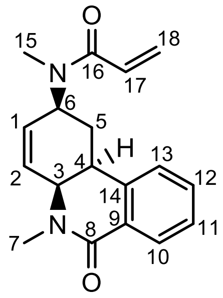

Compound 75:

Compound **74** (66 mg, 0.083 mmol) was taken in ACN (2 mL) and $AgNO_3$ (56 mg, 0.330 mmol) added. The reaction was then heated at 60°C for 20 min. The crude reaction mixture was then evaporated to an oil and purified by CombiFlash (4 g silica column) with a hexane to EtOAc to gradient. The product was observed as a uv absorbance peak, eluting in 80% EtOAc/Hex. This fraction was evaporated to dryness, redissolved in ACN, filtered through 0.45 µm syringe filter, and collected in a weighed vial to yield compound **75** as a colourless oil. (10mg, 0.034 mmol, 41%, 26% from Compound 73) %) as a mixture of rotamers which are distinct by NMR at room temperature. Peaks are labeled Ma and mi for major and minor if distinct, otherwise reported as one peak.

^{1}H NMR (800 MHz, CD_3CN) δ 7.97 (1H, d, *J* = 7.3 Hz, H10), 7.49 (1H, dt, *J* = 14.5, 7.5 Hz, H12), 7.36 (1H, q, *J* = 7.7 Hz, H11), 7.32 (1H, t, *J* = 7.1 Hz, H13), 6.79 (1H, dd, *J* = 16.8, 10.6 Hz, H17mi), 6.64 (1H, dd, *J* = 16.7, 10.4 Hz, H17Ma), 6.24 (1H, ddd, *J* = 9.3, 5.8, 2.7 Hz, H2), 6.18 (1H, dd, *J* = 16.8, 2.4 Hz, H18Ma), 6.13 (1H, d, *J* = 16.8, H18mi), 5.99 (1H, d, *J* = 10.2 Hz, H1mi), 5.90 (1H, d, *J* = 10.2 Hz, H1Ma), 5.65 (1H, dd, *J* = 10.6, 2.3 Hz, H18), 5.43 (1H, dd, *J* = 10.5, 6.5 Hz, H6Ma), 4.81 (1H, t, *J* = 8.5 Hz, H6mi), 4.24 (1H, t, *J* = 4.9 Hz, H4), 3.18 (1H, m, H4), 3.08 (3H, s, H7), 2.81 (3H, s, H15Ma), 2.70 (3H, s, H15mi), 2.00 (1H, q, *J* = 12.5 Hz, H5mi), 1.88 (1H, q, *J* = 12.5 Hz, H5Ma), 1.73 (1H, m, H5mi), 1.67 (1H, m, H5Ma). **^{13}C NMR (201 MHz, CD_3CN) δ** 167.3 (1C, C16mi), 166.8 (1C, C16Ma), 164.7 (1C, C8), 142.7 (1C, C17Ma), 142.5 (1C, C14mi), 134.8 (1C, C1Ma), 134.6 (1C, C1mi), 133.1 (1C, C12), 129.8 (1C, C17mi), 129.7 (1C, C17Ma), 129.5 (1C, C2Ma), 129.4 (1C, C2mi), 128.9 (1C, C9), 128.8 (1C, C10mi), 128.8 (1C, C10Ma), 128.2 (1C, C13Ma), 128.2 (1C, C13mi), 128.1 (1C, C11mi), 128.1 (1C, C11Ma), 127.8 (1C, C18Ma), 127.1 (1C, C18mi), 55.5 (1C, C6mi), 52.4 (1C, C3Ma), 52.2 (1C, C3mi), 51.9 (1C, C6Ma), 38.0 (1C, C4Ma), 37.9 (1C, C4mi), 31.1 (1C, C15Ma), 30.8 (1C, C5mi), 30.2 (1C, C7), 29.2 (1C, C5Ma/C15mi). **HRMS ($APCI^+$, deconvoluted):** [M] calcd. for $C_{18}H_{20}N_2O_2$, 296.1525; found, 296.1529.

DFT Analysis

Ground-state structures were optimized at the M06 level of theory using the 6-31G** [LANL2DZ for W] basis set in Gaussian 16. Previous literature demonstrates that this functional and basis set choice accurately corroborates experimental results.[3] Solvent effects of acetonitrile were modeled using SMD. Gaussian's default criteria were used for optimization, vibrational frequency analysis verified that structures were minima and thermal free energy corrections were applied.

Figure S123: Summary of DFT calculations.

Structure	Electronic Energy (Hartree)	Relative Free Energy (kcal/mol)
9D	-1839.489420	0.1
9P	-1839.489653	0.0
27-D	-1839.943947	0.0
27-P	-1839.943133	0.5
39D	-1932.879764	0.0
39P	-1932.879312	0.3

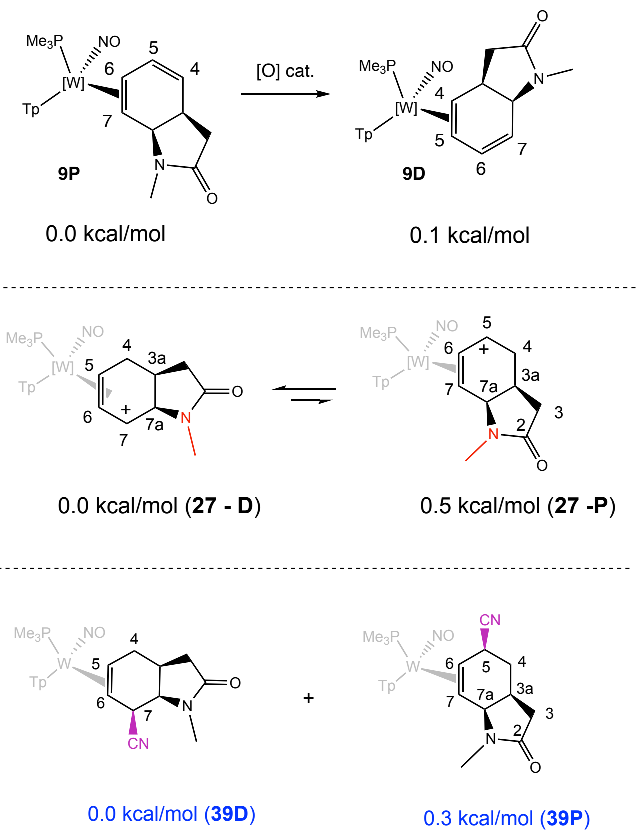

Crystallographic Data

A single crystal of each molecule listed in tables S1-S4 was coated with Paratone oil and mounted on a MiTeGen MicroLoop. The X-ray intensity data for all crystals except **13** were measured on a Bruker D8 Venture dual wavelength Mo/Cu Kappa four-circle diffractometer equipped with a PHOTON III detector and an Oxford Cryostream 800 or 800Plus low-temperature device. An Incoatec IμS 3.0 microfocus sealed X-ray tube (Cu K_{α}, λ=1.54178 Å) and either a HELIOS MX or HELIOS EF double bounce multilayer mirror monochromator were used for **19, 22A, 44, 48**. An Incoatec IμS 3.0 micro-focus sealed X-ray tube (Mo K_{α}, λ = 0.71073 Å) and a HELIOS double bounce multilayer mirror monochromator for were used for **12, 16, 16D, 20, 22B, 22C, 25, 26, 40, 41, 42, 47, 49, 52, 54, 72, 74**. The data for **13** were measured on a Bruker Kappa APEXII Duo system equipped with a fine-focus sealed tube (Mo K_{α}, λ = 0.71073 Å) and a graphite monochromator.

All frames were integrated with the Bruker SAINT software package[4] using a narrow-frame algorithm. Data were corrected for absorption effects using the Multi-Scan method (SADABS[5] or TWINABS (**16D**)[6]. Each structure was solved using the Bruker SHELXT Software Package[7] within either APEX3/APEX4/APEX5/APEX6[4] or OLEX2[8].

For all structures, non-hydrogen atoms were refined anisotropically using SHELXL.[9] The B-H hydrogen atom in each complex was located in the electron density map and freely refined, as were the hydrogen atoms on the carbons bound directly to W in all complexes except **16D, 19, 22A-22C, 47**, and **48**, and the O-H hydrogens in **22A-22C, 26, 42, 44, 45, 52** and **54** All other hydrogen atoms were placed in geometrically calculated positions with U_{iso} = 1.2U_{equiv} of the parent atom (U_{iso} = 1.5U_{equiv} for methyl). Most CIF files were prepared for publication using FinalCif.[10] Additional refinement details are given below.

The relative occupancy of the disordered atoms in **12, 16D, 41, 44** and **47** was freely refined, using constraints and/or restraints on the some of the disordered atoms and bonds. The structure of **16D** was refined as a two-domain twin on HKLF5 data, with the BASF for the twin domains converging at 0.358.

In **41** and **52,** there was severely disordered co-crystallized solvent that could not be adequately modeled with or without constraints. These solvents were accounted for using Platon SQUEEZE.[11] In **52**, void space of 98 Å^3 containing 35 electrons was found. This corresponds to approximately one molecule of dichloromethane in the ASU. In **41**, the void space of 201 Å^3 containing 18 electrons was assigned to an acetone molecule that is present when the minor position of the disordered W complex is occupied.

Table S1: Crystal data for **12, 13, 16, 16D, 19** and **20.**

	**12**	**13**	**16**	**16D**	**19**	**20**
CCDC number	2479766	2479767	2479768	2479769	2479770	2479771
Formula	$C_{28}H_{38}BN_8O_3PSW$	$C_{25}H_{32}BN_8O_3PW$	$C_{25}H_{37}BN_8O_3PW$	$C_{23}H_{32}BN_8O_2PW$	$C_{26}H_{38}BN_8O_4PW$	$C_{22}H_{31}BN_9O_3PW$
FW (g/mol)	792.35	718.21	723.25	678.19	752.27	695.19
Temp (K)	100.00	100.00	100.00	100.00	100.00	100.00

λ (Å)	0.71073	0.71073	0.71073	0.71073	1.54178	0.71073
Size (mm)	0.050 x 0.080 x 0.127	0.078 x 0.197 x 0.201	0.039 x 0.116 x 0.121	0.029×0.053×0.055	0.037×0.04×0.063	0.029 x 0.032 x 0.106
Crystal habit	colorless plate	yellow block	colorless plate	yellow needle	colourless plate	colorless plate
Crystal system	monoclinic	monoclinic	monoclinic	monoclinic	orthorhombic	monoclinic
Space group	P 2_1/c	P 2_1/c	P 2_1/c	P 2_1/n	P bca	P 2_1/c
a (Å)	13.3843(6)	9.5287(7) Å	11.6179(7)	9.5016(8)	20.0579(6)	8.3014(5)
b(Å)	23.9895(10)	16.8147(12)	15.6672(8)	9.0422(7)	13.1448(4)	22.8434(13)
c (Å)	9.8282(4)	17.3887(13)	15.8765(7)	30.722(3)	22.9346(7)	13.9364(8)
α (°)	90	90	90	90	90	90
β (°)	93.3370(10)	92.706(2)	91.465(2)	96.066(2)	90	99.836(2)
γ (°)	90	90	90	90	90	90
Volume ($Å^3$)	3150.3(2)	2782.9(4)	2888.9(3)	2624.7(4)	6046.9(3)	2603.9(3)
Z	4	4	4	4	8	4
Density (g/cm^3)	1.671	1.714	1.663	1.716	1.653	1.773
μ (mm^{-1})	3.828	4.251	4.096	4.499	7.962	4.541
F(000)	1584	1424	1444	1344	3008	1376
θ range (°)	2.24 to 28.31	1.69 to 27.56	2.51 to 25.69	2.18 to 25.37	3.86 to 68.42	2.32 to 27.50
Index ranges	$-17 \leq h \leq 17$<br>$-31 \leq k \leq 31$<br>$-13 \leq l \leq 12$	$-12 \leq h \leq 12$<br>$-21 \leq k \leq 21$<br>$-19 \leq l \leq 22$	$-14 \leq h \leq 13$ $-19 \leq k \leq 19$<br>$-19 \leq l \leq 19$	$-11 \leq h \leq 11$<br>$0 \leq k \leq 10$<br>$0 \leq l \leq 37$	$-21 \leq h \leq 24$<br>$-15 \leq k \leq 15$<br>$-27 \leq l \leq 22$	$-10 \leq h \leq 10$<br>$-29 \leq k \leq 29$<br>$-18 \leq l \leq 18$
Reflns collected	95376	41505	38094	194354	36472	45385
Independent reflns	7821 [R_{int} = 0.0534]	6416 [R_{int} = 0.0626]	5478 [R_{int} = 0.0629]	4807 [R_{int} = 0.1672]	5512 [R_{int} = 0.0906]	5983 [R_{int} = 0.1060]
Data / restraints /parameters	7821 / 2 / 416	6416 / 0 / 367	5478 / 0 / 368	4807 / 5 / 349	5512 / 0 / 380	5983 / 0 / 357
GOF on F^2	1.025	1.046	1.102	1.083	1.038	1.012
R_1 (I>2σ(I))	0.0195	0.0347	0.0398	0.0477	0.0364	0.0350
wR_2 (all data)	0.0448	0.0873	0.0914	0.0934	0.0788	0.0780

Table S2: Crystal data for **22A-22C, 25, 26,** and **40.**

	**22A**	**22B**	**22C**	**25**	**26**	**40**
CCDC number	2479772	2479773	2479774	2479775	2479776	2479777
Formula	$C_{30}H_{40}BN_8O_4PW$	$C_{29}H_{37}BN_9O_3PW$	$C_{28}H_{36}BCl_2N_8O_3PW$	$C_{33}H_{46}BN_8O_6PW$	$C_{27}H_{42}BCl_2N_8O_3PW$	$C_{26}H_{38}BN_8O_6PW$
FW (g/mol)	802.33	785.30	829.18	876.41	823.21	784.27
Temp (K)	100.00	100.00	100.00	100.00	100.00	100.00
λ (Å)	1.54178	0.71073	0.71073	0.71073	0.71073	0.71073
Size (mm)	0.035×0.104×0.147	0.058×0.096×0.149	0.048×0.091×0.153	0.087×0.11×0.125	0.064×0.067×0.073	0.056 x 0.065 x 0.097
Crystal habit	colourless plate	colourless plate	colourless block	colourless block	colourless needle	colorless block
Crystal system	monoclinic	monoclinic	monoclinic	triclinic	triclinic	monoclinic
Space group	P 2_1/c	P 2_1/c	P 2_1/c	P -1	P -1	P 2_1/c
a (Å)	13.4760(5)	13.3840(4)	13.4697(4)	12.9069(5)	10.0201(5)	12.0031(3)
b(Å)	24.0325(9)	24.2740(6)	24.0362(6)	13.1092(6)	13.1562(6)	15.3108(5)
c (Å)	9.8450(4)	9.6958(3)	9.7338(2)	13.8406(6)	13.6352(7)	17.4537(5)
α (°)	90	90	90	110.281(2)	112.735(2)	90

β (°)	93.390(2)	94.5760(10)	94.3460(10)	116.2650(10)	94.796(2)	106.1490(10)
γ (°)	90	90	90	96.791(2)	92.148(2)	90
Volume (Å^3)	3182.8(2)	3139.96(16)	3142.36(14)	1864.30(14)	1647.24(14)	3081.02(16)
Z	4	4	4	2	2	4
Density (g/cm^3)	1.674	1.661	1.753	1.561	1.660	1.691
μ (mm^{-1})	7.609	3.777	3.942	3.194	3.759	3.855
F(000)	1608	1568	1648	884	824	1568
θ range (°)	3.29 to 68.49	2.27 to 25.37	2.26 to 28.31	2.36 to 28.29	2.05 to 28.32	2.21 to 29.59
Index ranges	−16 ≤ h ≤ 16<br>−28 ≤ k ≤ 28<br>−11 ≤ l ≤ 11	−16 ≤ h ≤ 16<br>−27 ≤ k ≤ 29<br>−11 ≤ l ≤ 11	−17 ≤ h ≤ 17<br>−32 ≤ k ≤ 32<br>−12 ≤ l ≤ 12	−16 ≤ h ≤ 17<br>−17 ≤ k ≤ 17<br>−18 ≤ l ≤ 18	−13 ≤ h ≤ 13<br>−17 ≤ k ≤ 15<br>−18 ≤ l ≤ 18	-16 ≤ h ≤ 16<br>-21 ≤ k ≤ 21<br>-24 ≤ l ≤ 22
Reflns collected	37329	128682	171781	81614	56394	63243
Independent reflns	5821 [R_{int} = 0.0576]	5760 [R_{int} = 0.0824]	7806 [R_{int} = 0.0471]	9234 [R_{int} = 0.0448]	8214 [R_{int} = 0.0849]	8614 [R_{int} = 0.0498]
Data / restraints /parameters	5821 / 0 / 419	5760 / 0 / 409	7806 / 0 / 408	9234 / 0 / 471	8214 / 0 / 410	8614 / 0 / 406
GOF on F^2	1.057	1.108	1.052	1.032	1.021	1.008
R_1 (I>2σ(I))	0.0387	0.0257	0.0204	0.0179	0.0350	0.0228
wR_2 (all data)	0.1056	0.0571	0.0529	0.0388	0.0770	0.0512

Table S3: Crystal data for **41, 42, 44, 45, 47** and **48.**

	**41**	**42**	**44**	**45**	**47**	**48**
CCDC number	2479778	2479779	2479780	2479781	2479782	2479783
Formula	$C_{54.14}H_{73.87}B_2N_{16}O_4P_2S_{1.73}W_2$	$C_{23}H_{33}BN_9O_3PW$	$C_{29}H_{40}BN_8O_3PSW$	$C_{25}H_{38}BN_8O_5PSW$	$C_{32}H_{42}BN_8O_4PSW$	$C_{28}H_{40}BN_8O_6PW$
FW (g/mol)	1519.68	709.21	806.38	788.32	860.42	810.31
Temp (K)	100.00	100.00	100.00	100.00	100.00	100.00
λ (Å)	0.71073	0.71073	1.54178	0.71073	0.71073	1.54178
Size (mm)	0.054×0.095×0.141	0.106 x 0.264 x 0.437	0.031 x 0.045 x 0.098	0.058 x 0.061 x 0.106	0.055×0.065×0.192	0.031×0.046×0.053
Crystal habit	colourless plate	yellow block	colorless needle	yellow block	colourless plate	white plate
Crystal system	monoclinic	monoclinic	orthorhombic	triclinic	triclinic	triclinic
Space group	P 2_1/c	P 2_1/c	P b c a	P -1	P -1	P -1
a (Å)	8.3457(2)	13.1605(4)	15.9236(5)	10.1532(4)	11.1724(6)	11.3380(2)
b(Å)	37.5014(10)	11.5282(3)	16.8347(5)	12.5345(6)	12.0571(6)	13.0368(2)
c (Å)	19.8861(6)	19.0565(6)	23.8830(6)	13.0332(6)	13.6472(7)	13.8181(2)
α (°)	90	90	90	82.3060(10)	74.847(2)	63.3890(10)
β (°)	94.3000(10)	104.5070(10)	90	77.6650(10)	78.374(2)	88.9180(10)
γ (°)	90	90	90	70.7070(10)	88.142(2)	68.6850(10)
Volume (Å^3)	6206.3(3)	2799.01(14)	6402.3(3)	1525.78(12)	1737.65(16)	1675.60(5)
Z	4	4	8	2	2	2
Density (g/cm^3)	1.626	1.683	1.673	1.716	1.644	1.606
μ (mm^{-1})	3.871	4.227	8.137	3.956	3.479	7.278
F(000)	3034	1408	3232	788	864	812
θ range (°)	2.13 to 28.30	2.08 to 33.17	3.70 to 68.31	1.73 to 28.29	2.03 to 28.34	3.63 to 68.45

Index ranges	−11 ≤ h ≤ 11<br>−50 ≤ k ≤ 48<br>−26 ≤ l ≤ 26	-20 ≤ h ≤ 20<br>-17 ≤ k ≤ 17<br>-28 ≤ l ≤ 29	-19 ≤ h ≤ 18<br>-20 ≤ k ≤ 15<br>-28 ≤ l ≤ 22	-13 ≤ h ≤ 12<br>-16 ≤ k ≤ 16<br>-17 ≤ l ≤ 17	−14 ≤ h ≤ 14<br>−16 ≤ k ≤ 14<br>−18 ≤ l ≤ 18	−13 ≤ h ≤ 13<br>−15 ≤ k ≤ 15<br>−16 ≤ l ≤ 16
Reflns collected	100381	83221	26983	44728	55445	37279
Independent reflns	15399 [R_{int} = 0.0587]	10677 [R_{int} = 0.0406]	5844 [R_{int} = 0.0873]	7565 [R_{int} = 0.0525]	8639 [R_{int} = 0.1109]	6142 [R_{int} = 0.0835]
Data / restraints /parameters	15399 / 67 / 822	10677 / 0 / 362	5844 / 1 / 381	7565 / 0 / 399	8639 / 0 / 455	6142 / 0 / 415
GOF on F^2	1.085	1.074	1.018	1.049	1.015	1.043
R_1 (I>2σ(I))	0.0387	0.0225	0.0437	0.0256	0.0428	0.0321
wR_2 (all data)	0.0792	0.0427	0.0909	0.0618	0.0954	0.0771

Table S4: Crystal data for **49, 52, 54, 72** and **74**.

	**49**	**52**	**54**	**72**	**74**
CCDC number	2479784	2479785	2479786	2479788	2479789
Formula	$C_{27}H_{36}BCl_2N_{10}O_2PW$	$C_{34}H_{42}BN_8O_3PSW$	$C_{32}H_{46}BN_8O_3PW$	$C_{26}H_{32}BN_8O_2PW$	$C_{61}H_{80}B_2Cl_2N_{18}O_6P_2W_2$
FW (g/mol)	829.19	868.44	816.40	714.22	1683.59
Temp (K)	100.00	100.00	100.00	100.00	100.00
λ (Å)	0.71073	0.71073	0.71073	0.71073	0.71073
Size (mm)	0.07×0.079×0.128	0.035×0.076×0.107	0.089×0.084×0.042	0.035×0.082×0.12	0.026×0.062×0.105
Crystal habit	colourless block	colourless plate	colourless plate	colourless plate	colourless plate
Crystal system	monoclinic	triclinic	monoclinic	orthorhombic	monoclinic
Space group	P 2_1/n	P -1	P 2_1/c	P $2_12_12_1$	P 2_1/c
a (Å)	11.7873(5)	10.9432(4)	15.8125(4)	9.6122(2)	11.5189(4)
b(Å)	12.3160(5)	13.3828(5)	11.4375(3)	12.2291(3)	22.2807(8)
c (Å)	23.2393(8)	14.6510(6)	19.7702(5)	23.4589(6)	14.7446(4)
α (°)	90	110.6030(10)	90	90	90
β (°)	102.6230(10)	93.8430(10)	95.1980(10)	90	110.3830(10)
γ (°)	90	113.0670(10)	90	90	90
Volume ($Å^3$)	3292.2(2)	1794.77(12)	3560.84(16)	2757.56(11)	3547.2(2)
Z	4	2	4	4	2
Density (g/cm^3)	1.673	1.607	1.523	1.720	1.576
μ (mm^{-1})	3.762	3.368	3.333	4.287	3.422
F(000)	1648	872	1648	1416	1684
θ range (°)	2.15 to 28.32	2.07 to 28.28	2.06 to 28.30	1.88 to 30.53	1.89 to 28.30
Index ranges	−15 ≤ h ≤ 15<br>−16 ≤ k ≤ 16<br>−30 ≤ l ≤ 26	−14 ≤ h ≤ 13<br>−17 ≤ k ≤ 17<br>−19 ≤ l ≤ 19	−18 ≤ h ≤ 21<br>−15 ≤ k ≤ 15<br>−26 ≤ l ≤ 26	−10 ≤ h ≤ 13<br>−17 ≤ k ≤ 17<br>−33 ≤ l ≤ 33	−14 ≤ h ≤ 15<br>−29 ≤ k ≤ 29<br>−19 ≤ l ≤ 17
Reflns collected	66790	64453	65080	66392	56460
Independent reflns	8185 [R_{int} = 0.0707]	8909 [R_{int} = 0.0797]	8840 [R_{int} = 0.0602]	8439 [R_{int} = 0.0411]	8828 [R_{int} = 0.0799]
Data / restraints /parameters	8185 / 0 / 412	8909 / 0 / 461	8840/1/437	8439 / 0 / 368	8828 / 0 / 446
GOF on F^2	1.021	1.013	0.995	1.055	1.040
R_1 (I>2σ(I))	0.0300	0.0303	0.0256	0.0200	0.0421

wR_2 (all data)	0.0686	0.0647	0.0596	0.0475	0.0922

Supplementary References

[1] Simpson, S. R.; Siano, P.; Siela, D. J.; Diment, L. A.; Song, B. C.; Westendorff, K. S.; Ericson, M. N.; Welch, K. D.; Dickie, D. A.; Harman, W. D., Phenyl Sulfones: A Route to a Diverse Family of Trisubstituted Cyclohexenes from Three Independent Nucleophilic Additions. *Journal of the American Chemical Society* **2022,** *144* (21), 9489-9499.

[2] Siano, P.; Diment, L. A.; Siela, D. J.; Ericson, M. N.; McGraw, M.; Livaudais, B. F.; Dickie, D. A.; Harman, W. D., An organometallic approach to the synthesis of heteropolycyclic compounds from benzenes. *Nature Communications* **2025,** *16* (1), 7504.

[3] Smith, J. A., Scouten, A., Wilde, J.H., Westendorff, K.W., Dickie, D.A., Ess, D.H., Harman, W.D. Experiments and Direct Dynamics Simulations That Probe η2-Arene/Aryl Hydride Equilibria of Tungsten Benzene Complexes. *J. Am. Chem. Soc.* **2020**, *142*, 16437-16454

[4] Bruker (2019). *Saint; APEX3, APEX4, APEX5, APEX6*. Bruker AXS Inc., Madison, Wisconsin, USA.

[5] Krause, L.; Herbst-Irmer, R; Sheldrick, G. M.; Stalke, D., Comparison of silver and molybdenum microfocus X-ray sources for single-crystal structure determination. *J. Appl. Cryst.* **2015**, *48*, 3-10. doi:10.1107/ S1600576714022985

[6] Sevvana, M.; Ruf, M.; Usón, I.; Sheldrick, G. M.; and Herbst-Irmer, R., Non-merohedral twinning: from minerals to proteins. *Acta Crystallogr. Sect. D: Struct. Biol.* **2019**, *D75*, 1040-1050. doi:10.1107/S2059798319010179

[7] Sheldrick, G. M., SHELXT – Integrated space-group and crystal structure determination. *Acta Crystallogr. Sect. A: Found. Adv.* **2015**, *A71*, 3-8. doi:10.1107/S2053273314026370

[8] Dolomanov, O. V.; Bourhis, L. J.; Gildea, R. J.; Howard, J. A. K.; Puschmann, H., OLEX2: a complete structure solution, refinement and analysis program. *J. Appl. Cryst.* **2009**, ***42***, 339-341. doi:10.1107/S0021889808042726

[9] Sheldrick, G. M., Crystal structure refinement with SHELXL. *Acta Crystallogr. Sect C: Struct. Chem.* **2015**, *C71*, 3-8. doi: 10.1107/S2053229614024218

[10] Kratzert, D. FinalCif, https://dkratzert.de/finalcif.html.

[11] Spek, A. L., PLATON SQUEEZE: a tool for the calculation of the disordered solvent contribution to the calculated structure factors. *Acta Crystallogr. Sect C: Struct. Chem.* **2015**, *C71*, 9-18. doi: 10.1107/S2053229614024929
